# Stereoselective Synthesis and Functionalization of Acetylenic Boronic Esters

**DOI:** 10.1021/acs.orglett.6c01093

**Published:** 2026-04-20

**Authors:** Patrick Schäfer, Uli Kazmaier

**Affiliations:** 9379 Saarland University, Organic Chemistry I, Campus, Building C4.2, D-66123 Saarbrücken, Germany

## Abstract

Hydrozirconation of homopropargyl boronic esters, readily available by Matteson homologation, allows their selective functionalization while preserving the boronic ester functionality. Metal–halogen exchange yields reactive vinyl iodides, which can be further converted into functionalized alkenes via Negishi couplings and Heck reactions. These reactions are used to generate polyketide-like structures, which are relevant for the synthesis of natural products.

## Graphical abstract

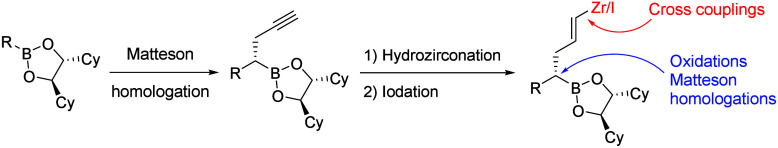

Among the natural products, the structurally very diverse class of polyketides plays an important role, especially due to their interesting biological activities.^1^ In many of these natural products, regions with a high density of stereogenic centers are separated by areas with (conjugated) double bonds, for example, α,β-unsaturated ketones or esters. Typical examples are (5*Z*)-7-oxozeaenol^2^ and efomycine M (Figure 1), which show interesting biological properties.^3^ (5*Z*)-7-Oxozeaenol is cytotoxic against a panel of human tumor cell lines and shows NF-KB inhibitory activity in a submicromolar range,^2^ while efomycine exhibits potent immunosuppressive activity.^3^ Biosynthetically, these structural motifs are formed from the corresponding β-hydroxycarbonyl compounds via water elimination.^4^

**Figure 1:**
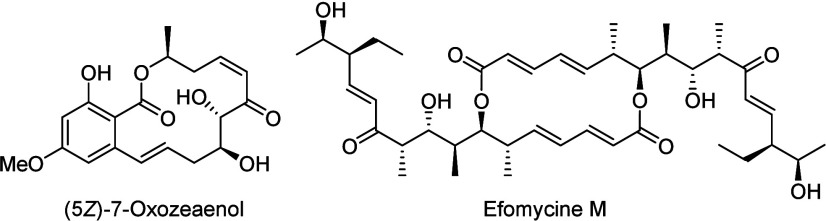
Polyketide natural products.

In view of the interesting biological activities, there is great interest in synthetic methods for the synthesis of this class of substances, because asymmetric total synthesis in particular is often anything but trivial.^5^ In most cases, the polyketide chain is obtained either via aldol reactions^6^ or by allylations/crotylations followed by ozonolysis.^7^ Both methods, the aldol addition and the allylation/ozonolysis approach, allow the construction of polyketide chains with OH groups at positions 3, 5, 7, ... and H/Me at positions 2, 4, 6, ..., but are less suitable for generating substitution patterns that deviate from them. In contrast, the Matteson homologation^8^ is particularly suitable for this purpose, especially since an asymmetric version was reported by Donald Matteson in the early 1980s (Scheme 1A).^9^

**Scheme 1:**
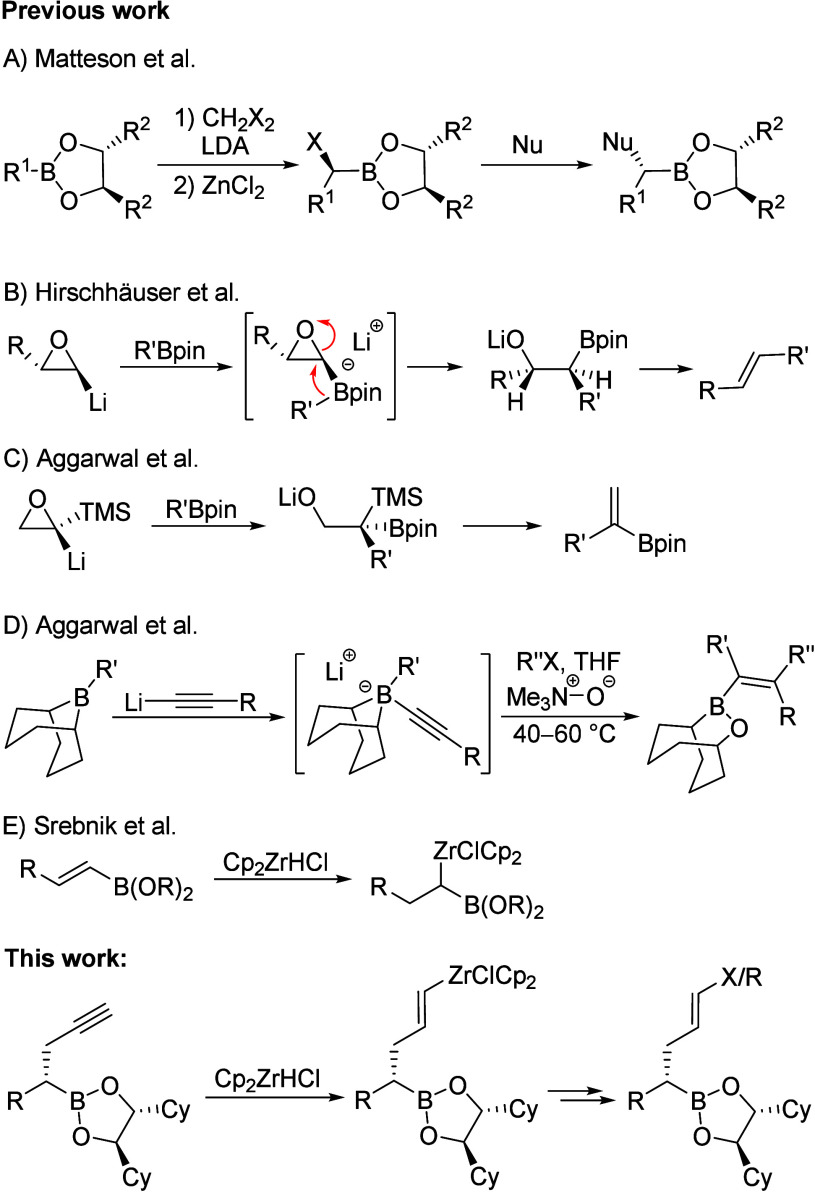
Previous Work on Matteson Homologation and New Application

For example, using alkylboronic esters of chiral diols, the alkyl chain can be extended via a reaction with deprotonated CH_2_Cl_2_ or CH_2_Br_2_ (lithium carbenoids). This process is highly stereoselective and is controlled almost exclusively by the chiral auxiliary (see Supporting Information).^8^ The α-haloboronic esters formed in this process can then be reacted under S_N_2 conditions with a variety of nucleophiles, e.g. Grignard reagents, enolates, or alkoxides. Thus, each carbon atom of the chain can be substituted individually, whereby already existing stereogenic centers have little effect on the generation of a new center. As a rule, 1,2-*anti*-configured products are formed preferentially, but the configuration of alkyl groups can be inverted, e.g. by a suitable selection of the lithium carbenoid, providing also 1,2-*syn* substituted products.^10^ Therefore, Matteson homologation is increasingly used in natural product^11^ and drug synthesis^12^ and has also aroused our interest.^13^

While the syntheses of densely functionalized regions are not a serious issue for this reaction, the incorporation of unsaturations into a carbon chain represents a significant obstacle to Matteson homologations. In 2019, Hirschhäuser and colleagues reported the conversion of achiral boronic esters into alkenes by insertion of lithiated epoxides (Scheme 1B).^14^ Subsequent thermal *syn*-elimination yields stereoselectively di- and trisubstituted alkenes. Mamane, Pale and co-workers applied the same principle to alkynyl-substituted oxiranes, resulting in 1,3-enynes.^15^ But in both reactions, the boronic ester is split off in the elimination step and is therefore no longer available for further transformations. In contrast, Aggarwal and co-workers were able to show that lithiated epoxysilanes are suitable for the synthesis of 1,1-disubstituted vinyl boronic esters, as the silane is preferentially cleaved off in the elimination step (Scheme 1C).^16^ Only recently, the same group reported on a boron-mediated assembly of tetrasubstituted alkenes by adding acetylides to alkylboranes (Scheme 1D).^17^ In the presence of electrophiles, the migrating group and electrophile add *syn* to the alkyne, generating the tetrasubstituted alkene in a highly stereoselective fashion.

We decided to choose an alternative approach based on cross-couplings. The goal was to couple two fragments with a high density of stereocenters, which were preferentially obtained by Matteson homologation. For this purpose, it was necessary to convert (complex) boronic esters into either metalated or halogenated vinyl substrates. We chose the appropriate vinyl zirconium compounds, which are easily obtained by hydrozirconation of terminal alkynes.^18^ Furthermore, Srebnik et al. were able to show that the two metals are compatible in one and the same molecule applying the hydrozirconation to vinyl boranes^19^ and vinyl boronic esters^20^ (Scheme 1E).

Vinyl zirconium reagents resulting from the hydrozirconation of alkynes can be functionalized either by reactions with electrophiles such as protons, halides, or acid chlorides^21^ or by Ni- or Pd-catalyzed Negishi couplings of a wide range of electrophiles.^22^ They are therefore common tools for the synthesis of unsaturated natural products.^23^

Initially, the homopropargyl boronic esters used as precursors for the hydrozirconation of alkynes were synthesized from boronic ester **1** via Matteson homologation and subsequent nucleophilic substitution with the corresponding TMS-protected propargylzinc reagent (Scheme 2).^24^ The diastereomerically pure boronic ester **2** was then deprotected with K_2_CO_3_ in methanol/ether^25^ to form boronic ester **3**, whereby these conditions yielded the best results (see Supporting Information). The addition of diethyl ether was necessary in this case because of the poor solubility of **2** in methanol, and even under these conditions, the reaction was rather slow.

**Scheme 2:**
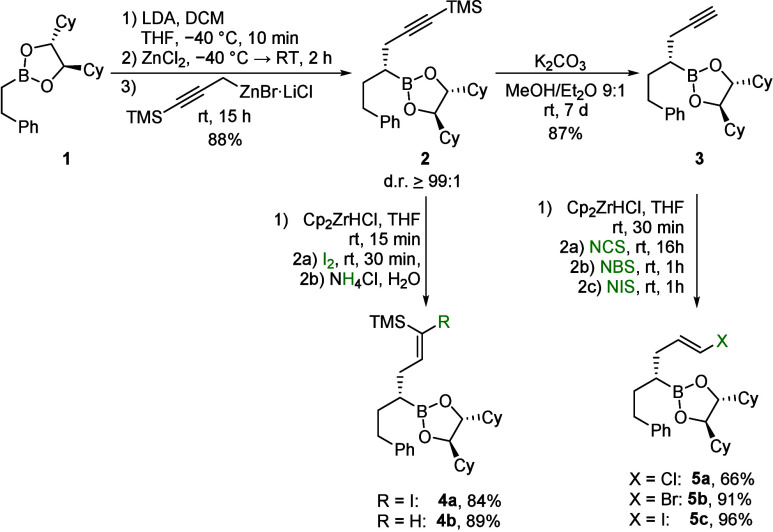
Synthesis and Hydrozirconation/Halogenation of Precursors **2** and **3**

The first hydrozirconations focused on the addition of Schwartz reagent to the protected boronic ester **2**, followed by the addition of iodine, resulting in the formation of boronic ester **4a**. Via mild acidic hydrolysis of the zirconium intermediate, the (*Z*)-alkene **4b** was obtained.^26^ The deprotected alkyne **3** could also be subjected to hydrozirconation. Subsequent addition of *N*-halosuccinimides led to the formation of the desired (*E*)-vinyl halides **5**. The iodide **5c** was obtained in a comparable yield to the bromide **5b**, while the chloride **5a** required a much longer reaction time and the yield was significantly lower.

The vinyl iodide **5c** obtained is an excellent substrate for the Pd-catalyzed cross-couplings, giving rise to (*E*)-configured homoallyl boronic esters **6**. Therefore, the reaction of **5c** in Negishi couplings with various alkyl and arylzinc reagents was investigated (Scheme 3). The required zinc reagents were obtained via transmetalation of the corresponding Grignard reagents or lithium organyls.^26^ Both electron-rich and electron-poor aromatics gave excellent results (**6a**–**6h**), as do the corresponding heterocycles (**6j**–**6l**). Benzyl, vinyl and even alkyl zinc reagents were also suitable as coupling partners (**6i,m,n**); only with the sterically demanding *tert*-butyl reagent, no cross-coupling product **6o** was observed. Instead, an allyl-substituted boronic ester (R = H) was formed. This indicates that, although the oxidative addition of the vinyl iodide and the transmetalation of the organozinc reagent to the palladium occurred, β-hydride elimination was obviously faster than reductive elimination. Therefore, hydride and not the *tert*-butyl group was transferred.

**Scheme 3:**
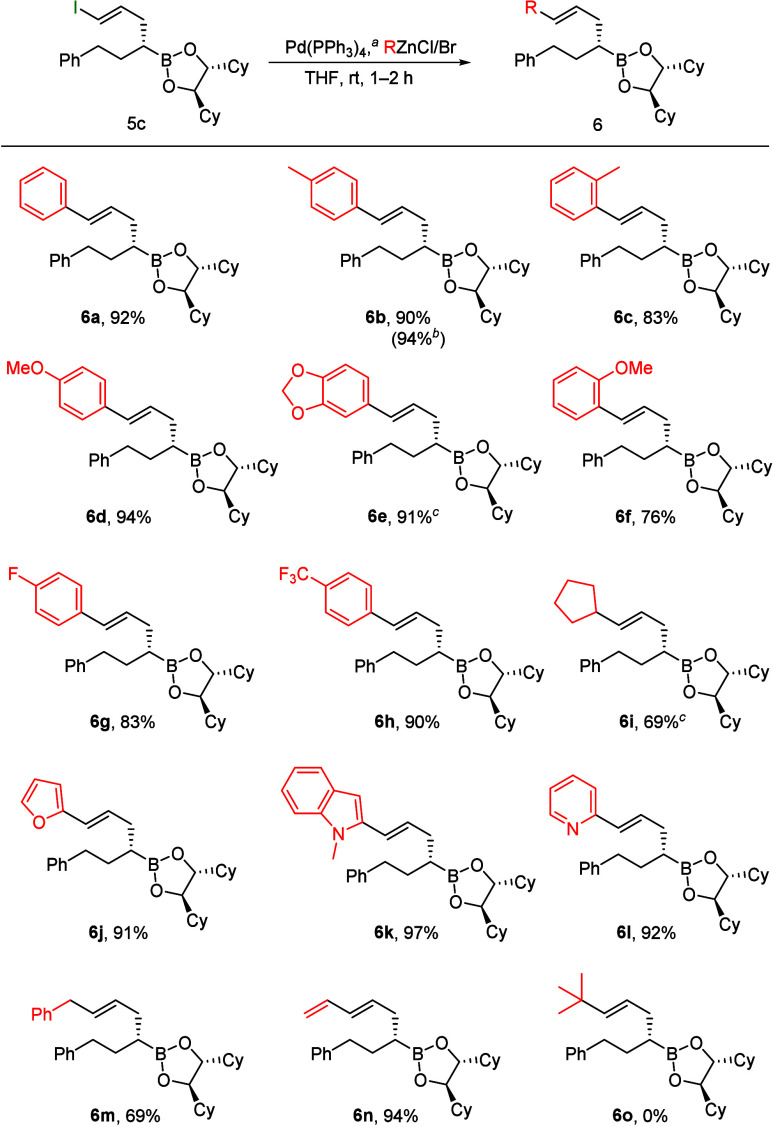
Negishi Coupling of Vinyl Iodide **5c**

a 10 mol % catalyst.
b Yield of 1 mmol-scale experiment.
c Boronic ester **6e** and **6i** were also oxidized to the respective alcohols **7** (**7e**, 89%, **7i**, 64%).

For analytical purposes, some of the boronic esters **6** were also oxidized to the corresponding homoallylic alcohols, because in the case of **6e** and **6i** some side products could not be separated by flash chromatography.

Next, we were interested in Heck reactions^27^ of vinyl iodide **5c** with unsaturated carbonyl compounds (Scheme 4). The Heck coupling was successful not only with methyl acrylate but also with acrylonitrile. However, while methyl acrylate cleanly yielded the (*E*,*E*)-configured product **8a**, the corresponding nitrile **8b** was obtained as a 2:3 mixture of (*E*,*E*) and (*E*,*Z*) isomer, probably due to the lower steric demand of the nitrile group. Ethyl crotonate could also be successfully converted stereoselectively to **8c**, whereby the configuration of the newly formed (*E*)-double bond was determined by 1D-NOESY. Similarly, **8d** could be obtained using (*Z*)-ethyl crotonate via stereospecific carbopalladation and subsequent β-hydride elimination. Interestingly, the corresponding product was not formed with methyl methacrylate. In this case, four unidentifiable boronic esters were formed under the usual reaction conditions.

**Scheme 4:**
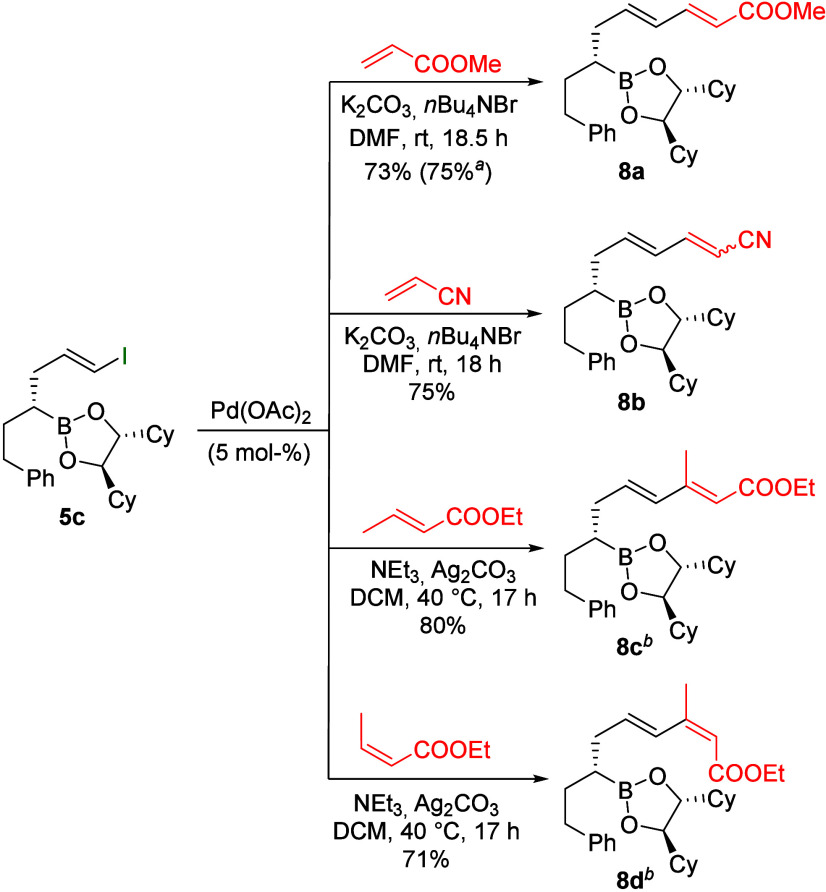
Heck Reactions of Vinyl Iodide **5c**

a Yield of 1 mmol-scale experiment.
b 10 mol % catalyst.

After the stereoselective synthesis of (*E*)-alkenes, the stereospecific synthesis of a trisubstituted alkene was also attempted (Scheme 5). Starting from vinyl iodide **4a**, boronic ester **9** was obtained in a Negishi coupling with MeMgBr.

**Scheme 5:**
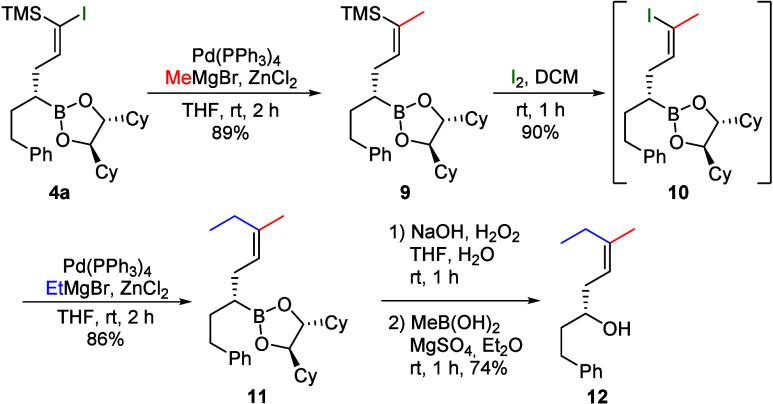
Synthesis of Trisubstituted Alkene **12**

Vinylsilane **9** could be converted into vinyl iodide **10** via desilylative iodination and subsequently subjected to another Negishi coupling.^26^ The trisubstituted (*Z*)-boronic ester **11** formed was oxidized directly to alcohol **12** to facilitate chromatographic purification. By reversal of the Negishi coupling steps, it should also be possible to obtain the (*E*)-configured product without any problems.

Next, we wanted to show the applicability of this method to generate more complex structures, such as those found in natural products.^28^ Therefore, boronic esters containing protected OH functionalities, such as boronic esters **13**, were also used (Scheme 6). Here, too, the introduction of the propargyl residue took place under the usual reaction conditions with an excellent yield. The cleavage of the silyl protection group and the subsequent hydrozirconation/iodination to vinyl iodide **15** also proceeded without any problems.

**Scheme 6:**
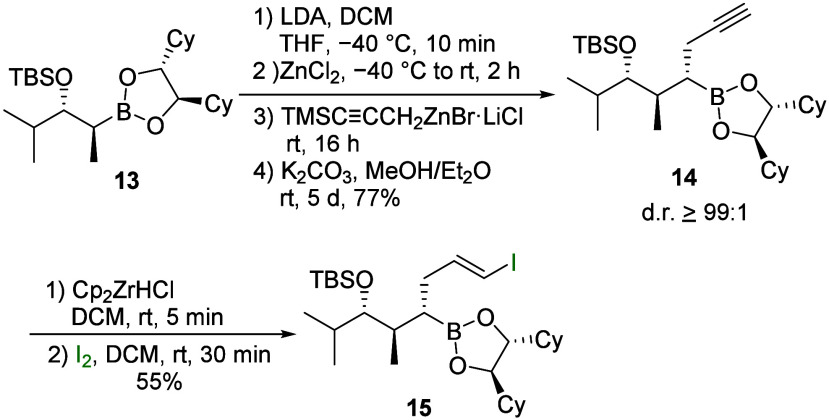
Synthesis of Vinyl Iodide **15**

a Yield of 1 mmol-scale experiment.

In a second example, starting from boronic ester **1**, the *p*-methoxybenzyl-protected alcohol was first introduced and then the silylated propargyl residue (Scheme 7) was introduced in the immediate vicinity.

**Scheme 7:**
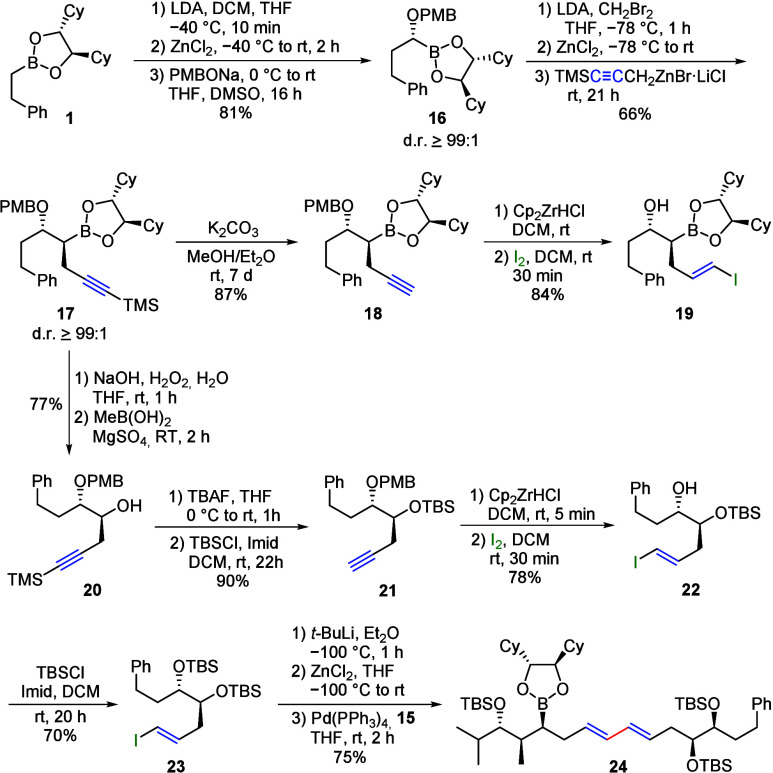
Synthesis of Vinyl Iodide **19** and Conjugated Diene **24**

As has often been observed in the past, nucleophilic substitution in the neighborhood of oxygen proceeded much more slowly and in sometimes moderate yields.^29^ Thus, under classical conditions with the lithium carbenoid, even after more than 2 days of reaction time, only a yield of about 50% could be achieved for **17**. However, this could be significantly improved and accelerated by using the corresponding bromine derivative.^30^ Subsequently, the terminal alkyne was deprotected to **18** and subjected to hydrozirconation/iodination. Surprisingly, the PMB protection group was also cleaved in this step. It is well-known that the PMB protection group can be split off with TMSI.^31^ Obviously, this also works with other Lewis acids in combination with the iodide. In the present case, Cp_2_ZrICl is formed during the metal–halogen exchange, which obviously causes the same cleavage. Therefore, these reactions should allow an easy exchange of the *O*-protection group, or the installation of e.g. silyl protecting groups, which cannot be introduced directly by Matteson homologation.^32^

If the silylated propargylboronic ester **17** was oxidized, the monoprotected diol **20** was selectively obtained, which can be individually protected or coupled at the newly formed OH group. For example, the reaction first with TBAF and then with TBSCl yielded the orthogonally protected terminal alkyne **21**. Hydrozirconation/iodination also yielded vinyl iodide **22** with a free OH group, as described before. This was also TBS-protected as an example (**23**). In order to couple the two polyketide fragments **15** and **23**, **23** was transferred to the corresponding organozinc reagent. This was achieved by halogen–metal exchange with *tert*-butyllithium, followed by transmetalation to zinc. After the subsequent addition of **15**, a Pd-catalyzed cross-coupling yielded polyketide-like boronic ester **24** in good yield.

In conclusion, we have shown that the hydrozirconation of homopropargyl boronic esters allows the selective functionalization of carbon chains obtained via Matteson homologation. The vinyl zirconium compounds formed can be converted to the corresponding vinyl halides. Vinyl iodides, in particular, are very suitable substrates for Negishi couplings with organozinc reagents or Heck reactions. The method also allows the stereoselective assembly of triple-substituted alkenes. Furthermore, the protocol can be used to join two fragments via 1,3-diene when one of the vinyl iodide components is converted into the corresponding zinc reagent via a halogen–metal exchange. Applications of this method in total syntheses are currently under investigation.

## Supplementary Material



## Data Availability

The data underlying this study are available in the published article and its Supporting Information.

## Notes

Detailed experimental procedures, NMR data and copies of ^1^H and ^13^C NMR spectra. (PDF)
