# Supplementary material for: Stereoselective Synthesis and Functionalization of Acetylenic Boronic Esters
Source: Org Lett. 2026 Apr 20;28(17):5477–82. doi: 10.1021/acs.orglett.6c01093 (PMC13140146; doi:10.1021/acs.orglett.6c01093)
Supplement: Supplementary file 1 [file ol6c01093_si_001.pdf]

Stereoselective Synthesis and Functionalization of Acetylenic Boronic Esters

Patrick Schäfer[a] and Uli Kazmaier[a]*

Organic Chemistry I, Saarland University, Campus, Building C4.2

D-66123 Saarbrücken, Germany

E-Mail: u.kazmaier@mx.uni-saarland.de

Supporting information

Table of contents

General information S2

General procedures S3

Test reactions and optimizations S5

Deprotection of alkyne 2 .... S5

Synthesis of compounds S6

Copies of NMR spectra S35

References S120

Assigning the absolute configuration of the Matteson homologation products S121

General information

All air and moisture sensitive reactions were conducted in dried glassware (>100 °C) under a nitrogen atmosphere if not stated otherwise. Dried solvents were distilled prior to use: THF was distilled from sodium/benzophenone and *N*,*N*-diisopropylamine was dried over CaH_2 before distillation. All other anhydrous solvents were purchased from *Thermo Scientific* and stored under nitrogen atmosphere over molecular sieves. Pentane and ethyl acetate were distilled prior to use. Column chromatography was performed using the *Büchi Pure C-815 Flash* system with *Teledyne Isco RediSep Rf Silver Silica* normal-phase columns (30-70 µm) with mixtures of cyclohexane and ethyl acetate as eluent. Reverse-phase chromatography was performed using the *Büchi Reveleris Prep* system with *Büchi FlashPure Select C18* 30 µm spherical cartridges with mixtures of water and acetonitrile as eluent. For analytical TLC, the precoated silica-gel plates *Polygram Sil G/UV$_{254}$* by *Macherey-Nagel* were used. Detection was accomplished with UV light (λ = 254 nm) and cerium(IV)/ammonium molybdate solution. ^{1}H-, ^{13}C- and ^{19}F-NMR spectra were measured on a *Bruker Advance II* 400 MHz spectrometer (^{1}H: 400 MHz, ^{13}C: 100 MHz, ^{19}F: 377 MHz), a *Bruker Advance I* 500 MHz spectrometer (^{1}H: 500 MHz, ^{13}C: 125 MHz) or a *Bruker Advance Neo* 500 MHz spectrometer (^{1}H: 500 MHz, ^{13}C: 125 MHz). Chemical shifts (δ) are reported in parts per million (ppm) relative to the internal solvent signal (7.26 ppm for $CDCl_3$ as NMR solvent) for ^{1}H- and ^{13}C-NMR and to a tertiary reference (−61.62 ppm for benzotrifluoride or −75.39 ppm for trifluoroacetic acid) for ^{19}F-NMR. The peaks were assigned using ^{1}H,^{1}H-COSY, ^{1}H,^{13}C-HSQC and ^{1}H,^{13}C-HMBC spectra. NMR spectra were evaluated using ACD Labs NMR Processor Version 12.01 or *Bruker Top Spin* Version 4.3.0. Mass spectra were recorded with a *Finnigan MAT95* spectrometer (quadrupol) using the CI technique or an *Orbitrap Q* exactive mass spectrometer, equipped with an heated ESI source and an quadrupole-orbitrap coupled mass detector and an *Ultimate3000* HPLC (Thermo Finnigan, San Jose, CA). The MS detection was carried out at a spray voltage of 3.8 kV in positive ionisation mode, a nitrogen sheath gas pressure of $4.0\cdot10^{-5}$ Pa, an auxiliary gas pressure of $1.0\cdot10^{-5}$ Pa and a capillary temperature of 300 °C. All samples were injected by autosampler with an injection volume of 15 µL. A *RP Nucleoshell Phenyle-hexyle*® (50-2, 3.0µm) column (Macherey-Nagel GmbH, Duehren, Germany) was used as stationary phase. The solvent system consisted of formic acid 0.1% (A) and acetonitrile with formic acid 0.1% (B). HPLC method: flow rate 600 µL/min. The percentage of B started at an initial of 1%, was kept at 1% for 1.5 min, then rapidly increased up to 100 % during 4.0 min, then kept at 100% until 1.5 min and flushed back to the initial 1 %. Xcalibur software was used for data acquisition and plotting. Optical rotations were measured with a *Krüss P8000-T* polarimeter in a thermostat-controlled cuvette (20.0 ± 0.1 °C) using a sodium vapour lamp (λ = 589 nm) as radiation source. $[\alpha]_D^{20}$ values are given in 10^{-1} deg·cm^{2}·g^{-1} and c is given in g·cm^{-3}.

General procedures

GP-1: Matteson homologation

To a solution of *N*,*N*-diisopropylamine (1.35 eq.) in anhydrous THF (0.2 mL/mmol) was added *n*-BuLi (1.25 eq., 1.6 M in hexanes) dropwise at a temperature between −40 °C and −50 °C. The reaction mixture was stirred for 20 min at room temperature. The freshly prepared LDA solution was added slowly at a temperature between −40 °C and −50 °C to a solution of the boronic ester (1.0 eq.) and anhydrous DCM (3.0 eq.) in anhydrous THF (1.4 mL/mmol).[1] After 10 min of stirring at the same temperature, a solution of $ZnCl_2$ (2.0–3.0 eq., flame dried *in vacuo*) in anhydrous THF (0.6 mL/mmol $ZnCl_2$) was added and the reaction was stirred for 2 h at room temperature before adding the nucleophile solution according to the respective variant.

After complete conversion, saturated NH_4Cl solution was added to the reaction mixture. The biphasic mixture was stirred for 5 min before separating the phases. The aqueous phase was extracted twice with pentane. The combined organic phases were dried over Na_2SO_4 and filtered. The solvent was removed under reduced pressure. The crude product was purified by column chromatography.

Variant A) Substitution with organozinc reagent

Organozinc reagent was prepared by suspending LiCl (1.1 eq., flame dried *in vacuo*) and zinc dust (2.0 eq.) in anhydrous THF (1.0 M) and adding 1,2-dibromoethane (2 mol-%). The reaction mixture is carefully heated with a heat gun to gentle boiling. After cooling to room temperature, trimethylsilyl chloride (5 mol-%) is added and the reaction mixture is again heated with a heat gun to gentle boiling. After cooling to room temperature, a solution of the alkyl bromide (1.0 eq.) in anhydrous THF (1.0 M) was added dropwise. An exothermic reaction could be observed. After 1 h excess zinc dust was allowed to settle and the supernatant solution was directly used in the next step. The concentration of the organozinc solution was determined by iodometric titration.[1]

The reaction mixture resulting from homologation was cooled to 0 °C and the freshly prepared organozinc solution was added dropwise. The reaction mixture was stirred at room temperature until complete conversion indicated by ^{1}H-NMR analysis.

Variant B) Substitution with Grignard reagent

The reaction mixture was cooled to 0 °C and the solution of the Grignard reagent (2.5 eq.) was added slowly. The reaction mixture was stirred at room temperature until complete conversion indicated by ^{1}H-NMR analysis.

Variant C) Substitution with sodium alcoholate

To a suspension of sodium hydride (1.3 eq., 60% in mineral oil) in anhydrous THF (0.5 mL/mmol) and anhydrous DMSO (1.3 mL/mmol) was added the respective alcohol (1.4 eq.). the reaction mixture was stirred for 6 – 7 h at room temperature.

The reaction mixture resulting from homologation was cooled to 0 °C and the alcoholate solution was added slowly. The reaction mixture was stirred at room temperature until complete conversion indicated by ^{1}H-NMR analysis.

---

[1] The LDA solution should be added very carefully by direct addition to the reaction mixture. If this is done too fast, the reaction mixture turns from intransparently dark brown to black. In this case, the reaction mixture should be discarded. If done correctly, the reaction mixture turns yellow to orange. When adding the $ZnCl_2$ solution, the intensity decreases slightly and returns after 2 h of stirring at room temperature.

GP-2: Hydrozirconation and Negishi cross-coupling with alkyl- and arylzinc reagents

The organozinc reagent was prepared by dropwise addition of the respective organolithium or Grignard reagent (10.0 eq.) to a solution of $ZnCl_2$ (10.0 eq., flame dried *in vacuo*) in anhydrous THF (1.75 mL/mmol) at 0 °C. the reaction mixture was allowed to warm to room temperature and was stirred for 1 h. The resulting solids were allowed to precipitate. The supernatant solution was directly used in the next step.

Vinyl iodide (1.0 eq.) and $Pd(PPh_3)_4$ (0.1 eq.) were dissolved in anhydrous THF (0.05 M). after adding half of the volume of the organozinc solution, the reaction mixture was stirred for 1–2 h until complete conversion indicated by ^{1}H-NMR analysis.

After complete conversion, water was added to the reaction mixture and the phases were separated. The aqueous phase was extracted three times with ethyl acetate. The combined organic phases were washed twice with water and once with brine. The organic phase was dried over Na_2SO_4 and filtered. The solvent was removed under reduced pressure and the crude product was purified by column chromatography.

GP-3: Oxidation of boronic esters to alcohols

At 0 °C H_2O_2 (5.0 eq., 33% in water) and a solution of NaOH (5.0 eq.) in water (2 mL/mmol) were added to a solution of the boronic ester (1.0 eq.) in THF (2 mL/mmol). The reaction mixture was warmed to room temperature and stirred at the same temperature until complete conversion indicated by TLC. The reaction mixture was then washed with brine and the aqueous phase was extracted three times with Et_2O. the combined organic phases were dried over Na_2SO_4 and filtered. The solvent was removed under reduced pressure. To improve separation of the alcohol and DICHED, the crude product was diluted in Et_2O (5 mL/mmol) and methylboronic acid (1.2 eq.) was added with an excess of $MgSO_4$ and the reaction mixture was stirred for 2 h at room temperature. The reaction mixture was filtered and the solvent was removed under reduced pressure. The crude product was purified by column chromatography.

Test reactions and optimizations

Deprotection of alkyne 2

Since the direct introduction of the unprotected propargyl substituent via Matteson homologation was not successful, the TMS protected substituent was used. To afford the terminal alkyne **3**, **2** had to be deprotected first. Fluoride-containing reagents as TBAF or KF are not compatible to the boronic ester functionality because the fluoride rather binds to the boron rather than the silicon leading to at least partial protodeboration.[2,3] The same could be observed when trying deprotection with TBAF (entry 1).[4] The attack on the boronic ester functionality was indicated by the formation of DICHED during the reaction. So, different deprotection conditions were tested. First, deprotection with potassium carbonate (entry 2)[5] was tested which showed to be the best method despite the relatively long reaction time because of complete conversion and a high isolated yield (87%). When sodium methylate was used as base, no reaction could be observed (entry 3).[6] The conditions in entry 4[7] offered the shortest reaction time but also a low isolated yield.

Tab. S1. Tested conditions for the deprotection of alkyne **2**.

Entry	Reagent	Solvent	Temperature	Time	Commentary
**1**	TBAF	THF	RT	1 h	Attack on boronic ester
**2**	K_2CO_3	$MeOH/Et_2O$ 9:1	RT	7 d	Complete conversion
**3**	NaOMe	$MeOH/Et_2O$ 9:1	RT	24 h	No reaction
**4**	$AgNO_3$, 2,6-lutidine	$THF/H_2O/EtOH$ 1:1:1	RT	1 h	Complete conversion, low isolated yield (24%)

Synthesis of compounds

((*R*)-4-((4*R*,5*R*)-4,5-Dicyclohexyl-1,3,2-dioxaborolan-2-yl)-6-phenylhex-1-yn-1-yl)trimethylsilane (2)

According to GP-1, 1.72 mL (1.22 g, 12.1 mmol) *N*,*N*-diisopropylamine, 6.98 mL (11.2 mmol, 1.6 M) *n*-butyllithium and 1.79 mL anhydrous THF were used for the LDA solution, 2.44 g (17.9 mmol) zinc chloride and 10.7 mL anhydrous THF were used for the $ZnCl_2$ solution and 3.04 g (8.93 mmol) boronic ester **1**,[8] 1.73 mL (2.28 g, 26.8 mmol) anhydrous DCM and 12.5 mL anhydrous THF were used for the homologation.

The organozinc reagent was prepared according to variant A using 2.10 g (49.5 mmol) LiCl, 5.88 g (90.0 mmol) zinc dust, 78.0 µL (0.90 mmol) 1,2-dibromoethane, 288 µL (2.25 mmol) trimethylsilyl chloride, 45.0 mL anhydrous THF and a solution of 7.35 mL (45.0 mmol) (3-bromoprop-1-yn-1-yl)trimethylsilane in 45.0 mL anhydrous THF. The reaction mixture was worked up after 15 h. After column chromatography (SiO_2, CyH/EtOAc 0-3%), the product **2** (3.65 g, 7.86 mmol, 88%) was obtained as a colorless oil. R_f (**2**) = 0.34 (pentane/EtOAc 97:3). $[\alpha]_D^{20}$ = +33.8 (c = 1.0, $CHCl_3$).

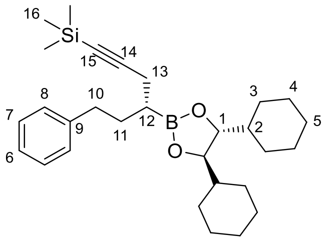

^{1}H-NMR (400 MHz, $CDCl_3$) δ: 7.30–7.26 (m, 2 H, 7-H), 7.21–7.16 (m, 3 H, 6-H, 8-H), 3.89–3.86 (m, 2 H, 1-H), 2.66 (m, 2 H, 10-H), 2.46–2.33 (m, 2 H, 13-H), 1.88–1.61 (m, 12 H, 3-Ha, 3-Hb, 4-Ha, 4-Hb, 5-Ha, 11-H), 1.37–0.97 (m, 13 H, 2-H, 3-Hc, 3-Hd, 4-Hc, 4-Hd, 5-Hb, 12-H), 0.14 (s, 9 H, 16-H).

^{13}C-NMR (100 MHz, $CDCl_3$) δ: 142.8 (C-9), 128.4 (C-8), 128.2 (C-7), 125.6 (C-6), 107.2 (C-14), 84.7 (C-1), 83.5 (C-15), 43.0 (C-2), 34.9 (C-10), 32.3 (C-11), 28.3 (C-3a), 27.4 (C-3b), 26.5 (C-5), 26.0 (C-4a), 25.9 (C-4b), 21.1 (C-13), 0.2 (C-16). The signal of C-12 could not be detected.

HRMS (CI) *m/z*: $[M]^+$ calcd for $C_{29}H_{45}BO_2Si$, 464.3282; found, 464.3312.

(4*R*,5*R*)-4,5-Dicyclohexyl-2-((*R*)-1-phenylhex-5-yn-3-yl)-1,3,2-dioxaborolane (3)

To a solution of 2.58 g (5.55 mmol, 1.0 eq.) boronic ester **2** in 76.9 mL methanol/diethyl ether (9:1 v/v, 0.07 M) was added 1.53 g (11.1 mmol, 2.0 eq.) K_2CO_3. The reaction mixture was stirred at room temperature for 7 d.

To the reaction mixture, saturated NH_4Cl solution and diethyl ether were added and the phases were separated. The aqueous phase was extracted twice with diethyl ether. The combined organic phases were dried over Na_2SO_4 and filtered. The solvent was removed under reduced pressure and the residue was purified by column chromatography (SiO_2, CyH/EtOAc 0-5%). The product **3** (1.89 g, 4.81 mmol, 87%) was obtained as colorless oil. R_f (**3**) = 0.30 (pentane/EtOAc 97:3). $[\alpha]_D^{20}$ = +49.4 (c = 1.0, $CHCl_3$).

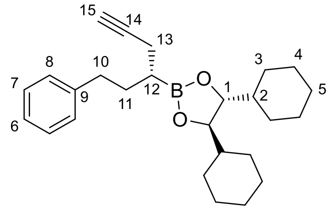

^{1}H-NMR (400 MHz, $CDCl_3$) δ: 7.29–7.24 (m, 2 H, 7-H), 7.23–7.16 (m, 3 H, 6-H, 8-H), 3.90–3.87 (m, 2 H, 1-H), 2.72–2.60 (m, 2 H, 10-H), 2.41–2.29 (m, 2 H, 13-H), 1.93 (t, *J* = 2.6 Hz, 1 H, 15-H), 1.94–1.66 (m, 12 H, 3-Ha, 3-Hb, 4-Ha, 4-Hb, 5-Ha, 11-H), 1.42–1.00 (m, 13 H, 2-H, 3-Hc, 3-Hd, 4-Hc, 4-Hd, 5-Hb, 12-H).

^{13}C-NMR (100 MHz, $CDCl_3$) δ: 142.7 (C-9), 128.4 (C-8), 128.2 (C-7), 125.6 (C-6), 84.3 (C-14), 85.5 (C-1), 68.6 (C-15), 43.0 (C-2), 35.0 (C-10), 32.4 (C-11), 28.4 (C-3a), 27.5 (C-3b), 26.5 (C-5), 26.0 (C-4a) 25.9 (C-4b), 19.8 (C-13). The signal of C-12 could not be detected.

HRMS (CI) *m/z*: $[M+H]^+$ calcd for $C_{26}H_{38}BO_2$, 393.2965; found, 393.2974.

((*R*,*E*)-4-((4*R*,5*R*)-4,5-Dicyclohexyl-1,3,2-dioxaborolan-2-yl)-1-iodo-6-phenylhex-1-en-1-yl)trimethylsilane (4a)

456 mg (982 µmol, 1.0 eq.) boronic ester **2** and 304 mg (1.18 mmol, 1.2 eq.) Schwartz reagent were suspended in 24.5 mL anhydrous THF (0.04 M) in the absence of light. The reaction mixture was stirred for 15 min at room temperature until the white precipitate was completely dissolved. Then, a 0.4 M solution of iodine (460 mg, 1.81 mmol) in 4.35 mL anhydrous THF was added until the brown colour remained. The reaction mixture was stirred for additional 30 min at room temperature.

Saturated $Na_2S_2O_3$ solution was added and the phases were separated. The aqueous phase was extracted three times with pentane. The combined organic phases were washed with brine twice, dried over Na_2SO_4 und filtered. The solvent was removed under reduced pressure. The crude product was purified by column chromatography (SiO_2, CyH/EtOAc 0-5%). The product **4a** (489 mg, 824 µmol, 84%) was obtained as a colorless oil. R_f (**4a**) = 0.48 (pentane/EtOAc 97:3). $[\alpha]_D^{20}$ = +23.0 (c = 1.0, $CHCl_3$).

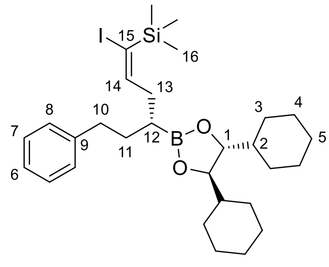

^{1}H-NMR (400 MHz, $CDCl_3$) δ: 7.29–7.26 (m, 2 H, 7-H), 7.20–7.14 (m, 4 H, 6-H, 8-H, 14-H), 3.88–3.85 (m, 2 H, 1-H), 2.68–2.53 (m, 2 H, 10-H), 2.39–2.14 (m, 2 H, 13-H), 1.81–1.59 (m, 12 H, 3-Ha, 3-Hb, 4-Ha, 4-Hb, 5-Ha, 11-H), 1.33–0.97 (m, 13 H, 2-H, 3-Hc, 3-Hd, 4-Hc, 4-Hd, 5-Hb, 12-H), 0.26 (s, 6 H, 16-Ha), 0.12 (s, 3 H, 16-Hb).

^{13}C-NMR (100 MHz, $CDCl_3$) δ: 156.2 (C-14), 142.7 (C-9), 128.4 (C-8), 128.3 (C-7), 125.7 (C-6), 107. 2 (C-15), 83.5 (C-1), 43.1 (C-2), 36.9 (C-13), 35.5 (C-10), 33.4 (C-11), 28.5 (C-3a), 27.5 (C-3b), 26.5 (C-5), 26.1 (C-4a), 25.9 (C-4b), 1.1 (C-16). The signal of C-12 could not be detected.

HRMS (CI) *m/z*: $[M]^+$ calcd for $C_{29}H_{46}BIO_2Si$, 592.2405; found, 592.2429.

((*R*,*Z*)-4-((4*R*,5*R*)-4,5-Dicyclohexyl-1,3,2-dioxaborolan-2-yl)-6-phenylhex-1-en-1-yl)trimethylsilane (4b)

623 mg (1.34 mmol, 1.0 eq.) boronic ester **2** and 380 mg (1,48 mmol, 1.1 eq.) Schwartz reagent were suspended in 33.5 mL anhydrous THF (0.04 M) in the absence of light. The reaction mixture was stirred for 15 min at room temperature until the white precipitate was completely dissolved.

Saturated NH_4Cl solution was added and the phases were separated. The aqueous phase was extracted three times with pentane. The combined organic phases were dried over Na_2SO_4 and filtered. The solvent was removed under reduced pressure. The crude product was purified by column chromatography (SiO_2, CyH/EtOAc 0-5%). The product **4b** (558 mg, 1.20 mmol, 89%) was obtained as a colorless oil. R_f (**4b**) = 0.33 (pentane/EtOAc 97:3). $[\alpha]_D^{20}$ = +36.5 (c = 1.0, $CHCl_3$).

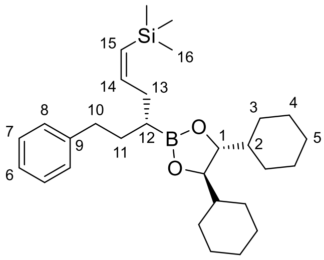

^{1}H-NMR (400 MHz, $CDCl_3$) δ: 7.29–7.25 (m, 2 H, 7-H), 7.19–7.15 (m, 3 H, 6-H, 8-H), 6.34–6.27 (m, 1 H, 14-H), 5.47 (d, *J* = 14.1 Hz, 1 H, 15-H), 3.87–3.84 (m, 2 H, 1-H), 2.70–2.56 (m, 2 H, 10-H), 2.36–2.20 (m, 2 H, 13-H), 1.83 – 1.56 (m, 12 H, 3-Ha, 3-Hb, 4-Ha, 4-Hb, 5-Ha, 11-H), 1.34 – 0.97 (m, 13 H, 2-H, 3-Hc, 3-Hd, 4-Hc, 4-Hd, 5-Hb, 12-H), 0.11 (s, 9 H, 16-H).

^{13}C-NMR (100 MHz, $CDCl_3$) δ: 148.8 (C-14), 143.0 (C-9), 129.2 (C-15), 128.4 (C-8), 128.2 (C-7), 125.5 (C-6), 83.4 (C-1), 43.1 (C-2), 35.7 (C-10), 35.1 (C-13), 33.3 (C-11), 28.5 (C-3a), 27.5 (C-3b), 26.5 (C-5), 26.0 (C-4a), 25.9 (C-4b), 0.2 (C-16). The signal of C-12 could not be detected.

HRMS (CI) *m/z*: $[M+H]^+$ calcd for $C_{29}H_{48}BO_2Si$, 467.3517; found, 467.3549.

(4*R*,5*R*)-2-((*R*,*E*)-6-Chloro-1-phenylhex-5-en-3-yl)-4,5-dicyclohexyl-1,3,2-dioxaborolane (5a)

To a solution of 139 mg (354 µmol, 1.0 eq.) boronic ester **3** in 0.82 mL anhydrous THF was added 183 mg (709 µmol, 2.0 eq.) Schwartz reagent at room temperature in the absence of light. The reaction mixture was stirred for 25 min at room temperature until it became clear. Then, a solution of 95.0 mg (709 µmol, 2.0 eq.) *N*-chlorosuccinimide in 1.63 mL anhydrous THF was added at room temperature. The reaction mixture was stirred for 16 h. Water was added to the reaction mixture and the phases were separated. The aqueous phase was extracted three times with ethyl acetate. The combined organic phases were washed with brine, dried over Na_2SO_4 and filtered. The solvent was removed under reduced pressure and the residue was purified by column chromatography (SiO_2, CyH/EtOAc 0-4%). The product **5a** (101 mg, 236 µmol, 66%) was obtained as a colorless oil. R_f (**5a**) = 0.30 (pentane/EtOAc 97:3). $[\alpha]_D^{20}$ = +33.1 (c = 1.0, $CHCl_3$).

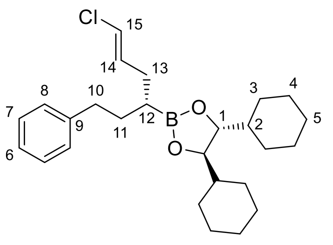

^{1}H-NMR (400 MHz, $CDCl_3$) δ: 7.29–7.26 (m, 2 H, 7-H), 7.21–7.16 (m, 3 H, 6-H, 8-H), 5.97–5.88 (m, 2 H, 14-H, 15-H), 3.87–3.85 (m, 2 H, 1-H), 2.68–2.55 (m, 2 H, 10-H), 2.26–2.14 (m, 2 H, 13-H), 1.79–1.56 (m, 12 H, 3-Ha, 3-Hb, 4-Ha, 4-Hb, 5-Ha, 11-H), 1.35–0.89 (m, 13 H, 2-H, 3-Hc, 3-Hd, 4-Hc, 4-Hd, 5-Hb, 12-H).

^{13}C-NMR (100 MHz, $CDCl_3$) δ: 142.7 (C-9), 133.5 (C-14), 128.4 (C-8), 128.3 (C-7), 125.7 (C-6), 117.2 (C-15), 83.5 (C-1), 43.1 (C-2), 35.4 (C-10), 33.1 (C-11), 32.5 (C-13), 28.5 (C-3a), 27.5 (C-3b), 26.4 (C-5), 26.0 (C-4a), 25.9 (C-4b). The signal of C-12 could not be detected.

HRMS (ESI) *m/z*: $[M+H]^+$ calcd for $C_{26}H_{39}BClO_2$, 429.2726; found, 429.2706.

(4*R*,5*R*)-2-((*R*,*E*)-6-Bromo-1-phenylhex-5-en-3-yl)-4,5-dicyclohexyl-1,3,2-dioxaborolane (5b)

To a solution of 241 mg (622 µmol, 1.0 eq.) boronic ester **6** in 0.72 mL anhydrous THF was added 241 mg (933 µmol, 1.5 eq.) Schwartz reagent at room temperature in the absence of light. The reaction mixture was stirred for 30 min until it became clear. Then, 177 mg (995 µmol, 1.6 eq.) *N*-bromosuccinimide were added and the reaction mixture was stirred for 1 h at room temperature. Water and diethyl ether were added and the phases were separated. The aqueous phase was extracted twice with diethyl ether. The combined organic phases were washed with brine, dried over Na_2SO_4 and filtered. The solvent was removed under reduced pressure and the residue was purified by column chromatography (SiO_2, CyH/EtOAc 0-5%). The product **5b** (269 mg, 569 µmol, 91%) was obtained as a colorless oil. R_f (**5b**) = 0.43 (pentane/EtOAc 97:3). $[\alpha]_D^{20}$ = +46.4 (c = 1.0, $CHCl_3$).

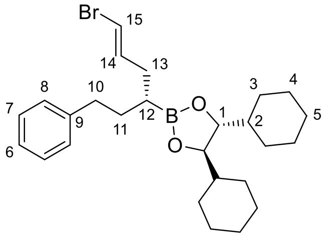

^{1}H-NMR (400 MHz, $CDCl_3$) δ: 7.30–7.25 (m, 2 H, 7-H), 7.19–7.16 (m, 3 H, 6-H, 8-H), 6.18 (dd, J = 13.5 Hz, 7.4 Hz, 1 H, 14-H), 6.03 (d, J = 13.5 Hz, 15-H), 3.88–3.85 (m, 2 H, 1-H), 2.69–2.56 (m, 2 H, 10-H), 2.28–2.13 (m, 2 H, 13-H), 1.82–1.60 (m, 12 H, 3-Ha, 3-Hb, 4-Ha, 4-Hb, 5-Ha, 11-H), 1.35–0.84 (m, 13 H, 2-H, 3-Hc, 3-Hd, 4-Hc, 4-Hd, 5-Hb, 12-H).

^{13}C-NMR (100 MHz, $CDCl_3$) δ: 142.7 (C-9), 137.7 (C-14), 128.4 (C-8), 128.3 (C-7), 125.7 (C-6), 104.7 (C-15), 83.5 (C-1), 43.1 (C-2), 35.4 (C-10), 34.6 (C-13), 33.1 (C-11), 28.5 (C-3a), 27.6 (C-3b), 26.5 (C-5), 26.0 (C-4a), 25.9 (C-4b). The signal of C-12 could not be detected.

HRMS (CI) *m/z*: $[M]^+$ calcd for $C_{26}H_{38}BBrO_2$, 472.2148; found, 472.2157.

(4*R*,5*R*)-4,5-Dicyclohexyl-2-((*R*,*E*)-6-iodo-1-phenylhex-5-en-3-yl)-1,3,2-dioxaborolane (5c)

1.21 g (3.09 mmol, 1.0 eq.) boronic ester **3** and 958 mg (3.71 mmol, 1.2 eq.) Schwartz reagent were suspended in 9.29 mL anhydrous DCM (0.33 M) in the absence of light. The reaction mixture was stirred for 5 min at room temperature until the white precipitate was completely dissolved. Then, 982 mg (3.87 mmol, 1.25 eq.) iodine was added. The reaction mixture was stirred for 30 min at room temperature.

Saturated $Na_2S_2O_3$ solution was added and the phases were separated. The aqueous phase was extracted three times with pentane. The combined organic phases were washed with brine, dried over Na_2SO_4 und filtered. The solvent was removed under reduced pressure. The crude product was purified by column chromatography (SiO_2, CyH/EtOAc 0-5%). The product **5c** (1.54 g, 2.97 mmol, 96%) was obtained as a colorless oil. R_f (**5c**) = 0.29 (pentane/EtOAc 97:3). $[\alpha]_D^{20}$ = +18.3 (c = 1.0, $CHCl_3$).

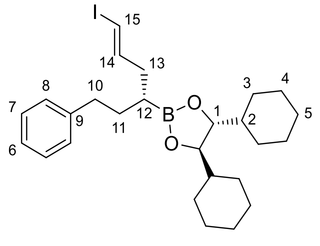

^{1}H-NMR (400 MHz, $CDCl_3$) δ: 7.29–7.26 (m, 2 H, 7-H), 7.19–7.16 (m, 3 H, 6-H, 8-H), 6.52 (dt, *J* = 14.4 Hz, 7.2 Hz, 1 H, 14-H), 6.00 (dt, *J* = 14.3 Hz, 1.4 Hz, 1 H, 15-H), 3.87–3.84 (m, 2 H, 1-H), 2.68–2.55 (m, 2 H, 10-H), 2.29–2.14 (m, 2 H, 13-H), 1.81–1.59 (m, 12 H, 3-Ha, 3-Hb, 4-Ha, 4-Hb, 5-Ha, 11-H), 1.36–0.93 (m, 13 H, 2-H, 3-Hc, 3-Hd, 4-Hc, 4-Hd, 5-Hb, 12-H).

^{13}C-NMR (100 MHz, $CDCl_3$) δ: 146.1 (C-14), 142.6 (C-9), 128.4 (C-8), 128.2 (C-7), 125.7 (C-6), 83.5 (C-1), 75.1 (C-15), 43.1 (C-2), 37.7 (C-13), 35.4 (C-10), 33.0 (C-11), 28. 5 (C-3a), 27.6 (C-3b), 26.5 (C-5), 26.0 (C-4a), 25.9 (C-4b). The signal of C-12 could not be detected.

HRMS (CI) *m/z*: $[M]^+$ calcd for $C_{26}H_{38}BIO_2$, 520.2010; found, 520.2020.

(4*R*,5*R*)-4,5-Dicyclohexyl-2-((*R*,*E*)-1,6-diphenylhex-5-en-3-yl)-1,3,2-dioxaborolane (6a)

According to GP-2, 226 mg (1.66 mmol) $ZnCl_2$, 0.55 mL (1.66 mmol, 3.0 M) phenylmagnesium bromide and 2.90 mL anhydrous THF were used for the preparation of the arylzinc reagent. 86.3 mg (166 µmol) boronic ester **5c**, 19.2 mg (17.0 µmol) $Pd(PPh_3)_4$, 3.32 mL anhydrous THF and 1.73 mL of the arylzinc reagent solution were used for the reaction mixture. The reaction was worked up after 2 h. After column chromatography (SiO_2, CyH/EtOAc 0-3%), the product **6a** (99.8 mg, 212 µmol, 92%) was obtained as a yellow oil. R_f (**6a**) = 0.31 (pentane/EtOAc 97:3). $[\alpha]_D^{20}$ = +20.0 (c = 1.0, $CHCl_3$).

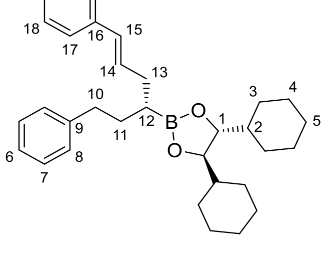

^{1}H-NMR (400 MHz, $CDCl_3$) δ: 7.32–7.25 (m, 6 H, 7-H, 17-H, 18-H), 7.21–7.15 (m, 4 H, 6-H, 8-H, 19-H), 6.38 (d, *J* = 15.9 Hz, 1 H, 15-H), 6.24 (dt, *J* = 15.8 Hz, 7.0 Hz, 1 H, 14-H), 3.86–3.84 (m, 2 H, 1-H), 2.70–2.59 (m, 2 H, 10-H), 2.41–2.35 (m, 2 H, 13-H), 1.84–1.58 (m, 12 H, 3-Ha, 3-Hb, 4-Ha, 4-Hb, 5-Ha, 11-H), 1.33–1.26 (m, 2 H, 2-H), 1.21–0.89 (m, 11 H, 3-Hc, 3-Hd, 4-Hc, 4-Hd, 5-Hb, 12-H).

^{13}C-NMR (100 MHz, $CDCl_3$) δ: 143.0 (C-9), 130.4 (C-16), 130.3 (C-15), 128.4 (C-8), 128.4 (C-7, C-17), 128.2 (C-18), 126.7 (C-14), 125.9 (C-6), 125.6 (C-19), 83.4 (C-1), 43.1 (C-2), 35.6 (C-10), 34.8 (C-13), 33.4 (C-11), 28.5 (C-3a), 27.6 (C-3b), 26.4 (C-5), 26.0 (C-4a), 25.8 (C-4b).The signal of C-12 could not be detected.

HRMS (CI) *m/z*: $[M]^+$ calcd for $C_{32}H_{43}BO_2$, 470.3356; found, 470.3355.

(4*R*,5*R*)-4,5-Dicyclohexyl-2-((*R*,*E*)-1-phenyl-6-(*p*-tolyl)hex-5-en-3-yl)-1,3,2-dioxaborolane (6b)

According to GP-2, 288 mg (2.11 mmol) $ZnCl_2$, 2.11 mL (2.11 mmol, 1.0 M) *p*-tolylmagnesium bromide and 3.69 mL anhydrous THF were used for the preparation of the arylzinc reagent. 110 mg (211 µmol) boronic ester **5c**, 24.4 mg (21.0 µmol) $Pd(PPh_3)_4$, 4.22 mL anhydrous THF and 2.90 mL of the arylzinc reagent solution were used for the reaction mixture. The reaction mixture was worked up after 2 h. After column chromatography (SiO_2, CyH/EtOAc 0-5%), the product **6b** (92.3 mg, 190 µmol, 90%) was obtained as a colorless to yellow oil.

1 mmol scale

According to GP-2, 1.71 g (12.5 mmol) $ZnCl_2$, 12.5 mL (12.5 mmol, 1.0 M) *p*-tolylmagnesium bromide and 21.9 mL anhydrous THF were used for the preparation of the arylzinc reagent. 651 mg (1.25 mmol) boronic ester **5c**, 145 mg (125 µmol) $Pd(PPh_3)_4$, 25.0 mL anhydrous THF and 17.2 mL of the arylzinc reagent solution were used for the reaction mixture. The reaction mixture was worked up after 2 h. After column chromatography (SiO_2, CyH/EtOAc 0-5%), the product **6b** (568 mg, 1.17 mmol, 94%) was obtained as a colorless to yellow oil.

R_f (**6b**) = 0.43 (pentane/EtOAc 97:3). $[\alpha]_D^{20}$ = +22.0 (c = 1.0, $CHCl_3$).

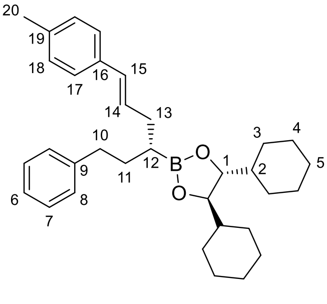

^{1}H-NMR (400 MHz, $CDCl_3$) δ: 7.28–7.25 (m, 2 H, 7-H), 7.22–7.15 (m, 5 H, 6-H, 8-H, 17-H), 7.09–7.07 (m, 2 H, 18-H), 6.35 (d, *J* = 15.8 Hz, 1 H, 15-H), 6.18 (dt, *J* = 15.8 Hz, 7.1 Hz, 1 H, 14-H), 3.86–3.84 (m, 2 H, 1-H), 2.71–2.59 (m, 2 H, 10-H), 2.41–2.33 (m, 2 H, 13-H), 2.31 (s, 3 H, 20-H), 1.83–1.58 (m, 12 H, 3-Ha, 3-Hb, 4-Ha, 4-Hb, 5-Ha, 11-H), 1.33–0.95 (m, 13 H, 2-H, 3-Hc, 3-Hd, 4-Hc, 4-Hd, 5-Hb, 12-H).

^{13}C-NMR (100 MHz, $CDCl_3$) δ: 143.0 (C-9), 136.4 (C-19), 135.1 (C-17), 130.2 (C-15), 129.4 (C-14), 129.1 (C-18), 128.4 (C-8), 128.2 (C-7), 125.8 (C-6), 125.6 (C-16), 83.4 (C-1), 43.1 (C-

2), 35.6 (C-10), 34.8 (C-13), 33.3 (C-11), 28.5 (C-3a), 27.6 (C-3b), 26.4 (C-5), 26.0 (C-4a), 25.8 (C-4b), 21.1 (C-20). The signal of C-12 could not be detected.

HRMS (CI) *m/z*: $[M]^+$ calcd for $C_{33}H_{45}BO_2$, 484.3515; found, 484.3481.

(4*R*,5*R*)-4,5-dicyclohexyl-2-((*R*,*E*)-1-phenyl-6-(*o*-tolyl)hex-5-en-3-yl)-1,3,2-dioxaborolane (6c)

According to GP-2, 278 mg (2.04 mmol) $ZnCl_2$, 1.02 mL (2.04 mmol, 2.0 M) *o*-tolylmagnesium bromide and 3.57 mL anhydrous THF were used for the preparation of the arylzinc reagent. Addition of the Grignard reagent was performed at −78 °C. 106 mg (204 µmol) boronic ester **5c**, 23.6 mg (20.0 µmol) $Pd(PPh_3)_4$, 4.08 mL anhydrous THF and 2.30 mL of the arylzinc reagent solution were used for the reaction mixture. The reaction mixture was worked up after 2 h. After column chromatography (SiO_2, CyH/EtOAc 0-3%), the product **6c** (81.8 mg, 169 µmol, 83%) was obtained as a colorless oil. R_f (**6c**) = 0.26 (pentane/EtOAc 97:3). $[\alpha]_D^{20}$ = +24.9 (c = 1.0, $CHCl_3$).

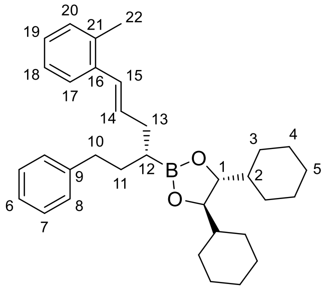

^{1}H-NMR (400 MHz, $CDCl_3$) δ: 7.40–7.38 (m, 1 H, 17-H), 7.29–7.25 (m, 2 H, 7-H), 7.19–7.11 (m, 6 H, 6-H, 8-H, 18-H, 19-H, 20-H), 6.58 (d, *J* = 15.7 Hz, 1 H, 15-H), 6.11 (dt, *J* = 15.7 Hz, 7.3 Hz, 1 H, 14-H), 3.87–3.84 (m, 2 H, 1-H), 2.72–2.60 (m, 2 H, 10-H), 2.46–2.35 (m, 2 H, 13-H), 2.32 (s, 3 H, 22-H), 1.84–1.58 (m, 12 H, 3-Ha, 3-Hb, 4-Ha, 4-Hb, 5-Ha, 11-H), 1.31–0.93 (m, 13 H, 2-H, 3-Hc, 3-Hd, 4-Hc, 4-Hd, 5-Hb, 12-H).

^{13}C-NMR (100 MHz, $CDCl_3$) δ: 143.0 (C-9), 136.9 (C-16), 134.8 (C-21), 131.8 (C-19), 130.1 (C-20), 128.4 (C-8), 128.3 (C-7), 128.2 (C-15), 126.7 (C-18), 125.9 (C-14), 125.6 (C-6), 125.4 (C-17), 83.5 (C-1), 43.1 (C-2), 35.6 (C-10), 35.1 (C-13), 33.2 (C-11), 28.5 (C-3a), 27.6 (C-3b), 26.4 (C-5), 26.0 (C-4a), 25.8 (C-4b), 19.9 (C-22). The signal of C-12 could not be detected.

HRMS (CI) *m/z*: $[M]^+$ calcd for $C_{33}H_{45}BO_2$, 484.3513; found, 484.3520.

(4*R*,5*R*)-4,5-Dicyclohexyl-2-((*R*,*E*)-6-(4-methoxyphenyl)-1-phenylhex-5-en-3-yl)-1,3,2-dioxaborolane (6d)

According to GP-2, 249 mg (1.83 mmol) $ZnCl_2$, 1.92 mL (1.83 mmol, 0.95 M) (4-methoxyphenyl)magnesium bromide and 3.20 mL anhydrous THF were used for the preparation of the arylzinc reagent. 95.0 mg (183 µmol) boronic ester **5c**, 21.1 mg (18 µmol) $Pd(PPh_3)_4$, 3.65 mL anhydrous THF and 2.56 mL of the arylzinc reagent solution were used for the reaction mixture. The reaction was worked up after 2 h. After column chromatography (SiO_2, CyH/EtOAc 0-3%), the product **6d** (85.8 mg, 171 µmol, 94%) was obtained as a colorless oil. R_f (**6d**) = 0.32 (pentane/EtOAc 97:3). $[\alpha]_D^{20}$ = +13.9 (c = 1.0, $CHCl_3$).

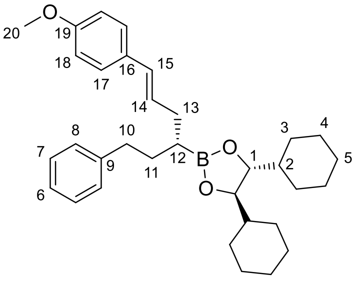

^{1}H-NMR (400 MHz, $CDCl_3$) δ: 7.29–7.24 (m, 4 H, 7-H, 17-H), 7.19–7.15 (m, 3 H, 6-H, 8-H), 6.83–6.81 (m, 2 H, 18-H), 6.33 (d, *J* = 15.8 Hz, 1 H, 15-H), 6.10 (dt, *J* = 15.8 Hz, 7.1 Hz, 1 H, 14-H), 3.87–3.84 (m, 2 H, 1-H), 3.80 (s, 3 H, 20-H), 2.71–2.59 (m, 2 H, 10-H), 2.37–2.33 (m, 2 H, 13-H), 1.81–1.57 (m, 12 H, 3-Ha, 3-Hb, 4-Ha, 4-Hb, 5-Ha, 11-H), 1.33–1.26 (m, 2 H, 2-H), 1.20–0.93 (m, 11 H, 3-Hc, 3-Hd, 4-Hc, 4-Hd, 5-Hb, 12-H).

^{13}C-NMR (100 MHz, $CDCl_3$) δ: 158.6 (C-19), 143.0 (C-9), 130.7 (C-16), 129.7 (C-15), 128.4 (C-8), 128.2 (C-7), 127.7 (C-14), 127.0 (C-17), 125.6 (C-6), 113.8 (C-18), 83.4 (C-1), 55.3 (C-20), 43.1 (C-2), 35.6 (C-10), 34.8 (C-13), 33.4 (C-11), 28.5 (C-3a), 27.6 (C-3b), 26.4 (C-5), 26.0 (C-4a), 25.8 (C-4b). The signal of C-12 could not be detected.

HRMS (CI) *m/z*: $[M+H]^+$ calcd for $C_{33}H_{46}BO_3$, 501.3540; found, 501.3544.

(4*R*,5*R*)-2-((*R*,*E*)-6-(Benzo[d][1,3]dioxol-5-yl)-1-phenylhex-5-en-3-yl)-4,5-dicyclohexyl-1,3,2-dioxaborolane (6e/7e)

According to GP-2, 287 mg (2.11 mmol) $ZnCl_2$, 2.29 mL (2.11 mmol, 0.92 M) benzo[d][1,3]dioxol-5-ylmagnesium bromide and 3.69 mL anhydrous THF were used for the preparation of the arylzinc reagent. 110 mg (211 µmol) boronic ester **5c**, 24.3 mg (19.0 µmol) $Pd(PPh_3)_4$, 4.21 mL anhydrous THF and 2.99 mL of the arylzinc reagent solution were used for the reaction mixture. The reaction mixture was worked up after 1 h. After column chromatography (SiO_2, CyH/EtOAc 0-3%), the product **6e** (98.9 mg, 192 µmol, 91%) was obtained as a colorless oil. R_f (**6e**) = 0.31 (pentane/EtOAc 97:3). $[\alpha]_D^{20}$ = +16.3 (c = 1.0, $CHCl_3$).

For the Grignard reagent solution, 0.84 mL (1.41 g, 7.00 mmol, 1.0 eq.) 5-bromobenzo[d][1,3]dioxol in 7.00 mL anhydrous THF was added dropwise to 255 mg (10.5 mmol, 1.5 eq.) magnesium with a catalytic amount of iodine at room temperature. After 5 h stirring at room temperature, the concentration was determined by iodometric titration.

80.2 mg (156 µmol) boronic ester **6e** was subjected to oxidation according to GP-3 using 312 µL THF, 71.1 µL (26.5 mg, 779 µmol, 33%wt) aqueous H_2O_2 solution and a solution of 31.2 mg (779 µmol) NaOH in 312 µL water. The reaction was worked up after 1 h. To improve chromatographic separation, 11.2 mg (187 µmol) methylboronic acid and 0.78 mL diethyl ether were used. After column chromatography (SiO_2, CyH/EtOAc 0-20%; C18-SiO_2, H_2O → MeCN), the product **7e** (41.2 mg, 139 µmol, 89%) was obtained as a colorless oil. R_f (**7e**) = 0.27 (pentane/EtOAc 8:2). $[\alpha]_D^{20}$ = +6.9 (c = 1.0, $CHCl_3$).

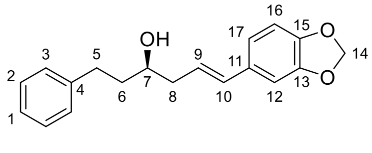

^{1}H-NMR (400 MHz, $CDCl_3$) δ: 7.31–7.27 (m, 2 H, 2-H), 7.23–7.18 (m, 3 H, 1-H, 3-H), 6.91–6.90 (m, 1 H, 12-H), 6.79–6.73 (m, 2 H, 16-H, 17-H), 6.39 (d, *J* = 15.9 Hz, 1 H, 10-H), 6.04 (dt, *J* = 15.8 Hz, 7.6 Hz, 1 H, 9-H), 5.94 (s, 2 H, 14-H), 3.76–3.70 (m, 1 H, 7-H), 2.87–2.80 (m,

1 H, 5-Ha), 2.75–2.67 (m, 1 H, 5-Hb), 2.47–2.40 (m, 1 H, 8-Ha), 2.35–2.27 (m, 1 H, 8-Hb), 1.86–1.80 (m, 2 H, 6-H), 1.66 (br s, 1 H, OH).

^{13}C-NMR (100 MHz, $CDCl_3$) δ: 148.0 (C-13), 147.0 (C-15), 142.0 (C-4), 132.9 (C-10), 131.7 (C-11), 128.4 (C-3), 128.4 (C-2), 125.8 (C-1), 124.2 (C-9), 120.6 (C-17), 108.2 (C-16), 105.5 (C-12), 101.0 (C-14), 70.4 (C-7), 41.2 (C-8), 38.5 (C-6), 32.1 (C-5).

HRMS (CI) *m/z*: $[M]^+$ calcd for $C_{19}H_{20}O_3$, 296.1412; found, 296.1417.

(4*R*,5*R*)-4,5-Dicyclohexyl-2-((*R*,*E*)-6-(2-methoxyphenyl)-1-phenylhex-5-en-3-yl)-1,3,2-dioxaborolane (6f)

According to GP-2, 262 mg (1.93 mmol) $ZnCl_2$, 2-methoxyphenyllithium and 1.93 mL anhydrous THF were used for the preparation of the arylzinc reagent. 100 mg (193 µmol) boronic ester **5c**, 22.3 mg (19.0 µmol) $Pd(PPh_3)_4$, 3.85 mL anhydrous THF and 3.87 mL of the arylzinc reagent solution were used for the reaction mixture. The reaction mixture was worked up after 2 h. After column chromatography (SiO_2, CyH/EtOAc 0-3%), the product **6f** (73.4 mg, 147 µmol, 76%) was obtained as a colorless oil. R_f (**6f**) = 0.30 (pentane/EtOAc 97:3). $[\alpha]_D^{20}$ = +28.6 (c = 1.0, $CHCl_3$).

For the 2-methoxyphenyllithium solution, 0.77 mL (1.93 mmol, 2.5 M) *n*BuLi were added according to literature procedure at −78 °C to a solution of 210 µL (208 mg, 1.93 mmol) anisole in 4.82 mL anhydrous THF. The reaction mixture was stirred at 0 °C for 30 min until addition to the $ZnCl_2$ solution.

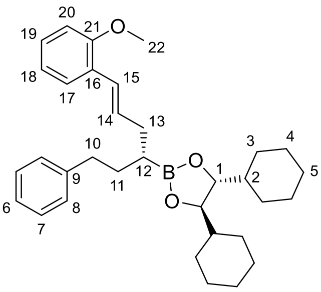

^{1}H-NMR (400 MHz, $CDCl_3$) δ: 7.40 (dd, *J* = 7.6 Hz, 1.5 Hz, 1 H, 17-H), 7.28 – 7.25 (m, 2 H, 7-H), 7.19–7.14 (m, 4 H, 6-H, 8-H, 19-H), 6.90–6.83 (m, 2 H, 18-H, 20-H), 6.73 (d, *J* = 16.0 Hz, 1 H, 15-H), 6.22 (dt, *J* = 15.9 Hz, 7.2 Hz, 1 H, 14-H), 3.86–3.85 (m, 2 H, 1-H), 3.83 (s, 3 H, 22-H), 2.68–2.61 (m, 2 H, 10-H), 2.42–2.38 (m, 2 H, 13-H), 1.83 – 1.59 (m, 12 H, 3-Ha, 3-Hb, 4-Ha, 4-Hb, 5-Ha, 11-H), 1.33 – 0.87 (m, 13 H, 2-H, 3-Hc, 3-Hd, 4-Hc, 4-Hd, 5-Hb, 12-H).

^{13}C-NMR (100 MHz, $CDCl_3$) δ: 156.2 (C-21), 143.1 (C-9), 131.1 (C-14), 128.4 (C-8), 128.2 (C-7), 127.7 (C-16), 127.0 (C-17), 126.3 (C-19), 125.5 (C-6), 124.9 (C-15), 120.6 (C-18), 110.7 (C-20), 83.4 (C-1), 55.4 (C-22), 43.1 (C-2), 35.6 (C-10), 35.2 (C-13), 33.3 (C-11), 28.5 (C-3a), 27.6 (C-3b), 26.4 (C-5), 26.0 (C-4a), 25.8 (C-4b). The signal of C-12 could not be detected.

HRMS (CI) *m/z*: $[M+H]^+$ calcd for $C_{33}H_{46}BO_3$, 501.3540; found, 501.3533.

(4R,5R)-4,5-Dicyclohexyl-2-((R,E)-6-(4-fluorophenyl)-1-phenylhex-5-en-3-yl)-1,3,2-dioxaborolane (6g)

According to GP-2, 298 mg (2.19 mmol) $ZnCl_2$, 2.19 mL (2.18 mmol, 1.0 M) (4-fluorophenyl)magnesium bromide and 3.83 mL anhydrous THF were used for the preparation of the arylzinc reagent. 114 mg (219 µmol) boronic ester **5c**, 25.3 mg (22.0 µmol) $Pd(PPh_3)_4$, 4.37 mL anhydrous THF and 3.01 mL of the arylzinc reagent solution were used for the reaction

mixture. The reaction mixture was worked up after 2 h. After column chromatography (SiO_2, CyH/EtOAc 0-3%), the product **6g** (89.1 mg, 182 µmol, 83%) was obtained as a yellow oil. R_f (**6g**) = 0.33 (pentane/EtOAc 97:3). $[\alpha]_D^{20}$ = +26.1 (c = 1.0, $CHCl_3$).

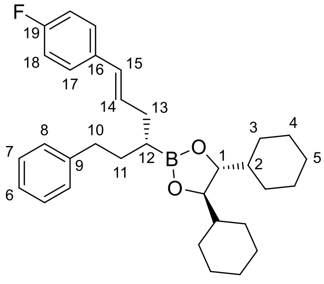

¹H-NMR (400 MHz, $CDCl_3$) δ: 7.28–7.25 (m, 4 H, 7-H, 17-H), 7.19–7.15 (m, 3 H, 6-H, 8-H), 6.98–6.94 (m, 2 H, 18-H), 6.34 (d, *J* = 15.9 Hz, 1 H, 15-H), 6.15 (dt, *J* = 15.8 Hz, 7.1 Hz, 1 H, 14-H), 3.86–3.83 (m, 2 H, 1-H), 2.70–2.59 (m, 2 H, 10-H), 2.37–2.33 (m, 2 H, 13-H), 1.79–1.58 (m, 12 H, 3-Ha, 3-Hb, 4-Ha, 4-Hb, 5-Ha, 11-H), 1.30–1.25 (m, 2 H, 2-H), 1.19–0.94 (m, 11 H, 3-Hc, 3-Hd, 4-Hc, 4-Hd, 5-Hb, 12-H).

¹³C-NMR (100 MHz, $CDCl_3$) δ: 161.8 (d, *J* = 245.0 Hz, C-19), 142.9 (C-9), 133.9 (d, *J* = 3.7 Hz, C-16), 130.1 (C-14), 129.1 (C-15), 128.4 (C-8), 128.3 (C-7), 127.3 (d, *J* = 8.1 Hz, C-17), 125.6 (C-6), 115.2 (d, *J* = 21.3 Hz, C-18), 83.5 (C-1), 43.1 (C-2), 35.6 (C-10), 34.8 (C-13), 33.4 (C-11), 28.5 (C-3a), 27.6 (C-3b), 26.4 (C-5), 26.0 (C-4a), 25.8 (C-4b). The signal of C-12 could not be detected.

¹⁹F-NMR (377 MHz, $CDCl_3$, δ in ppm): −115.9.

HRMS (CI) *m/z*: $[M]^+$ calcd for $C_{32}H_{42}BFO_2$, 488.3262; found, 488.3267.

(4*R*,5*R*)-4,5-Dicyclohexyl-2-((*R*,*E*)-1-phenyl-6-(4-(trifluoromethyl)phenyl)hex-5-en-3-yl)-1,3,2-dioxaborolane (6h)

According to GP-2, 265 mg (1.95 mmol) $ZnCl_2$, 3.74 mL (1.95 mmol, 0.52 M) (4-(trifluormethyl)phenyl)magnesium bromide and 3.41 mL anhydrous THF were used for the preparation of the arylzinc reagent. 101 mg (195 µmol) boronic ester **5c**, 22.5 mg (19 µmol) $Pd(PPh_3)_4$, 3.89 mL anhydrous THF and 3.58 mL of the arylzinc reagent solution were used for the reaction mixture. The reaction was worked up after 2 h. After column chromatography (SiO_2, CyH/EtOAc 0-3%), the product **6h** (94.4 mg, 175 µmol, 90%) was obtained as a colorless oil. R_f (**6h**) = 0.25 (pentane/EtOAc 97:3). $[\alpha]_D^{20}$ = +20.6 (c = 1.0, $CHCl_3$).

For the Grignard reagent solution, 66 µL (145 mg, 0.77 mmol, 0.11 eq.) 1,2-dibromoethane was added to 187 mg (7.70 mmol, 1.1 eq.) magnesium in 1.06 mL anhydrous Et_2O according to literature procedure.[9] The solution was gently heated with a heat gun. After cooling to room temperature, a portion of a solution of 0.98 mL (1.58 g, 7.0 mmol, 1.0 eq.) 1-bromo-4-(trifluoromethyl)benzene in 10.6 mL anhydrous Et_2O was added and the reaction mixture was refluxed before the rest of the solution was added dropwise. After refluxing for 1 h, the reaction mixture was cooled to room temperature and the concentration was determined by iodometric titration.

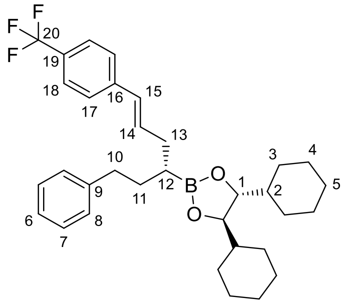

^{1}H-NMR (400 MHz, $CDCl_3$) δ: 7.53–7.51 (m, 2 H, 18-H), 7.41–7.38 (m, 2 H, 17-H), 7.29–7.25 (m, 2 H, 7-H), 7.19–7.16 (m, 3 H, 6-H, 8-H), 6.43–6.31 (m, 2 H, 14-H, 15-H), 3.86–3.84 (m, 2 H, 1-H), 2.72–2.59 (m, 2 H, 10-H), 2.41–2.37 (m, 2 H, 13-H), 1.83–1.57 (m, 12 H, 3-Ha, 3-Hb, 4-Ha, 4-Hb, 5-Ha, 11-H), 1.33–1.25 (m, 2 H, 2-H), 1.19–0.92 (m, 11 H, 3-Hc, 3-Hd, 4-Hc, 4-Hd, 5-Hb, 12-H).

^{13}C-NMR (100 MHz, $CDCl_3$) δ: 142.8 (C-9), 141.3 (C-19), 133.4 (C-14), 129.1 (C-15), 128.4 (C-8), 128.3 (C-7), 126.0 (C-17), 125.6 (C-6), 125.4 (C-16), 125.3 (C-18), 83.5 (C-1), 43.1 (C-2), 35.6 (C-10), 34.9 (C-13), 33.4 (C-11), 28.5 (C-3a), 27.6 (C-3b), 26.4 (C-5), 25.9 (C-4a), 25.8 (C-4b). The signals of C-12 and C-20 could not be detected.

^{19}F-NMR (377 MHz, $CDCl_3$, δ in ppm): −65.2.

HRMS (CI) *m/z*: $[M]^+$ calcd for $C_{33}H_{42}BF_3O_2$, 538.3230; found, 538.3235.

(4*R*,5*R*)-4,5-Dicyclohexyl-2-((*R*,*E*)-6-cyclopentyl-1-phenylhex-5-en-3-yl)-1,3,2-dioxaborolane (6i/7i)

According to GP-2, 270 mg (1.98 mmol) $ZnCl_2$, 0.99 mL (1.98 mmol, 2.0 M) cyclopentylmagnesium bromide and 3.47 mL anhydrous THF were used for the preparation of the arylzinc reagent. 103 mg (198 µmol) boronic ester **5c**, 22.9 mg (20.0 µmol) $Pd(PPh_3)_4$, 3.96 mL anhydrous THF and 2.23 mL of the arylzinc reagent solution were used for the reaction mixture. The reaction mixture was worked up after 4 h. After column chromatography (SiO_2, CyH/EtOAc 0-3%), the product **6i** (63.5 mg, 137 µmol, 69%) was obtained as a colorless oil. R_f (**6i**) = 0.29 (pentane/EtOAc 97:3). $[\alpha]_D^{20}$ = +22.0 (c = 1.0, $CHCl_3$).

56.7 mg (123 µmol) boronic ester **6i** was subjected to oxidation according to GP-3 using 245 µL THF, 55.9 µL (20.9 mg, 613 µmol, 33%wt) aqueous H_2O_2 solution and a solution of 24.5 mg (613 µmol) NaOH in 245 µL water. The reaction was worked up after 1 h. To improve chromatographic separation, 8.81 mg (147 µmol) methylboronic acid and 0.62 mL diethyl ether were used. After column chromatography (SiO_2, CyH/EtOAc 0-20%; C18-SiO_2, H_2O → MeCN), the product **7i** (19.2 mg, 78.7 µmol, 64%) was obtained as a colorless oil. R_f (**7i**) = 0.43 (pentane/EtOAc 8:2). $[\alpha]_D^{20}$ = +10.4 (c = 1.0, $CHCl_3$).

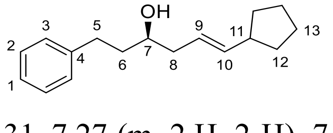

^{1}H-NMR (400 MHz, $CDCl_3$) δ: 7.31–7.27 (m, 2 H, 2-H), 7.22–7.17 (m, 3 H, 1-H, 3-H), 5.54 (dd, *J* = 15.3 Hz, 7.5 Hz, 1 H, 10-H), 5.38 (dt, *J* = 15.3 Hz, 7.5 Hz, 1 H, 9-H), 3.64–3.58 (m, 1 H, 7-H), 2.84–2.77 (m, 1 H, 5-Ha), 2.72–2.65 (m, 1 H, 5-Hb), 2.47–2.37 (dq (app sex), *J* = 7.8 Hz, 1 H, 11-H), 2.29–2.23 (m, 1 H, 8-Ha), 2.13–2.06 (m, 1 H, 8-Hb), 1.80–1.73 (m, 4 H, 6-H, 12-Ha), 1.66–1.52 (m, 4 H, 13-H), 1.31–1.23 (m, 3 H, 12-Hb, OH).

^{13}C-NMR (100 MHz, $CDCl_3$) δ: 142.2 (C-4), 139.7 (C-10), 128.4 (C-3), 128.3 (C-2), 125.7 (C-1), 123.5 (C-9), 70.1 (C-7), 43.4 (C-11), 40.8 (C-8), 38.3 (C-6), 33.2 (C-12), 32.0 (C-5), 25.1 (C-13).

HRMS (CI) *m/z*: $[M+H]^+$ calcd for $C_{17}H_{25}O$, 245.1905; found, 245.1905.

(4*R*,5*R*)-4,5-Dicyclohexyl-2-((*R*,*E*)-6-(furan-2-yl)-1-phenylhex-5-en-3-yl)-1,3,2-dioxaborolane (6j)

According to GP-2, 278 mg (2.04 mmol) $ZnCl_2$, 2-furyllithium solution and 3.56 mL anhydrous THF were used for the preparation of the arylzinc reagent. For the 2-furyllithium solution, 0.82 mL *n*BuLi (2.04 mmol, 2.5 M) were added according to literature procedure[10] at -78 °C to a solution of 149 µL furan (149 mg, 2.04 mmol) in 4.07 mL anhydrous THF. The resulting mixture was warmed at room temperature and stirred for 1 h until the addition of the $ZnCl_2$ solution. 106 mg (204 µmol) boronic ester **5c**, 23.5 mg (20.0 µmol) $Pd(PPh_3)_4$, 4.08 mL anhydrous THF and 4.30 mL of the arylzinc reagent solution were used for the reaction mixture. The reaction mixture was worked up after 1 h. After column chromatography (SiO_2, CyH/EtOAc 0-3%), the product **6j** (85.3 mg, 185 µmol, 91%) was obtained as a colorless oil. R_f (**6j**) = 0.23 (pentane/EtOAc 97:3). $[\alpha]_D^{20}$ = +23.5 (c = 1.0, $CHCl_3$).

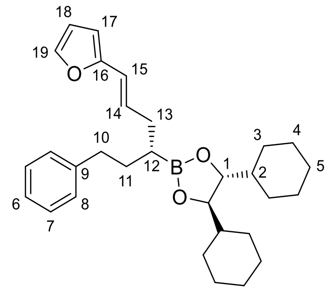

^{1}H-NMR (400 MHz, $CDCl_3$) δ: 7.29–7.25 (m, 3 H, 7-H, 19-H), 7.19–7.15 (m, 3 H, 6-H, 8-H), 6.32 (dd, *J* = 3.2 Hz, 1.8 Hz, 1 H, 18-H), 6.23–6.13 (m, 2 H, 14-H, 15-H), 6.10 (d, *J* = 3.1 Hz, 1 H, 17-H), 3.87–3.84 (m, 2 H, 1-H), 2.71–2.58 (m, 2 H, 10-H), 2.39–2.28 (m, 2 H, 13-H), 1.81–1.59 (m, 12 H, 3-Ha, 3-Hb, 4-Ha, 4-Hb, 5-Ha, 11-H), 1.32–0.95 (m, 13 H, 2-H, 3-Hc, 3-Hd, 4-Hc, 4-Hd, 5-Hb, 12-H).

^{13}C-NMR (100 MHz, $CDCl_3$) δ: 153.3 (C-16), 142.9 (C-9), 141.1 (C-19), 129.5 (C-14), 128.4 (C-8), 128.2 (C-7), 125.6 (C-6), 119.1 (C-15), 111.0 (C-18), 105.9 (C-17), 83.5 (C-1), 43.8 (C-2), 35.6 (C-10), 34.6 (C-13), 33.3 (C-11), 28.5 (C-3a), 27.6 (C-3b), 26.4 (C-5), 26.0 (C-4a), 25.8 (C-4b). The signal of C-12 could not be detected.

HRMS (CI) *m/z*: $[M-H]^+$ calcd for $C_{30}H_{40}BO_3$, 459.3071; found, 459.3054.

2-((*R*,*E*)-4-((4*R*,5*R*)-4,5-Dicyclohexyl-1,3,2-dioxaborolan-2-yl)-6-phenylhex-1-en-1-yl)-1-methyl-1*H*-indole (6k)

According to GP-2, 278 mg (2.04 mmol) $ZnCl_2$, (1-methyl-1*H*-indol-2-yl)lithium solution and 3.56 mL anhydrous THF were used for the preparation of the arylzinc reagent. For the (1-methyl-1*H*-indol-2-yl)lithium solution, 0.82 mL (2.04 mmol, 2.5 M, 10.0 eq.) *n*-BuLi were added dropwise at −78 °C to a solution of 254 µL (267 mg, 2.04 mmol, 10.0 eq.) 1-methyl-1*H*-indol in 2.17 mL anhydrous THF. The reaction mixture was stirred for 5 min at the same temperature and was warmed to room temperature until the $ZnCl_2$ solution was added. 106 mg (204 µmol) boronic ester **5c**, 23.5 mg (23.0 µmol) $Pd(PPh_3)_4$, 4.08 mL anhydrous THF and

3.28 mL of the arylzinc reagent solution were used for the reaction mixture. The reaction was worked up after 2 h. After column chromatography (SiO_2, CyH/EtOAc 0-4%), the product **6k** (104 mg, 198 µmol, 97%) was obtained as a yellow oil. R_f (**6k**) = 0.24 (pentane/EtOAc 97:3). $[\alpha]_D^{20}$ = +20.4 (c = 1.0, $CHCl_3$).

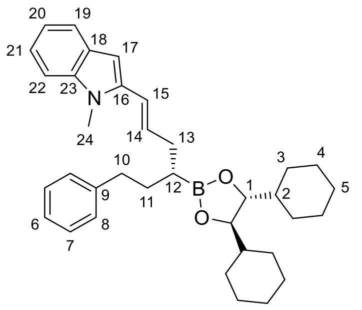

^{1}H-NMR (400 MHz, $CDCl_3$) δ: 7.53 (d, *J* = 7.8 Hz, 1 H, 19-H), 7.29–7.24 (m, 3 H, 7-H, 22-H), 7.20–7.13 (m, 4 H, 6-H, 8-H, 21-H), 7.03–7.04 (m, 1 H, 20-H), 6.54–6.44 (m, 2 H, 15-H, 17-H), 6.35–6.28 (m, 1 H, 14-H), 3.88–3.87 (m, 2 H, 1-H), 3.71 (s, 3 H, 24-H), 2.74–2.61 (m, 2 H, 10-H), 2.49–2.36 (m, 2 H, 13-H), 1.88–1.60 (m, 12 H, 3-Ha, 3-Hb, 4-Ha, 4-Hb, 5-Ha, 11-H), 1.35–0.94 (m, 13 H, 2-H, 3-Hc, 3-Hd, 4-Hc, 4-Hd, 5-Hb, 12-H).

^{13}C-NMR (100 MHz, $CDCl_3$) δ: 142.9 (C-9), 138.7 (C-15), 137.6 (C-23), 133.9 (C-16), 128.4 (C-8), 128.3 (C-7), 128.0 (C-18), 125.6 (C-6), 121.0 (C-21), 120.0 (C-19), 119.5 (C-20), 119.4 (C-14), 108.9 (C-22), 97.9 (C-17), 83.5 (C-1), 43.1 (C-2), 35.6 (C-10), 35.2 (C-13), 33.3 (C-11), 29.8 (C-24), 28.5 (C-3a), 27.6 (C-3b), 26.4 (C-5), 26.0 (C-4a), 25.8 (C-4b). The signal of C-12 could not be detected.

HRMS (CI) *m/z*: $[M+H]^+$ calcd for $C_{35}H_{47}BNO_2$, 524.3700; found, 524.3697.

2-((*R*,*E*)-4-((4*R*,5*R*)-4,5-Dicyclohexyl-1,3,2-dioxaborolan-2-yl)-6-phenylhex-1-en-1-yl)-pyridine (6l)

According to GP-2, 313 mg (2.30 mmol) $ZnCl_2$, pyridin-2-yllithium solution and 3.40 mL anhydrous THF were used for the preparation of the arylzinc reagent. For the 2-pyridin-2-yllithium solution,[11] 0.92 mL (2.30 mmol, 2.5 M, 10.0 eq.) *n*-BuLi were added dropwise at −78 °C to a solution of 219 µL (2.30 mmol, 10.0 eq.) 2-bromopyridine in 4.59 mL anhydrous THF. The reaction mixture was stirred for 20 min at the same temperature until the $ZnCl_2$ solution was added. 119 mg (230 µmol) boronic ester **5c**, 26.5 mg (23.0 µmol) $Pd(PPh_3)_4$, 4.59 mL anhydrous THF and 4.31 mL of the arylzinc reagent solution were used for the reaction mixture. The reaction was worked up after 1 h. After column chromatography (SiO_2, CyH/EtOAc 0-3%), the product **6l** (99.8 mg, 212 µmol, 92%) was obtained as a yellow oil. R_f (**6l**) = 0.36 (pentane/EtOAc 97:3). $[\alpha]_D^{20}$ = +17.8 (c = 1.0, $CHCl_3$).

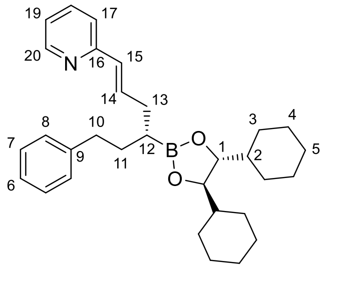

^{1}**H-NMR** (400 MHz, $CDCl_3$) δ: 8.51 (d, *J* = 4.0 Hz, 1 H, 20-H), 7.57 (ddd, *J* = 7.7 Hz, 1.8 Hz, 1.8 Hz, 1 H, 18-H), 7.28–7.22 (m, 3 H, 7-H, 17-H), 7.19–7.14 (m, 3 H, 6-H, 8-H), 7.07 (dd, *J* = 6.6 Hz, 4.9 Hz, 1 H, 19-H), 6.72 (dt, J = 15.7 Hz, 7.3 Hz, 1 H, 14-H), 6.49 (d, J = 15.6 Hz, 1 H, 15-H), 3.87–3.84 (m, 2 H, 1-H), 2.72–2.59 (m, 2 H, 10-H), 2.49–2.35 (m, 2 H, 13-H), 1.85–1.57 (m, 12 H, 3-Ha, 3-Hb, 4-Ha, 4-Hb, 5-Ha, 11-H), 1.35–0.93 (m, 13 H, 2-H, 3-Hc, 3-Hd, 4-Hc, 4-Hd, 5-Hb, 12-H).

^{13}**C-NMR** (100 MHz, $CDCl_3$) δ: 156.1 (C-16), 149.3 (C-20), 142.9 (C-9), 136.2 (C-18), 135.4 (C-14), 130.6 (C-15), 128.4 (C-8), 128.2 (C-7), 125.6 (C-6), 121.4 (C-19), 120.7 (C-17), 83.4 (C-1), 43.0 (C-2), 35.6 (C-10), 34.7 (C-13), 33.4 (C-11), 28.5 (C-3a), 27.5 (C-3b), 26.4 (C-5), 26.0 (C-4a), 25.8 (C-4b). The signal of C-12 could not be detected.

HRMS (CI) *m/z*: $[M+H]^+$ calcd for $C_{31}H_{43}BNO_2$, 472.3387; found, 472.3391.

(4*R*,5*R*)-4,5-Dicyclohexyl-2-((*R*,*E*)-1,7-diphenylhept-5-en-3-yl)-1,3,2-dioxaborolane (6m)

According to GP-2, 265 mg (1.94 mmol) $ZnCl_2$, 2.16 mL (1.94 mmol, 0.9 M) benzylmagnesium chloride and 3.40 mL anhydrous THF were used for the preparation of the arylzinc reagent. 101 mg (194 µmol) boronic ester **5c**, 22.4 mg (19 µmol) $Pd(PPh_3)_4$, 3.88 mL anhydrous THF and 2.78 mL of the arylzinc reagent solution were used for the reaction mixture. The reaction was worked up after 2 h. After column chromatography (SiO_2, CyH/EtOAc 0-4%), the product **6m** (64.5 mg, 133 µmol, 69%) was obtained as a colorless oil. R_f (**6m**) = 0.33 (pentane/EtOAc 97:3). $[\alpha]_D^{20}$ = +21.9 (c = 1.0, $CHCl_3$).

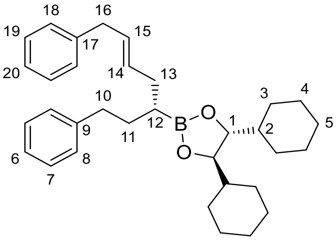

^{1}**H-NMR** (400 MHz, $CDCl_3$) δ: 7.29–7.24 (m, 4 H, 7-H, 19-H), 7.19–7.14 (m, 6 H, 6-H, 8-H, 18-H, 20-H), 5.20–5.48 (m, 2 H, 14-H, 15-H), 3.85–3.82 (m, 2 H, 1-H), 3.31 (d, *J* = 6.0 Hz, 2 H, 16-H), 2.66–2.57 (m, 2 H, 10-H), 2.26–2.14 (m, 2 H, 13-H), 1.82–1.59 (m, 12 H, 3-Ha, 3-Hb, 4-Ha, 4-Hb, 5-Ha, 11-H), 1.32–0.94 (m, 13 H, 2-H, 3-Hc, 3-Hd, 4-Hc, 4-Hd, 5-Hb, 12-H).

^{13}**C-NMR** (100 MHz, $CDCl_3$) δ: 143.1 (C-9), 141.0 (C-17), 131.4 (C-14), 129.5 (C-15), 128.5 (C-18), 128.4 (C-8), 128.3 (C-19), 128.2 (C-7), 125.8 (C-20), 125.5 (C-6), 83.4 (C-1), 43.1 (C-2), 39.1 (C-16), 35.5 (C-10), 34.0 (C-13), 33.0 (C-11), 28.5 (C-3a), 27.6 (C-3b), 26.5 (C-5), 26.0 (C-4a), 25.9 (C-4b). The signal of C-12 could not be detected.

HRMS (CI) *m/z*: $[M]^+$ calcd for $C_{33}H_{45}BO_2$, 484.3513; found, 484.3506.

(4*R*,5*R*)-4,5-Dicyclohexyl-2-((*R*,*E*)-1-phenylocta-5,7-dien-3-yl)-1,3,2-dioxaborolane (6n)

According to GP-2, 263 mg (1.93 mmol) $ZnCl_2$, 1.93 mL (1.93 mmol, 1.0 M) vinylmagnesium bromide and 3.38 mL anhydrous THF were used for the preparation of the arylzinc reagent. 101 mg (193 µmol) boronic ester **5c**, 22.3 mg (19.0 µmol) $Pd(PPh_3)_4$, 3.87 mL anhydrous THF and 2.66 mL of the arylzinc reagent solution were used for the reaction mixture. The reaction mixture was worked up after 2 h. After column chromatography (SiO_2, CyH/EtOAc 0-3%), the product **6n** (76.2 mg, 181 µmol, 94%) was obtained as a colorless oil. R_f (**6n**) = 0.31 (pentane/EtOAc 97:3). $[\alpha]_D^{20}$ = +32.7 (c = 1.0, $CHCl_3$).

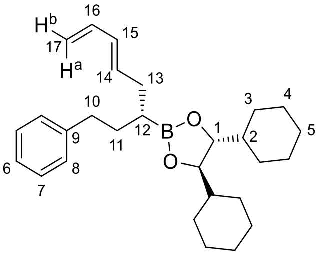

^{1}H-NMR (400 MHz, $CDCl_3$) δ: 7.29–7.25 (m, 2 H, 7-H), 7.18–7.15 (m, 3 H, 6-H, 8-H), 6.28 (ddd, *J* = 17.0 Hz, 10.3 Hz, 10.3 Hz, 1 H, 16-H), 6.05 (dd, *J* = 15.1 Hz, 10.4 Hz, 1 H, 15-H), 5.71 (dt, *J* = 15.0 Hz, 7.3 Hz, 1 H, 14-H), 5.06 (d, *J* = 17.0 Hz, 1 H, 17-Ha), 4.94 (d, *J* = 10.2 Hz, 1 H, 17-Hb), 3.86–3.83 (m, 2 H, 1-H), 2.69–2.56 (m, 2 H, 10-H), 2.31–2.19 (m, 2 H, 13-H), 1.82–1.59 (m, 12 H, 3-Ha, 3-Hb, 4-Ha, 4-Hb, 5-Ha, 11-H), 1.32–0.92 (m, 13 H, 2-H, 3-Hc, 3-Hd, 4-Hc, 4-Hd, 5-Hb, 12-H).

^{13}C-NMR (100 MHz, $CDCl_3$) δ: 143.0 (C-9), 137.3 (C-16), 134.8 (C-14), 131.5 (C-15), 128.4 (C-8), 128.2 (C-7), 125.6 (C-6), 114.7 (C-17), 83.5 (C-1), 43.1 (C-2), 35.6 (C-10), 34.3 (C-13), 33.3 (C-11), 28.5 (C-3a), 27.6 (C-3b), 26.5 (C-5), 26.0 (C-4a), 25.9 (C-4b). The signal of C-12 could not be detected.

HRMS (CI) *m/z*: $[M]^+$ calcd for $C_{28}H_{41}BO_2$, 420.3200; found, 420.3234.

Methyl (*R*,2*E*,4*E*)-7-((4*R*,5*R*)-4,5-dicyclohexyl-1,3,2-dioxaborolan-2-yl)-9-phenylnona-2,4-dienoate (8a)

103 mg (119 µmol, 1.0 eq.) boronic ester **5c**, 54.0 µL (51.3 mg, 596 µmol, 3.0 eq.) methyl acrylate, 2.2 mg (9.94 µmol, 5 mol-%) $PdOAc_2$, 70.5 mg (219 µmol, 1.1 eq.) tetrabutylammonium bromide and 68.7 mg (497 µmol, 2.5 eq.) K_2CO_3 were suspended in 0.66 mL anhydrous DMF under an argon atmosphere. The reaction mixture was stirred at room temperature for 18.5 h.

Ethyl acetate was added and the mixture was washed with water and brine. The organic phase was dried over Na_2SO_4, filtered and the solvent was removed under reduced pressure. After purification by column chromatography (SiO_2, CyH/EtOAc 0-5%), the product **8a** (69.8 mg, 146 µmol, 73%) was obtained as a yellow oil.

1 mmol scale

750 mg (1.44 mmol, 1.0 eq.) boronic ester **5c**, 392 µL (372 mg, 4.32 mmol, 3.0 eq.) methyl acrylate, 16.2 mg (72.0 µmol, 5 mol-%) $PdOAc_2$, 511 mg (1.59 mmol, 1.1 eq.) tetrabutylammonium bromide and 498 mg (3.60 mmol, 2.5 eq.) K_2CO_3 were suspended in 4.81 mL anhydrous DMF under an argon atmosphere. The reaction mixture was stirred at room temperature for 18.5 h.

Ethyl acetate was added and the mixture was washed with water and brine. The organic phase was dried over Na_2SO_4, filtered and the solvent was removed under reduced pressure. After purification by column chromatography (SiO_2, CyH/EtOAc 0-5%), the product **8a** (517 mg, 1.08 mmol, 75%) was obtained as a yellow oil R_f (**8a**) = 0.26 (pentane/EtOAc 95:5). $[\alpha]_D^{20}$ = +23.6 (c = 1.0, $CHCl_3$).

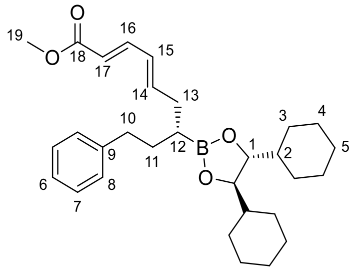

^{1}H-NMR (400 MHz, $CDCl_3$) δ: 7.29–7.23 (m, 3 H, 7-H, 16-H), 7.21–7.15 (m, 3 H, 6-H, 8-H), 6.21–6.09 (m, 2 H, 14-H, 15-H), 5.76 (d, J = 15.3 Hz, 1 H, 17-H), 3.86–3.83 (m, 2 H, 1-H), 3.73 (s, 3 H, 19-H), 2.69–2.56 (m, 2 H, 10-H), 2.38–2.26 (m, 2 H, 13-H), 1.82–1.57 (m, 12 H, 3-Ha, 3-Hb, 4-Ha, 4-Hb, 5-Ha, 11-H), 1.32–0.91 (m, 13 H, 2-H, 3-Hc, 3-Hd, 4-Hc, 4-Hd, 5-Hb, 12-H).

^{13}C-NMR (100 MHz, $CDCl_3$) δ: 167.7 (C-18), 145.3 (C-16), 144.5 (C-14), 142.7 (C-9), 128.9 (C-15), 128.4 (C-8), 128.3 (C-7), 125.7 (C-6), 118.7 (C-17), 83.5 (C-1), 51.4 (C-19), 43.1 (C-2), 35.5 (C-10), 34.7 (C-13), 33.3 (C-11), 28.5 (C-3a), 27.6 (C-3b), 26.4 (C-5), 26.0 (C-4a), 25.9 (C-4b). The signal of C-12 could not be detected.

HRMS (CI) *m/z*: $[M]^+$ calcd for $C_{30}H_{43}BO_4$, 478.3254; found, 478.3236.

(*R*,4*E*)-7-((4*R*,5*R*)-4,5-Dicyclohexyl-1,3,2-dioxaborolan-2-yl)-9-phenylnona-2,4-dienenitrile (8b)

118 mg (226 µmol, 1.0 eq.) boronic ester **5c**, 44.7 µL (36.0 mg, 679 µmol, 3.0 eq.) acrylonitrile, 2.5 mg (11.0 µmol, 5 mol-%) $PdOAc_2$, 80.0 mg (249 µmol, 1.1 eq.) tetrabutylammonium bromide and 78.0 mg (566 µmol, 2.5 eq.) K_2CO_3 were suspended in 0.76 mL anhydrous DMF under an argon atmosphere. The reaction mixture was stirred at room temperature for 18 h.

Ethyl acetate was added and the mixture was washed with water and brine. The organic phase was dried over Na_2SO_4, filtered and the solvent was removed under reduced pressure. After purification by column chromatography (SiO_2, CyH/EtOAc 0-5%), the product **8b** (75.2 mg, 169 µmol, 75%) was obtained as a yellow oil. R_f (**8b**) = 0.31 (pentane/EtOAc 95:5). $[\alpha]_D^{20}$ = +25.8 (c = 1.0, $CHCl_3$).

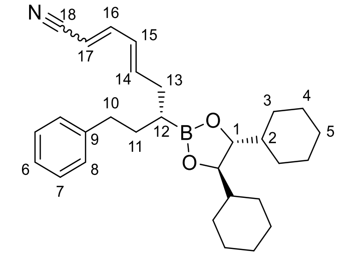

Major double bond isomer (E,E isomer):

^{1}H-NMR (400 MHz, $CDCl_3$) δ: 7.29–7.26 (m, 2 H, 7-H), 7.19–7.15 (m, 3 H, 6-H, 8-H), 6.98–6.91 (m, 1 H, 16-H), 6.14–6.12 (m, 2 H, 14-H, 15-H), 5.21 (d, J = 16.0 Hz, 1 H, 17-H), 3.87–3.85 (m, 2 H, 1-H), 2.70–2.55 (m, 2 H, 10-H), 2.44–2.26 (m, 2 H, 13-H), 1.79–1.58 (m, 12 H, 3-Ha, 3-Hb, 4-Ha, 4-Hb, 5-Ha, 11-H), 1.33–0.94 (m, 13 H, 2-H, 3-Hc, 3-Hd, 4-Hc, 4-Hd, 5-Hb, 12-H).

^{13}C-NMR (100 MHz, $CDCl_3$) δ: 150.8 (C-16), 145.6 (C-14), 142.5 (C-9), 128.4 (C-8), 128.4 (C-7), 128.3 (C-15), 125.7 (C-6), 116.6 (C-18), 96.4 (C-17), 83.5 (C-1), 43.1 (C-2), 35.4 (C-

10), 34.7 (C-13), 33.3 (C-11), 28.5 (C-3a), 27.5 (C-3b), 26.4 (C-5), 26.0 (C-4a), 25.9 (C-4b). The signal of C-12 could not be detected.

Minor double bond isomer (Z,E isomer, selected signals):

^{1}H-NMR (400 MHz, $CDCl_3$) δ: 6.76 (dd, *J* = 10.9 Hz, 1 H, 16-H), 6.56 (dd, *J* = 15.0 Hz, 11.1 Hz, 1 H, 15-H), 6.18 (dt, *J* = 14.9 Hz, 7.3 Hz, 1 H, 14-H), 5.08 (d, *J* = 10.8 Hz, 1 H, 17-H).

^{13}C-NMR (100 MHz, $CDCl_3$) δ: 149.7 (C-16), 146.0 (C-14), 142.6 (C-9), 127.4 (C-15), 118.4 (C-18), 94.8 (C-17), 43.0 (C-2), 34.7 (C-13). The signal of C-12 could not be detected.

HRMS (CI) *m/z*: $[M-2H]^+$ calcd for $C_{29}H_{38}BNO_2$, 443.2996; found, 443.3004.

Ethyl (*R*,2*E*,4*E*)-7-((4*R*,5*R*)-4,5-dicyclohexyl-1,3,2-dioxaborolan-2-yl)-3-methyl-9-phenylnona-2,4-dienoate (8c)

100 mg (192 µmol, 1.0 eq.) boronic ester **5c**, 53.0 µL (48.3 mg, 423 µmol, 2.2 eq.) ethyl crotonate, 4.3 mg (19.0 µmol, 10 mol-%) $Pd(OAc)_2$, 58.9 µL (42.8 mg, 423 µmol, 2.2 eq.) triethylamine and 58.3 mg (211 µmol, 1.1 eq.) Ag_2CO_3 were suspended in 1.92 mL anhydrous DCM. The reaction mixture was stirred at 40 °C for 17 h.

An 85:15 (v/v) mixture of pentane/ethyl acetate was added at 0 °C. The mixture was filtered over Celite and rinsed with pentane. The organic phase was dried over Na_2SO_4, filtered and the solvent was removed under reduced pressure. After purification by column chromatography (SiO_2, CyH/EtOAc 0-5%), the product **8c** (77.5 mg, 153 µmol, 80%) was obtained as a yellow oil. R_f (**8c**) = 0.29 (pentane/EtOAc 95:5). $[\alpha]_D^{20}$ = +33.6 (c = 1.0, $CHCl_3$).

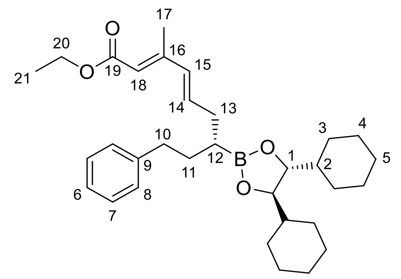

^{1}H-NMR (400 MHz, $CDCl_3$) δ: 7.29–7.24 (m, 2 H, 7-H), 7.19–7.13 (m, 3 H, 6-H, 8-H), 6.18–6.07 (m, 2 H, 14-H, 15-H), 5.67 (s, 1 H, 18-H), 4.16 (q, *J* = 7.1 Hz, 2 H, 20-H), 3.86–3.83 (m, 2 H, 1-H), 2.65–2.59 (m, 2 H, 10-H), 2.35–2.28 (m, 2 H, 13-H), 2.25 (s, 3 H, 17-H), 1.80–1.61 (m, 12 H, 3-Ha, 3-Hb, 4-Ha, 4-Hb, 5-Ha, 11-H), 1.29–0.92 (m, 16 H, 2-H, 3-Hc, 3-Hd, 4-Hc, 4-Hd, 5-Hb, 12-H, 21-H).

^{13}C-NMR (100 MHz, $CDCl_3$) δ: 167.3 (C-19), 152.6 (C-16), 142.8 (C-9), 137.0 (C-15), 134.2 (C-14), 128.4 (C-8), 128.3 (C-7), 125.6 (C-6), 117.7 (C-18), 83.5 (C-1), 59.6 (C-20), 43.1 (C-2), 35.5 (C-10), 34.9 (C-13), 33.4 (C-11), 28.5 (C-3a), 27.5 (C-3b), 26.4 (C-5), 26.0 (C-4a), 25.8 (C-4b), 14.3 (C-21), 13.8 (C-17). The signal of C-12 could not be detected.

HRMS (CI) *m/z*: $[M+H]^+$ calcd for $C_{32}H_{48}BO_4$, 507. 3646; found, 507.3666.

Ethyl (*R*,2*Z*,4*E*)-7-((4*R*,5*R*)-4,5-dicyclohexyl-1,3,2-dioxaborolan-2-yl)-3-methyl-9-phenylnona-2,4-dienoate (8d)

102 mg (197 µmol, 1.0 eq.) boronic ester **5c**, 82.0 mg (433 µmol, 2.2 eq.) ethyl (*Z*)-but-2-enoate, 4.4 mg (20.0 µmol, 10 mol-%) $Pd(OAc)_2$, 60.3 µL (43.8 mg, 433 µmol, 2.2 eq.)

triethylamine and 59.6 mg (216 µmol, 1.1 eq.) Ag_2CO_3 were suspended in 1.97 mL anhydrous DCM. The reaction mixture was stirred at 40 °C for 17 h.

An 85:15 (v/v) mixture of pentane/ethyl acetate was added at 0 °C. The mixture was filtered over Celite and rinsed with pentane. The organic phase was dried over Na_2SO_4, filtered and the solvent was removed under reduced pressure. After purification by column chromatography (SiO_2, CyH/EtOAc 0-5%), the product **8d** (70.3 mg, 139 µmol, 71%) was obtained as a yellow oil. R_f (**8d**) = 0.32 (pentane/EtOAc 95:5). $[\alpha]_D^{20}$ = +42.3 (c = 1.0, $CHCl_3$).

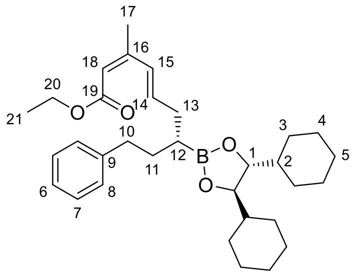

¹H-NMR (500 MHz, $CDCl_3$) δ: 7.58 (d, *J* = 15.8 Hz,1 H, 15-H), 7.28–7.24 (m, 2 H, 7-H), 7.18–7.15 (m, 3 H, 6-H, 8-H), 6.14 (dt, *J* = 15.8 Hz, 7.6 Hz, 1 H, 14-H), 5.59 (s, 1 H, 18-H), 4.15 (q, *J* = 7.1 Hz, 2 H, 20-H), 3.86–3.84 (m, 2 H, 1-H), 2.68–2.58 (m, 2 H, 10-H), 2.42–2.33 (m, 2 H, 13-H), 1.96 (d, *J* = 1.3 Hz, 3 H, 17-H), 1.80–1.61 (m, 12 H, 3-Ha, 3-Hb, 4-Ha, 4-Hb, 5-Ha, 11-H), 1.32–1.30 (m, 2 H, 2-H), 1.27 (t, *J* = 7.1 Hz, 3 H, 21-H), 1.24–0.93 (m, 11 H, 3-Hc, 3-Hd, 4-Hc, 4-Hd, 5-Hb, 12-H).

¹³C-NMR (125 MHz, $CDCl_3$) δ: 166.3 (C-19), 151.1 (C-16), 142.9 (C-9), 138.8 (C-14), 128.4 (C-8, C-15), 128.2 (C-7), 125.6 (C-6), 115.8 (C-18), 83.4 (C-1), 59.6 (C-20), 43.1 (C-2), 35.5 (C-10), 35.2 (C-13), 33.4 (C-11), 28.5 (C-3a), 27.5 (C-3b), 26.4 (C-5), 26.0 (C-4a), 25.9 (C-4b), 21.1 (C-17), 14.3 (C-21). The signal of C-12 could not be detected.

HRMS (ESI) *m/z*: $[M+H]^+$ calcd for $C_{32}H_{48}BO_4$, 507.3640; found, 507.3619.

((*R*,*Z*)-5-((4*R*,5*R*)-4,5-Dicyclohexyl-1,3,2-dioxaborolan-2-yl)-7-phenylhept-2-en-2-yl)trimethylsilane (9)

According to GP-2, 661 mg (4.85 mmol) $ZnCl_2$, 1.62 mL (4.85 mmol, 3.0 M) methylmagnesium bromide solution and 8.49 mL anhydrous THF were used for the preparation of the arylzinc reagent. 287 mg (485 µmol) boronic ester **4a**, 56.1 mg (48.5 µmol) $Pd(PPh_3)_4$, 9.70 mL anhydrous THF and 5.06 mL of the arylzinc reagent solution were used for the reaction mixture. The reaction mixture was worked up after 2 h. After column chromatography (SiO_2, CyH/EtOAc 0-3%), the product **9** (207 mg, 430 µmol, 89%) was obtained as a colorless oil. R_f (**9**) = 0.29 (pentane/EtOAc 97:3). $[\alpha]_D^{20}$ = +30.7 (c = 1.0, $CHCl_3$).

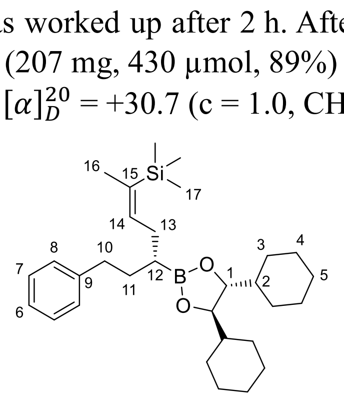

¹H-NMR (400 MHz, $CDCl_3$) δ: 7.28–7.25 (m, 2 H, 7-H), 7.20–7.14 (m, 3 H, 6-H, 8-H), 5.96 (dt, *J* = 7.2 Hz, 1.3 Hz, 1 H, 14-H), 3.86–3.84 (m, 2 H, 1-H), 2.68–2.59 (m, 2 H, 10-H), 2.29–

2.16 (m, 2 H, 13-H), 1.82–1.60 (m, 15 H, 3-Ha, 3-Hb, 4-Ha, 4-Hb, 5-Ha, 11-H, 16-H), 1.33–0.94 (m, 13 H, 2-H, 3-Hc, 3-Hd, 4-Hc, 4-Hd, 5-Hb, 12-H), 0.12 (s, 9 H, 17-H).

^{13}C-NMR (100 MHz, $CDCl_3$) δ: 143.1 (C-14), 142.3 (C-9), 134.6 (C-15), 128.4 (C-8), 128.2 (C-7), 125.5 (C-6), 83.3 (C-1), 43.1 (C-2), 35.8 (C-10), 33.7 (C-11), 33.6 (C-13), 28.5 (C-3a), 27.5 (C-3b), 26.5 (C-5), 26.0 (C-4a), 25.9 (C-4b), 24.6 (C-16), −0.17 (C-17). The signal of C-12 could not be detected.

HRMS (ESI) *m/z*: $[M+H]^+$ calcd for $C_{30}H_{50}BO_2Si$, 481.3668; found, 481.3657.

(*R*,*Z*)-6-methyl-1-phenyloct-5-en-3-ol (12)

To a solution of 183 mg (381 µmol, 1.0 eq.) boronic ester **9** in 3.81 mL anhydrous DCM was added 97.0 mg (381 µmol, 1.0 eq.) iodine. The reaction mixture was stirred for 1 h at room temperature. Aqueous $Na_2S_2O_3$ solution (50%) was added and the phases were separated. The aqueous phase was extracted three times with DCM. The combined organic phases were washed twice with brine, dried over Na_2SO_4 and filtered. The solvent was removed under reduced pressure. The residue was purified by column chromatography (SiO_2, CyH/EtOAc 0-3%). The product **10** (184 mg, 345 µmol, 90%) was obtained as a colorless oil.

According to GP-4, 427 mg (3.13 mmol) $ZnCl_2$, 1.04 mL (3.13 mmol, 3.0 M) methylmagnesium bromide solution and 5.48 mL anhydrous THF were used for the preparation of the arylzinc reagent. 167 mg (313 µmol) boronic ester **10**, 36.2 mg (31.3 µmol) $Pd(PPh_3)_4$, 6.26 mL anhydrous THF and 3.26 mL of the arylzinc reagent solution were used for the reaction mixture. The reaction mixture was worked up after 2 h. After column chromatography (SiO_2, CyH/EtOAc 0-3%), the product **11** (117 mg, 269 µmol, 86%) was obtained as a colorless oil.

111 mg (254 µmol) boronic esters **11** was subjected to oxidation according to GP-3 using 508 µL THF, 116 µL (43.2 mg, 1.27 mmol, 33 %wt) aqueous H_2O_2 solution and a solution of 50.8 mg (1.27 mmol) NaOH in 508 µL water. The reaction was worked up after 1 h. To improve chromatographic separation, 18.2 mg (305 µmol) methylboronic acid and 1.27 mL diethyl ether were used. After column chromatography (SiO_2, CyH/EtOAc 0-20%; C18-SiO_2, $H_2O \rightarrow$ MeCN), the product **12** (40.8 mg, 187 µmol, 74%) was obtained as a colorless oil. R_f (**12**) = 0.33 (pentane/EtOAc 8:2). $[\alpha]_D^{20}$ = +6.5 (c = 1.0, $CHCl_3$).

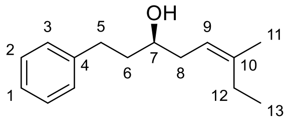

^{1}H-NMR (400 MHz, $CDCl_3$) δ: 7.30–7.27 (m, 2 H, 2-H), 7.22–7.17 (m, 3 H, 1-H, 3-H), 5.18–5.10 (m, 1 H, 9-H), 3.67–3.58 (m, 1 H, 7-H), 2.85–2.78 (m, 1 H, 5-Ha), 2.72–2.65 (m, 1 H, 5-Hb), 2.23–2.18 (m, 2 H, 8-H), 2.09–2.00 (m, 2 H, 12-H), 1.82–1.76 (m, 2 H, 6-H), 1.73 (s, 3 H, 11-H), 1.64 (s, 1 H, OH), 0.99 (m, 3 H, 13-H).

^{13}C-NMR (100 MHz, $CDCl_3$) δ: 142.2 (C-4), 141.2 (C-10), 128.4 (C-3), 128.3 (C-2), 125.7 (C-1), 119.4 (C-9), 70.9 (C-7), 38.4 (C-6), 35.9 (C-8), 32.1 (C-5), 24.9 (C-12), 16.3 (C-11), 12.8 (C-13).

HRMS (ESI) *m/z*: $[M+H]^+$ calcd for $C_{15}H_{23}O$, 219.1743; found, 219.1740.

tert-Butyl(((2S,3R)-2-((4R,5R)-4,5-dicyclohexyl-1,3,2-dioxaborolan-2-yl)-4-methylpentan-3-yl)oxy)dimethylsilane (13)

Boronic ester **13** was prepared according to the procedure by M. Tost and U. Kazmaier.[12]

R_f (**13**) = 0.31 (pentane/EtOAc 98:2). $[\alpha]_D^{20}$ = +34.6 (c = 1.0, $CHCl_3$).

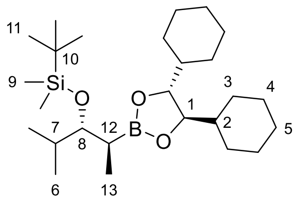

^{1}H-NMR (400 MHz, $CDCl_3$) δ: 3.79–3.76 (m, 2 H, 1-H), 3.55 (dd, J = 7.7 Hz, 2.6 Hz, 1 H, 8-H), 1.82–1.56 (m, 10 H, 3-Ha, 3-Hb, 4-Ha, 4-Hb, 5-Ha), 1.35–1.11 (m, 10 H, 2-H, 4-Hc, 4-Hd, 5-Hb, 7-H, 12-H), 1.05–0.93 (m, 7 H, 3-Hc, 3-Hd, 13-H) 0.92–0.90 (m, 12 H, 6-Ha, 11-H), 0.79 (d, J = 6.9 Hz, 2 H, 6-Hb), 0.05 (s, 3 H, 9-Ha), 0.04 (s, 3 H, 9-Hb).

^{13}C-NMR (100 MHz, $CDCl_3$) δ: 83.3 (C-1), 80.0 (C-8), 43.0 (C-2), 32.7 (C-7), 28.5 (C-3a), 27.7 (C-3b), 26.5 (C-5), 26.0 (C-4a), 25.9 (C-4b, C-11), 20.3 (C-6a), 19.4 (C-6b), 18.2 (C-10), 10.1 (C-13), −4.3 (C-9a), −4.4 (C-9b). The signal of C-12 could not be detected.

HRMS (CI) *m/z*: $[M-H]^+$ calcd for $C_{26}H_{50}BO_3Si$, 449.3617; found, 449.3617.

tert-Butyl(((3S,4R,5S)-5-((4R,5R)-4,5-dicyclohexyl-1,3,2-dioxaborolan-2-yl)-2,4-dimethyloct-7-yn-3-yl)oxy)dimethylsilane (14)

According to GP-1, 0.32 mL (229 mg, 2.26 mmol) *N*,*N*-diisopropylamine, 1.31 mL (2.10 mmol, 1.6 M) *n*-butyllithium and 0.34 mL anhydrous THF were used for the LDA solution. 755 mg (1.68 mmol) boronic ester **13**, 0.32 mL (427 mg, 5.03 mmol) anhydrous DCM, 2.35 mL anhydrous THF and a solution of 457 mg (3.35 mmol) zinc chloride in 2.01 mL anhydrous THF were used for the homologation. The further reaction was performed according to variant A using 11.6 mL (4.19 mmol, 0.36 M) of a solution of (3-(trimethylsilyl)prop-2-yn-1-yl)zinc(II) bromide lithium chloride in anhydrous THF. The nucleophile solution was prepared using 420 mg (9.90 mmol) LiCl, 1.18 g (18.0 mmol) zinc dust, 9.00 mL anhydrous THF, 16.0 µL (34.0 mg, 180 µmol) 1,2-dibromoethane, 58.0 µL (49.0 mg, 450 µmol) TMSCl, 1.47 mL (1.72 g, 9.00 mmol) (3-bromoprop-1-yn-1-yl)trimethylsilane and 9.00 mL anhydrous THF. The rection was worked up after 16 h. After column chromatography (SiO_2, CyH/EtOAc 0-3%), the product **S1** (902 mg) was obtained as a colorless oil. R_f (**S1**) = 0.40 (pentane/EtOAc 97:3). $[\alpha]_D^{20}$ = +22.2 (c = 1.0, $CHCl_3$).

To a solution of 793 mg (1.38 mmol, 1.0 eq.) boronic ester **S1** in 17.2 mL methanol and 1.91 mL diethyl ether was added 381 mg (2.76 mmol, 2.0 eq.) K_2CO_3. The reaction mixture was stirred for 5 d. The reaction mixture was added to saturated NH_4Cl solution and diethyl ether. The phases were separated and the aqueous phase was extracted twice with diethyl ether. The combined organic phases were dried over Na_2SO_4, filtered and concentrated under reduced pressure. The residue was purified by column chromatography (SiO_2, CyH/EtOAc 0-3%). The product **14** (566 mg, 1.13 mmol, 77%) was obtained as a colorless oil. R_f (**14**) = 0.50 (pentane/EtOAc 97:3). $[\alpha]_D^{20}$ = +34.1 (c = 1.0, $CHCl_3$).

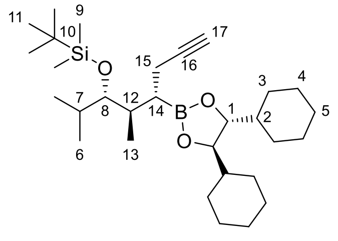

^{1}H-NMR (400 MHz, $CDCl_3$, δ in ppm): 3.83–3.80 (m, 2 H, 1-H), 3.52 (dd, *J* = 7.0 Hz, 2.1 Hz, 1 H, 8-H), 2.37 (ddd, *J* = 16.9 Hz, 7.6 Hz, 2.5 Hz, 1 H, 15-Ha), 2.24 (ddd, *J* = 16.8 Hz, 7.7 Hz, 2.5 Hz, 1 H, 15-Hb), 1.90 (t, *J* = 2.5 Hz, 1 H, 17-H), 1.87–1.59 (m, 13 H, 3-Ha, 3-Hb, 4-Ha, 4-Hb, 5-Ha, 7-H, 12-H, 14-H), 1.33–0.94 (m, 15 H, 2-H, 3-Hc, 3-Hd, 4-Hc, 4-Hd, 5-Hb, 13-H), 0.91 (s, 9 H, 11-H), 0.85 (d, *J* = 7.1 Hz, 3 H, 6-Ha), 0.84 (d, *J* = 6.6 Hz, 3 H, 6-Hb), 0.09 (s, 3 H, 9-Ha), 0.05 (s, 3 H, 9-Hb).

^{13}C-NMR (100 MHz, $CDCl_3$, δ in ppm): 84.8 (C-16), 83.6 (C-1), 78.9 (C-8), 68.4 (C-17), 43.1 (C-2), 39.5 (C-7), 29.9 (C-10), 28.6 (C-3a), 27.5 (C-3b), 26.3 (C-5, C-11), 26.0 (C-4a), 25.9 (C-4b), 21.8 (C-12), 19.1 (C-15), 18.6 (C-13), 16.0 (C-6b), 14.3 (C-6a), −3.5 (C-9a), −4.0 (C-9b). The signal of C-14 could not be detected.

HRMS (CI) *m/z*: $[M]^+$ calcd for $C_{30}H_{55}BO_3Si$, 502.4014; found, 502.4008.

***tert*-Butyl(((3*S*,4*R*,5*S*,*E*)-5-((4*R*,5*R*)-4,5-dicyclohexyl-1,3,2-dioxaborolan-2-yl)-8-iodo-2,4-dimethyloct-7-en-3-yl)oxy)dimethylsilane (15)**

468 mg (931 µmol, 1.0 eq.) alkyne **14** and 288 mg (1.12 mmol, 1.2 eq.) Schwartz reagent were suspended in 2.80 mL anhydrous DCM in the absence of light. The reaction mixture was stirred for 5 min at room temperature. Then, 295 mg (1.16 mmol, 1.25 eq.) iodine were added and the reaction mixture was stirred for additional 30 min at room temperature.

Saturated $Na_2S_2O_3$ solution was added and the phases were separated. The aqueous phase was extracted three times with diethyl ether. The combined organic phases were washed with brine, dried over $MgSO_4$ and filtered. The solvent was removed under reduced pressure. The residue was purified by column chromatography (SiO_2, CyH/EtOAc 0-3%). The product **15** (320 mg, 508 µmol, 55%) was obtained as a colorless oil. R_f (**15**) = 0.47 (pentane/EtOAc 97:3). $[\alpha]_D^{20}$ = +21.1 (c = 1.0, $CHCl_3$).

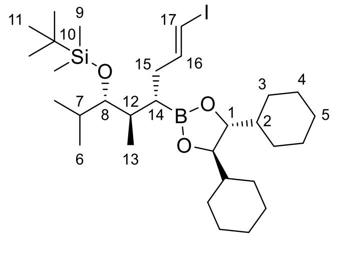

^{1}H-NMR (400 MHz, C_6D_6, δ in ppm): 6.65 (dt, *J* = 14.3 Hz, 7.2 Hz, 1 H, 16-H), 5.94 (d, *J* = 14.3 Hz, 1 H, 17-H), 3.72–3.65 (m, 3 H, 1-H, 8-H), 2.36–2.28 (m, 1 H, 15-Ha), 2.06–1.99 (m, 1 H, 15-Hb), 1.92–1.78 (m, 6 H, 3-Ha, 4-Ha, 7-H, 12-H), 1.70–1.63 (m, 5 H, 3-Hb, 4-Hb, 14-H), 1.29–1.11 (m, 10 H, 2-H, 4-Hc, 4-Hd, 5-Ha, 5-Hb), 1.06 (s, 9 H, 11-H), 1.05–0.97 (m, 13 H, 3-Hc, 3-Hd, 6-H, 13-H), 0.25 (s, 3 H, 9-Ha), 0.18 (s, 3 H, 9-Hb).

^{13}C-NMR (100 MHz, C_6D_6, δ in ppm): 147.3 (C-16), 84.3 (C-1), 80.0 (C-8), 75.6 (C-17), 43.9 (C-2), 41.2 (C-7), 37.7 (C-15), 30.7 (C-10), 29.4 (C-12), 28.6 (C-3a), 27.2 (C-3b), 26.9 (C-5),

26.7 (C-4a), 26.7 (C-4b), 22.3 (C-11), 16.7 (C-13), 15.2 (C-6b), 14.6 (C-6a), −2.7 (C-9a), −3.4 (C-9b). The signal of C-14 could not be detected.

HRMS (CI) *m/z*: $[M]^+$ calcd for $C_{30}H_{56}BIO_3Si$, 630.3137; found, 630.3096.

(4*R*,5*R*)-4,5-Dicyclohexyl-2-((*R*)-1-((4-methoxybenzyl)oxy)-3-phenylpropyl)-1,3,2-dioxa-borolane (16)

According to GP-1, 0.29 mL (206 mg, 2.04 mmol) *N*,*N*-diisopropylamine, 1.18 mL (1.89 mmol, 1.6 M) *n*-butyllithium and 0.30 mL anhydrous THF were used for the LDA solution. 514 mg (1.51 mmol) boronic ester **1**, 0.29 mL (384 mg, 4.53 mmol) anhydrous DCM, 2.11 mL anhydrous THF and a solution of 411 mg (3.02 mmol) zinc chloride in 1.81 mL anhydrous THF were used for homologation. The further reaction was performed according to variant C using 78.0 mg (1.96 mmol, 60%wt) NaH, 0.26 mL (292 mg, 2.11 mmol) 4-methoxybenzyl alcohol, 0.78 mL anhydrous THF and 2.16 mL anhydrous DMSO for the nucleophile solution. The reaction was worked up after 16 h. After column chromatography (SiO_2, CyH/EtOAc 0-7%), the product **16** (597 mg, 1.22 mmol, 81%) was obtained as a yellowish oil. R_f (**16**) = 0.23 (pentane/EtOAc 95:5). $[\alpha]_D^{20}$ = +76.2 (c = 1.0, $CHCl_3$).

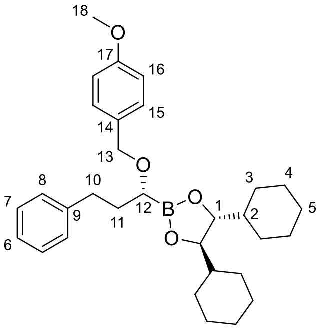

^{1}H-NMR (500 MHz, $CDCl_3$) δ: 7.31–7.24 (m, 4 H, 7-H, 15-H), 7.18–7.16 (m, 3 H, 6-H, 8-H), 6.88–6.85 (m, 2 H, 16-H), 4.55 (d, *J* = 11.4 Hz, 1 H, 13-Ha), 4.41 (d, *J* = 11.4 Hz, 1 H, 13-Hb), 3.92–3.91 (m, 2 H, 1-H), 3.81 (s, 3 H, 18-H), 3.35 (dd, *J* = 8.2 Hz, 5.5 Hz, 1 H, 12-H), 2.82–2.75 (m, 1 H, 10-Ha), 2.72–2.64 (m, 1 H, 10-Hb), 2.05–1.88 (m, 2 H, 11-H), 1.78–1.58 (m, 10 H, 3-Ha, 3-Hb, 4-Ha, 4-Hb, 5-Ha), 1.36–0.95 (m, 12 H, 2-H, 3-Hc, 3-Hd, 4-Hc, 4-Hd, 5-Hb).

^{13}C-NMR (125 MHz, $CDCl_3$) δ: 159.0 (C-17), 142.5 (C-9), 131.2 (C-15), 129.5 (C-14), 128.6 (C-8), 128.3 (C-7), 125.6 (C-6), 113.6 (C-16), 83.7 (C-1), 71.9 (C-13), 55.3 (C-18), 42.9 (C-2), 33.5 (C-10), 32.8 (C-11), 28.3 (C-3a), 27.4 (C-3b), 26.4 (C-5), 26.0 (C-4a), 35.9 (C-4b). The signal of C-12 could not be detected.

HRMS (ESI) *m/z*: $[M+H]^+$ calcd for $C_{31}H_{44}BO_4$, 491.3333; found, 491.3327.

((4*S*,5*S*)-4-((4*R*,5*R*)-4,5-Dicyclohexyl-1,3,2-dioxaborolan-2-yl)-5-((4-methoxybenzyl)oxy)-7-phenylhept-1-yn-1-yl)trimethylsilane (17)

Method A

According to GP-1, 0.43 mL (308 mg, 3.04 mmol) *N*,*N*-diisopropylamine, 1.76 mL (2.81 mmol, 1.6 M) *n*-butyllithium and 0.45 mL anhydrous THF were used for the LDA solution. 1.10 g (2.25 mmol) boronic ester **16**, 0.44 mL (574 mg, 6.75 mmol) anhydrous DCM, 3.15 mL anhydrous THF and a solution of 921 mg (6.75 mmol) zinc chloride in 4.05 mL anhydrous THF were used for homologation. The further reaction was performed according to

variant A using 14.5 mL (5.63 mmol, 0.39 M) of a solution of (3-(trimethylsilyl)prop-2-yn-1-yl)zinc(II) bromide lithium chloride in anhydrous THF. The nucleophile solution was prepared using 560 mg (13.2 mmol) LiCl, 1.57 g (24.0 mmol) zinc dust, 12.0 mL anhydrous THF, 21.0 µL (45.0 mg, 240 µmol) 1,2-dibromoethane, 77.0 µL (65.0 mg, 600 µmol) TMSCl, 1.96 mL (2.29 g, 12.0 mmol) (3-bromoprop-1-yn-1-yl)trimethylsilane and 12.0 mL anhydrous THF. The reaction was worked up after 64.5 h. After column chromatography (SiO_2, CyH/EtOAc 0-3%), the product **17** (749 mg, 1.22 mmol, 54%) was obtained as a colorless oil.

Method B

According to GP-1, the LDA solution was prepared using 193 µL (137 mg, 1.35 mmol, 1.35 eq.) *N*,*N*-diisopropylamine, 0.78 mL (1.25 mmol, 1.6 M, 1.25 eq.) *n*-butyllithium and 0.20 mL anhydrous THF. The freshly prepared LDA solution was added to a solution of 492 mg (1.00 mmol, 1.0 eq.) boronic ester **16** and 0.21 mL (523 mg, 3.01 mmol, 3.0 eq.) dibromomethane in 1.40 mL anhydrous THF at −78 °C. The reaction mixture was stirred for 1 h at −78 °C. Then, a solution of 410 mg (3.01 mmol, 3.0 eq.) zinc chloride in 1.81 mL anhydrous THF was added and the reaction mixture was slowly warmed to room temperature overnight. The reaction mixture was worked up according to GP-1 and the crude α-bromoboronic ester was used without further purification.

The crude α-bromoboronic ester was dissolved in 2.71 mL anhydrous THF and according to GP-1 variant A a solution of the protected propargylzinc reagent (2.92 mL, 1.10 mmol, 377 mM, 1.1 eq.) which was prepared using 223 mg (5.50 mmol) LiCl, 654 mg (10.0 mmol) zinc dust, 8.26 µL (19.0 mg, 0.10 mmol) 1,2-dibromoethane, 32.0 µL (27.0 mg, 0.25 mmol) TMSCl and 5.00 mL anhydrous THF as well as 0.82 mL (956 mg, 5.00 mmol) (3-bromoprop-1-yn-1-yl)trimethylsilane and 5.00 mL anhydrous THF was added. The reaction was worked up after 21 h. After column chromatography (SiO_2, CyH/EtOAc 0-3%), the product **17** (404 mg, 657 µmol, 66%) was obtained as a colorless oil. R_f (**17**) = 0.24 (pentane/EtOAc 97:3). $[\alpha]_D^{20}$ = +25.3 (c = 1.0, $CHCl_3$).

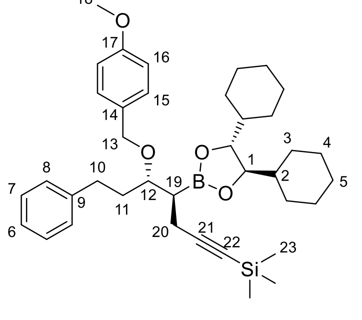

^{1}H-NMR (400 MHz, $CDCl_3$) δ: 7.28–7.24 (m, 4 H, 7-H, 15-H), 7.18–7.16 (m, 3 H, 6-H, 8-H), 6.87–6.85 (m, 2 H, 16-H), 4.50 (d, *J* = 11.3 Hz, 1 H, 13-Ha), 4.43 (d, *J* = 11.3 Hz, 1 H, 13-Hb), 3.85–3.83 (m, 2 H, 1-H), 3.80 (s, 3 H, 18-H), 3.70–3.66 (m, 1 H, 12-H), 2.76–2.69 (m, 1 H, 10-Ha), 2.66–2.58 (m, 1 H, 10-Hb), 2.49–2.38 (m, 2 H, 20-H), 2.03–1.94 (m, 1 H, 11-Ha), 1.92–1.84 (m, 1 H, 11-Hb), 1.76–1.57 (m, 10 H, 3-Ha, 3-Hb, 4-Ha, 4-Hb, 5-Ha), 1.33–0.93 (m, 13 H, 2-H, 3-Hc, 3-Hd, 4-Hc, 4-Hd, 5-Hb, 19-H), 0.12 (s, 9 H, 23-H).

^{13}C-NMR (100 MHz, $CDCl_3$) δ: 158.9 (C-17), 142.5 (C-9), 131.3 (C-15), 129.0 (C-14), 128.4 (C-8), 128.3 (C-7), 125.6 (C-6), 113.6 (C-16), 108.2 (C-22), 84.3 (C-21), 83.5 (C-1), 79.2 (C-12), 70.9 (C-13), 55.3 (C-18), 43.0 (C-2), 34.8 (C-11), 32.4 (C-10), 28.3 (C-3a), 27.4 (C-3b), 26.4 (C-5), 26.0 (C-4a), 25.9 (C-4b), 17.4 (C-20), 0.2 (C-23). The signal of C-19 could not be detected.

HRMS (CI) *m/z*: $[M]^+$ calcd for $C_{38}H_{55}BO_4Si$, 614.3963; found, 614.3985.

(4*R*,5*R*)-4,5-Dicyclohexyl-2-((4*S*,5*S*)-5-((4-methoxybenzyl)oxy)-7-phenylhept-1-yn-4-yl)-1,3,2-dioxaborolane (18)

To a solution of 201 mg (327 µmol, 1.0 eq.) boronic ester **17** in 4.08 mL methanol and 0.45 mL diethyl ether was added 90.0 mg (653 µmol, 2.0 eq.) K_2CO_3. The reaction mixture was stirred for 7 d. The reaction mixture was added to saturated NH_4Cl solution and diethyl ether. The phases were separated and the aqueous phase was extracted twice with diethyl ether. The combined organic phases were dried over Na_2SO_4, filtered and concentrated under reduced pressure. The residue was purified by column chromatography (SiO_2, CyH/EtOAc 0-5%). The product **18** (153 mg, 283 µmol, 87%) was obtained as a colorless oil. R_f (**18**) = 0.27 (pentane/EtOAc 97:3). $[\alpha]_D^{20}$ = +23.8 (c = 1.0, $CHCl_3$).

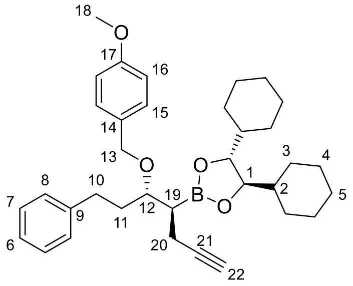

^{1}H-NMR (400 MHz, $CDCl_3$) δ: 7.28–7.24 (m, 4 H, 7-H, 15-H), 7.19–7.16 (m, 3 H, 6-H, 8-H), 6.87–6.85 (m, 2 H, 2 H, 16-H), 4.50 (d, *J* = 11.3 Hz, 1 H, 13-Ha), 4.43 (d, *J* = 11.1 Hz, 1 H, 13-Hb), 3.86–3.85 (m, 2 H, 1-H), 3.80 (s, 3 H, 18-H), 3.72–3.67 (m, 1 H, 12-H), 2.78–2.70 (m, 1 H, 10-Ha), 2.67–2.59 (m, 1 H, 10-Hb), 2.47–2.34 (m, 2 H, 20-H), 2.02–1.94 (m, 1 H, 11-Ha), 1.92 (t, *J* = 2.5 Hz, 22-H), 1.89 – 1.85 (m, 1 H, 11-Hb), 1.81–1.61 (m, 10 H, 3-Ha, 3-Hb, 4-Ha, 4-Hb, 5-Ha), 1.32–1.26 (m, 2 H, 2-H), 1.20–0.91 (m, 11 H, 3-Hc, 3-Hd, 4-Hc, 4-Hd, 5-Hb, 19-H).

^{13}C-NMR (100 MHz, $CDCl_3$) δ: 158.9 (C-17), 142.4 (C-9), 131.2 (C-15), 129.1 (C-14), 128.4 (C-8), 128.3 (C-7), 125.6 (C-6), 113.6 (C-16), 85.2 (C-1), 83.6 (C-12), 79.1 (C-21), 70.9 (C-13), 68.2 (C-22), 55.3 (C-18), 42.9 (C-2), 34.7 (C-10), 32.3 (C-11), 28.3 (C-3a), 27.5 (C-3b), 26.4 (C-5), 26.0 (C-4a), 25.9 (C-4b), 15.8 (C-20). The signal of C-19 could not be detected.

HRMS (CI) *m/z*: $[M]^+$ calcd for $C_{35}H_{47}BO_4$, 542.3567; found, 542.3570.

(3*S*,4*S*,*E*)-4-((4*R*,5*R*)-4,5-Dicyclohexyl-1,3,2-dioxaborolan-2-yl)-7-iodo-1-phenylhept-6-en-3-ol (19)

138 mg (254 µmol, 1.0 eq.) boronic ester **18** and 79.0 mg (305 µmol, 1.2 eq.) Schwartz reagent were suspended in 0.76 mL anhydrous DCM in the absence of light and stirred for 5 min at room temperature until the reaction mixture became transparent. 81.0 mg (317 µmol, 1.25 eq.) Iodine was added and the reaction mixture was stirred for 30 min at room temperature. The reaction mixture was added to saturated $Na_2S_2O_3$ solution. The phases were separated and the aqueous phase was extracted three times with pentane. The combined organic phases were washed with brine, dried over Na_2SO_4, filtered and concentrated under reduced pressure. The residue was purified by column chromatography (SiO_2, CyH/EtOAc 0-5%). The product **19** (117 mg, 213 µmol, 84%) was obtained as a colorless oil. R_f (**19**) = 0.17 (pentane/EtOAc 97:3). $[\alpha]_D^{20}$ = +17.9 (c = 1.0, $CHCl_3$).

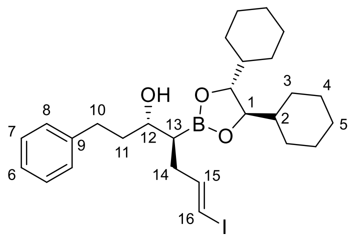

^{1}H-NMR (400 MHz, $CDCl_3$) δ: 7.30–7.28 (m, 2 H, 7-H), 7.19–7.16 (m, 3 H, 6-H, 8-H), 6.51 (dt, *J* = 14.2 Hz, 7.2 Hz, 1 H, 15-H), 6.03 (d, *J* = 14.3 Hz, 1 H, 16-H), 3.88–3.85 (m, 2 H, 1-H), 3.66–3.60 (m, 1 H, 12-H), 2.88–2.81 (m, 1 H, 10-Ha), 2.68–2.61 (m, 1 H, 10-Hb), 2.35–2.24 (m, 2 H, 14-H), 2.18 (d, *J* = 8.3 Hz, 1 H, OH), 1.79–1.61 (m, 12 H, 3-Ha, 3-Hb, 4-Ha, 4-Hb, 5-Ha, 11-H), 1.37–0.92 (m, 13 H, 2-H, 3-Hc, 3-Hd, 4-Hc, 4-Hd, 5-Hb, 13-H).

^{13}C-NMR (100 MHz, $CDCl_3$) δ: 145.6 (C-15), 142.3 (C-9), 128.4 (C-8), 128.4 (C-7), 125.7 (C-6), 83.8 (C-1), 75.6 (C-16), 72.3 (C-12), 42.9 (C-2), 39.6 (C-11), 35.2 (C-14), 32.4 (C-10), 28.6 (C-3a), 27.6 (C-3b), 26.4 (C-5), 25.9 (C-4a), 25.8 (C-4b). The signal of C-13 could not be detected.

HRMS (CI) *m/z*: $[M\text{-}H]^+$ calcd for $C_{27}H_{39}BIO_2$, 533.2088; found, 533.2067.

(4*S*,5*S*)-5-((4-Methoxybenzyl)oxy)-7-phenyl-1-(trimethylsilyl)hept-1-yn-4-ol (20)

According to GP-5, 510 mg (830 µmol) boronic ester **17** was subjected to oxidation using 1.66 mL anhydrous THF, 379 µL (141 mg, 4.15 mmol, 33%wt) aqueous H_2O_2 solution and a solution of 166 mg (4.15 mmol) NaOH in 1.66 mL water. The reaction was worked up after 1 h. To improve chromatographic separation, 59.6 mg (996 µmol) methylboronic acid and 4.15 mL diethyl ether were used. After column chromatography (SiO_2, CyH/EtOAc 0-20%), the product **20** (254 mg, 640 µmol, 77%) was obtained as a colorless oil. R_f (**20**) = 0.34 (pentane/EtOAc 97:3). $[\alpha]_D^{20}$ = +38.0 (c = 1.0, $CHCl_3$).

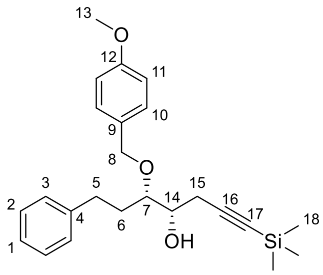

^{1}H-NMR (400 MHz, $CDCl_3$) δ: 7.31–7.18 (m, 7 H, 1-H, 2-H, 3-H, 10-H), 6.89–6.87 (m, 2 H, 11-H), 4.57 (d, *J* = 11.0 Hz, 1 H, 8-Ha), 4.47 (d, *J* = 11.0 Hz, 1 H, 8-Hb), 3.81 (s, 3 H, 13-H), 3.80–3.76 (m, 1 H, 14-H), 3.60–3.56 (m, 1 H, 7-H), 2.69 (t, *J* = 8.0 Hz, 2 H, 5-H), 2.50 (d, *J* = 6.5 Hz, 2 H, 15-H), 2.33 (d, *J* = 6.7 Hz, 1 H, OH), 2.05–1.86 (m, 2 H, 6-H), 0.14 (s, 9 H, 18-H).

^{13}C-NMR (100 MHz, $CDCl_3$) δ: 159.3 (C-12), 141.9 (C-4), 130.2 (C-10), 129.7 (C-9), 128.4 (C-3), 128.3 (C-2), 125.9 (C-1), 113.9 (C-11), 103.3 (C-17), 87.1 (C-16), 78.8 (C-7), 72.3 (C-8), 71.1 (C-14), 55.3 (C-13), 32.0 (C-5), 31.6 (C-6), 25.3 (C-15), 0.1 (C-18).

HRMS (CI) *m/z*: $[M{-}H]^+$ calcd for $C_{24}H_{31}O_3Si$, 395.2042; found, 395.2002.

***tert*-Butyl(((4*S*,5*S*)-5-((4-methoxybenzyl)oxy)-7-phenylhept-1-yn-4-yl)oxy)dimethylsilane (21)**

To a solution of 240 mg (606 µmol, 1.0 eq.) alcohol **20** in 4.66 mL anhydrous THF were added 606 µL (606 µmol, 1.0 M) of a solution of TBAF in THF at 0 °C. The reaction mixture was warmed to room temperature and stirred for 1 h. The reaction mixture was concentrated under reduced pressure. The residue was purified by column chromatography (SiO_2, CyH/EtOAc 0-30%). The product **S2** (191 mg, 589 µmol, 97%) was obtained as a yellowish oil. R_f (**S2**) = 0.26 (pentane/EtOAc 8:2). $[\alpha]_D^{20}$ = +30.2 (c = 1.0, $CHCl_3$).

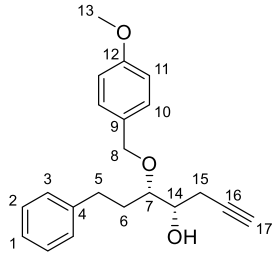

^{1}H-NMR (400 MHz, $CDCl_3$, δ in ppm): 7.31–7.18 (m, 7 H, 1-H, 2-H, 3-H, 10-H), 6.89–6.87 (m, 2 H, 11-H), 4.59 (d, *J* = 11.0 Hz, 1 H, 8-Ha), 4.47 (d, *J* = 10.9 Hz, 1 H, 8-Hb), 3.84–3.78 (m, 4 H, 13-H, 14-H), 3.60–3.56 (m, 1 H, 7-H), 2.71 (t, *J* = 8.1 Hz, 2 H, 5-H), 2.46 (dd, *J* = 6.1 Hz, 2.0 Hz, 2 H, 15-H), 2.38 (d, *J* = 6.6 Hz, 1 H, OH), 2.03 (t, *J* = 2.6 Hz, 1 H, 17-H), 2.00–1.87 (m, 2 H, 6-H).

^{13}C-NMR (100 MHz, $CDCl_3$, δ in ppm): 159.4 (C-12), 141.8 (C-4), 130.2 (C-10), 129.6 (C-9), 128.4 (C-3), 128.4 (C-2), 125.9 (C-1), 113.9 (C-11), 80.8 (C-16), 79.1 (C-7), 72.3 (C-8), 71.0 (C-14), 70.5 (C-17), 55.3 (C-13), 32.0 (C-6), 31.5 (C-5), 23.8 (C-15).

HRMS (CI) *m/z*: $[M]^+$ calcd for $C_{21}H_{24}O_3$, 324.1725; found, 324.1729.

177 mg (545 µmol, 1.0 eq.) alcohol **S2** and 215 mg (3.16 mmol, 5.8 eq.) imidazole were dissolved in 2.95 mL anhydrous DCM. 304 mg (2.02 mmol, 3.7 eq.) TBSCl were added and the reaction mixture was stirred at room temperature for 22 h.

Saturated $NaHCO_3$ solution is added and the phases were separated. The aqueous phase was extracted with DCM. The organic phase was dried over $MgSO_4$, filtered and concentrated under reduced pressure. The residue was purified by column chromatography (SiO_2, CyH/EtOAc 0-10%). The product **21** (215 mg, 489 µmol, 90%) was obtained as a colorless oil. R_f (**21**) = 0.44 (pentane/EtOAc 9:1). $[\alpha]_D^{20}$ = −46.6 (c = 1.0, $CHCl_3$).

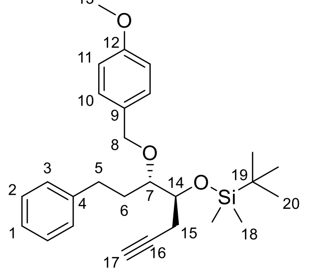

^{1}H-NMR (400 MHz, $CDCl_3$, δ in ppm): 7.29–7.24 (m, 4 H, 2-H, 10-H), 7.18–7.11 (m, 3 H, 1-H, 3-H), 6.91–6.88 (m, 2 H, 11-H), 4.58 (d, *J* = 11.2 Hz, 1 H, 8-Ha), 4.45 (d, *J* = 11.2 Hz, 1 H, 8-Hb), 3.98 (m, 1 H, 14-H), 3.82 (s, 3 H, 13-H), 3.38 (m, 1 H, 7-H), 2.83–2.76 (m, 1 H, 5-Ha), 2.58–2.51 (m, 2 H, 5-Hb, 15-Ha), 2.23 (ddd, *J* = 16.8 Hz, 8.3 Hz, 2.6 Hz, 1 H, 15-Hb), 1.98–

1.90 (m, 2 H, 6-Ha, 17-H), 1.71–1.62 (m, 1 H, 6-Hb), 0.85 (s, 9 H, 20-H), 0.08 (s, 3 H, 18-Ha), −0.04 (s, 3 H, 18-Hb).

^{13}C-NMR (100 MHz, $CDCl_3$, δ in ppm): 159.3 (C-12), 142.1 (C-4), 130.7 (C-9), 129.5 (C-10), 128.5 (C-3), 128.3 (C-2), 125.7 (C-1), 113.8 (C-11), 82.9 (C-16), 79.9 (C-7), 72.0 (C-8), 71.2 (C-14), 69.4 (C-17), 55.3 (C-13), 32.3 (C-5), 30.3 (C-6), 25.8 (C-20), 21.9 (C-15), 18.0 (C-19), −4.5 (C-18a), −4.8 (C-18b).

HRMS (CI) *m/z*: $[M]^+$ calcd for $C_{27}H_{38}O_3Si$, 438.2590; found, 438.2598.

(3*S*,4*S*,*E*)-4-((*tert*-Butyldimethylsilyl)oxy)-7-iodo-1-phenylhept-6-en-3-ol (22)

199 mg (453 µmol, 1.0 eq.) alkyne **21** and 140 mg (543 µmol, 1.2 eq.) Schwartz reagent were suspended in 1.36 mL anhydrous DCM in the absence of light. The reaction mixture was stirred for 5 min at room temperature. Then, 144 mg (566 µmol, 1.25 eq.) iodine were added and the reaction mixture was stirred for additional 30 min at room temperature.

Saturated $Na_2S_2O_3$ solution was added and the phases were separated. The aqueous phase was extracted three times with diethyl ether. The combined organic phases were washed with brine, dried over $MgSO_4$ and filtered. The solvent was removed under reduced pressure. The residue was purified by column chromatography (SiO_2, CyH/EtOAc 0-10%). The product **22** (159 mg, 351 µmol, 78%) was obtained as a colorless oil. R_f (**22**) = 0.41 (pentane/EtOAc 9:1). $[\alpha]_D^{20}$ = −19.4 (c = 1.0, $CHCl_3$).

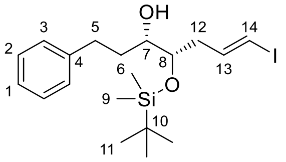

^{1}H-NMR (400 MHz, $CDCl_3$, δ in ppm): 7.31–7.28 (m, 2 H, 2-H), 7.20–7.18 (m, 3 H, 1-H, 3-H), 6.45 (dt, J = 14.4 Hz, 7.5 Hz, 1 H, 13-H), 6.05 (d, J = 14.4 Hz, 1 H, 14-H), 3.59–3.55 (m, 1 H, 8-H), 3.46–3.40 (m, 1 H, 7-H), 2.88–2.81 (m, 1 H, 5-Ha), 2.71–2.63 (m, 1 H, 5-Hb), 2.41–2.34 (m, 1 H, 12-Ha), 2.22–2.15 (m, 1 H, 12-Hb), 2.07 (d, J = 7.0 Hz, 1 H, OH), 1.75–1.69 (m, 2 H, 6-H), 0.90 (s, 9 H, 11-H), 0.09 (s, 3 H, 9-Ha), 0.06 (s, 3 H, 9-Hb).

^{13}C-NMR (100 MHz, $CDCl_3$, δ in ppm): 142.1 (C-4), 141.9 (C-13), 128.4 (C-2, C-3), 125.9 (C-1), 77.5 (C-14), 73.8 (C-8), 72.1 (C-7), 40.5 (C-12), 35.4 (C-6), 32.1 (C-5), 25.8 (C-11), 18.0 (C-10), −4.1 (C-9a), −4.6 (C-9b).

HRMS (ESI) *m/z*: $[M+H]^+$ calcd for $C_{19}H_{32}IO_2Si$, 447.1211; found, 447.1191.

(5*S*,6*S*)-5-((*E*)-3-Iodoallyl)-2,2,3,3,8,8,9,9-octamethyl-6-phenethyl-4,7-dioxa-3,8-disila-decane (23)

54.4 mg (122 µmol, 1.0 eq.) alcohol **22** and 48.1 mg (707 µmol, 5.8 eq.) imidazole were diluted in 0.66 mL anhydrous DCM. 68.0 mg (451 µmol, 3.7 eq.) TBSCl was added and the reaction mixture was stirred for 20 h at room temperature.

Saturated $NaHCO_3$ solution was added and the phases were separated. The aqueous phase was extracted with DCM, dried over $MgSO_4$ and filtered. The solvent was removed under reduced pressure and the residue was purified by column chromatography (SiO_2, CyH/EtOAc 0-3%). The product **23** (47.7 mg, 85.0 µmol, 70%) was obtained as a colorless oil. R_f (**23**) = 0.63 (pentane/EtOAc 98:2). $[\alpha]_D^{20}$ = −19.4 (c = 1.0, $CHCl_3$).

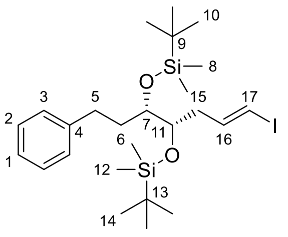

^{1}H-NMR (400 MHz, $CDCl_3$, δ in ppm): 7.30–7.28 (m, 2 H, 2-H), 7.19–7.17 (m, 3 H, 1-H, 3-H), 6.50–6.43 (m, 1 H, 16-H), 5.98 (d, J = 14.4 Hz, 1 H, 17-H), 3.61–3.69 (m, 2 H, 7-H, 11-H), 2.84–2.77 (m, 1 H, 5-Ha), 2.52–2.38 (m, 2 H, 5-Hb, 15-Ha), 2.06–1.95 (m, 2 H, 6-Ha, 15-Hb), 1.59–1.52 (m, 1 H, 6-Hb), 0.92 (s, 9 H, 14-H), 0.86 (s, 9 H, 10-H), 0.10 (s, 3 H, 12-Ha), 0.06 (s, 3 H, 12-Hb), 0.03 (s, 3 H, 8-Ha), −0.03 (s, 3 H, 8-Hb).

^{13}C-NMR (100 MHz, $CDCl_3$, δ in ppm): 144.8 (C-16), 142.5 (C-4), 128.4 (C-3), 128.3 (C-2), 125.7 (C-1), 76.2 (C-17), 74.6 (C-7), 74.3 (C-11), 37.3 (C-15), 33.0 (C-6), 32.1 (C-5), 25.9 (C-14), 25.7 (C-10), 18.0 (C-13), 17.9 (C-9), −4.0 (C-12a), −4.5 (C-8, C-12b).

HRMS (CI) *m/z*: $[M+H]^+$ calcd for $C_{25}H_{46}IO_2Si_2$, 561.2081; found, 561.2053.

(5*S*,6*S*,8*E*,10*E*,13*R*,14*R*,15*S*)-6-((*tert*-Butyldimethylsilyl)oxy)-13-((4*R*,5*R*)-4,5-dicyclohexyl-1,3,2-dioxaborolan-2-yl)-15-isopropyl-2,2,3,3,14,17,17,18,18-nonamethyl-5-phenethyl-4,16-dioxa-3,17-disilanonadeca-8,10-diene (24)

To a solution of 133 mg (237 µmol, 3.0 eq.) vinyliodide **23** in 1.42 mL anhydrous Et_2O was added dropwise 0.25 mL (474 µmol, 6.0 eq., 1.9 M in pentane) *t*BuLi at −100 °C. The reaction mixture was stirred for 1 h at the same temperature. Then, a solution of 32.3 mg (237 µmol, 3.0 eq.) $ZnCl_2$ in 0.41 mL anhydrous THF was added and the reaction mixture was warmed to room temperature. According to GP-4, 49.8 mg (79.0 µmol) boronic ester **15**, 9.1 mg (7.89 µmol) $Pd(PPh_3)_4$, 1.58 mL anhydrous THF and 1.04 mL of the arylzinc reagent solution were used for the Negishi coupling. The reaction mixture was worked up after 2 h. After column chromatography (SiO_2, CyH/EtOAc 0-3%), the product **24** (55.3 mg, 59.0 µmol, 75%) was obtained as a colorless oil. R_f (**24**) = 0.28 (pentane/EtOAc 99:1). $[\alpha]_D^{20} = -11.4$ (c = 1.0, $CHCl_3$).

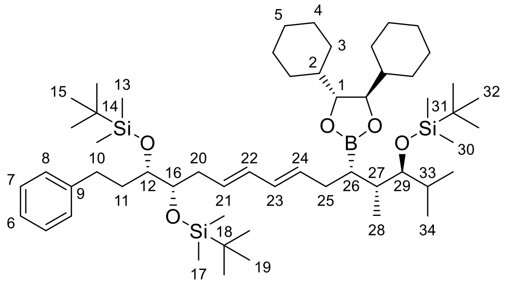

^{1}H-NMR (400 MHz, $CDCl_3$, δ in ppm): 7.29–7.26 (m, 2 H, 7-H), 7.20–7.15 (m, 3 H, 6-H, 8-H), 6.01–5.91 (m, 2 H, 22-H, 23-H), 5.56–5.47 (m, 2 H, 21-H, 24-H), 3.78–3.76 (m, 2 H, 1-H), 3.61–3.52 (m, 3 H, 12-H, 16-H, 29-H), 2.84–2.77 (m, 1 H, 10-Ha), 2.53–2.45 (m, 1 H, 10-Hb), 2.42–2.37 (m, 1 H, 20-Ha), 2.29–2.21 (m, 1 H, 25-Ha), 2.16–2.09 (m, 1 H, 25-Hb), 2.04–1.96 (m, 2 H, 11-Ha, 20-Hb), 1.82–1.56 (m, 13 H, 3-Ha, 3-Hb, 4-Ha, 4-Hb, 5-Ha, 11-Hb, 27-H, 33-H), 1.42–0.97 (m, 16 H, 2-H, 3-Hc, 3-Hd, 4-Hc, 4-Hd, 5-Hb, 26-H, 28-H), 0.93 (s, 9 H, 19-H), 0.90 (s, 9 H, 32-H), 0.85 (s, 9 H, 15-H), 0.83–0.81 (m, 6 H, 34-H), 0.09 (s, 3 H, 17-Ha), 0.07 (s, 3 H, 17-Hb), 0.06 (s, 3 H, 30-Ha), 0.04 (s, 3 H, 30-Hb), −0.02 (s, 3 H, 13-Ha), −0.04 (s, 3 H, 13-Hb).

^{13}**C-NMR** (100 MHz, $CDCl_3$, δ in ppm): 142.7 (C-9), 132.4 (C-22), 132.4 (C-23), 130.7 (C-24), 129.7 (C-21), 128.4 (C-8), 128.3 (C-7), 125.6 (C-6), 83.5 (C-1), 78.9 (C-29), 75.6 (C-16), 74.9 (C-12), 43.1 (C-2), 40.2 (C-33), 33.7 (C-20), 33.2 (C-10, C-25), 32.2 (C-11), 29.6 (C-27), 28.7 (C-3a), 27.9 (C-3b), 26.5 (C-5), 26.2 (C-32), 26.0 (C-4a), 25.9 (C-4b, C-19), 25.8 (C-15), 22.0 (C-28), 18.5 (C-31), 18.1 (C-18), 18.0 (C-14), 16.3 (C-34a), 14.3 (C-34b), −3.5 (C-17a), −4.0 (C-17b), −4.1 (C-13a), −4.4 (C-13b), −4.5 (C-30a), −4.5 (C-30b). The signal of C-26 could not be detected.

HRMS (ESI) *m/z*: $[M+H]^+$ calcd for $C_{55}H_{102}BO_5Si_3$, 937.7123; found, 937.7094.

Copies of NMR spectra

((*R*)-4-((4*R*,5*R*)-4,5-Dicyclohexyl-1,3,2-dioxaborolan-2-yl)-6-phenylhex-1-yn-1-yl)trimethylsilane (2)

^{1}H-NMR (400 MHz, $CDCl_3$):

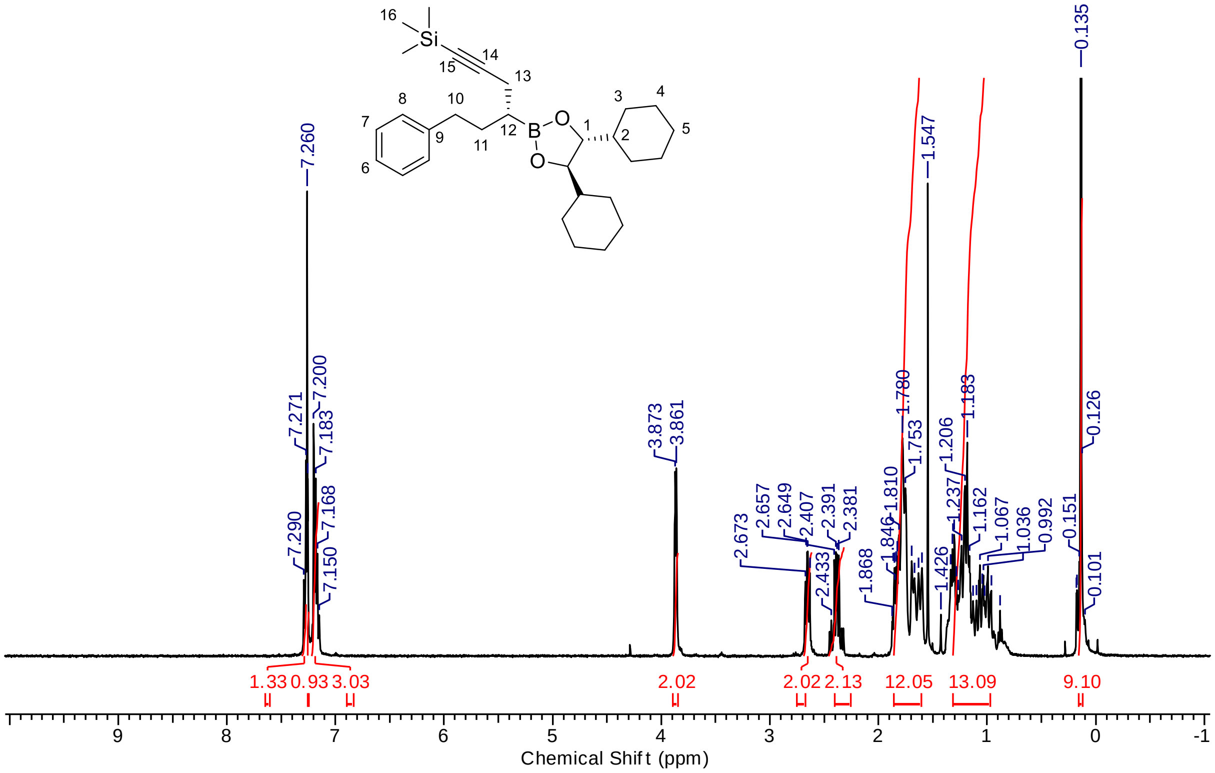

^{13}C-NMR (100 MHz, $CDCl_3$):

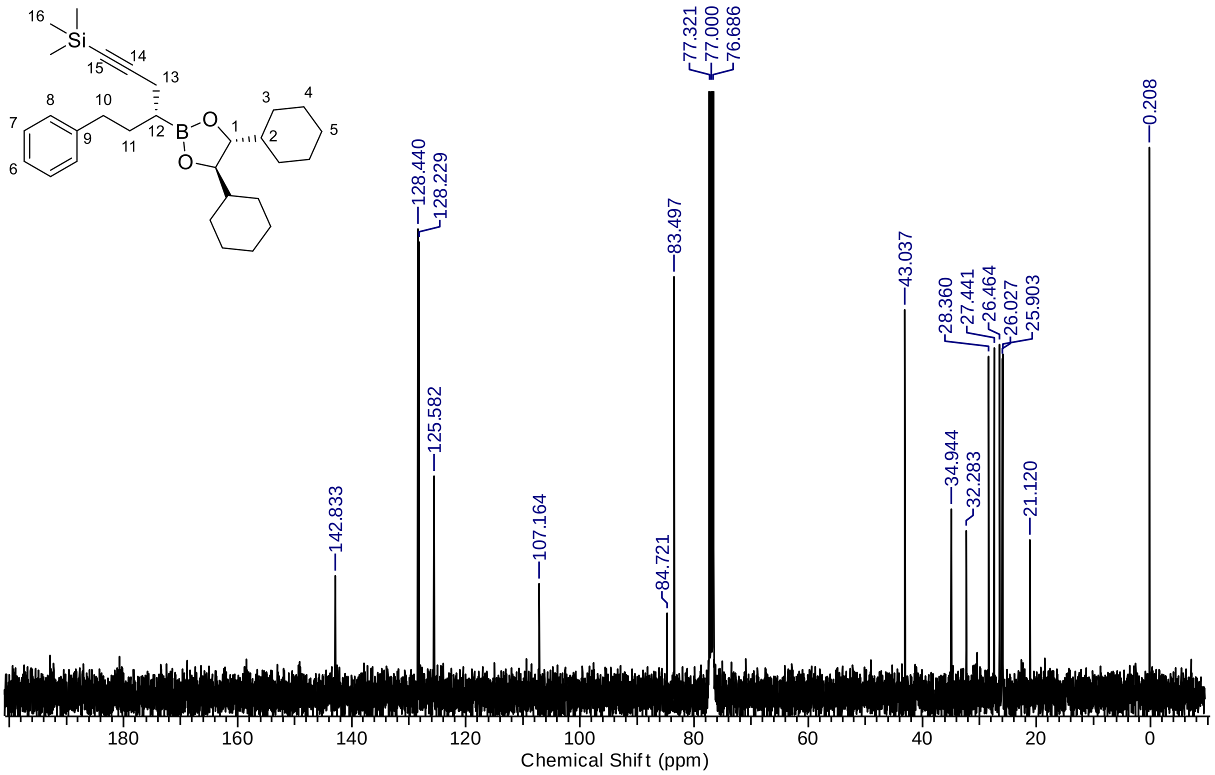

^{1}H,^{1}H-COSY

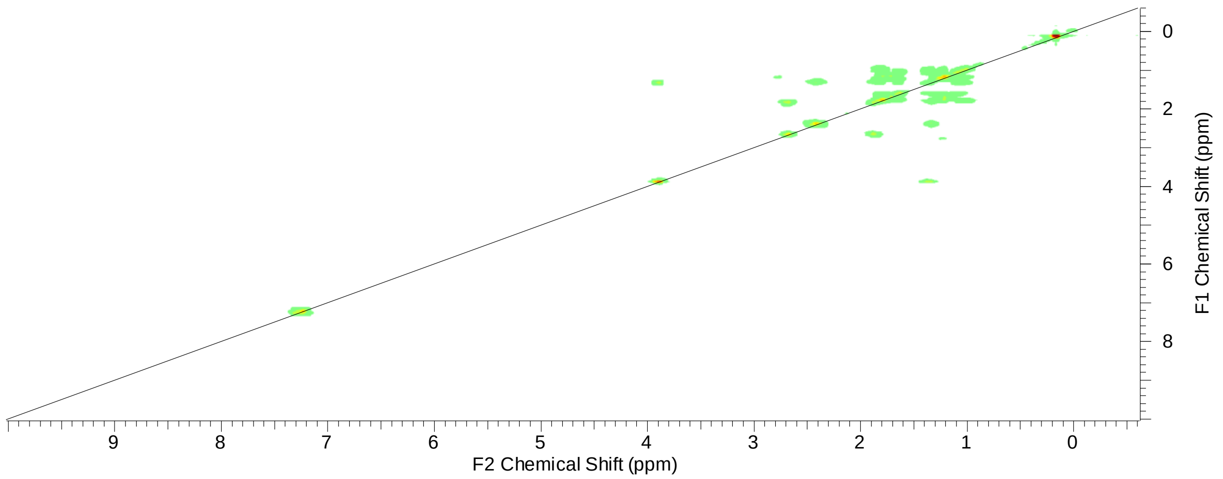

^{1}H,^{13}C-HSQC

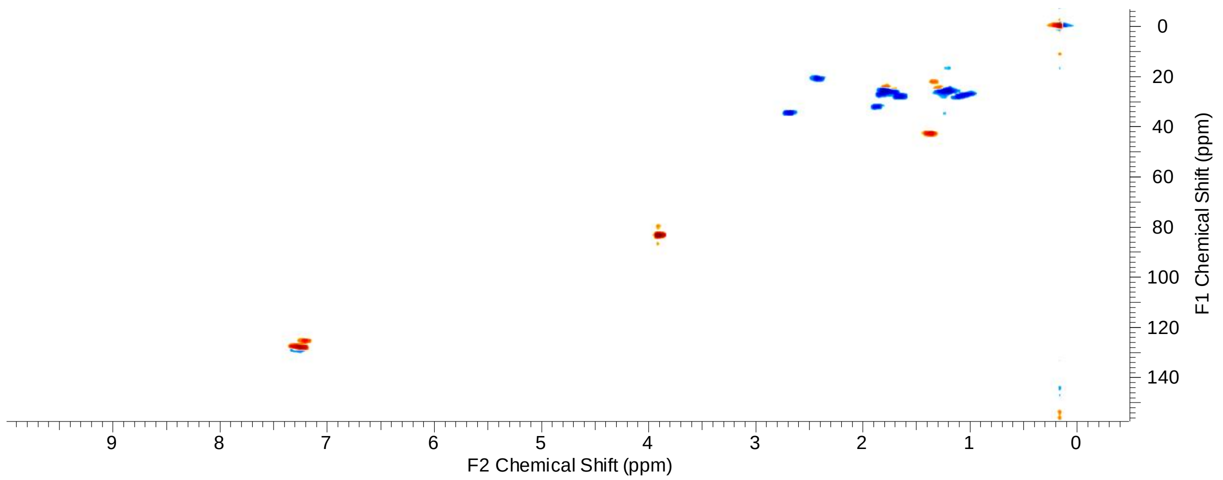

^{1}H,^{13}C-HMBC

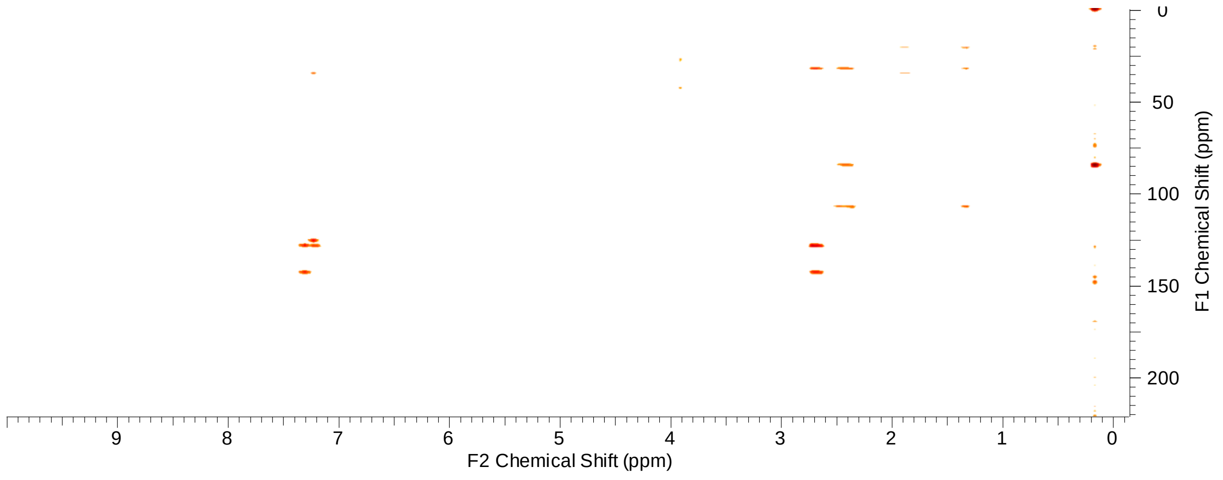

(4*R*,5*R*)-4,5-Dicyclohexyl-2-((*R*)-1-phenylhex-5-yn-3-yl)-1,3,2-dioxaborolane (3)

^{1}H-NMR (400 MHz, $CDCl_3$):

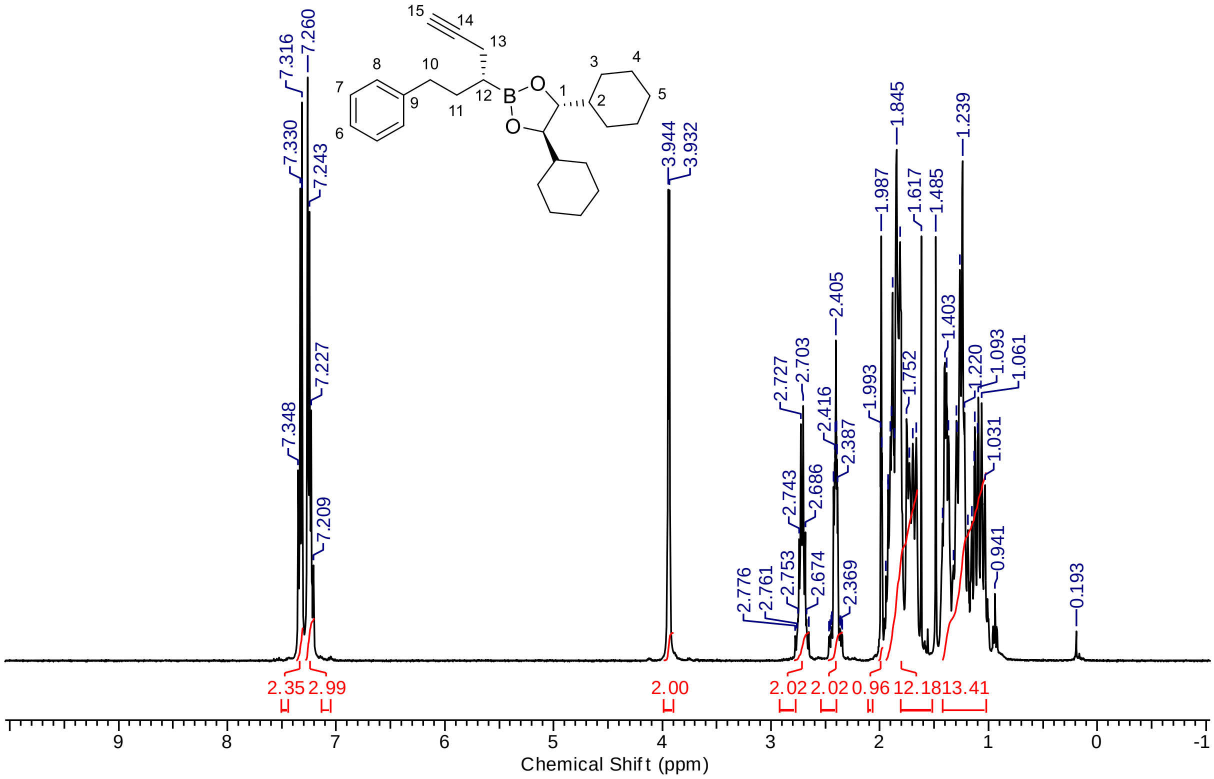

^{13}C-NMR (100 MHz, $CDCl_3$):

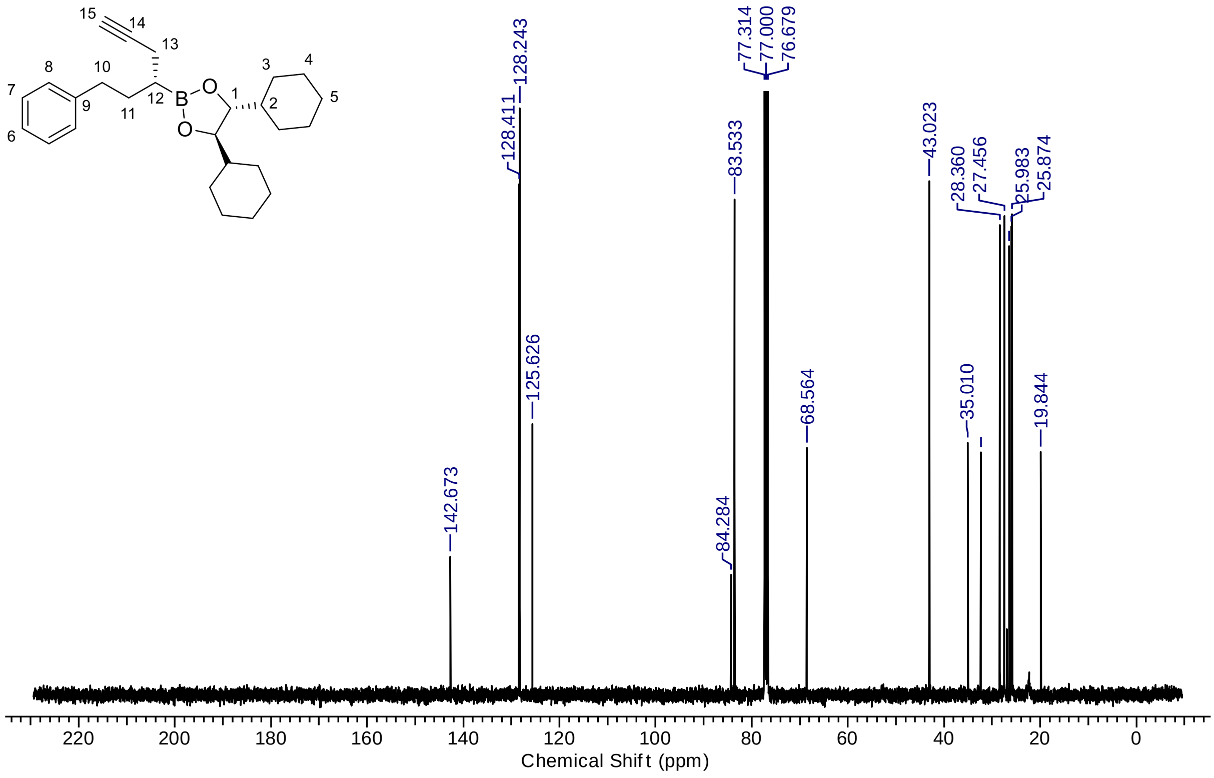

$^{1}H,^{1}H$-COSY

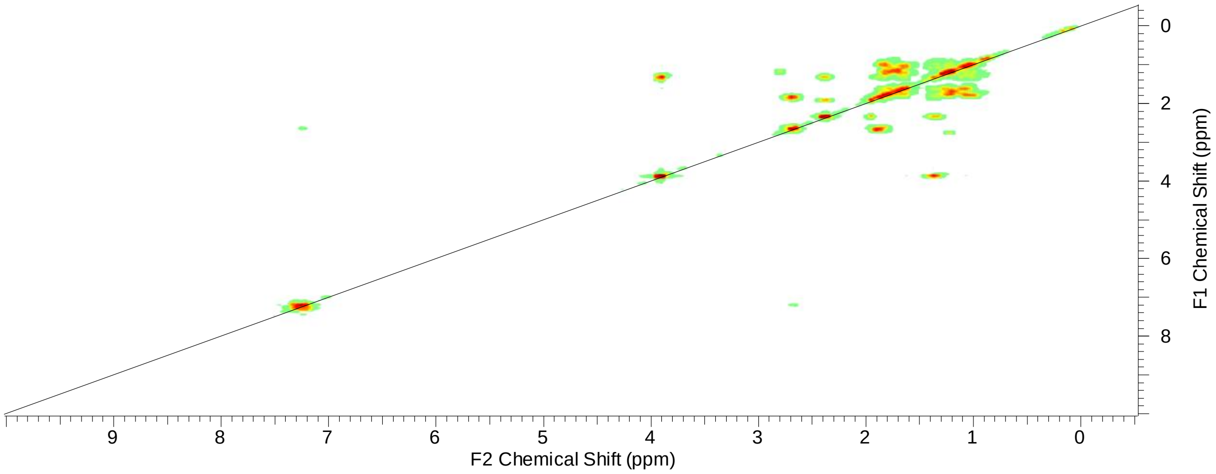

$^{1}H,^{13}C$-HSQC

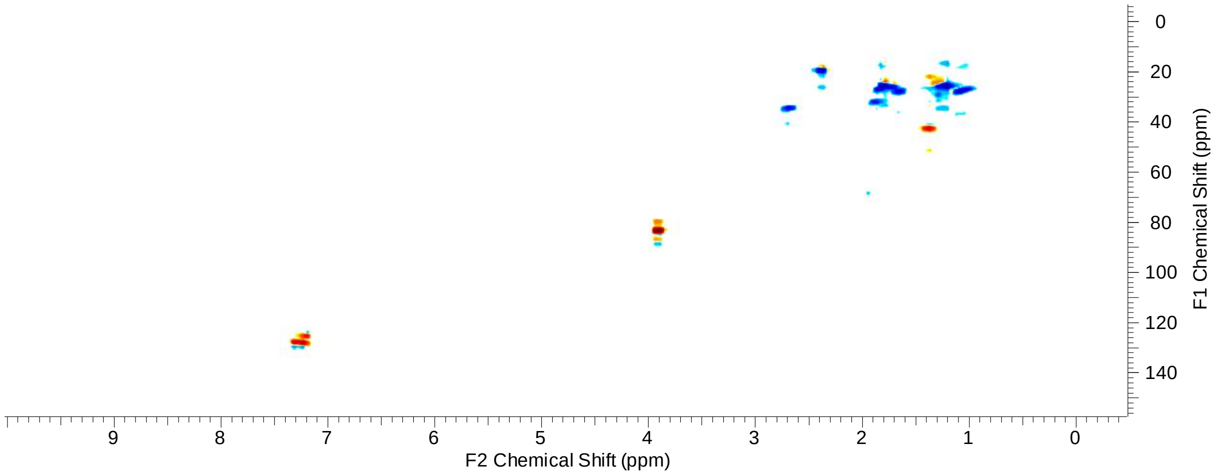

$^{1}H,^{13}C$-HMBC

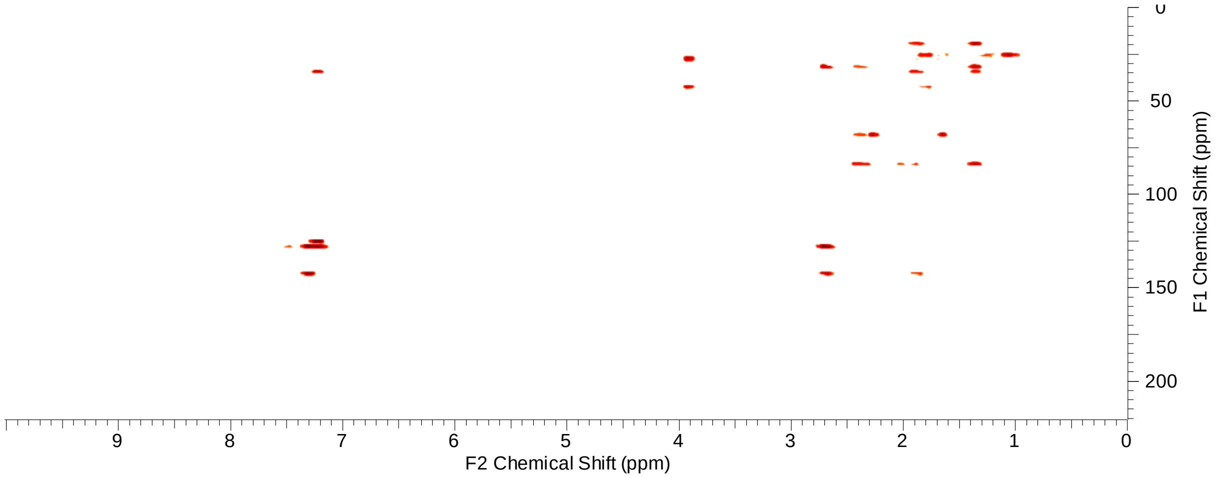

((*R*,*E*)-4-((4*R*,5*R*)-4,5-Dicyclohexyl-1,3,2-dioxaborolan-2-yl)-1-iodo-6-phenylhex-1-en-1-yl)trimethylsilane (4a)

^{1}H-NMR (400 MHz, $CDCl_3$):

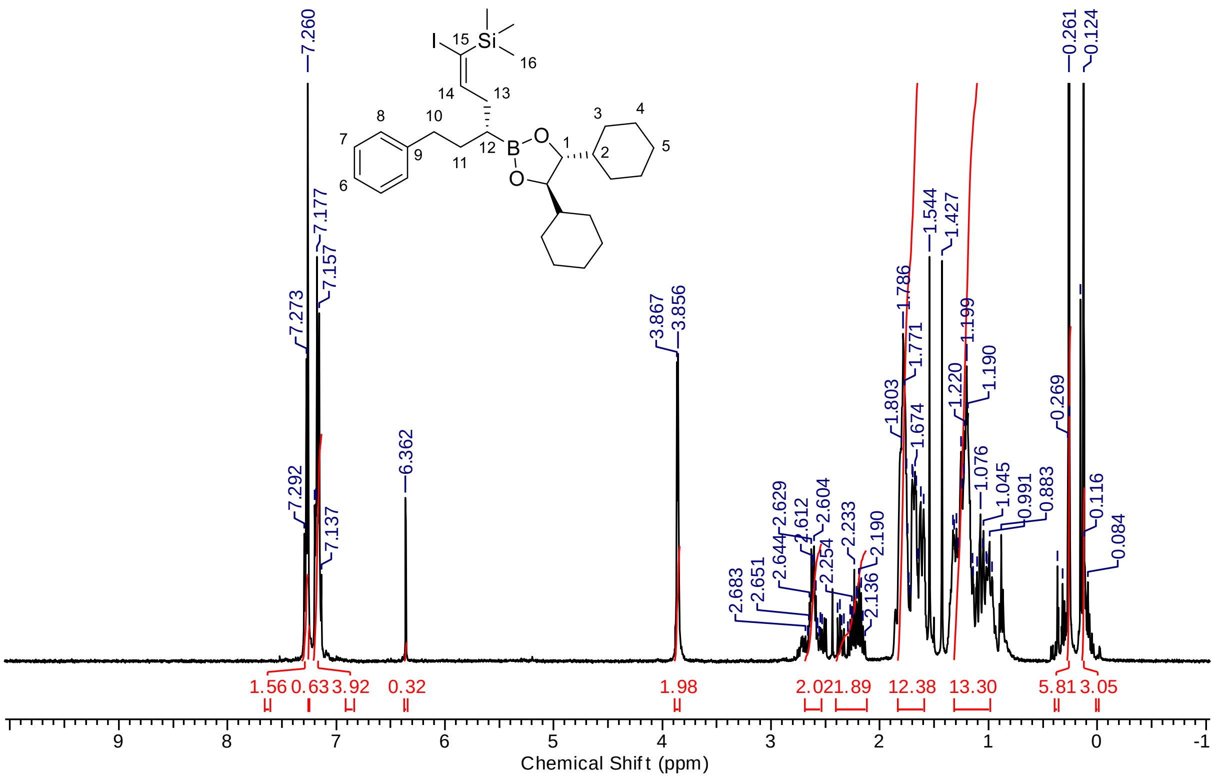

^{13}C-NMR (100 MHz, $CDCl_3$):

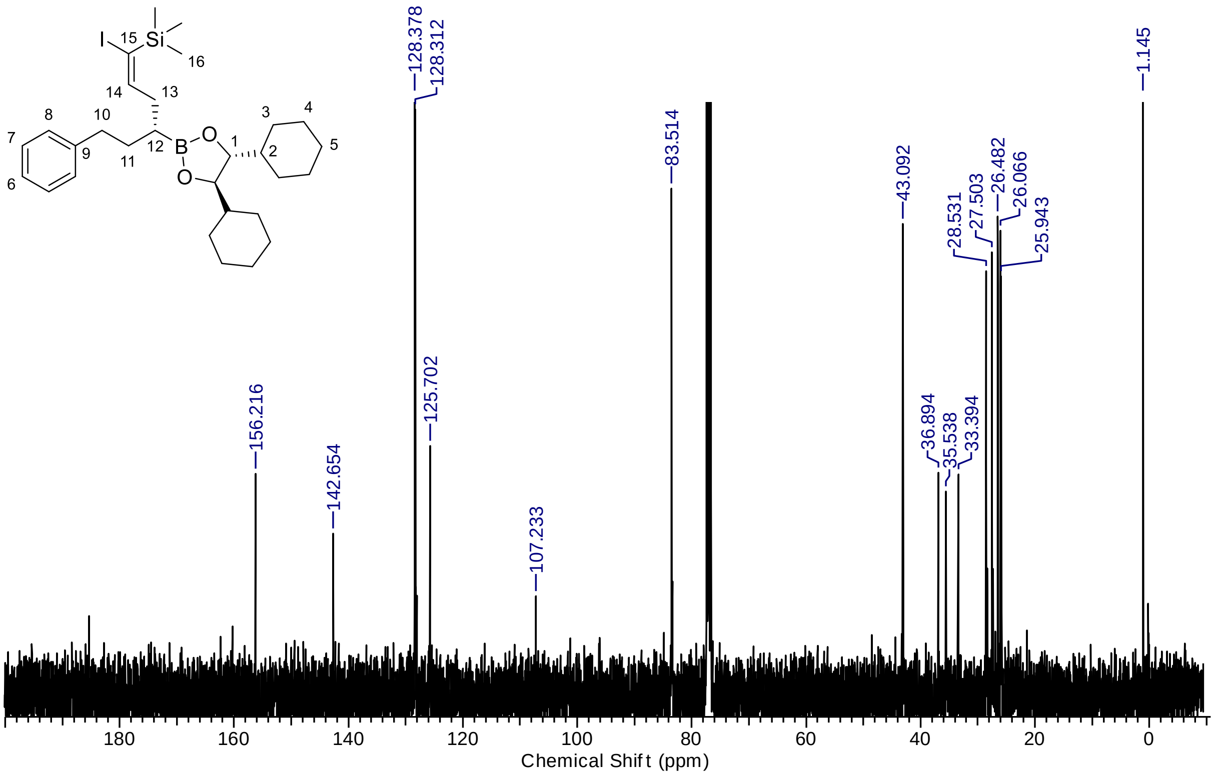

^{1}H,^{1}H-COSY

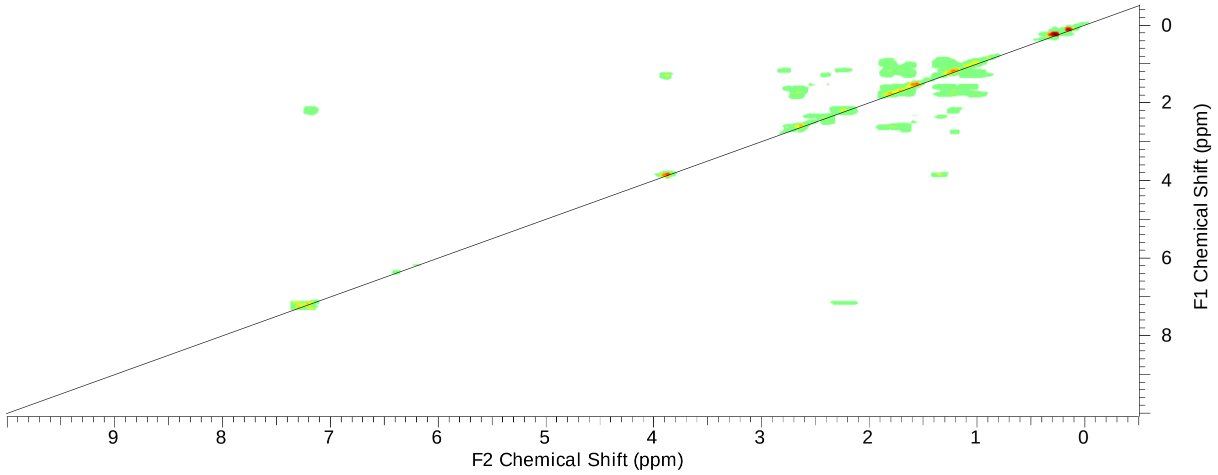

^{1}H,^{13}C-HSQC

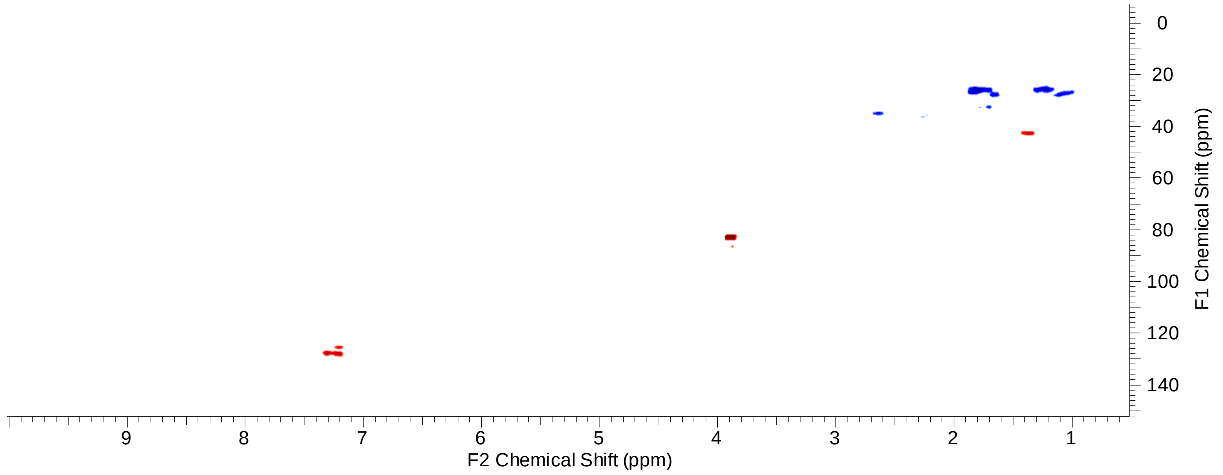

^{1}H,^{13}C-HMBC

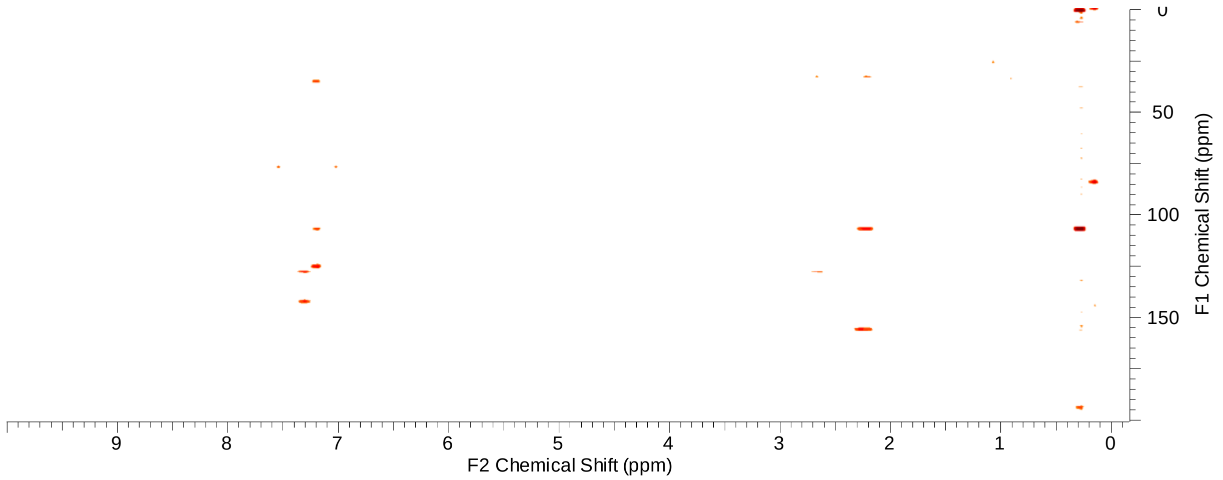

((*R*,*Z*)-4-((4*R*,5*R*)-4,5-Dicyclohexyl-1,3,2-dioxaborolan-2-yl)-6-phenylhex-1-en-1-yl)-trimethylsilane (4b)

^{1}H-NMR (400 MHz, $CDCl_3$):

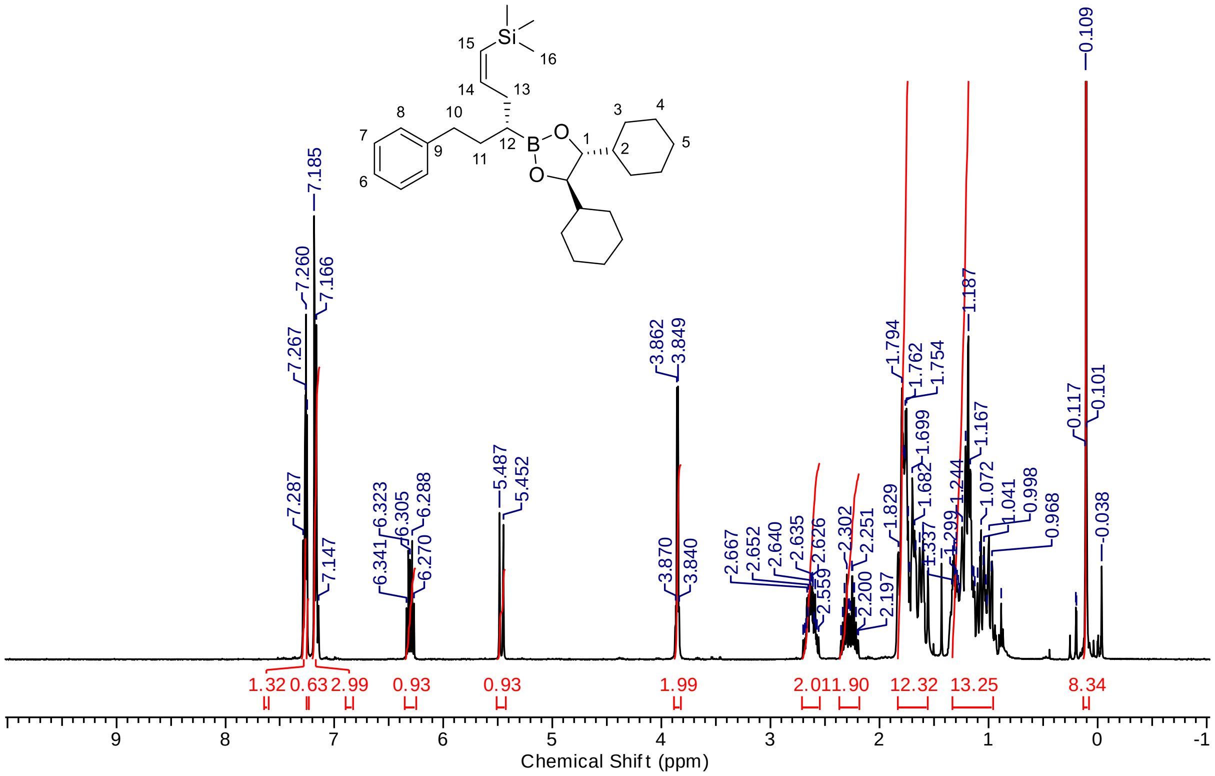

^{13}C-NMR (100 MHz, $CDCl_3$):

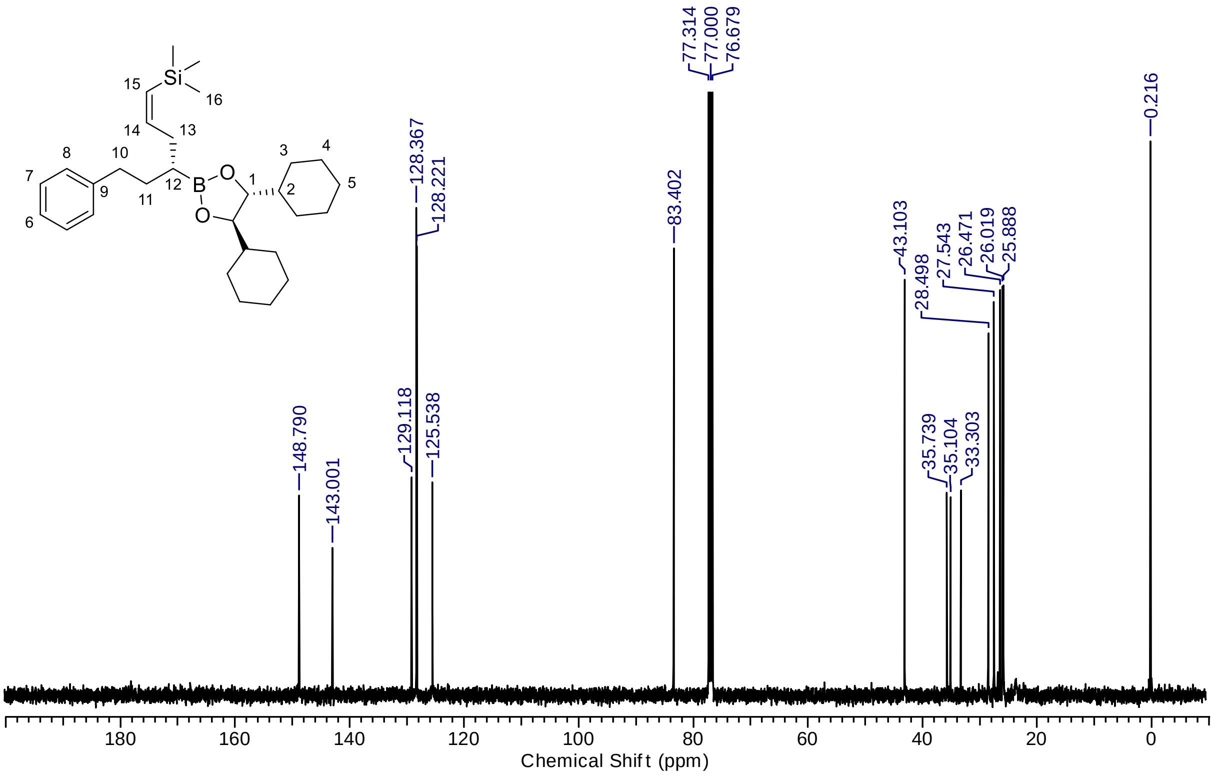

$^{1}H,^{1}H$-COSY

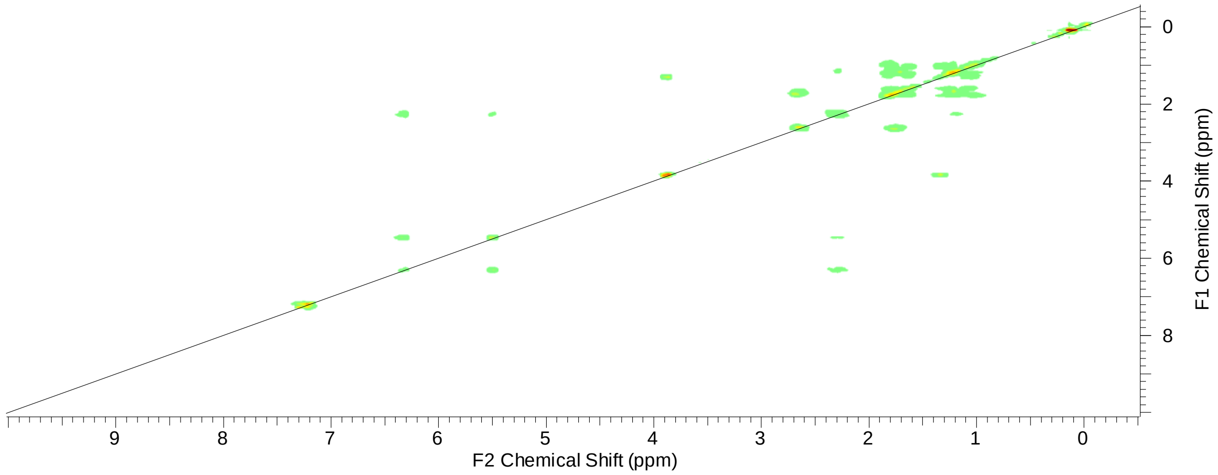

$^{1}H,^{13}C$-HSQC

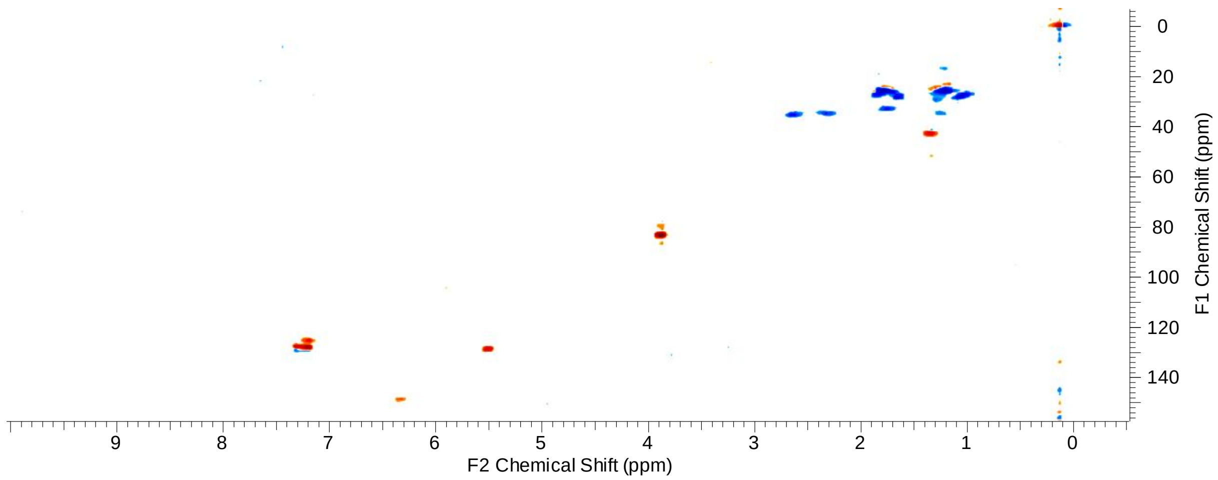

$^{1}H,^{13}C$-HMBC

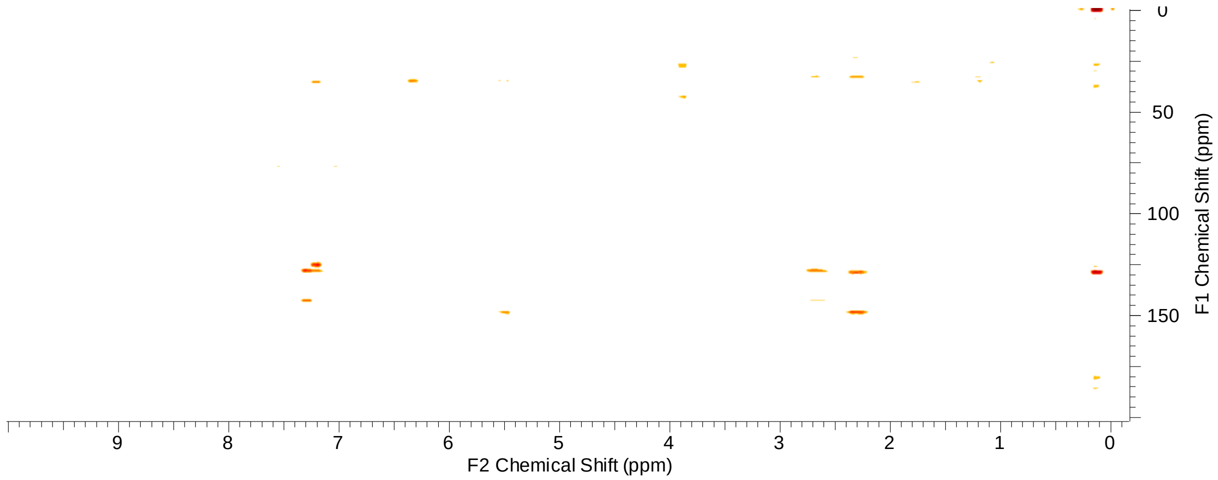

(4*R*,5*R*)-2-((*R*,*E*)-6-Chloro-1-phenylhex-5-en-3-yl)-4,5-dicyclohexyl-1,3,2-dioxaborolane (5a)

^{1}H-NMR (400 MHz, $CDCl_3$):

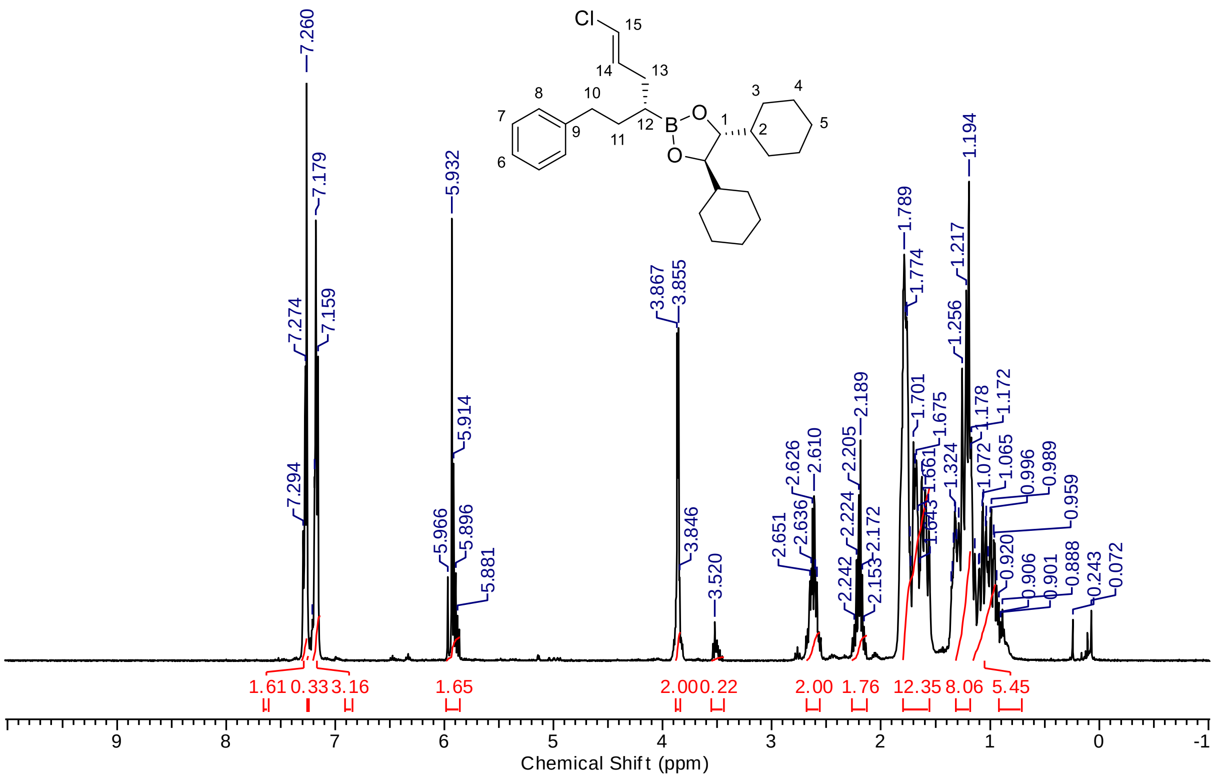

^{13}C-NMR (100 MHz, $CDCl_3$):

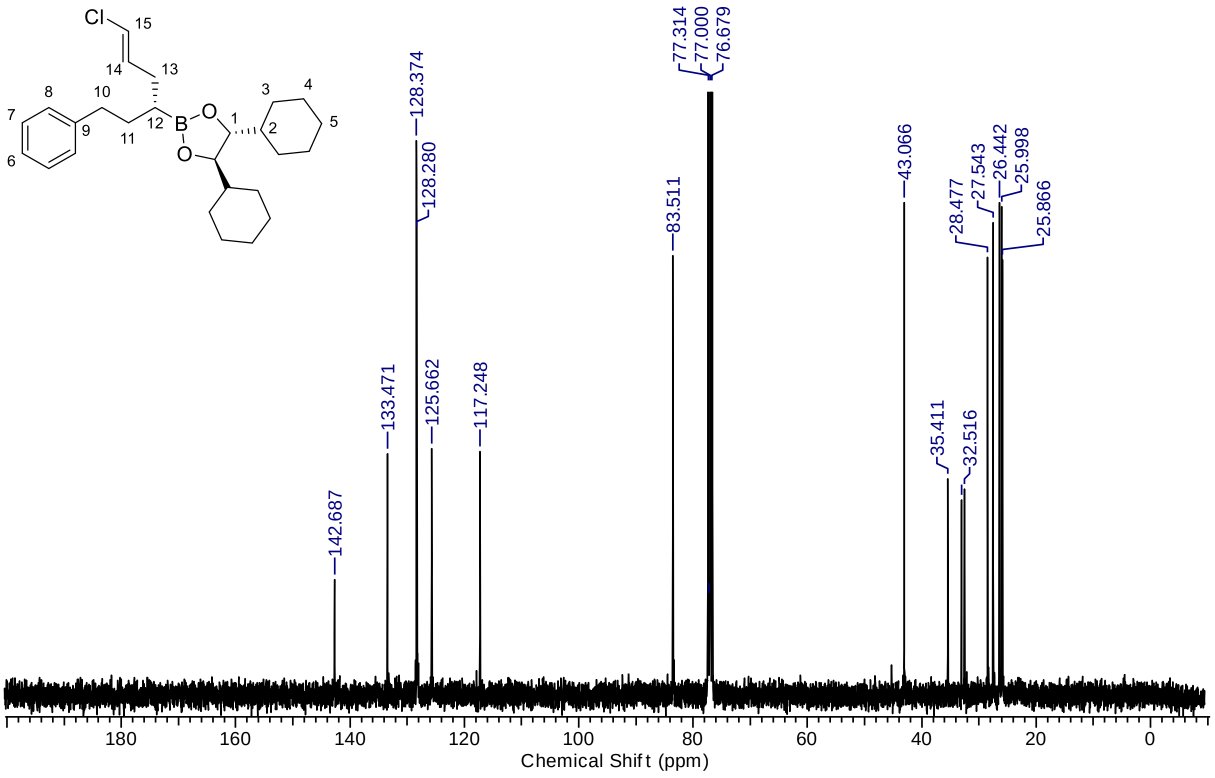

$^{1}H,^{1}H$-COSY

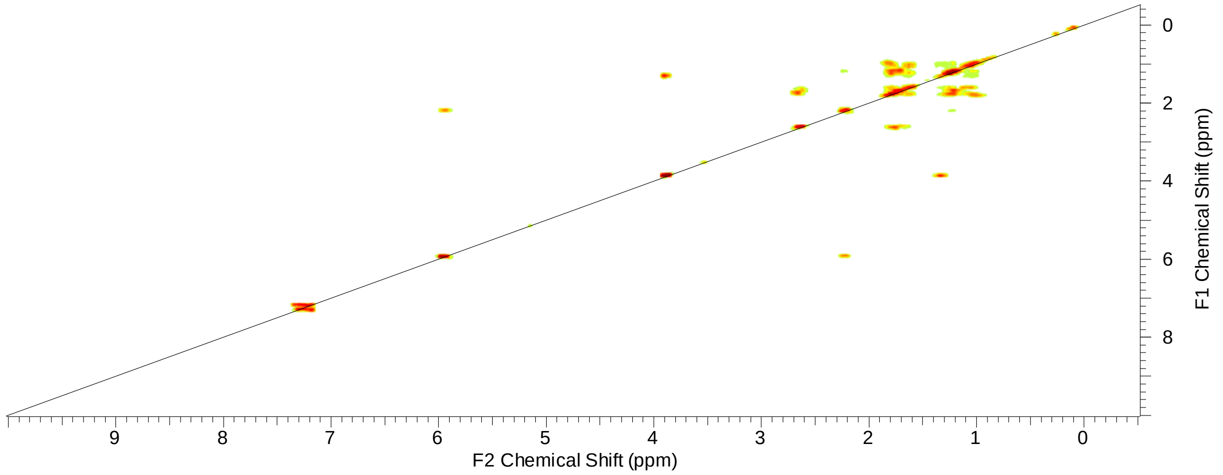

$^{1}H,^{13}C$-HMBC

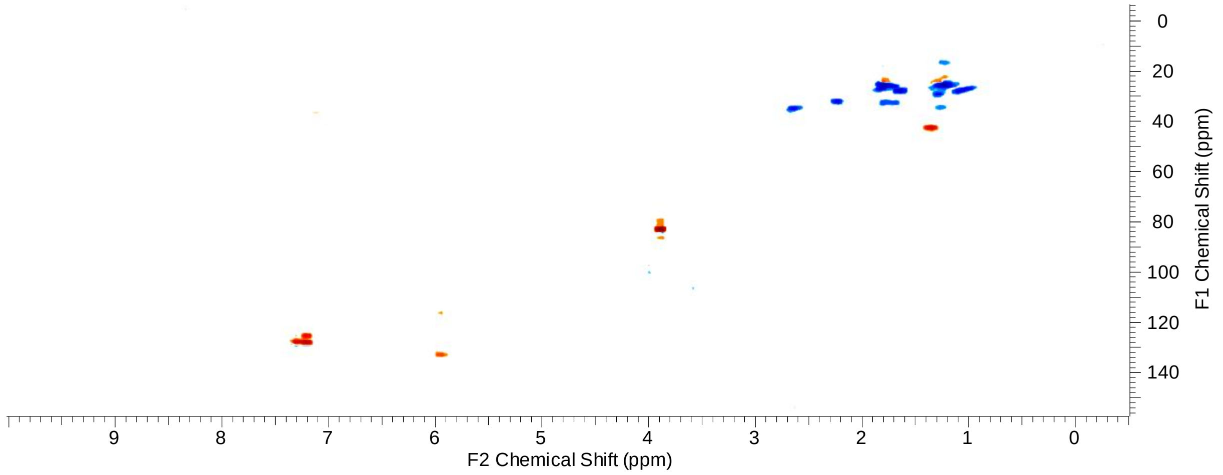

$^{1}H,^{13}C$-HSQC

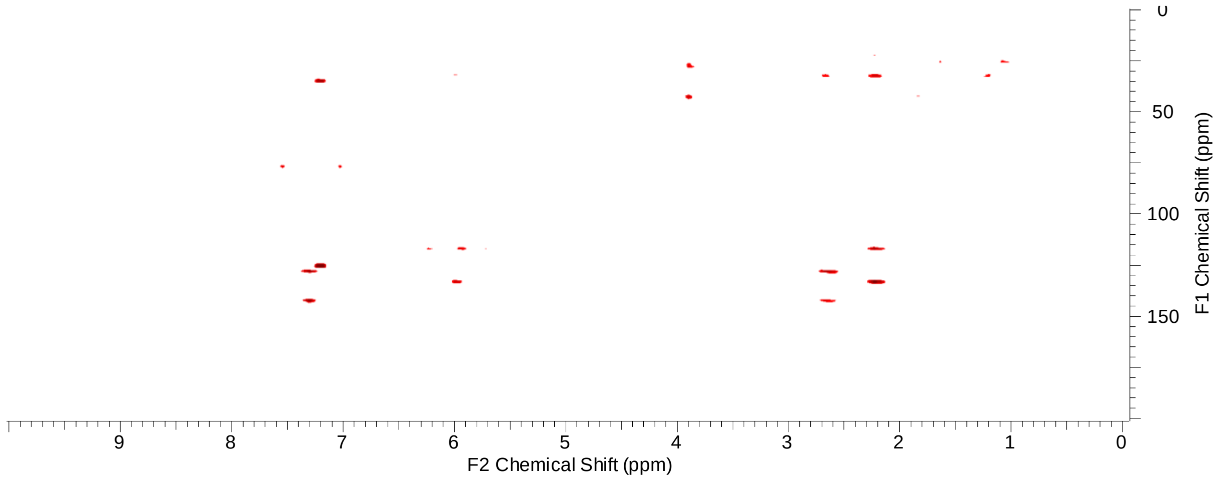

(4*R*,5*R*)-2-((*R*,*E*)-6-Bromo-1-phenylhex-5-en-3-yl)-4,5-dicyclohexyl-1,3,2-dioxaborolane (5b)

^{1}H-NMR (400 MHz, $CDCl_3$):

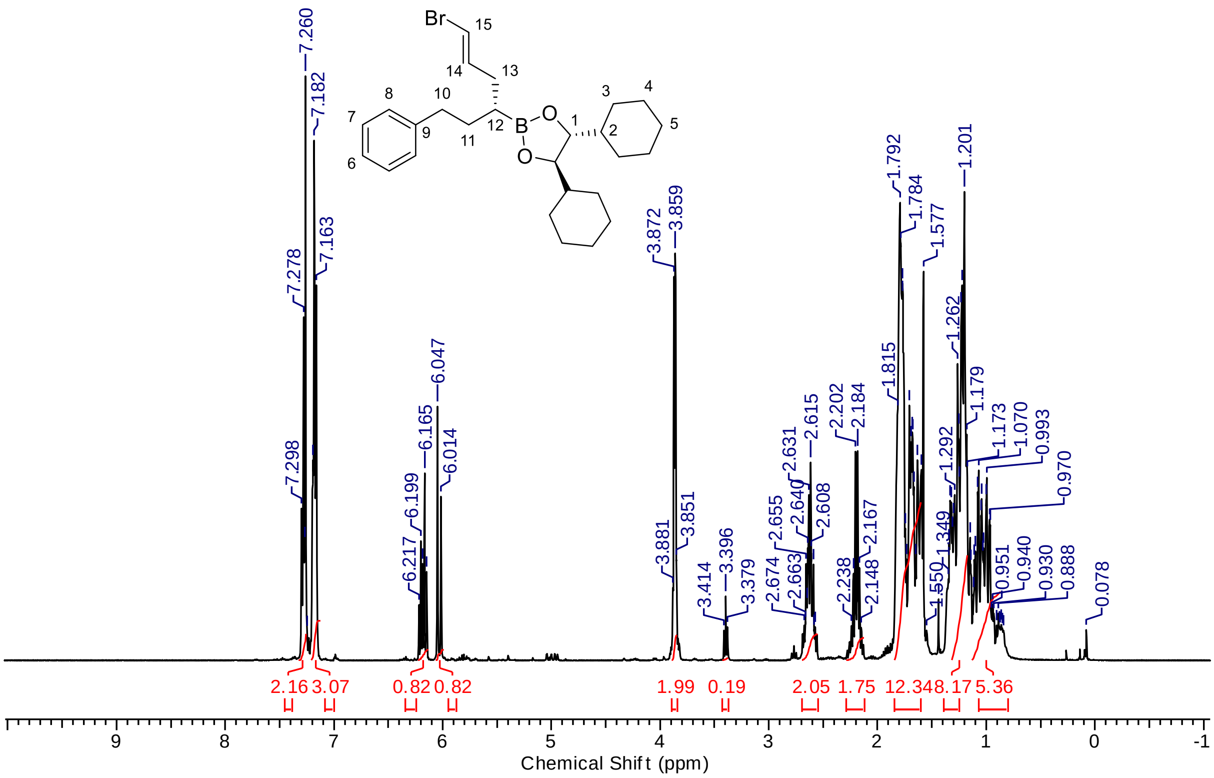

^{13}C-NMR (100 MHz, $CDCl_3$):

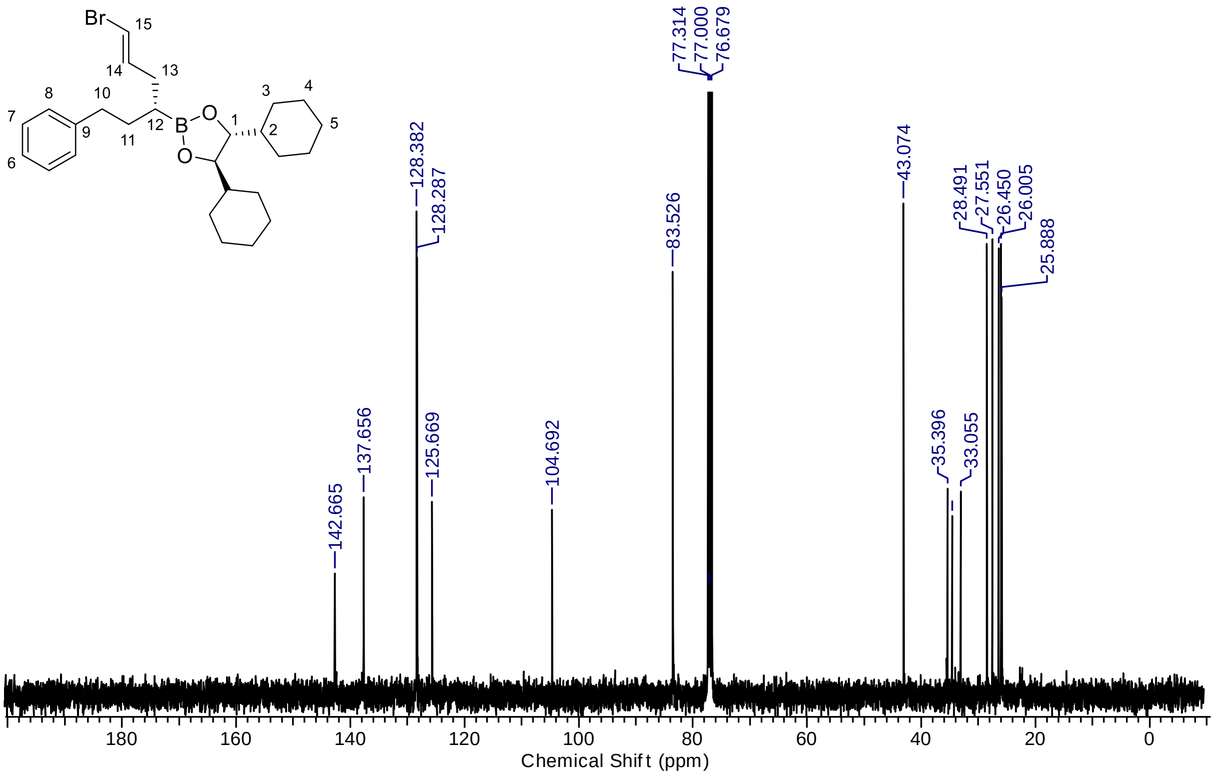

$^{1}H,^{1}H$-COSY

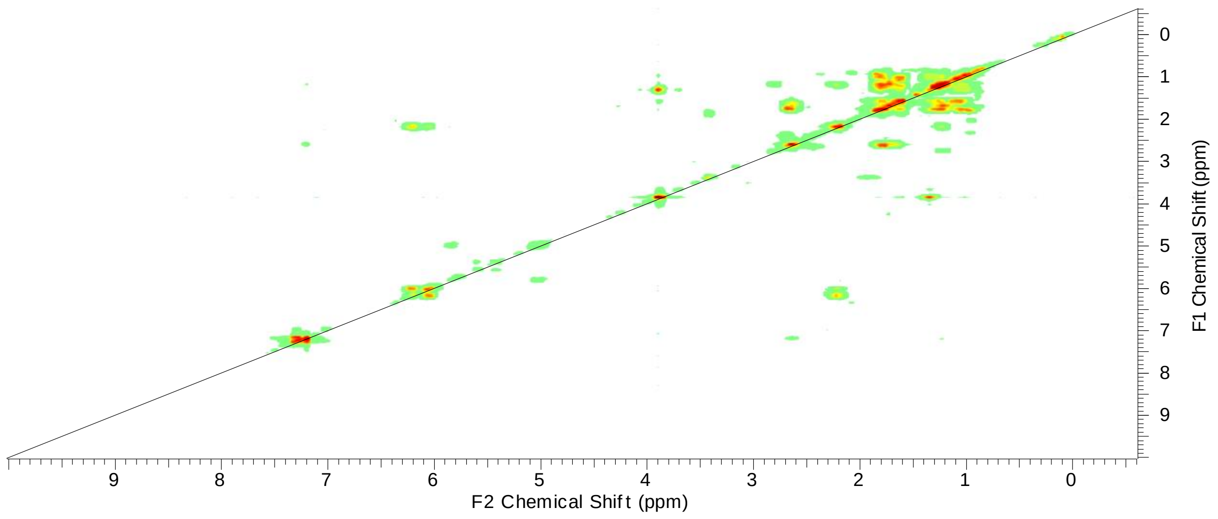

$^{1}H,^{13}C$-HSQC

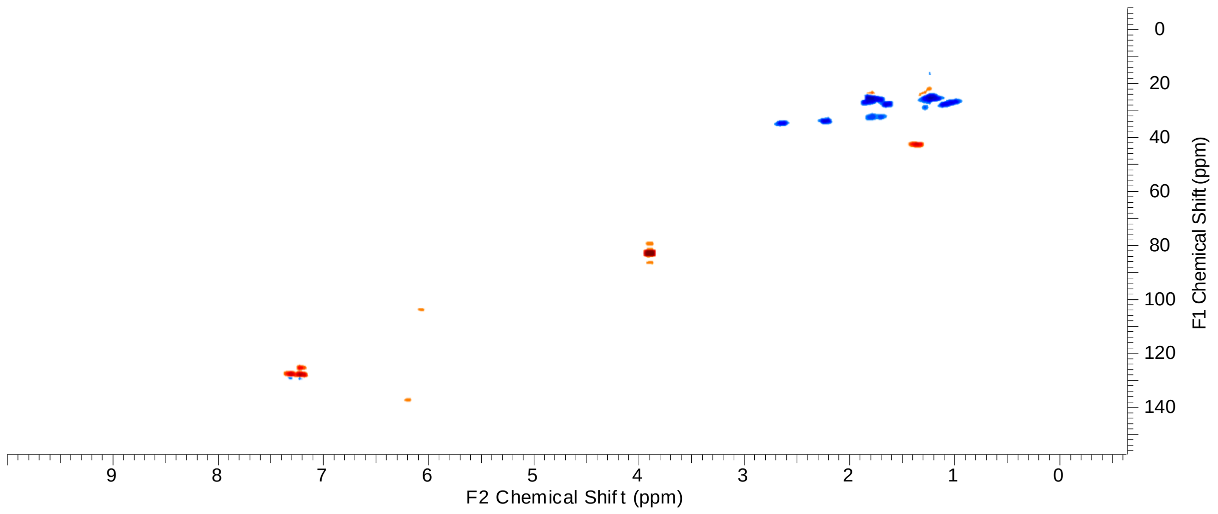

$^{1}H,^{13}C$-HMBC

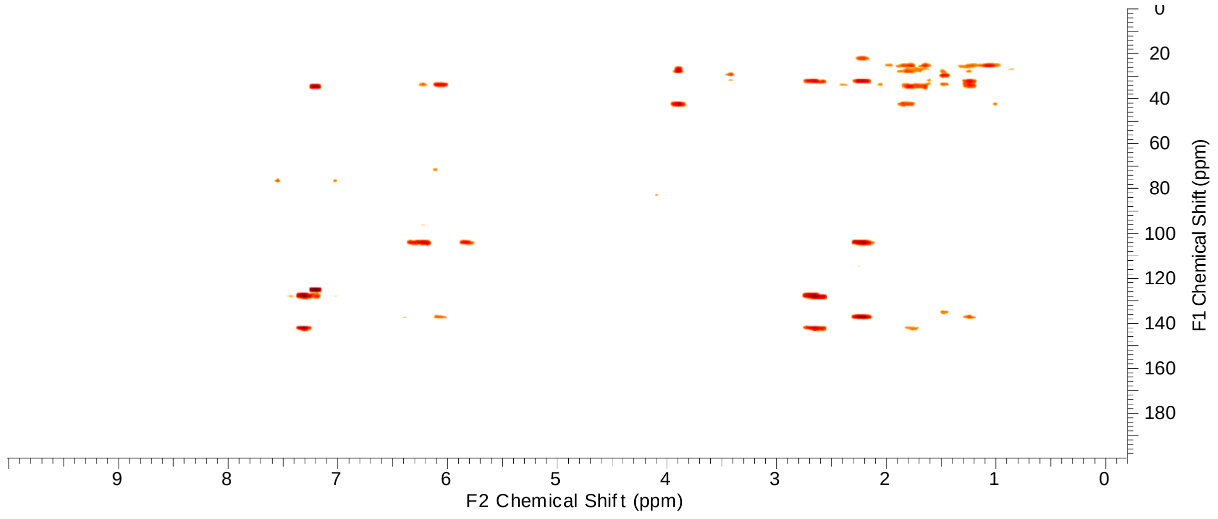

(4*R*,5*R*)-4,5-Dicyclohexyl-2-((*R*,*E*)-6-iodo-1-phenylhex-5-en-3-yl)-1,3,2-dioxaborolane (5c)

^{1}H-NMR (400 MHz, $CDCl_3$):

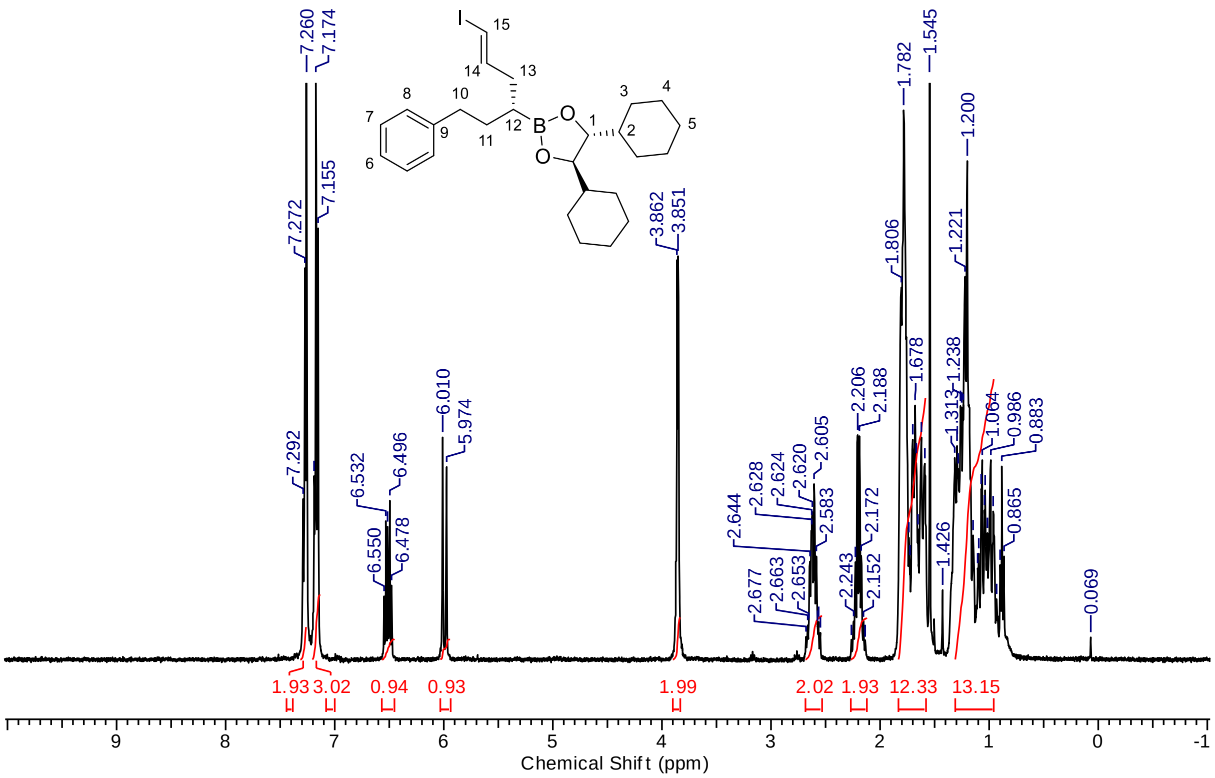

^{13}C-NMR (400 MHz, $CDCl_3$):

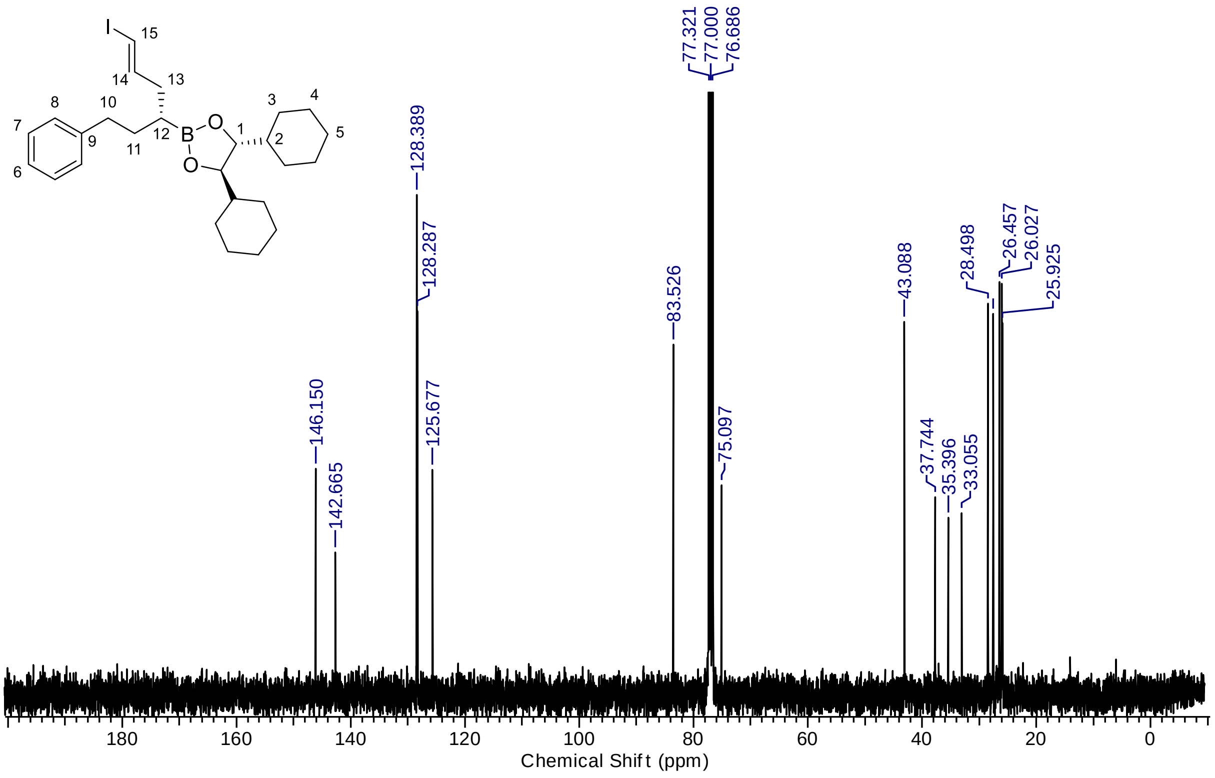

$^{1}H,^{1}H$-COSY

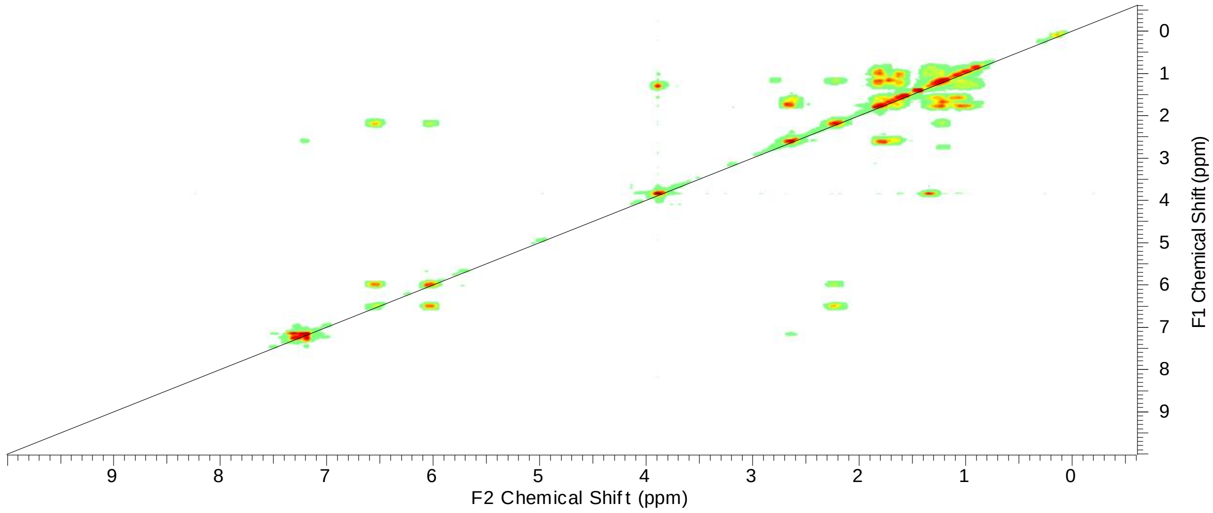

$^{1}H,^{13}C$-HSQC

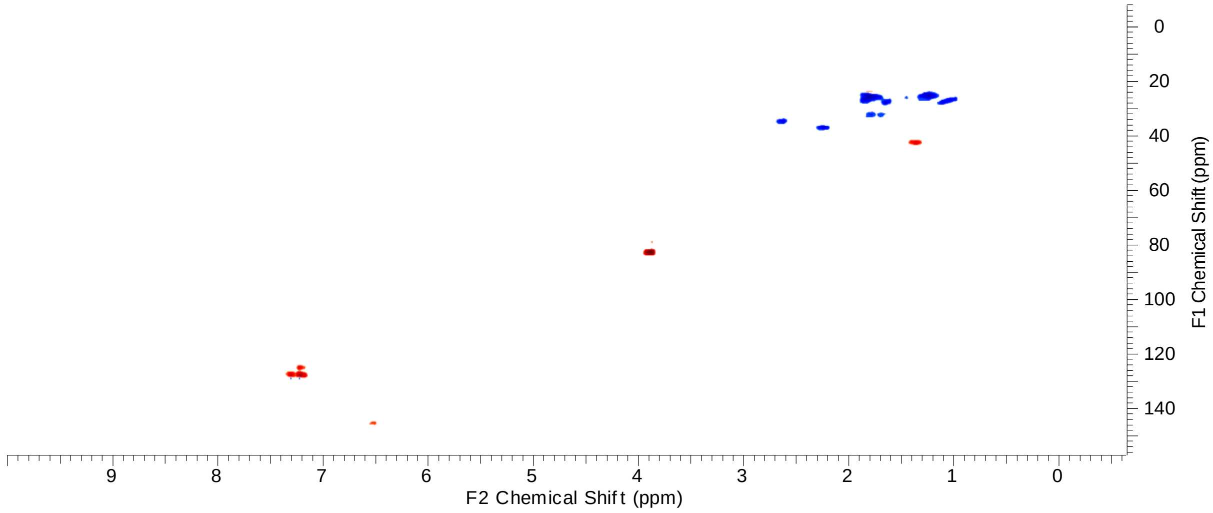

$^{1}H,^{13}C$-HMBC

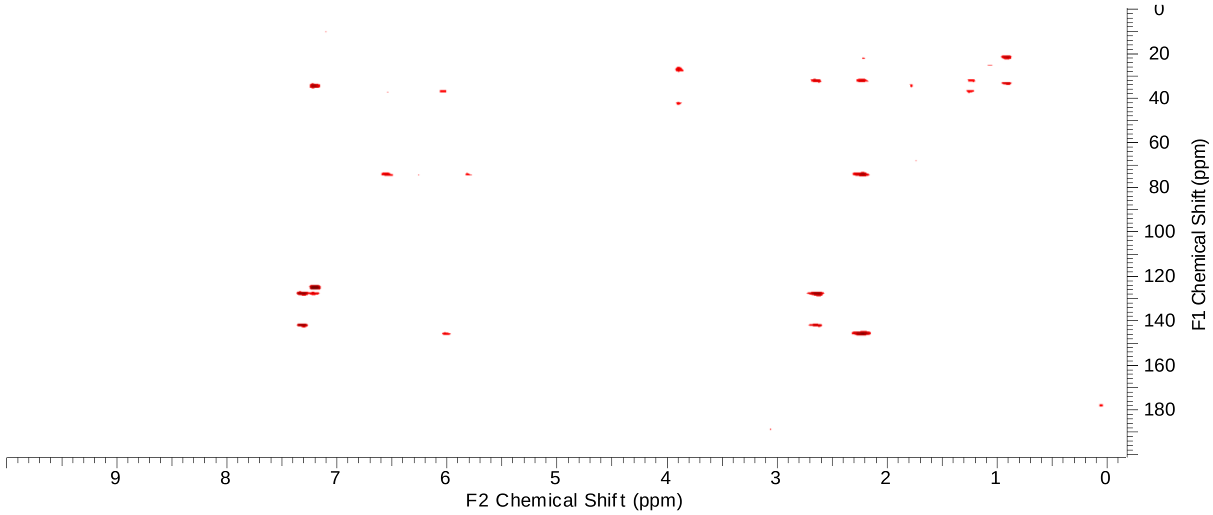

(4*R*,5*R*)-4,5-Dicyclohexyl-2-((*R*,*E*)-1,6-diphenylhex-5-en-3-yl)-1,3,2-dioxaborolane (6a)

^{1}H-NMR (400 MHz, $CDCl_3$):

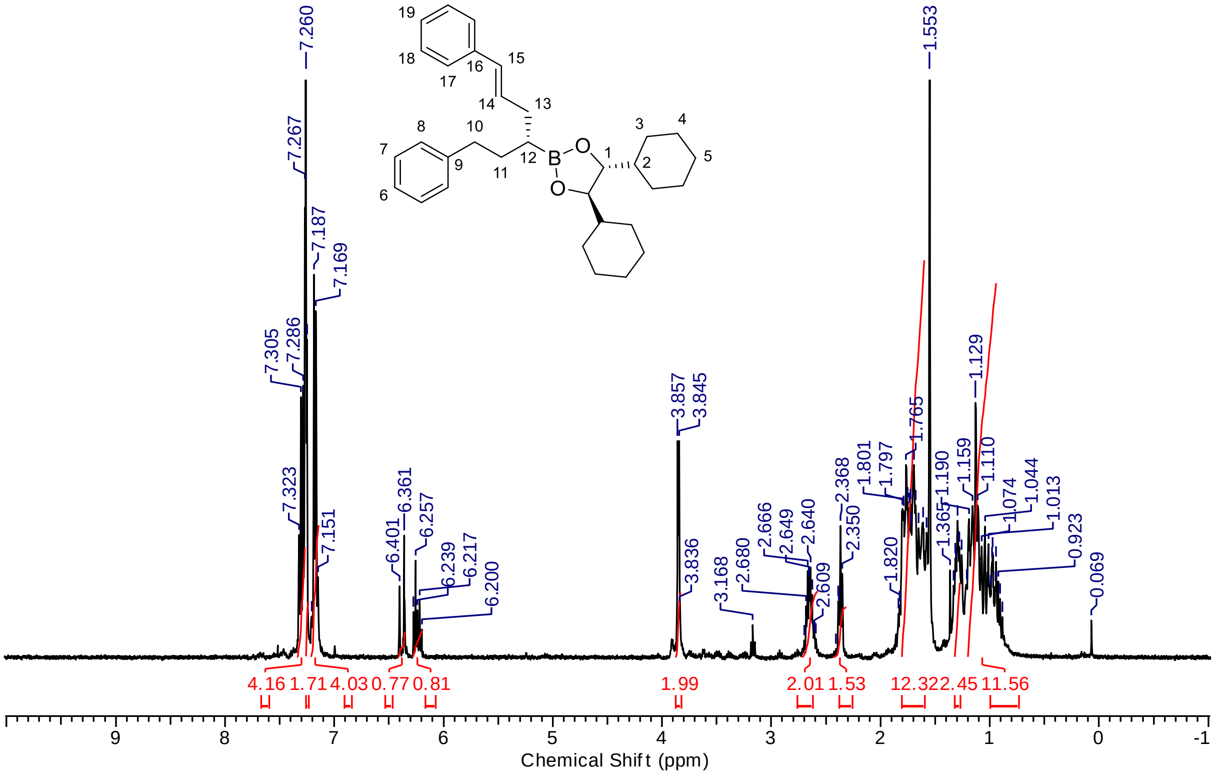

^{13}C-NMR (100 MHz, $CDCl_3$):

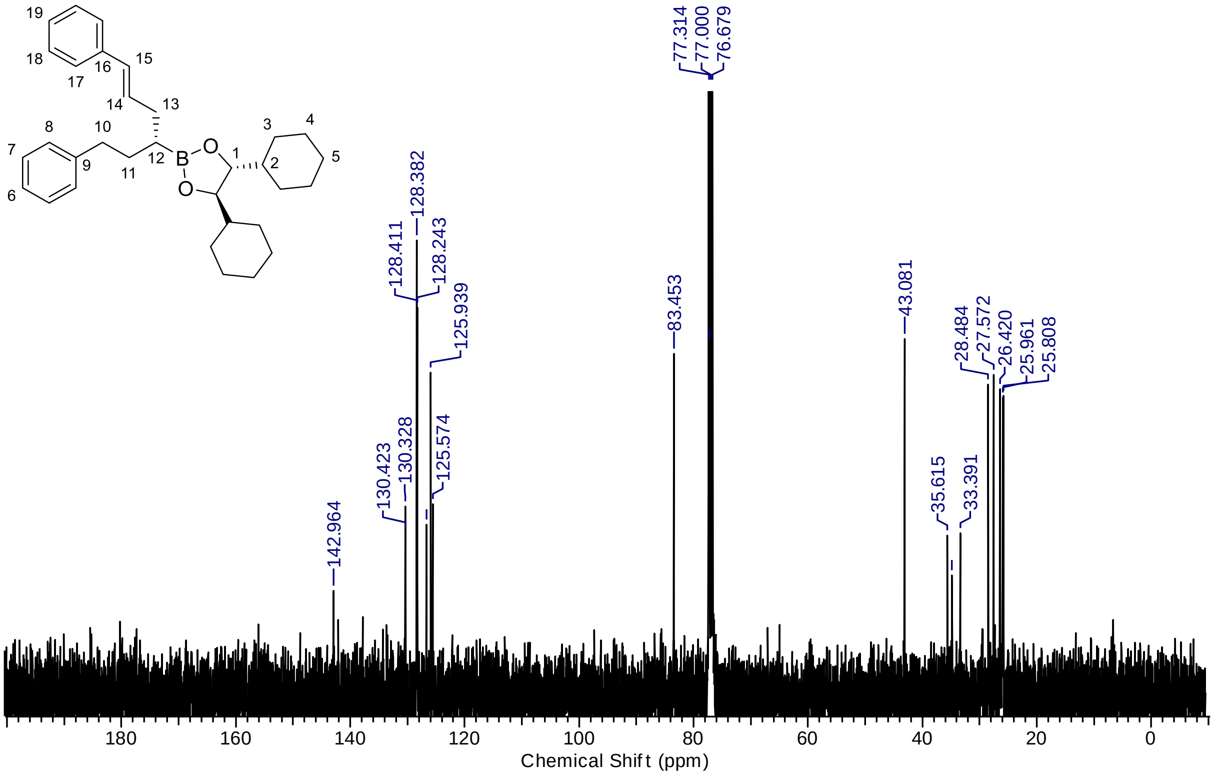

$^{1}H,^{1}H$-COSY

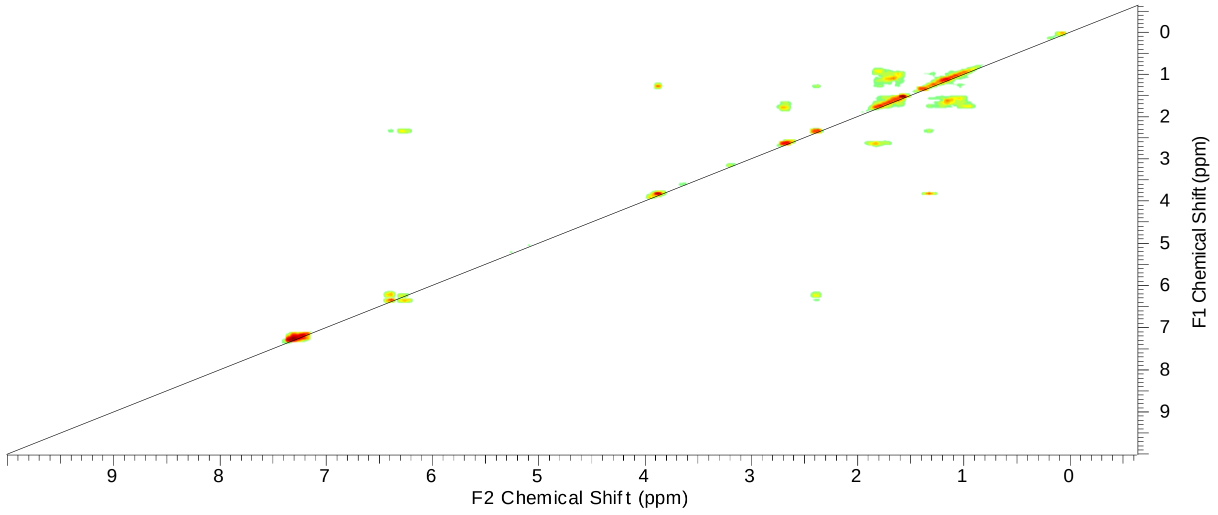

$^{1}H,^{13}C$-HSQC

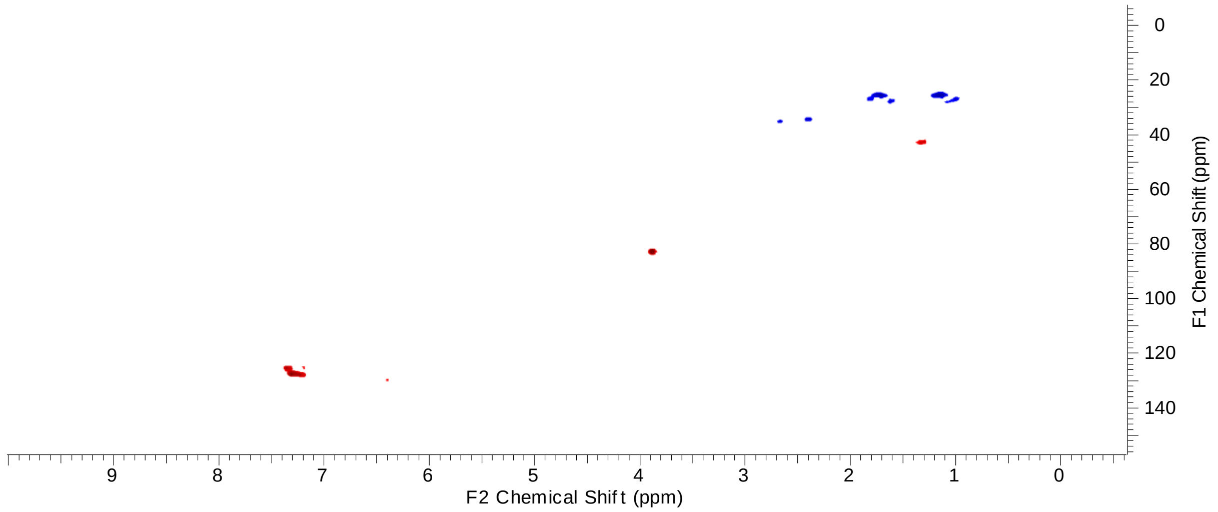

$^{1}H,^{13}C$-HMBC

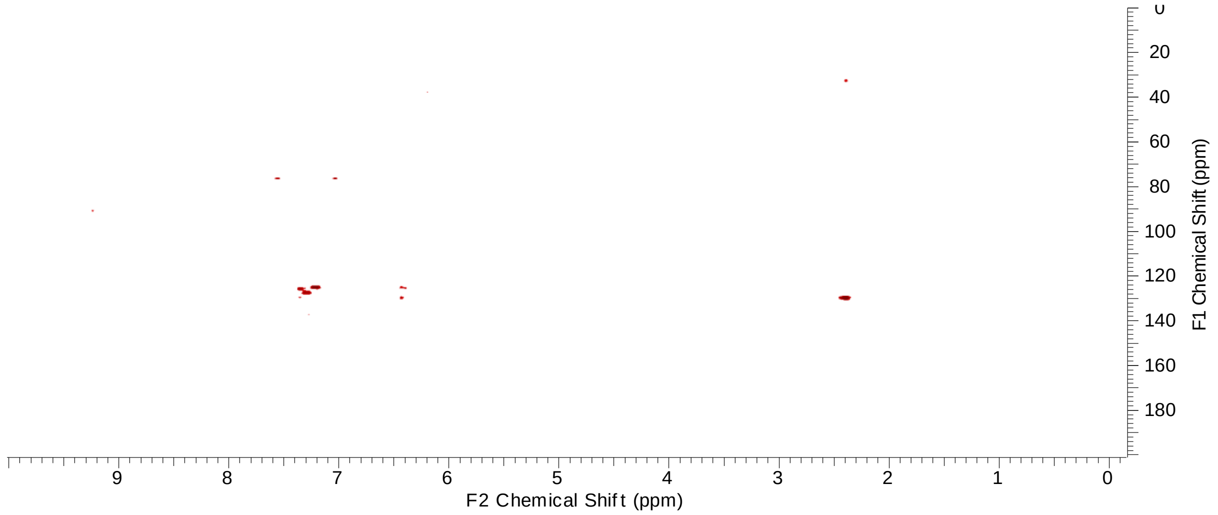

(4*R*,5*R*)-4,5-Dicyclohexyl-2-((*R*,*E*)-1-phenyl-6-(*p*-tolyl)hex-5-en-3-yl)-1,3,2-dioxaborolane (6b)

^{1}H-NMR (400 MHz, $CDCl_3$):

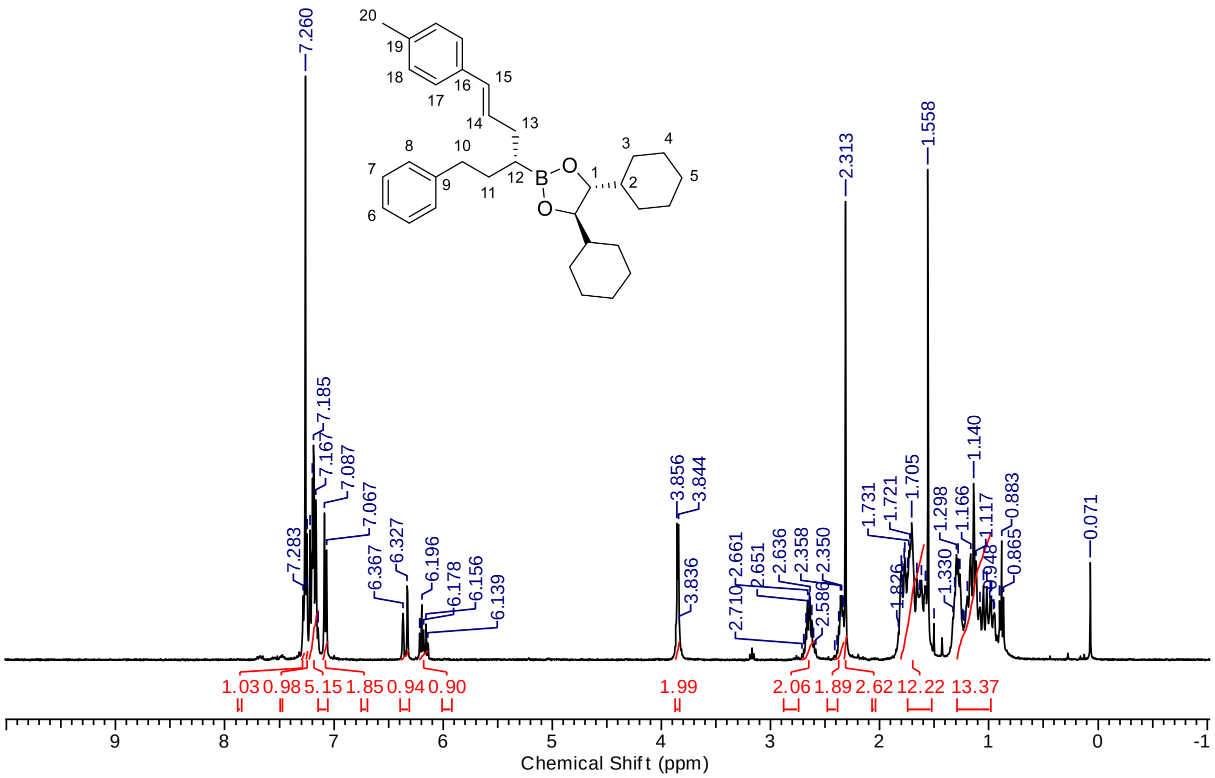

^{13}C-NMR (100 MHz, $CDCl_3$):

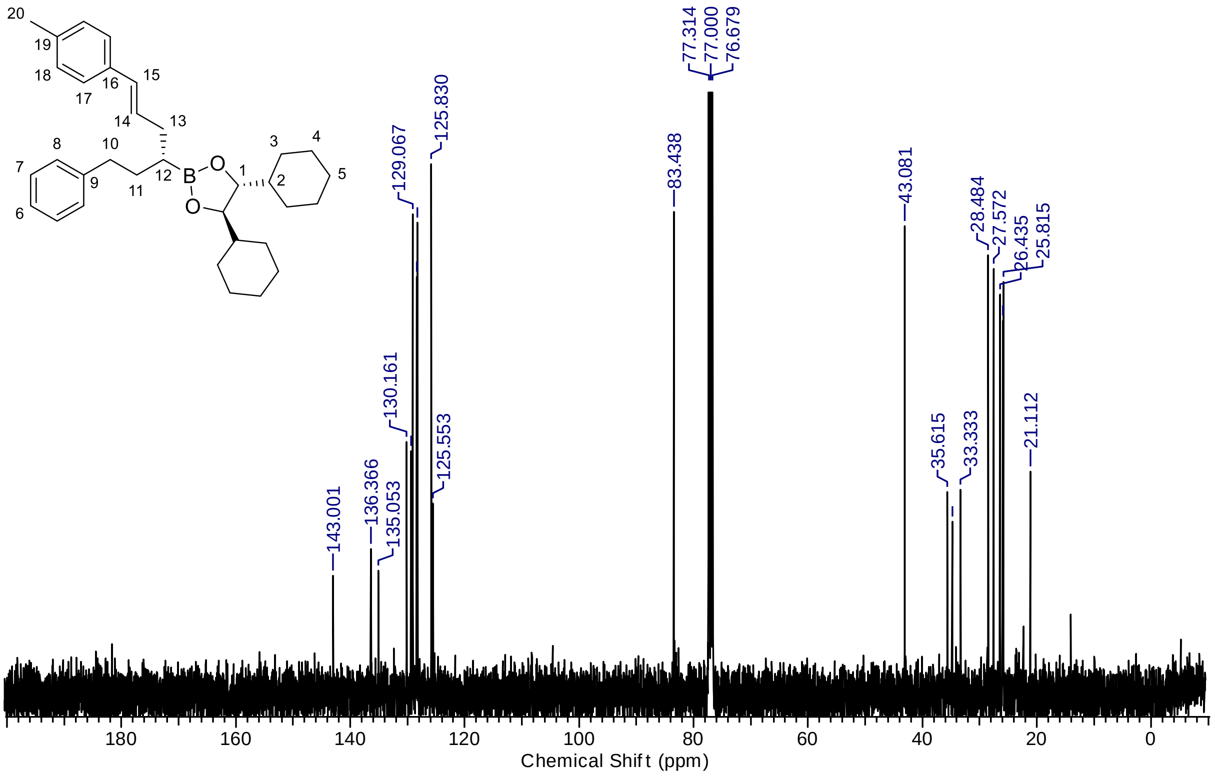

$^{1}H,^{1}H$-COSY

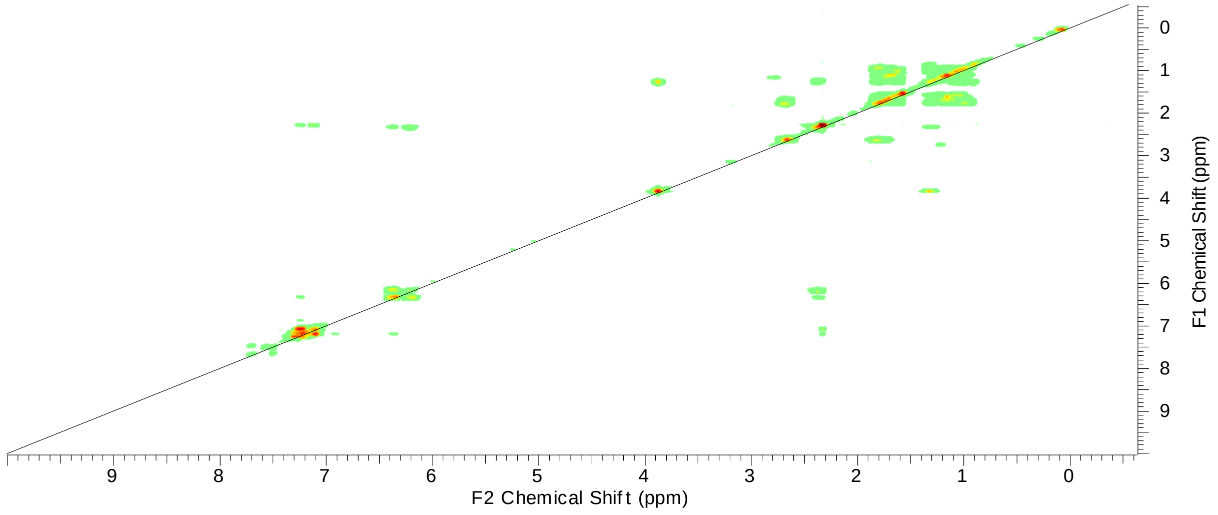

$^{1}H,^{13}C$-HSQC

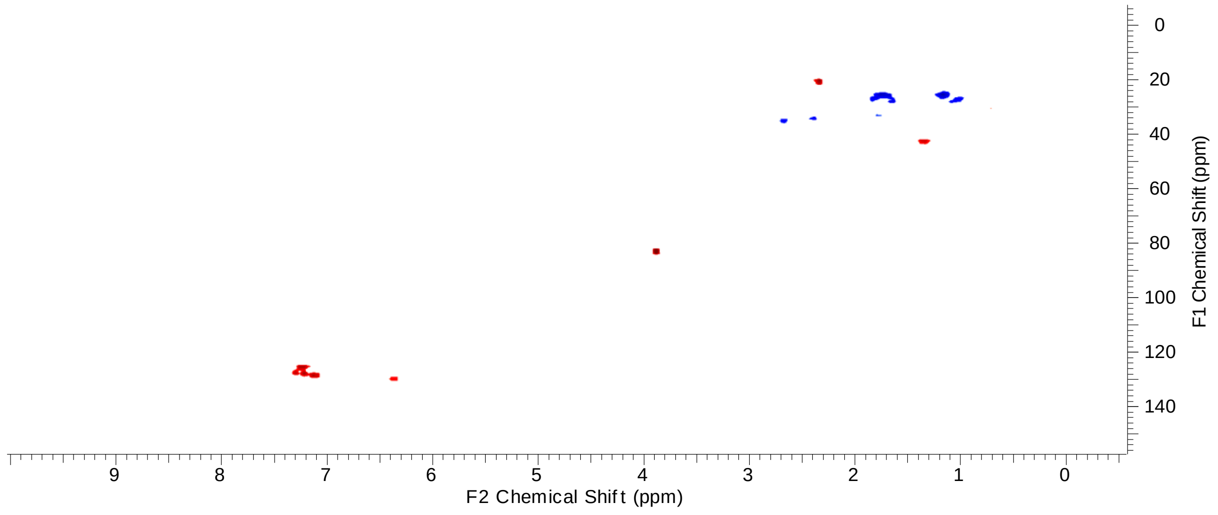

$^{1}H,^{13}C$-HMBC

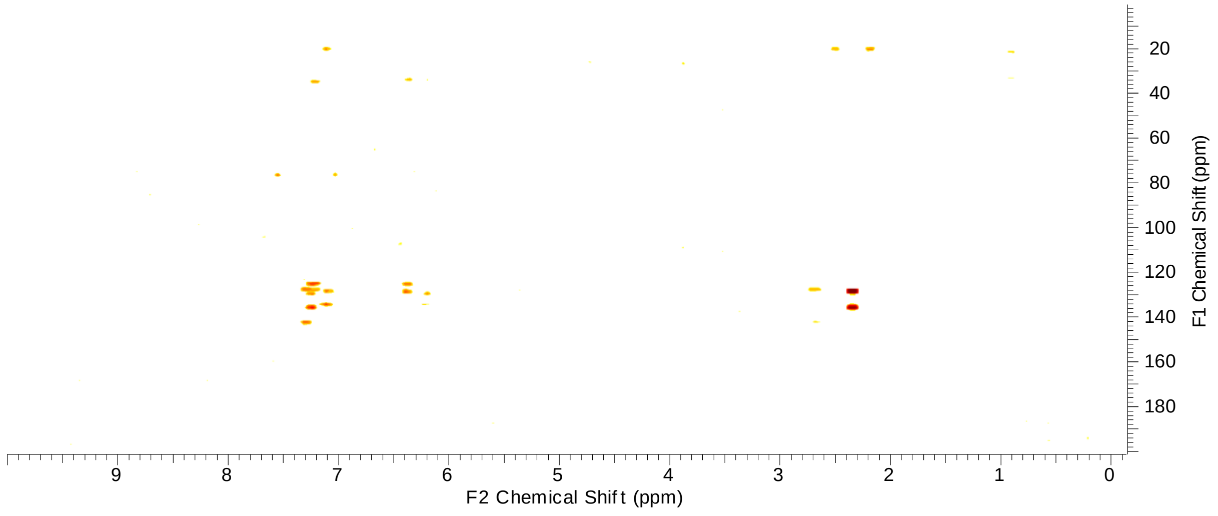

(4*R*,5*R*)-4,5-dicyclohexyl-2-((*R*,*E*)-1-phenyl-6-(*o*-tolyl)hex-5-en-3-yl)-1,3,2-dioxaborolane (6c)

^{1}H-NMR (400 MHz, $CDCl_3$):

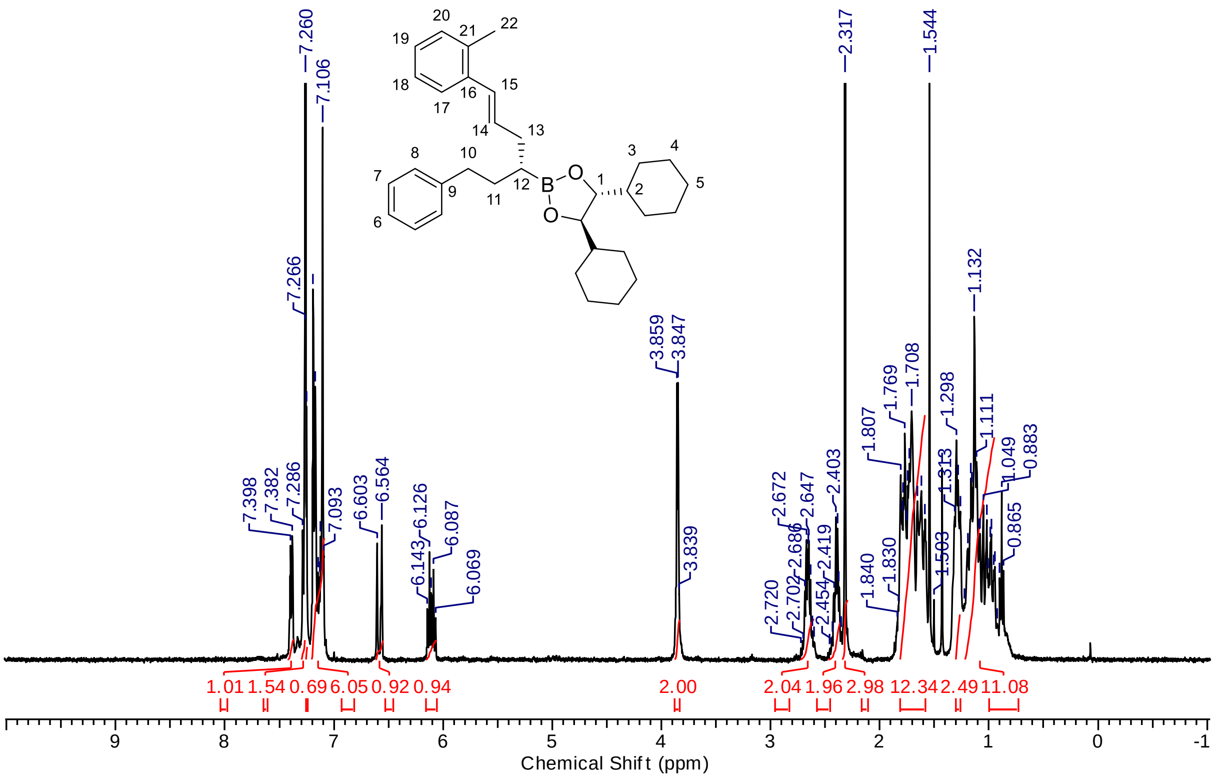

^{13}C-NMR (100 MHz, $CDCl_3$):

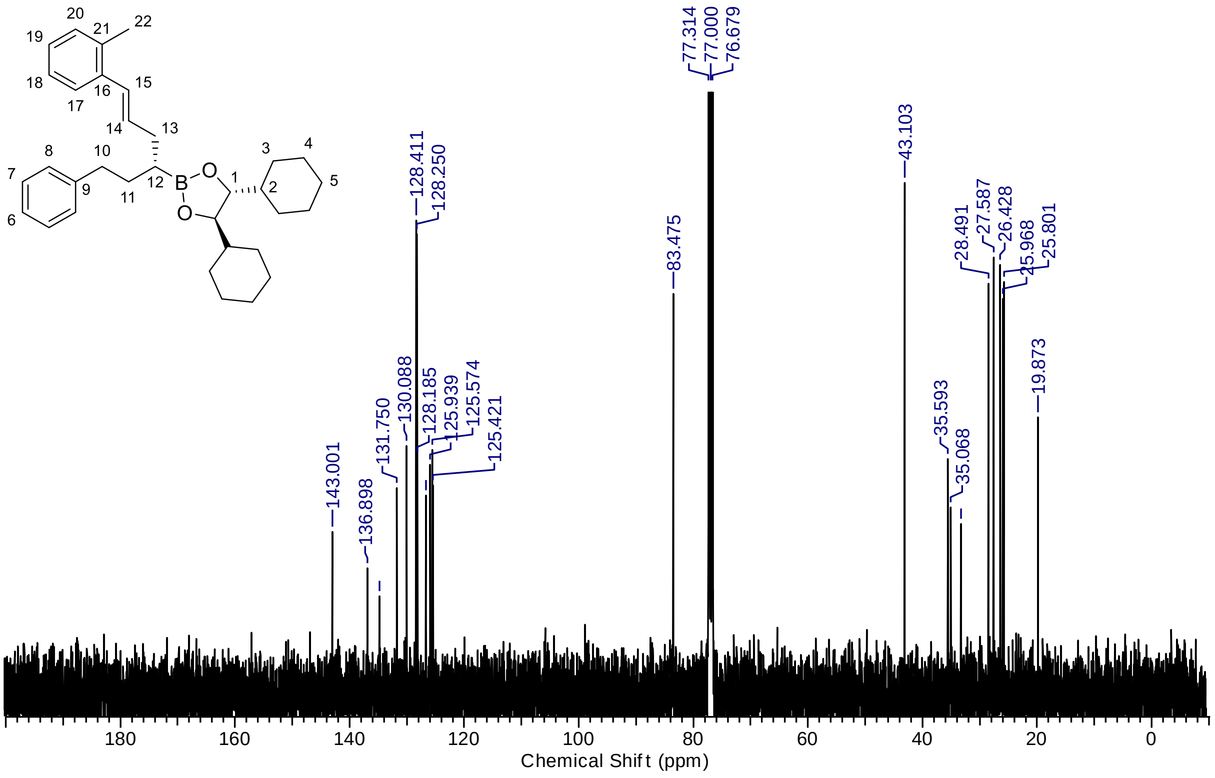

$^1H,^1H$-COSY

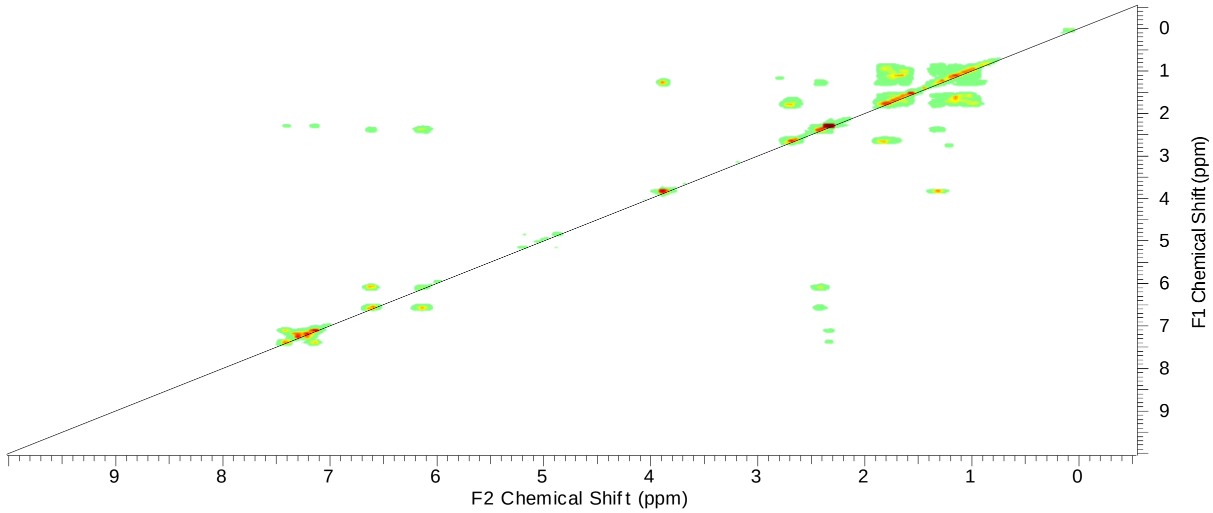

$^1H,^{13}C$-HSQC

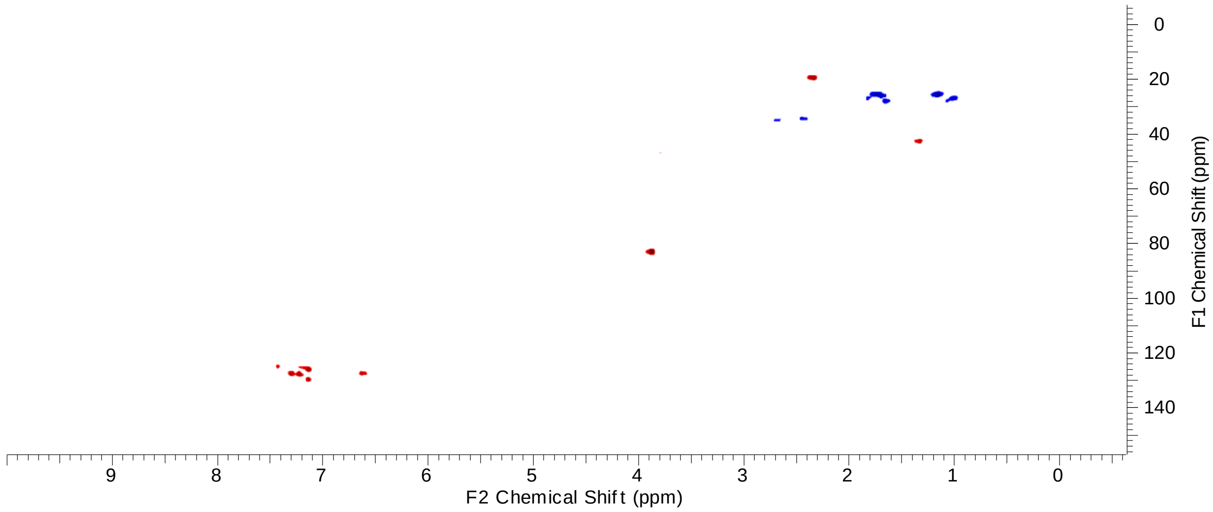

$^1H,^{13}C$-HMBC

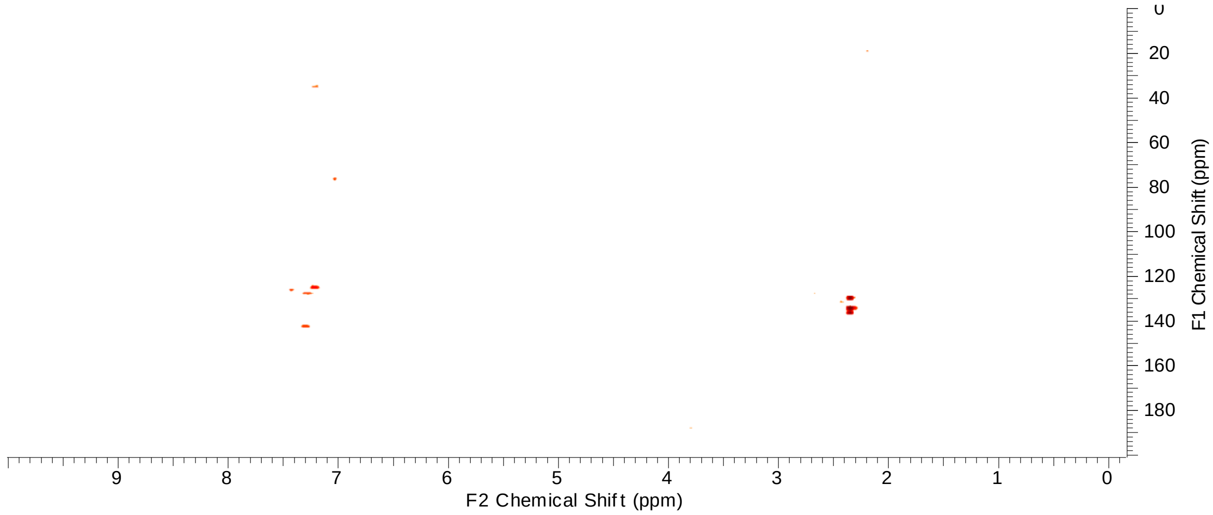

(4*R*,5*R*)-4,5-Dicyclohexyl-2-((*R*,*E*)-6-(4-methoxyphenyl)-1-phenylhex-5-en-3-yl)-1,3,2-dioxaborolane (6d)

^{1}H-NMR (400 MHz, $CDCl_3$):

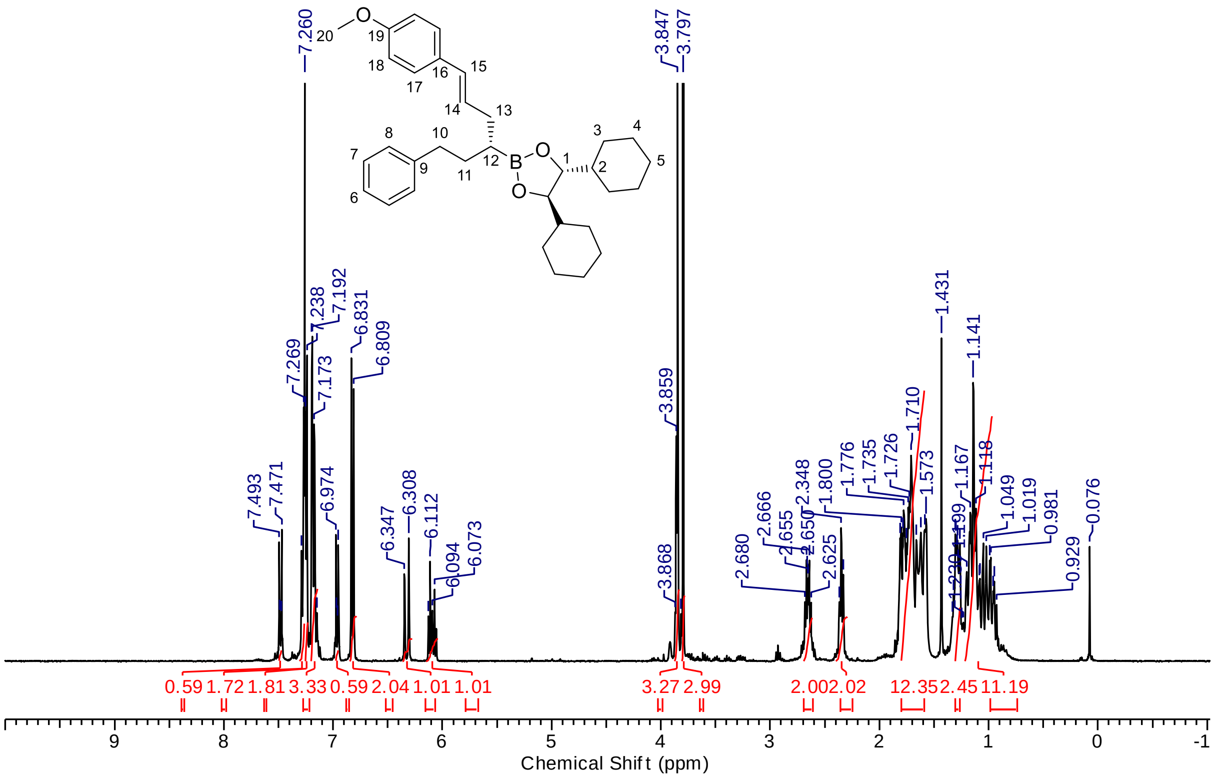

Additional signals arising from anisole

^{13}C-NMR (100 MHz, $CDCl_3$):

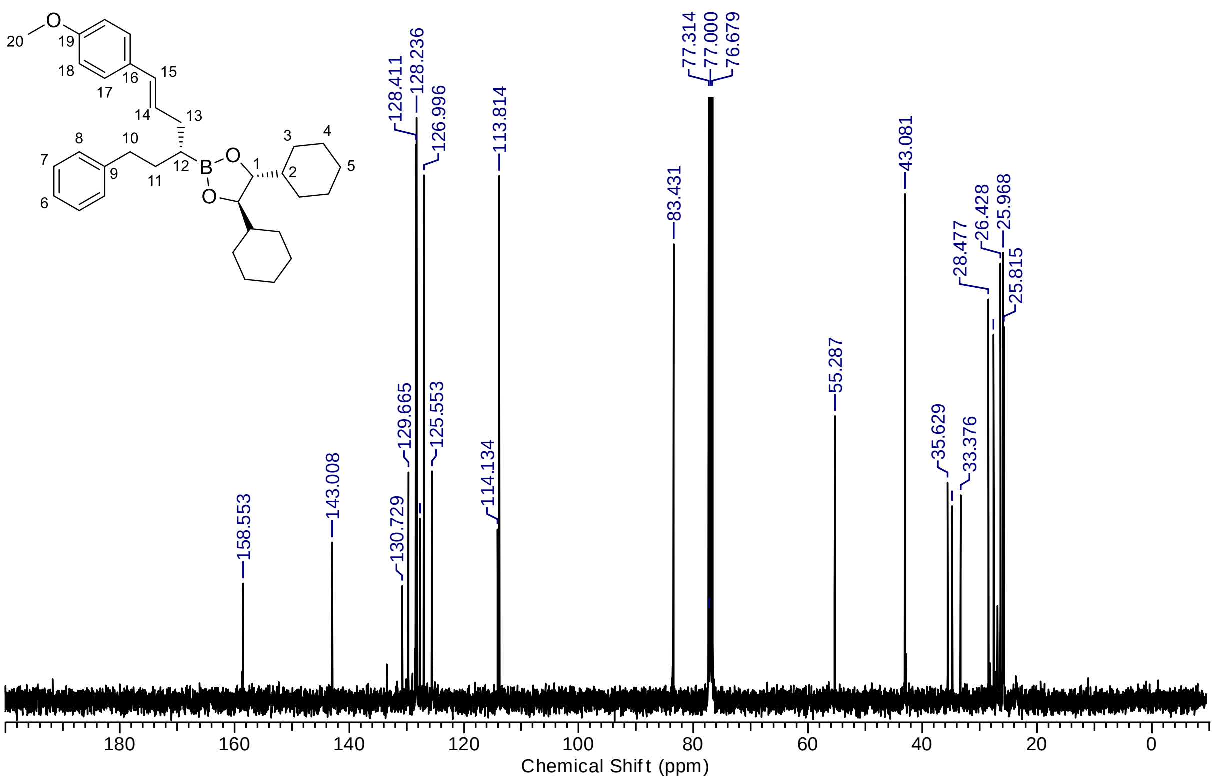

Additional signals arrising from anisole

$^{1}H,^{1}H$-COSY

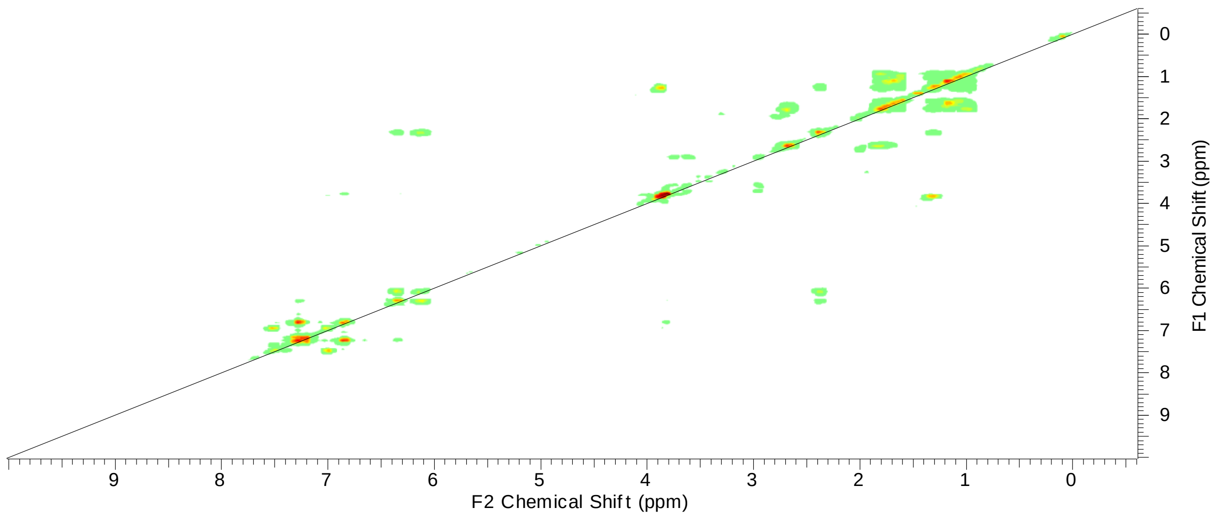

$^{1}H,^{13}C$-HSQC

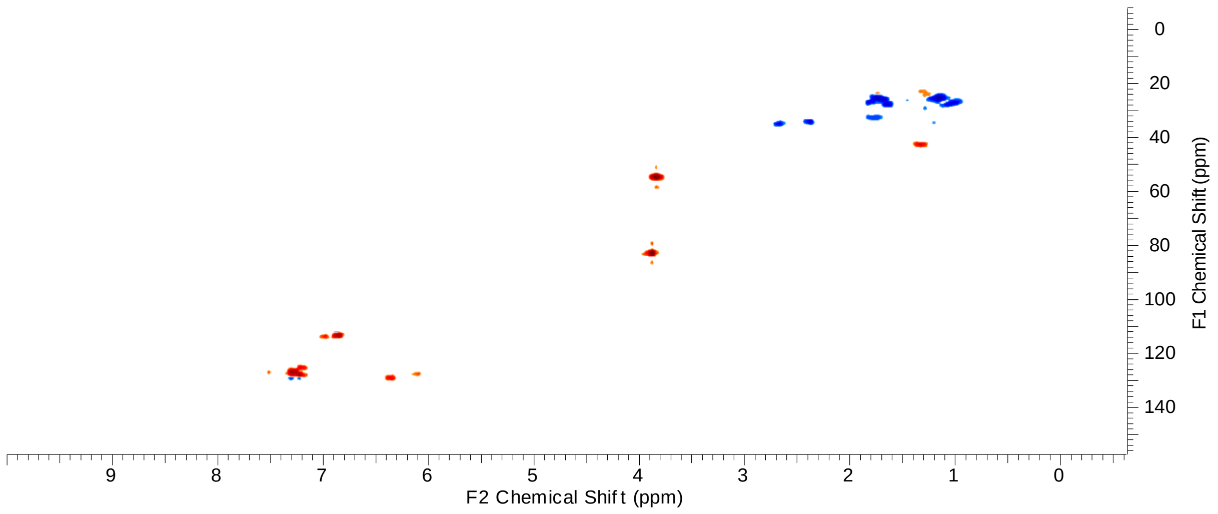

$^{1}H,^{13}C$-HMBC

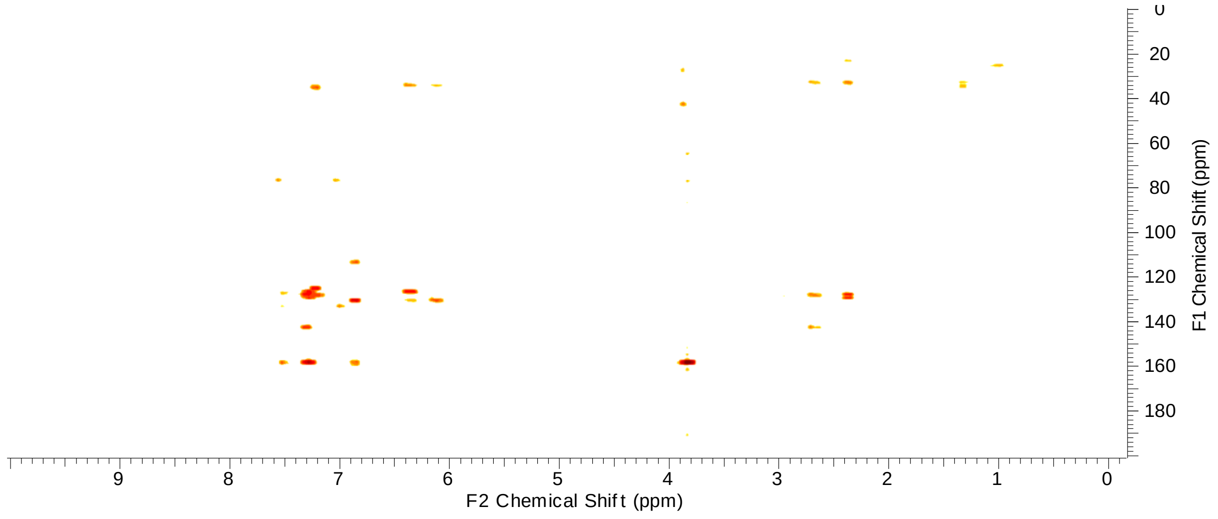

(*R*,*E*)-6-(benzo[d][1,3]dioxol-5-yl)-1-phenylhex-5-en-3-ol (7e)

^{1}**H-NMR** (400 MHz, $CDCl_3$):

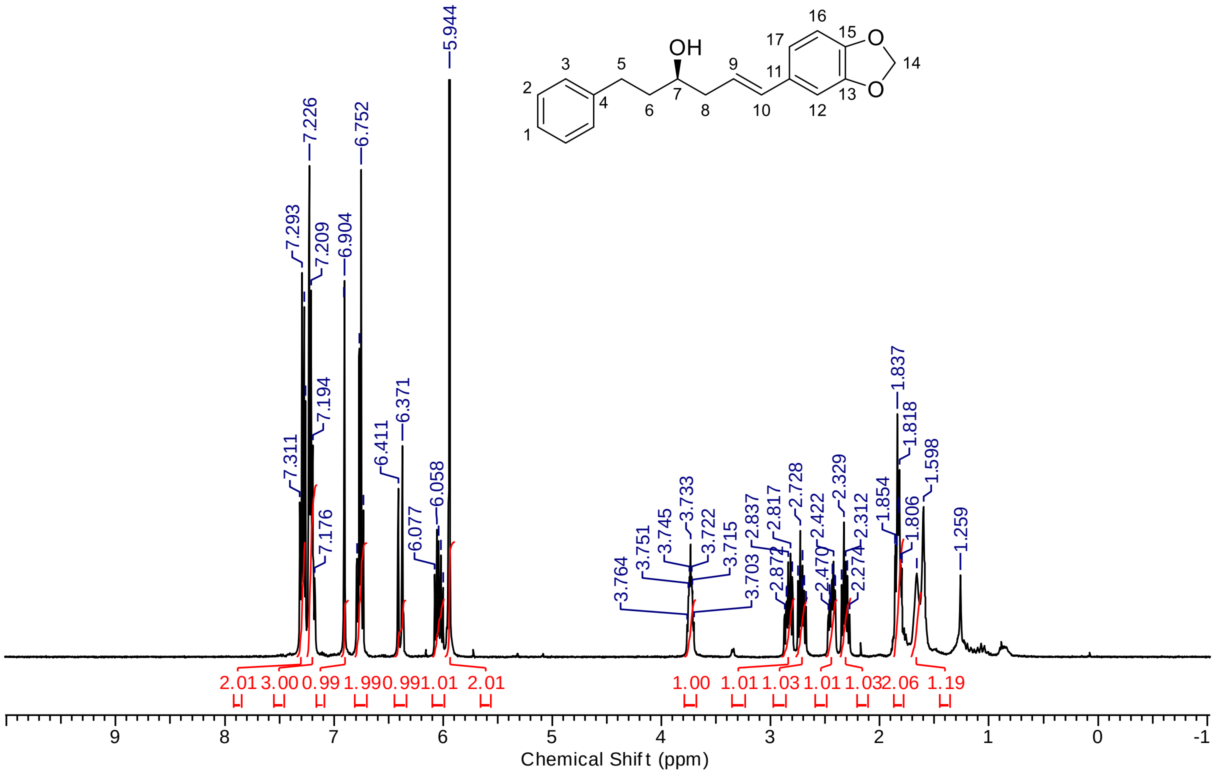

^{13}**C-NMR** (100 MHz, $CDCl_3$):

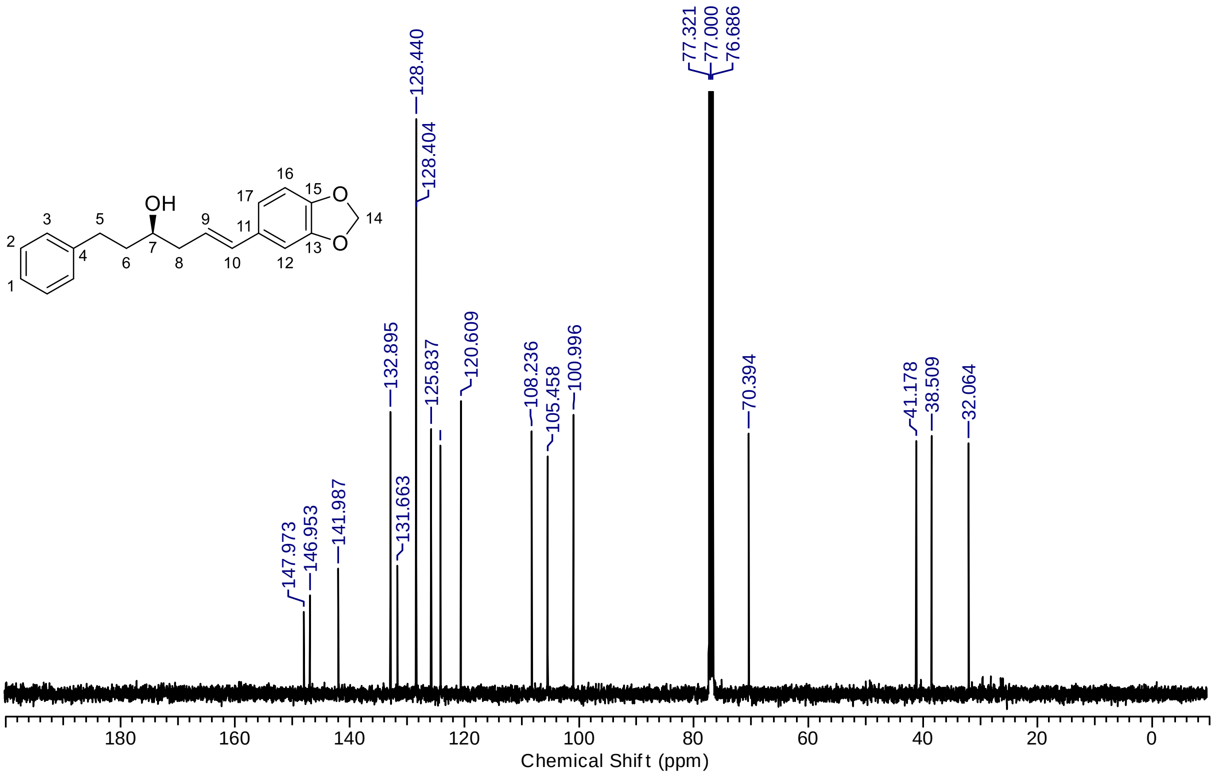

$^{1}H,^{1}H$-COSY

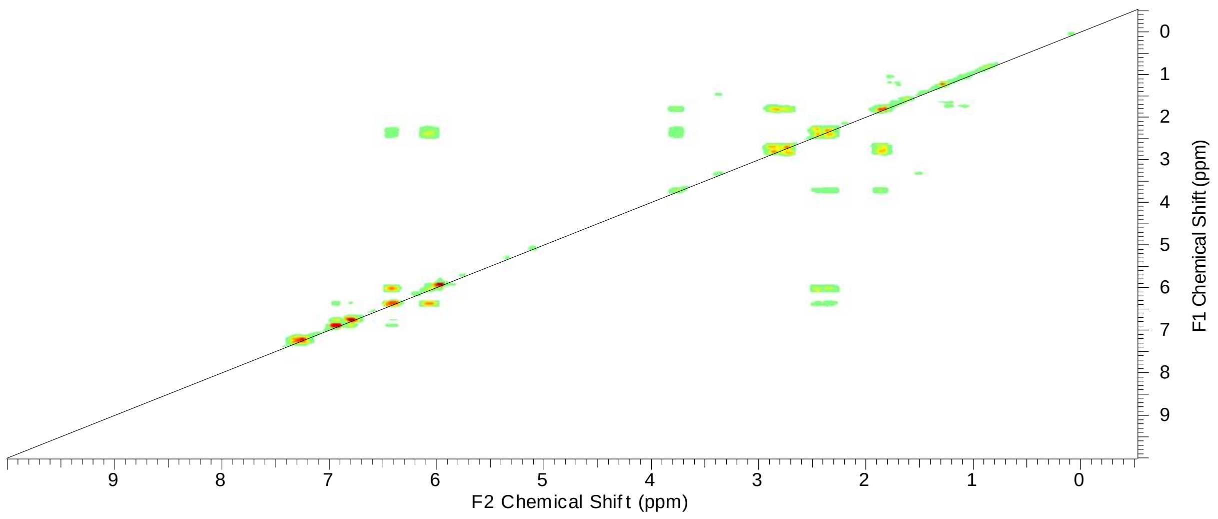

$^{1}H,^{13}C$-HSQC

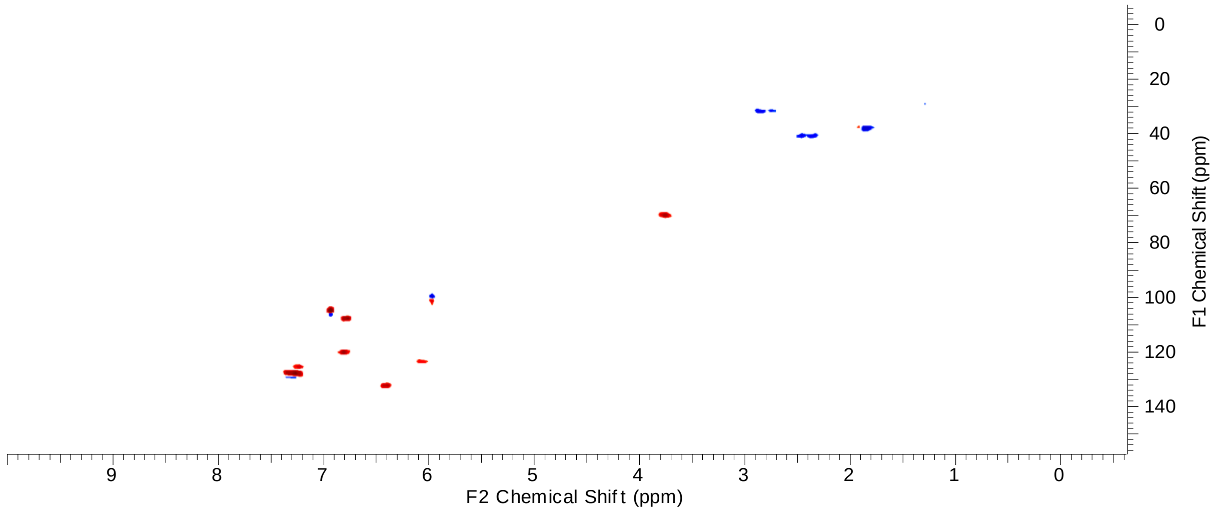

$^{1}H,^{13}C$-HMBC

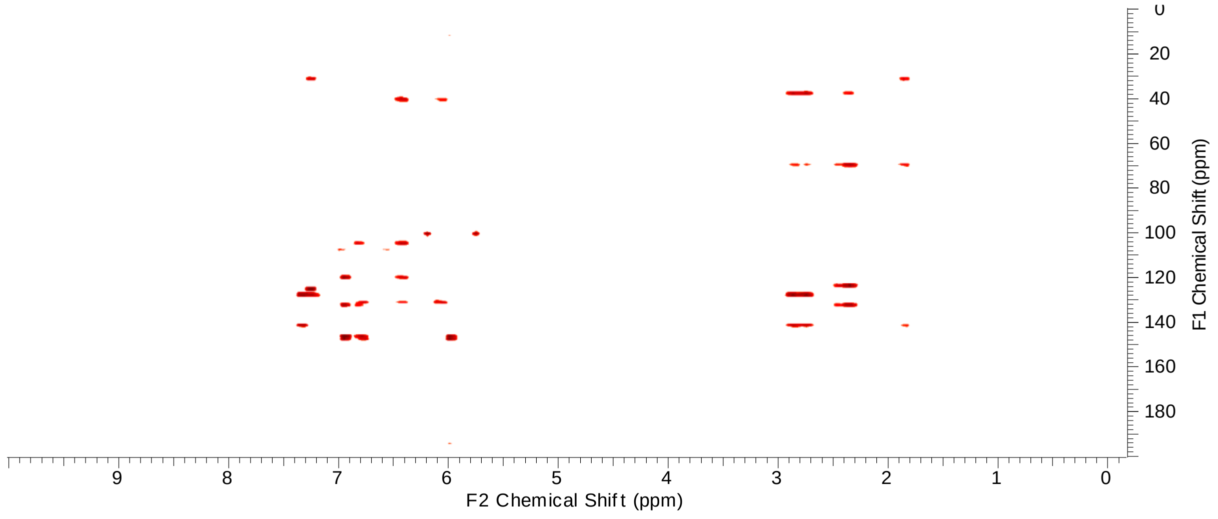

(4*R*,5*R*)-4,5-Dicyclohexyl-2-((*R*,*E*)-6-(2-methoxyphenyl)-1-phenylhex-5-en-3-yl)-1,3,2-dioxaborolane (6f)

^{1}H-NMR (400 MHz, $CDCl_3$):

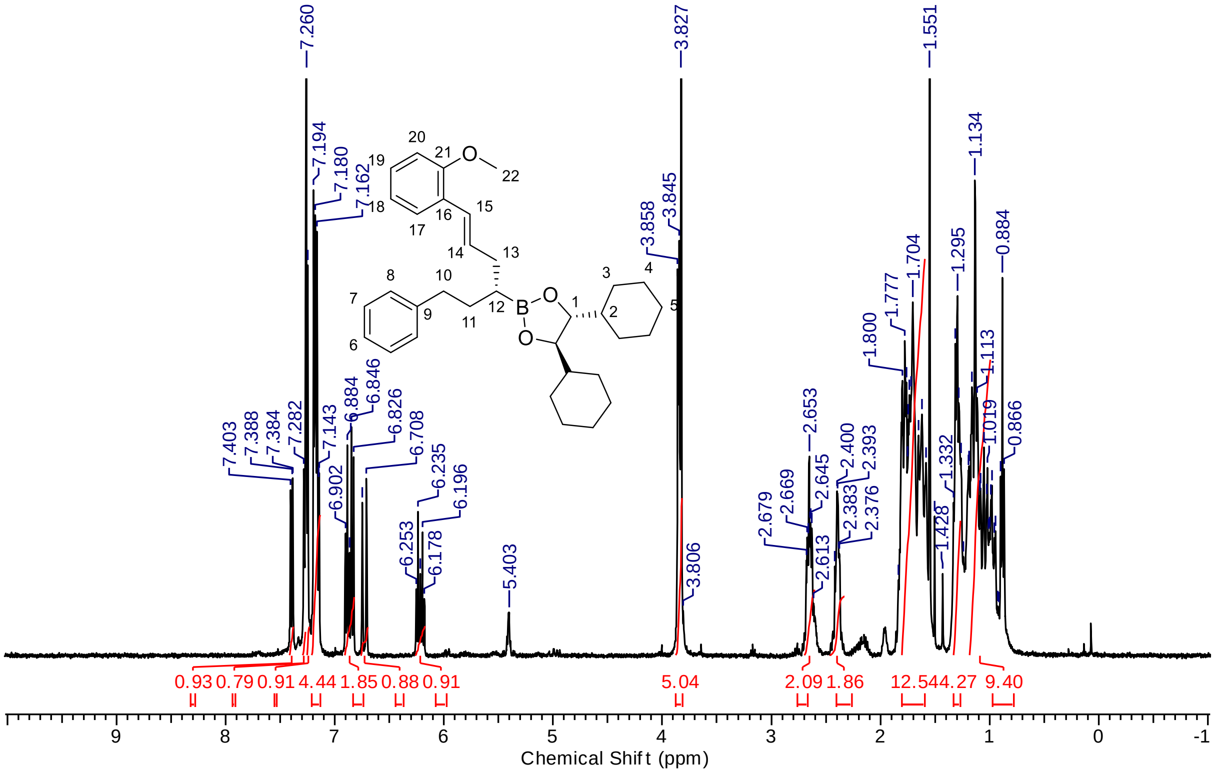

^{13}C-NMR (100 MHz, $CDCl_3$):

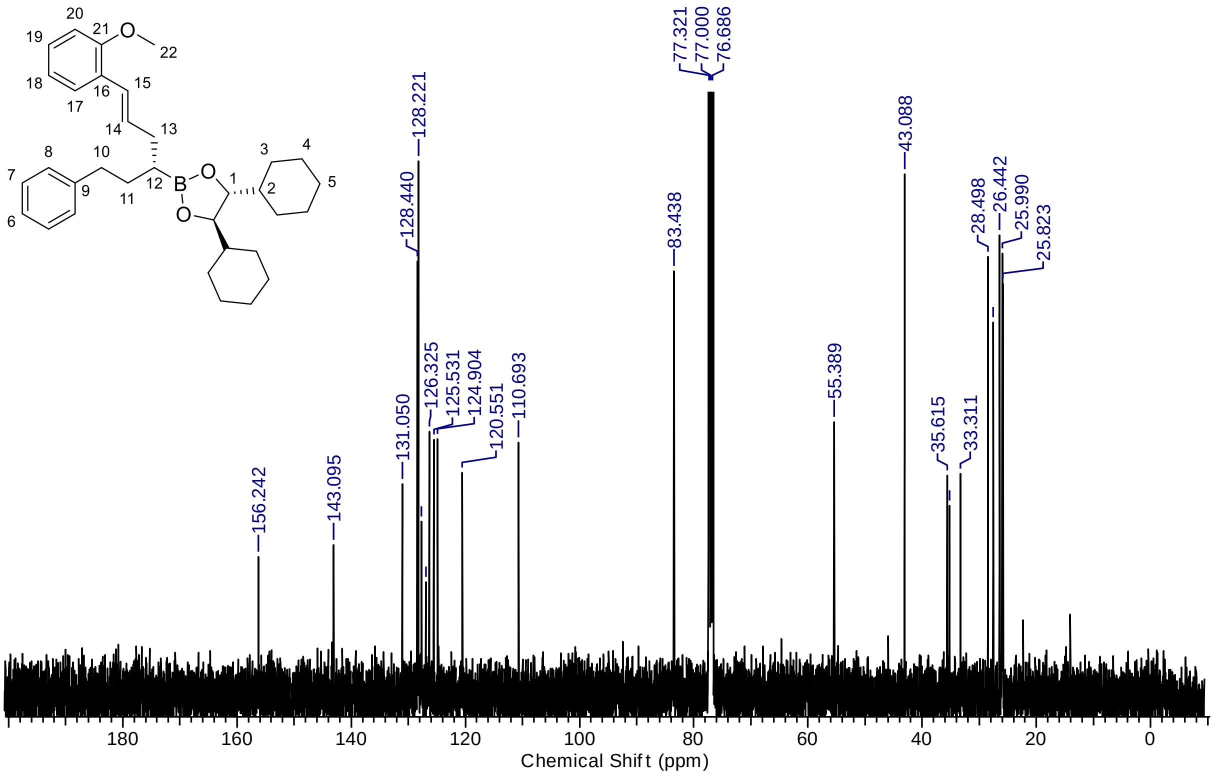

$^1H,^1H$-COSY

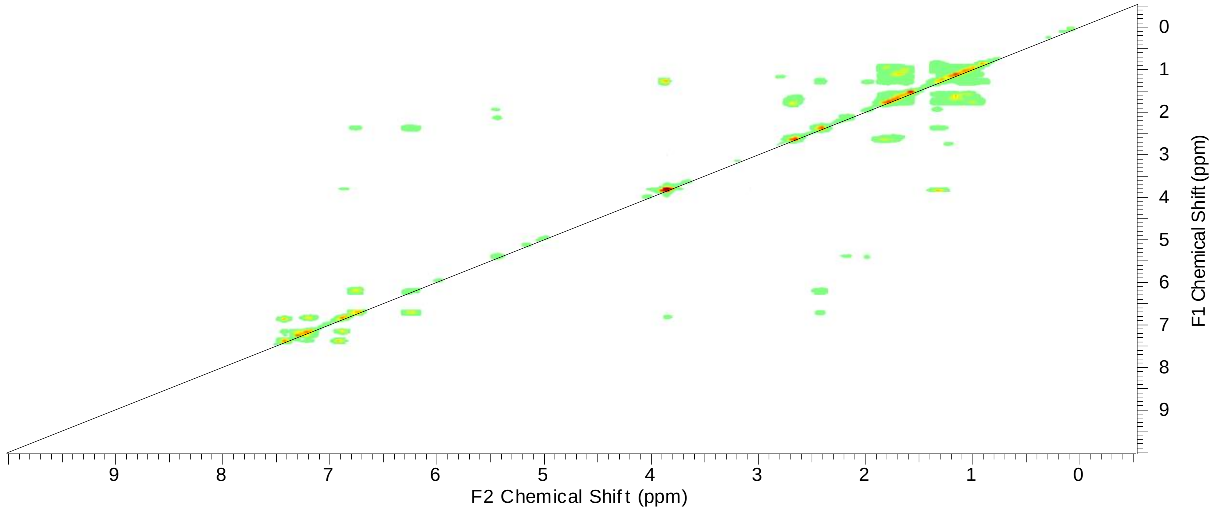

$^1H,^{13}C$-HSQC

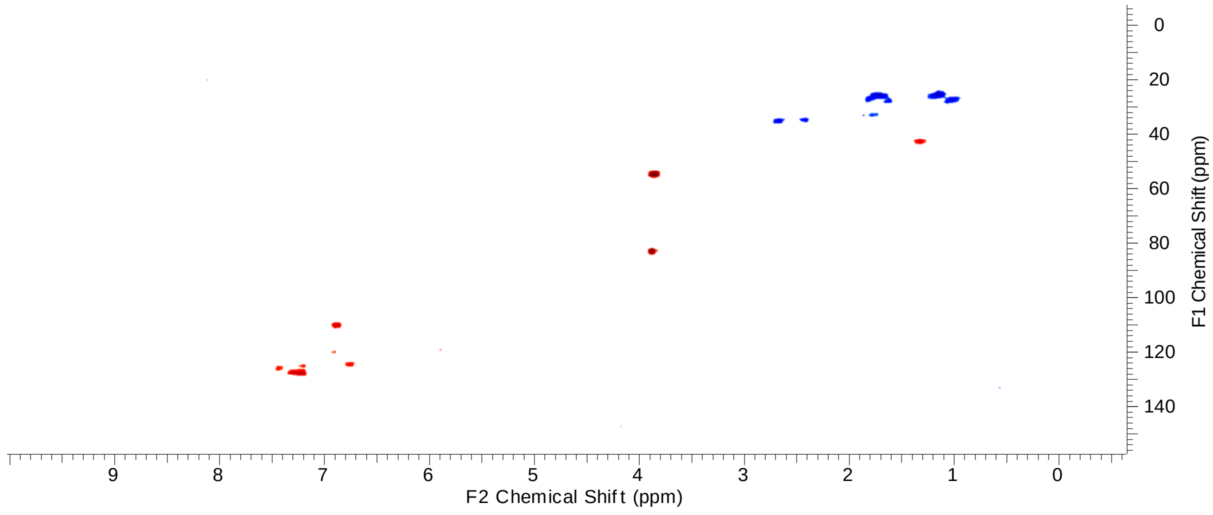

$^1H,^{13}C$-HMBC

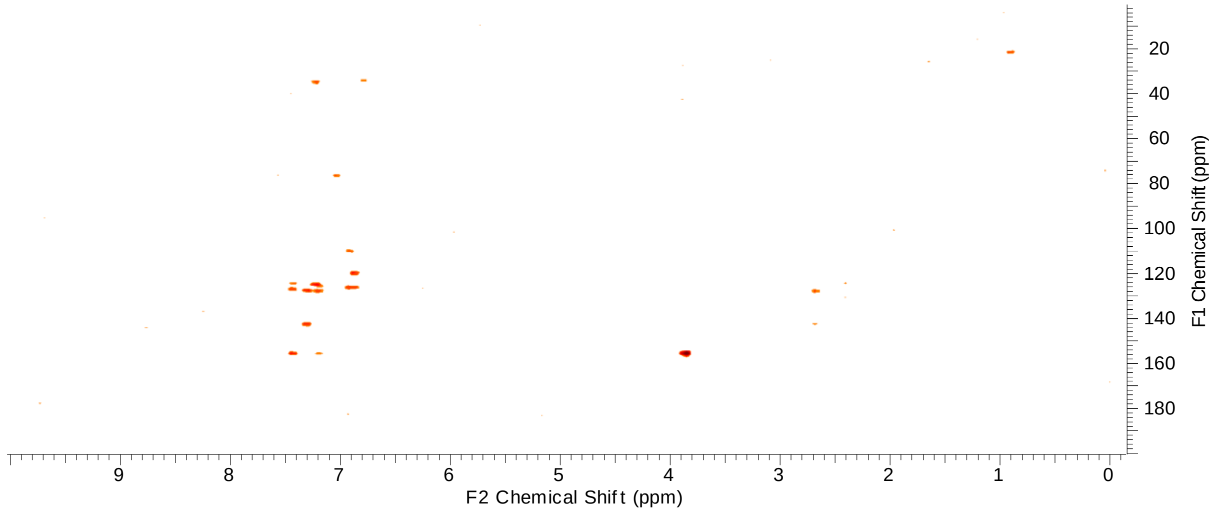

(4R,5R)-4,5-Dicyclohexyl-2-((R,E)-6-(4-fluorophenyl)-1-phenylhex-5-en-3-yl)-1,3,2-dioxaborolane (6g)

^{1}H-NMR (400 MHz, $CDCl_3$):

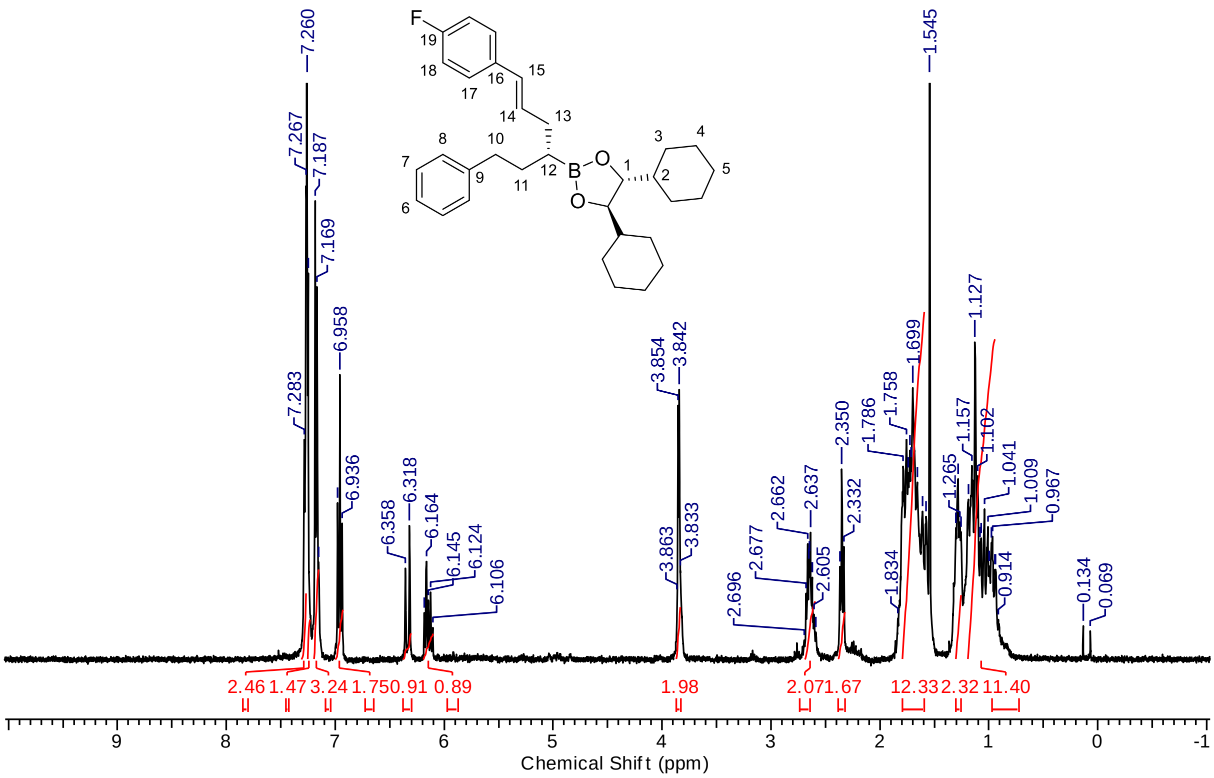

^{13}C-NMR (100 MHz, $CDCl_3$):

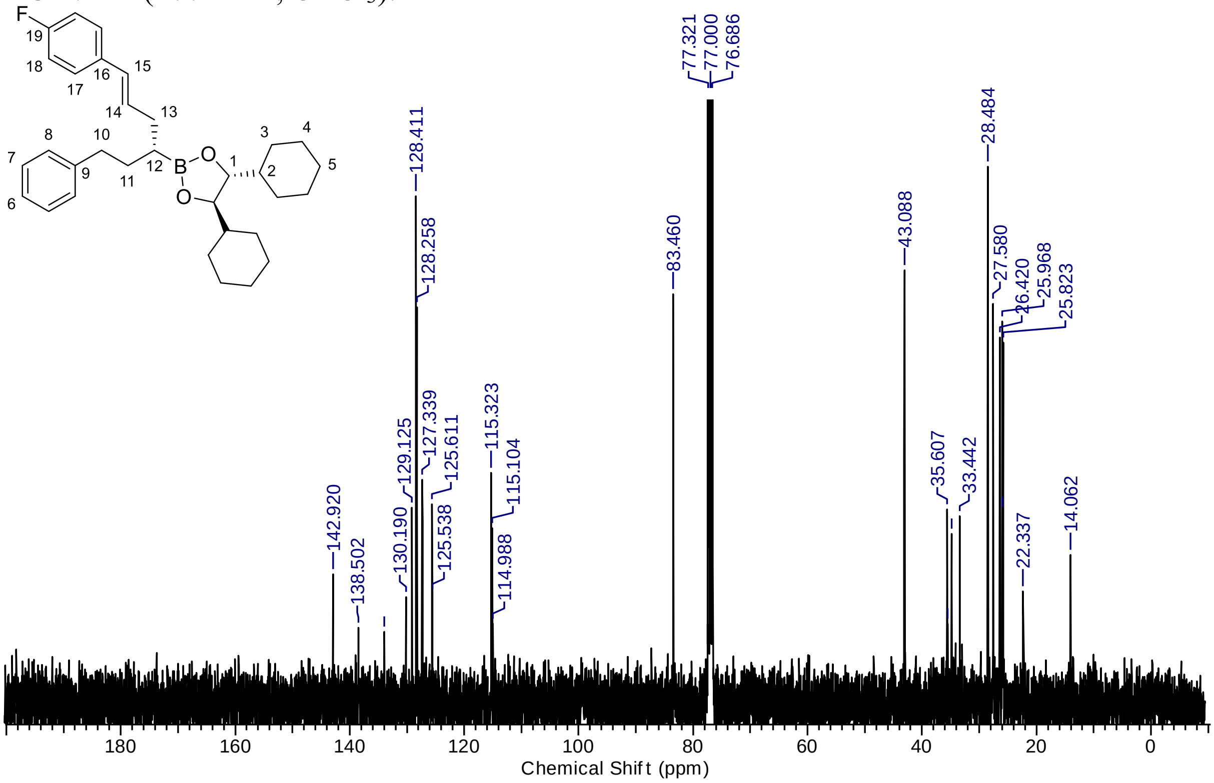

^{19}F-NMR (377 MHz, $CDCl_3$):

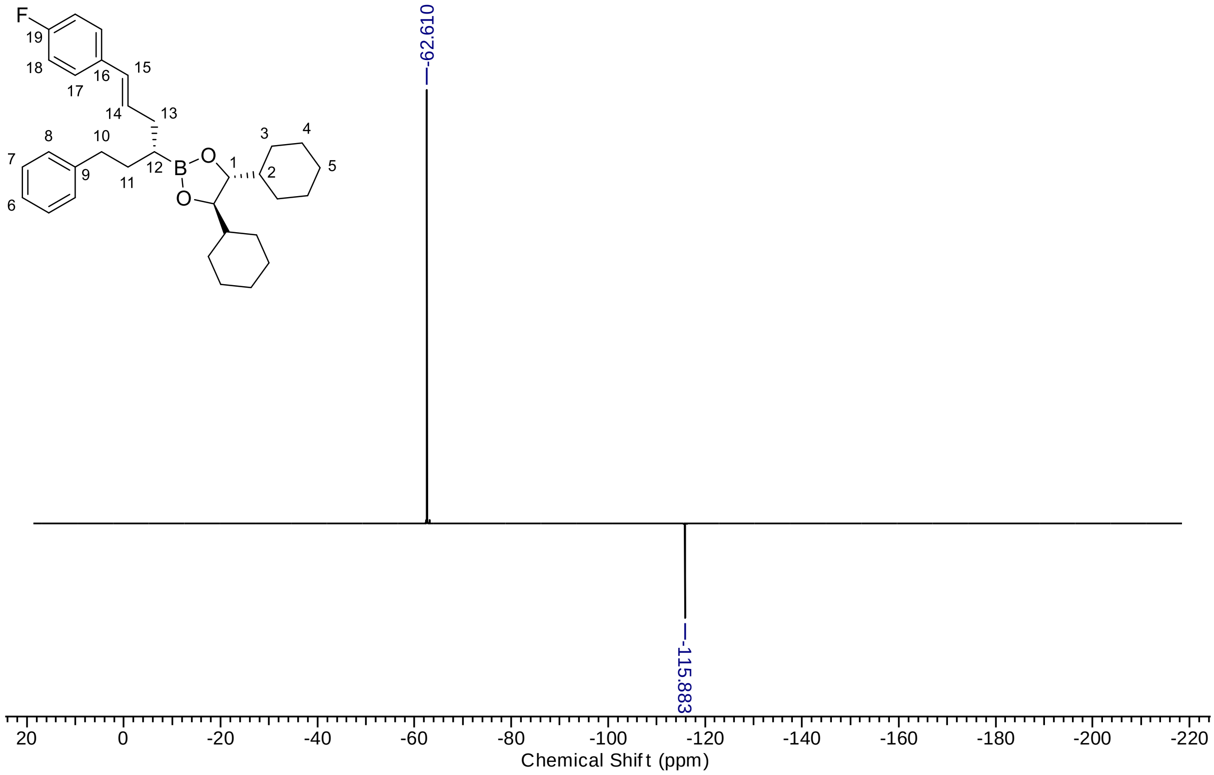

^{1}H,^{1}H-COSY

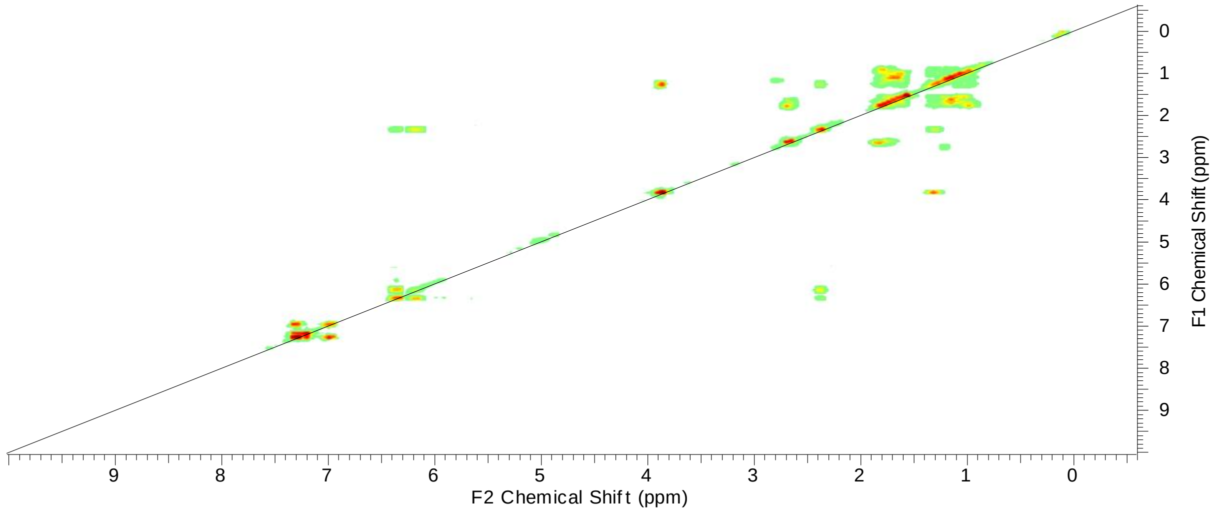

$^{1}H,^{13}C$-HSQC

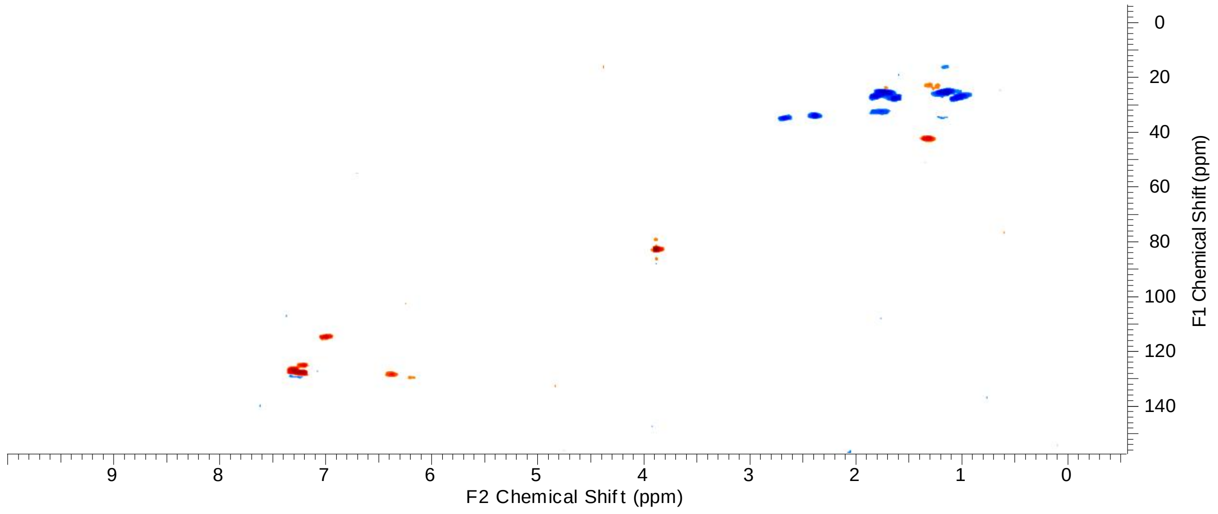

$^{1}H,^{13}C$-HMBC

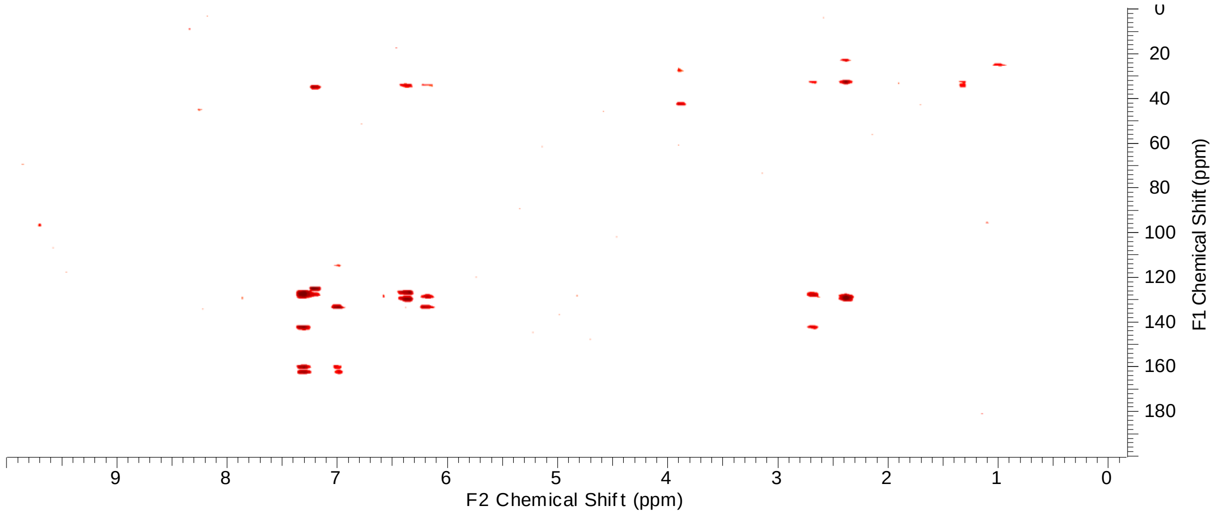

(4*R*,5*R*)-4,5-Dicyclohexyl-2-((*R*,*E*)-1-phenyl-6-(4-(trifluoromethyl)phenyl)hex-5-en-3-yl)-1,3,2-dioxaborolane (6h)

^{1}H-NMR (400 MHz, $CDCl_3$):

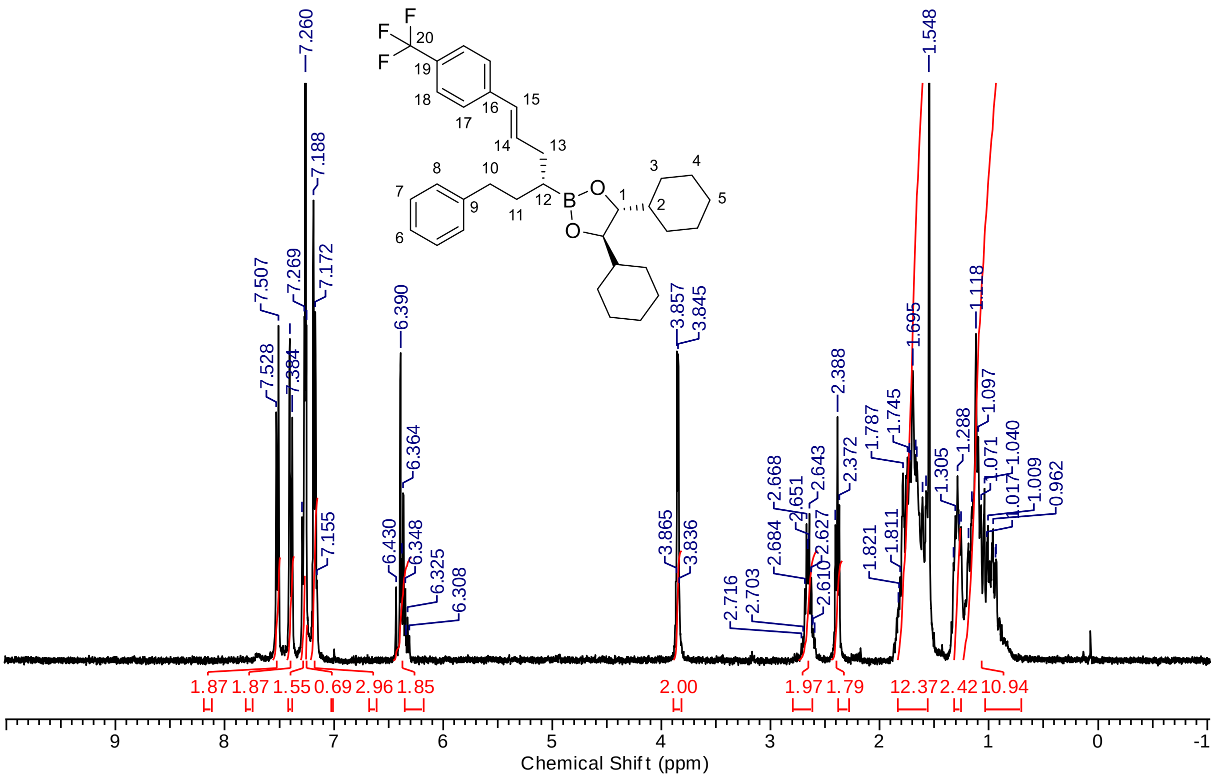

^{13}C-NMR (100 MHz, $CDCl_3$):

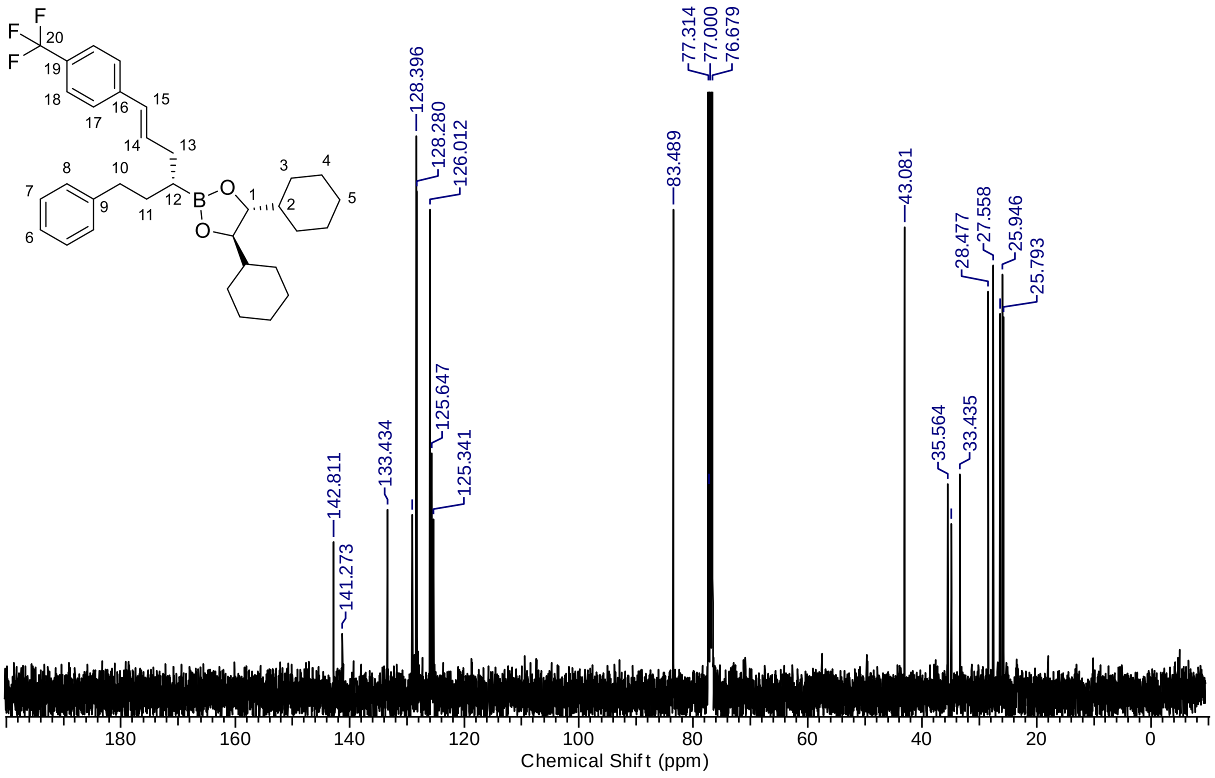

^{19}F-NMR (377 MHz, $CDCl_3$):

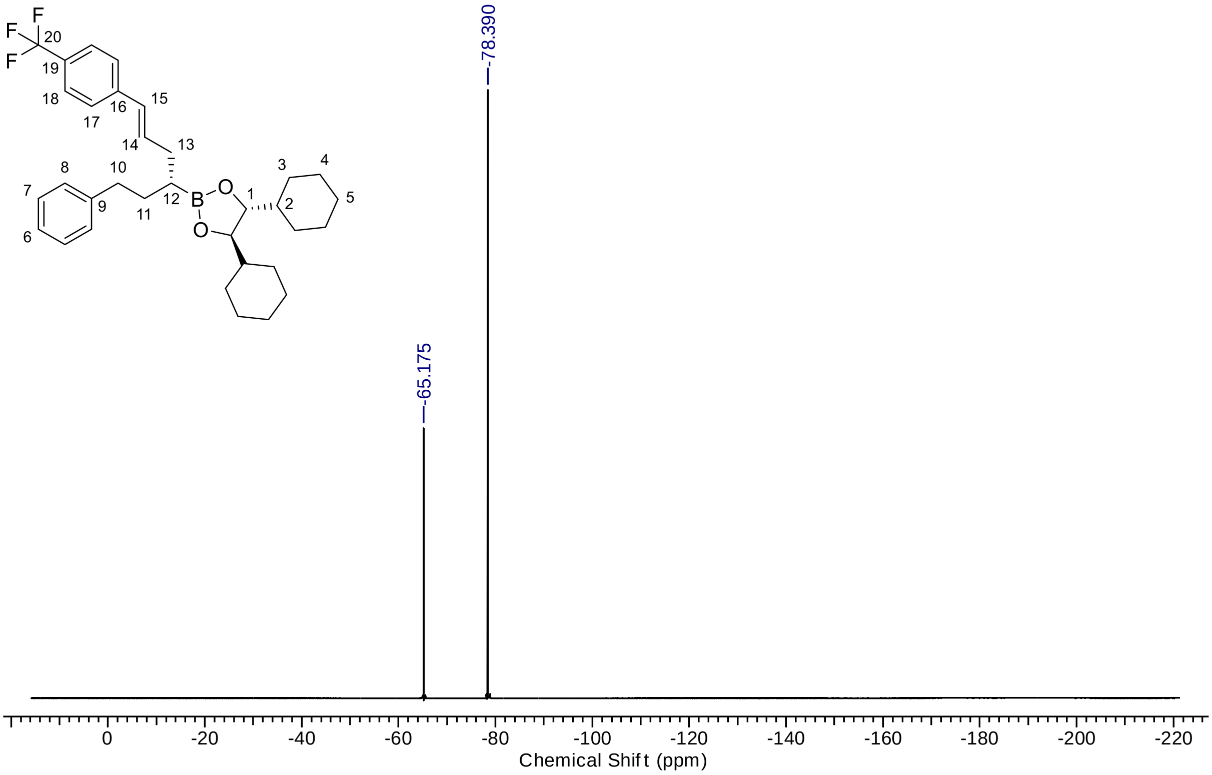

^{1}H,^{1}H-COSY

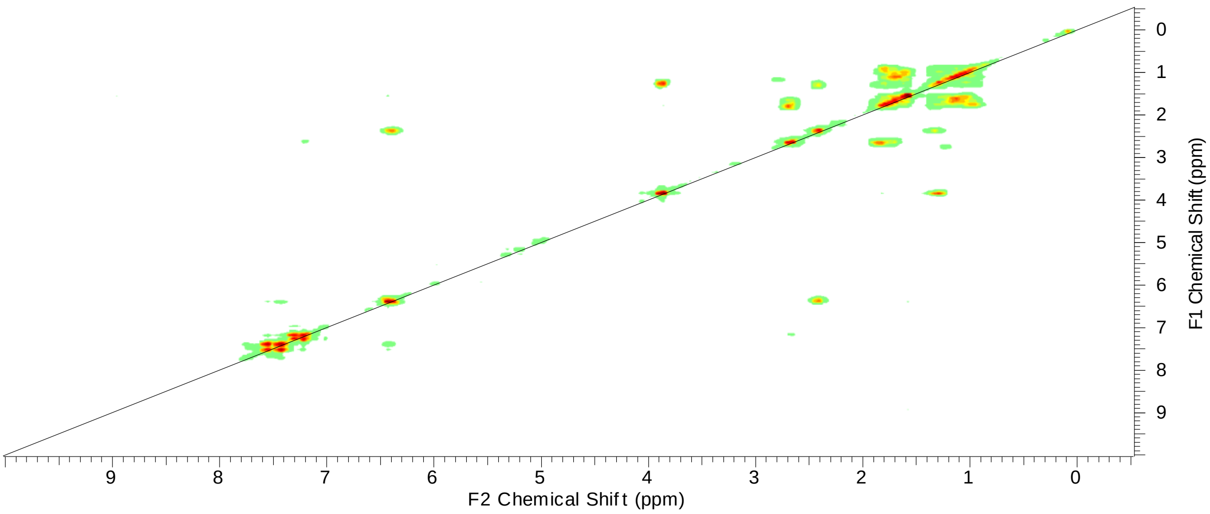

$^{1}H,^{13}C$-HSQC

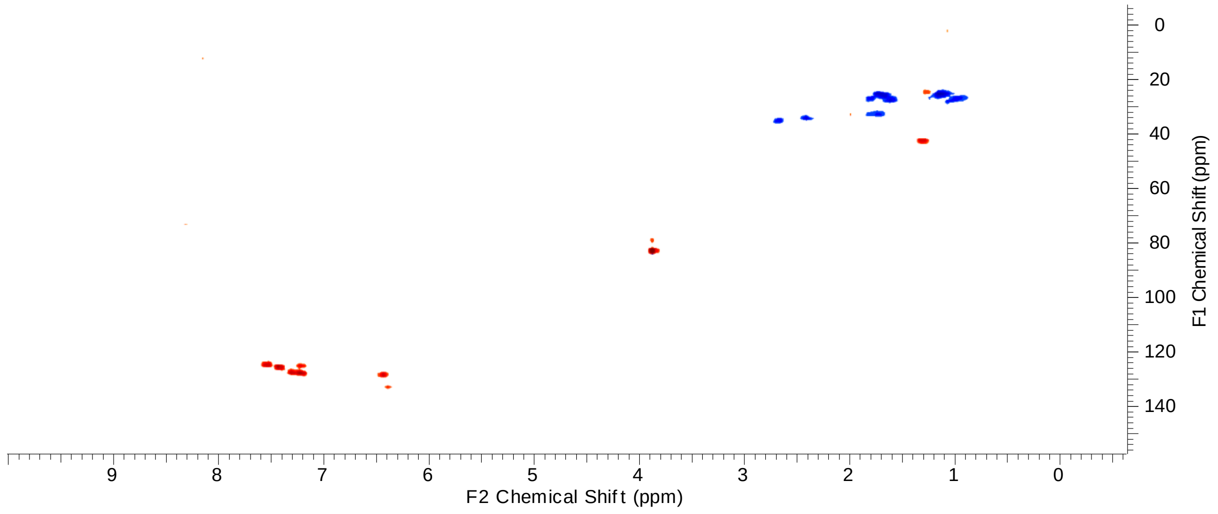

$^{1}H,^{13}C$-HMBC

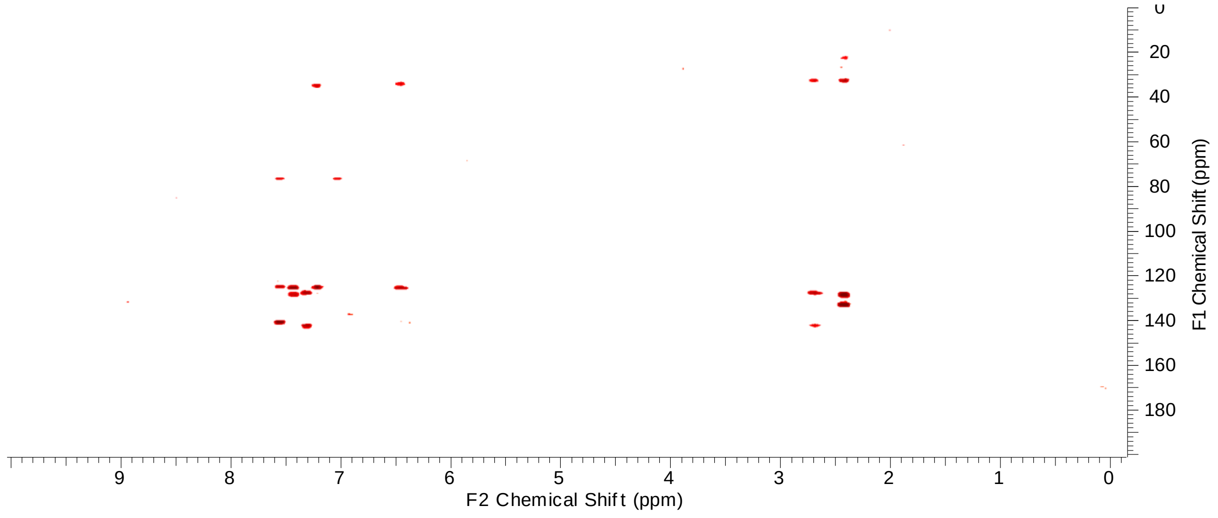

(*R*,*E*)-6-cyclopentyl-1-phenylhex-5-en-3-ol (7i)

^{1}H-NMR (400 MHz, $CDCl_3$):

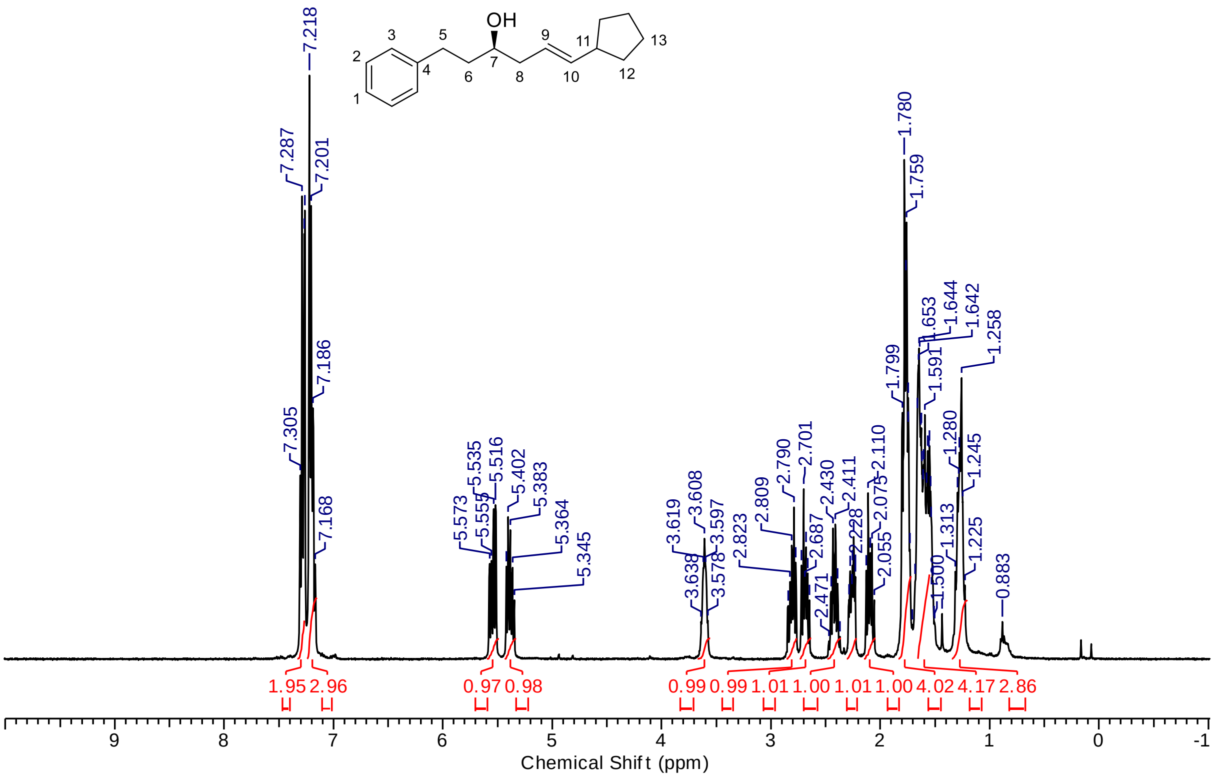

^{13}C-NMR (100 MHz, $CDCl_3$):

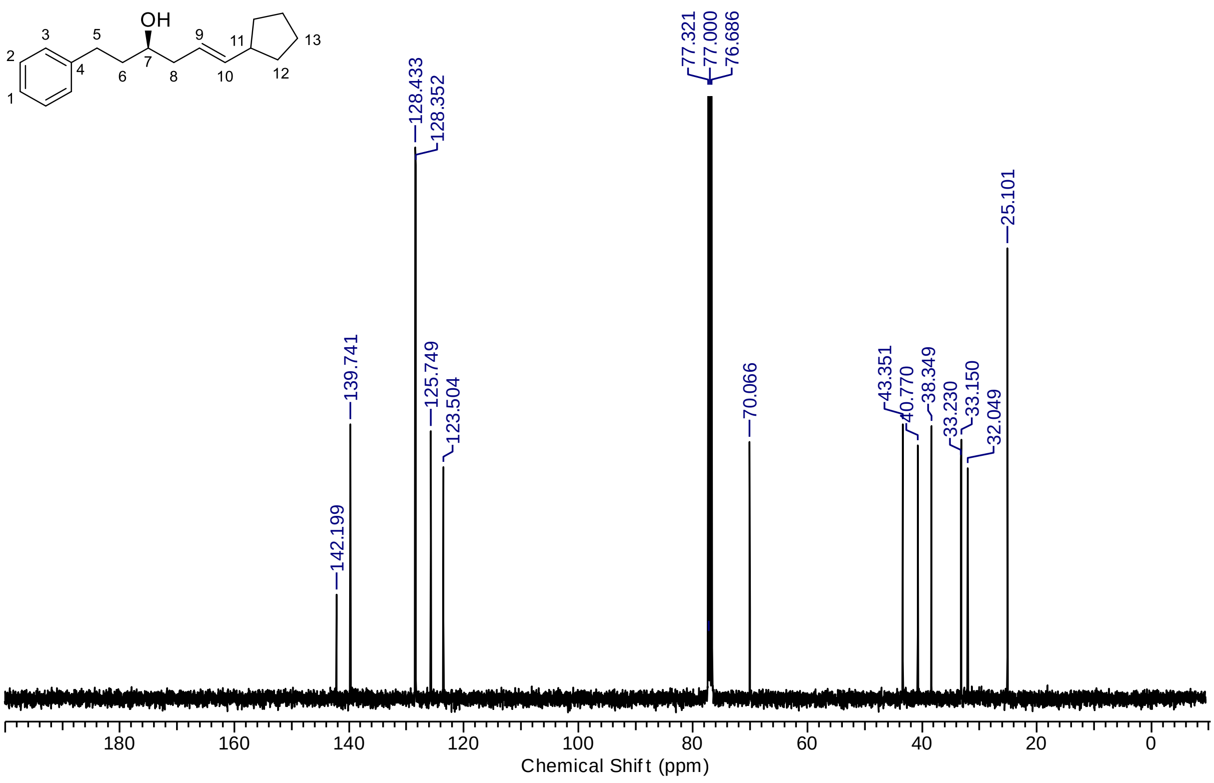

$^{1}H,^{1}H$-COSY

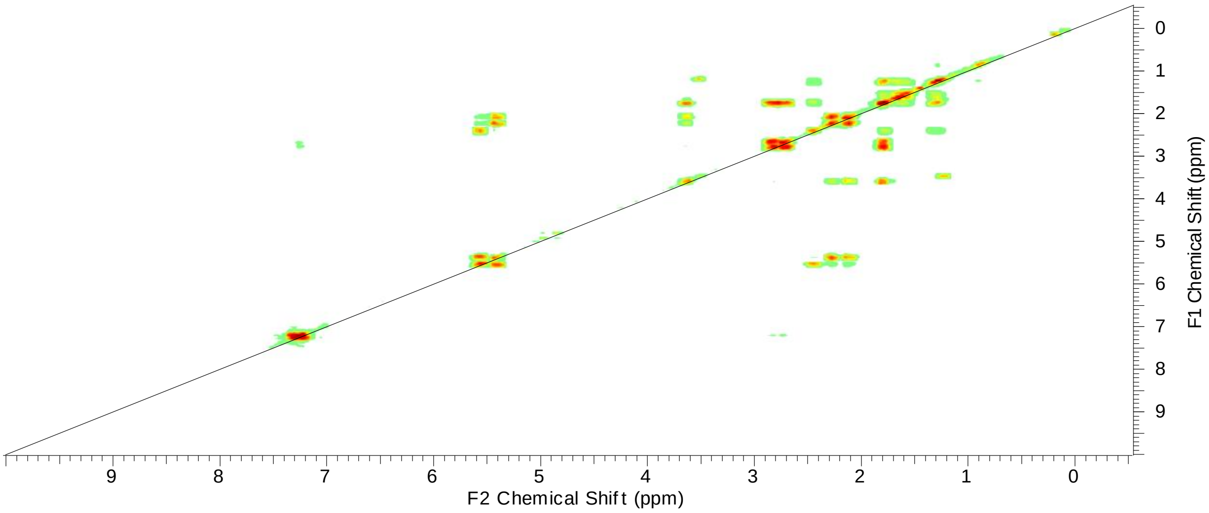

$^{1}H,^{13}C$-HSQC

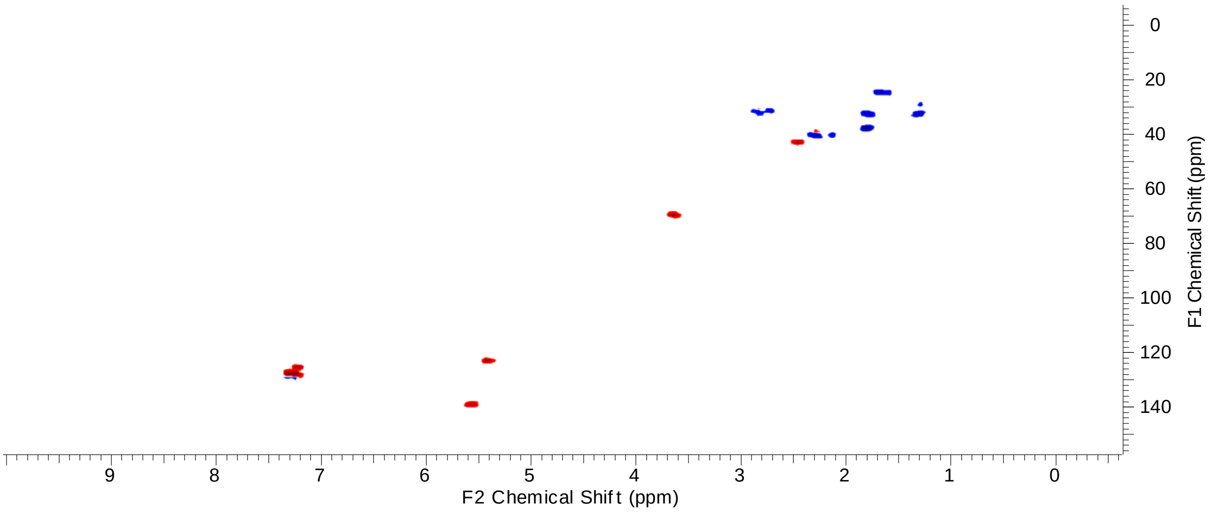

$^{1}H,^{13}C$-HMBC

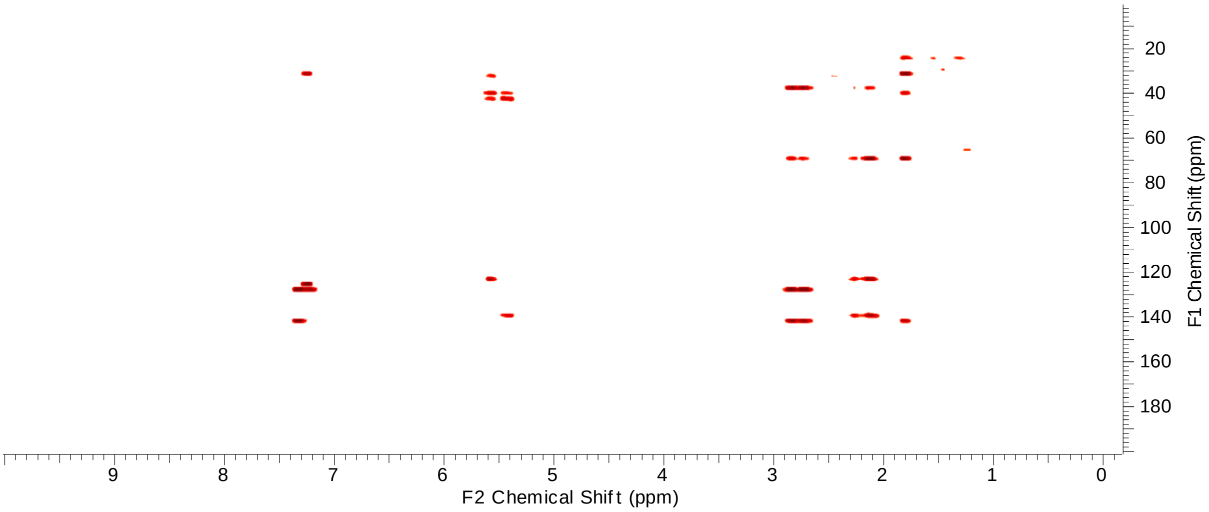

(4*R*,5*R*)-4,5-Dicyclohexyl-2-((*R*,*E*)-6-(furan-2-yl)-1-phenylhex-5-en-3-yl)-1,3,2-dioxaborolane (6j)

^{1}H-NMR (400 MHz, $CDCl_3$):

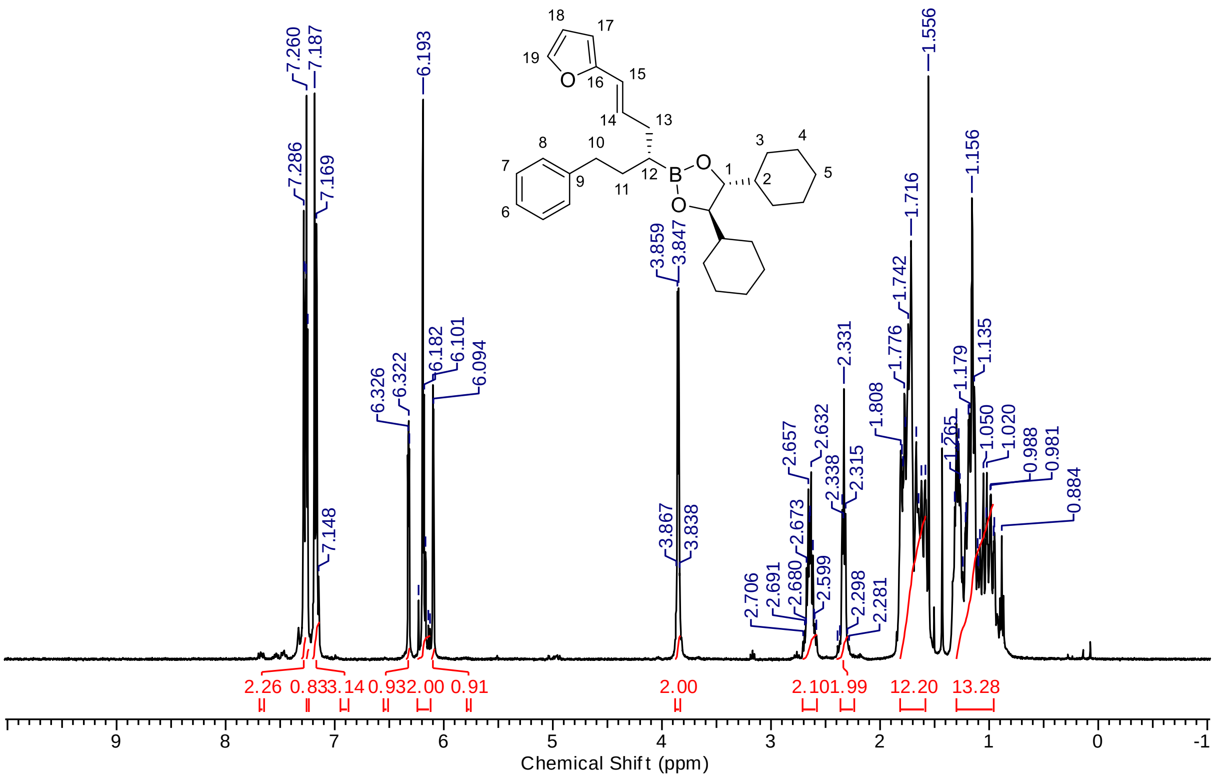

^{13}C-NMR (100 MHz, $CDCl_3$):

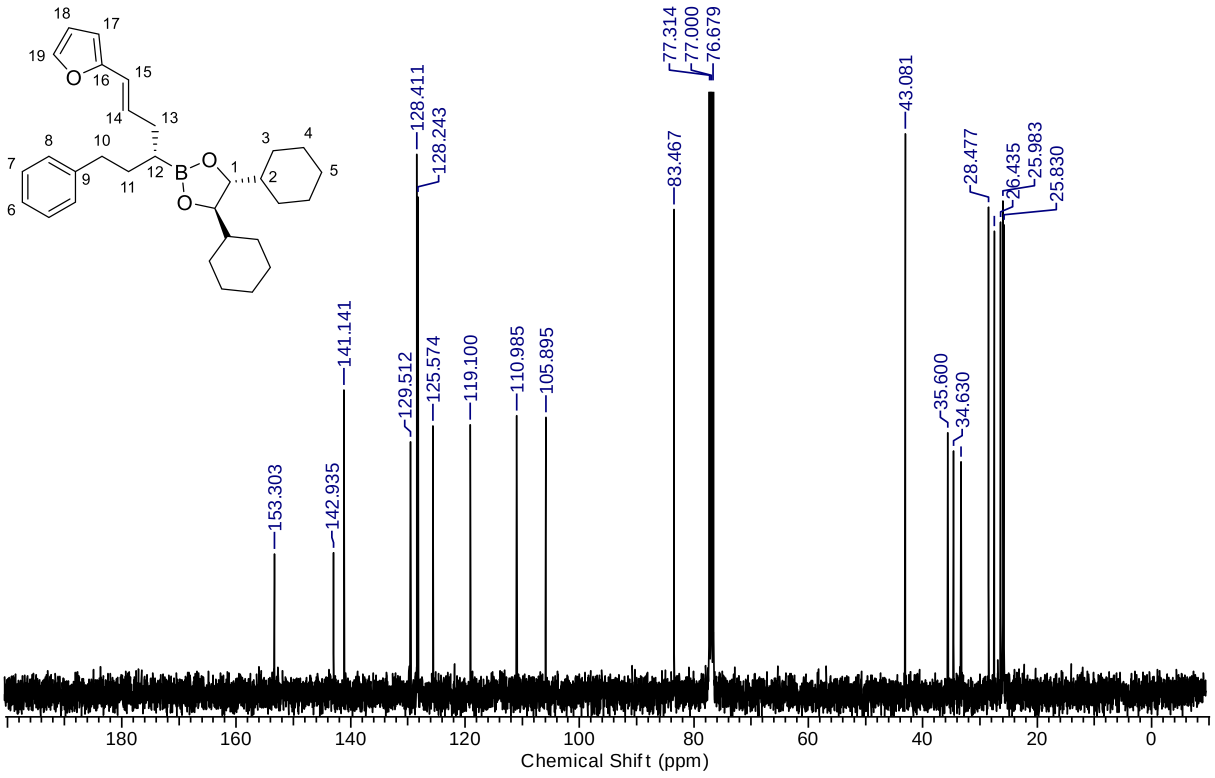

$^{1}H,^{1}H$-COSY

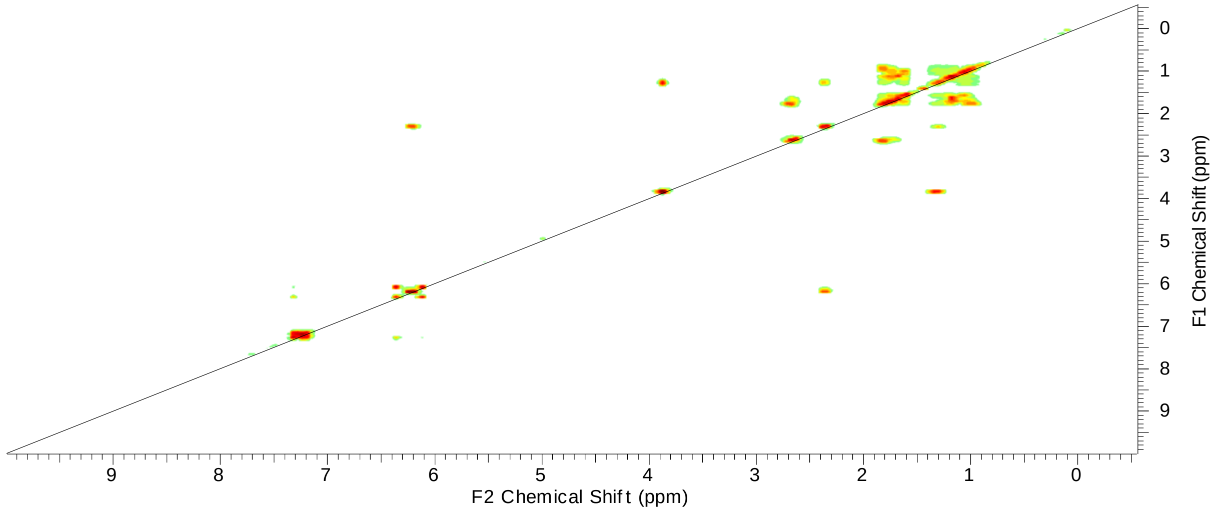

$^{1}H,^{13}C$-HSQC

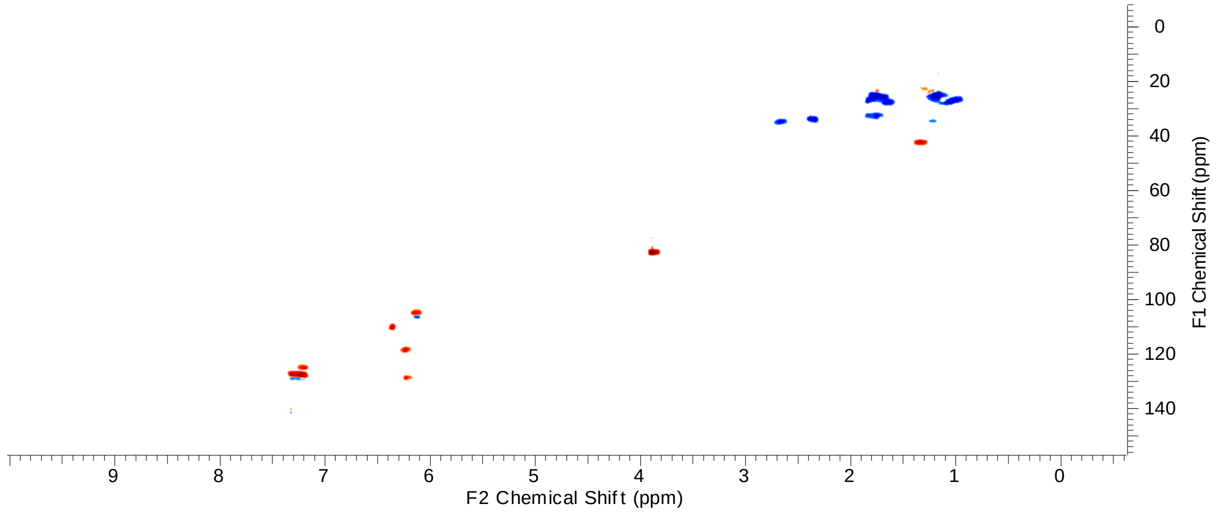

$^{1}H,^{13}C$-HMBC

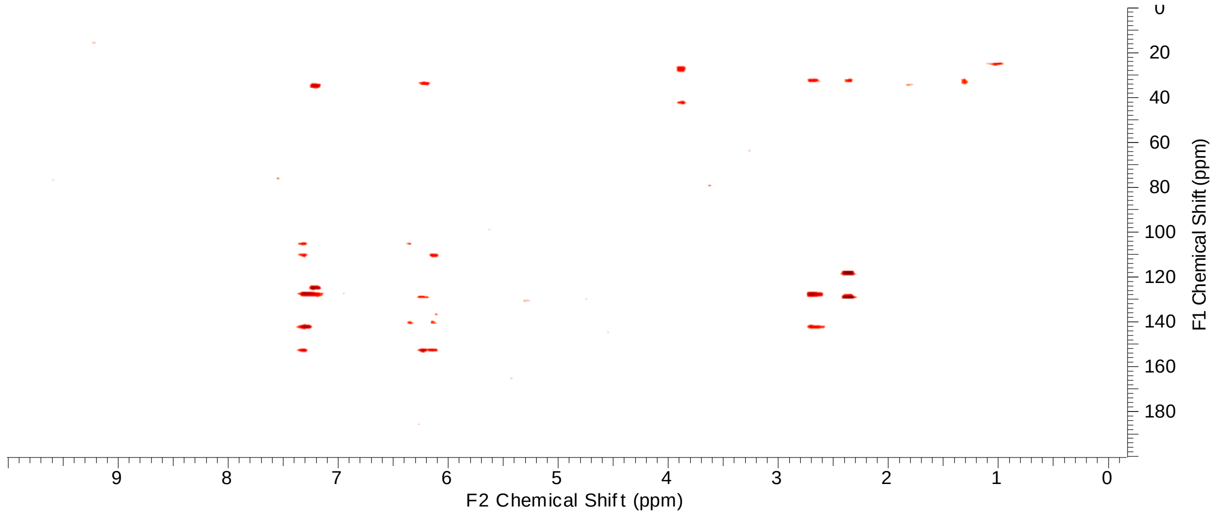

2-((*R*,*E*)-4-((4*R*,5*R*)-4,5-Dicyclohexyl-1,3,2-dioxaborolan-2-yl)-6-phenylhex-1-en-1-yl)-1-methyl-1*H*-indole (6k)

^{1}H-NMR (400 MHz, CDCl$_3$):

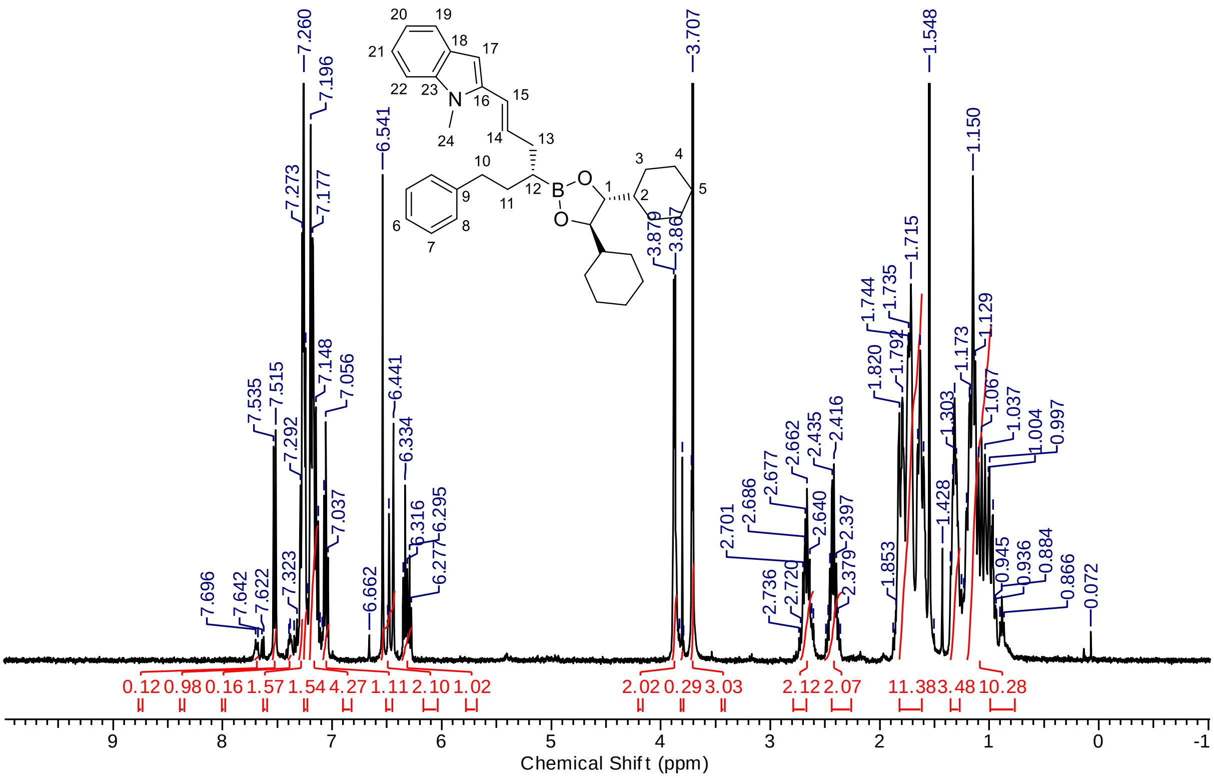

Additional signals arising from methyl-1*H*-indole

^{13}C-NMR (100 MHz, CDCl$_3$):

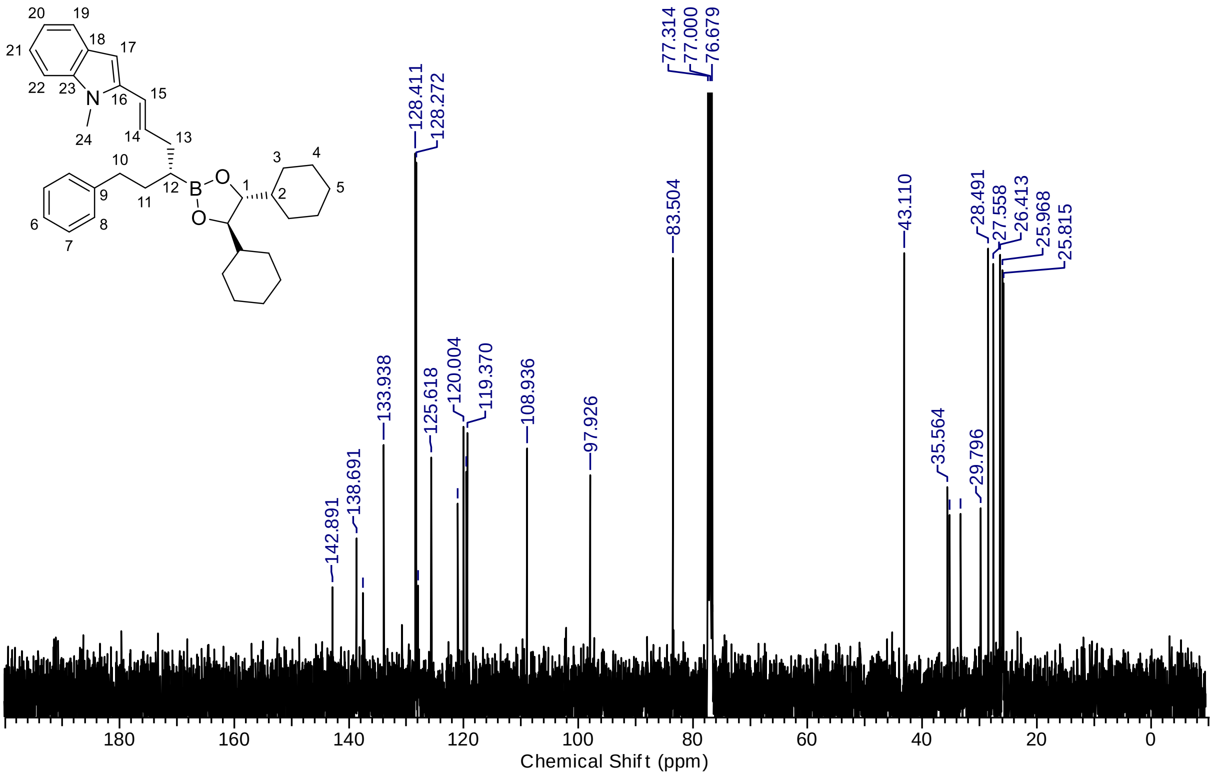

Additional signals arising from methyl-1*H*-indole

$^{1}H,^{1}H$-COSY

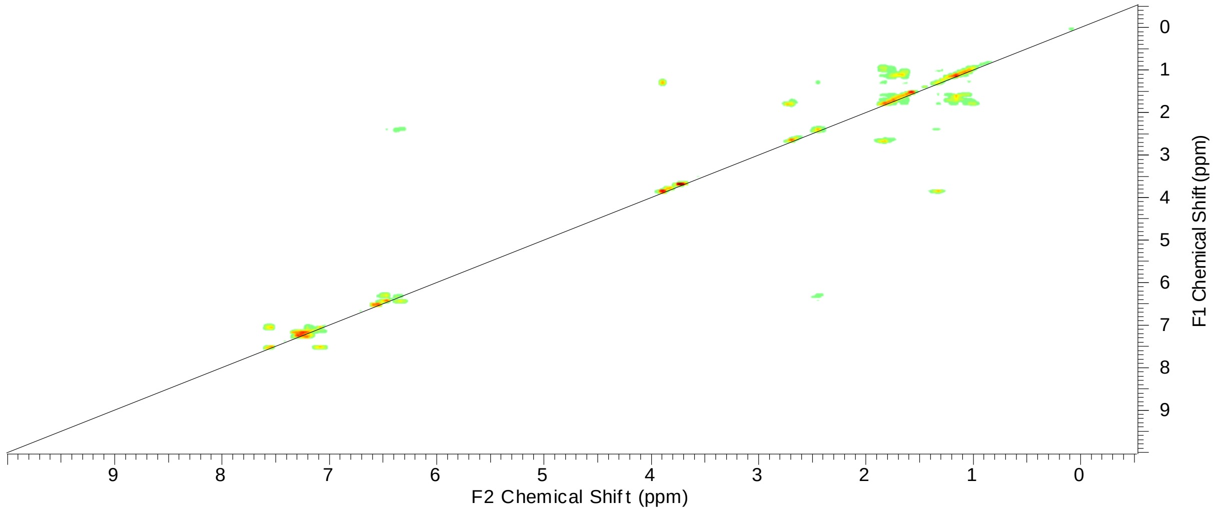

$^{1}H,^{13}C$-HSQC

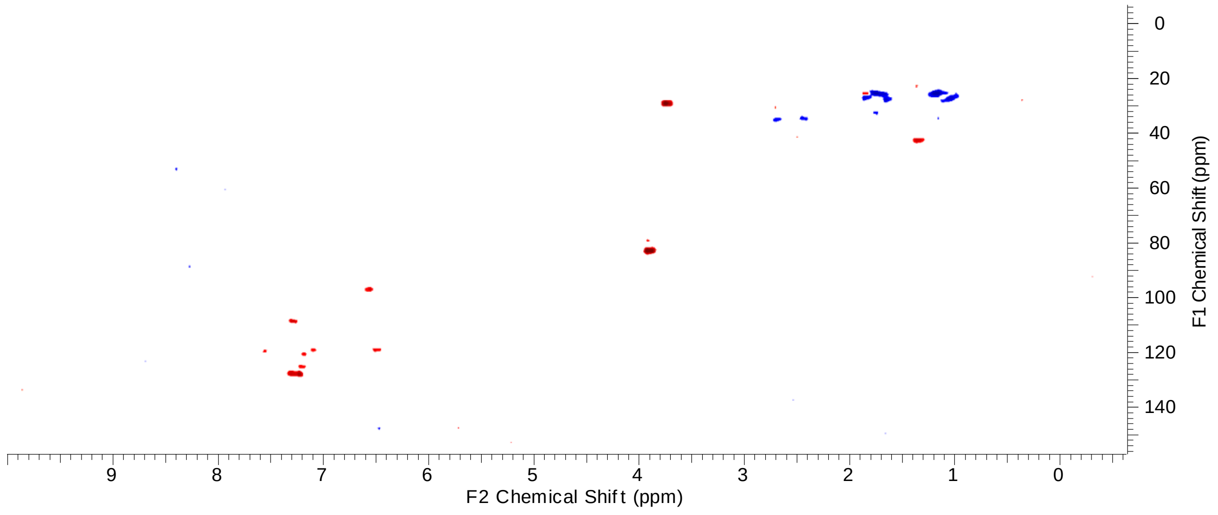

$^{1}H,^{13}C$-HMBC

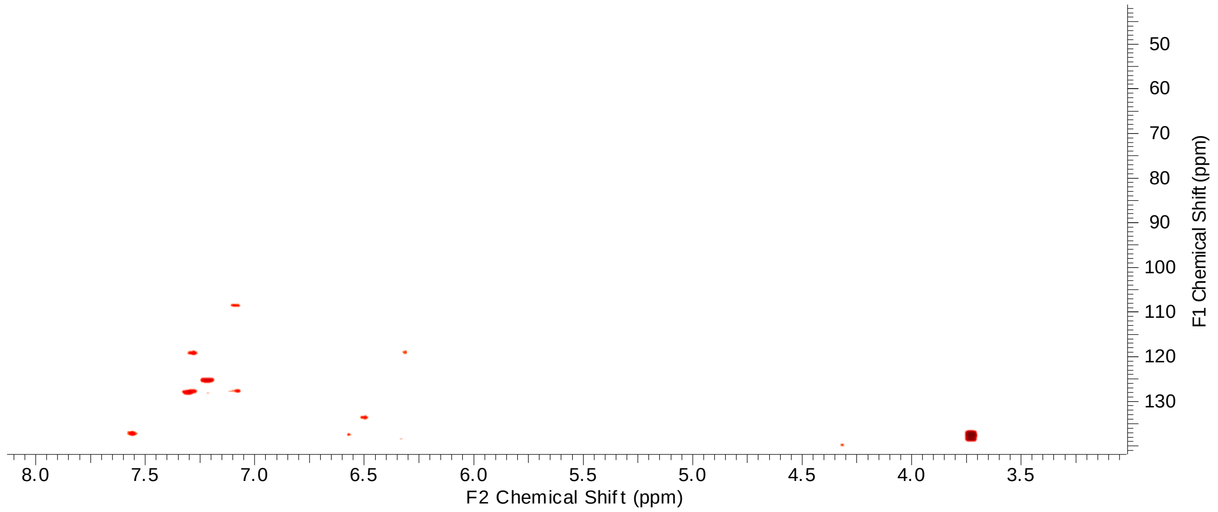

2-((*R*,*E*)-4-((4*R*,5*R*)-4,5-Dicyclohexyl-1,3,2-dioxaborolan-2-yl)-6-phenylhex-1-en-1-yl)-pyridine (6l)

^{1}H-NMR (400 MHz, $CDCl_3$):

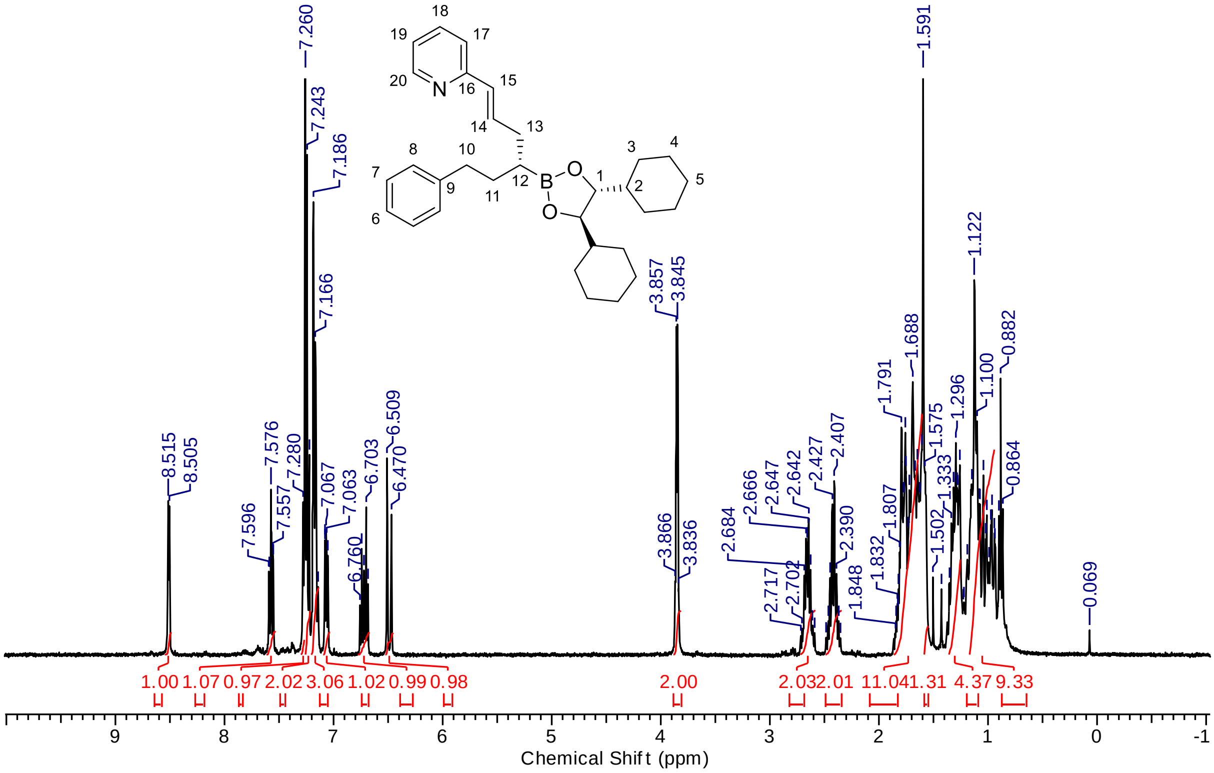

^{13}C-NMR (100 MHz, $CDCl_3$):

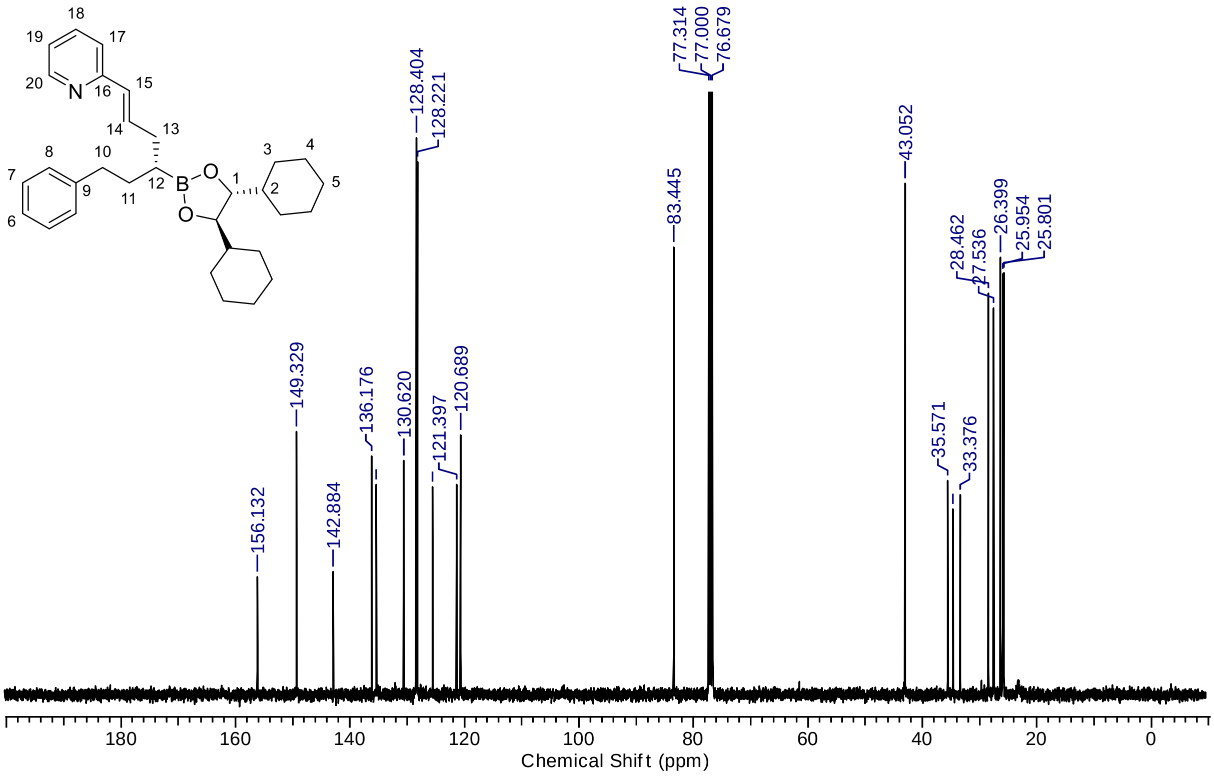

$^1H,^1H$-COSY

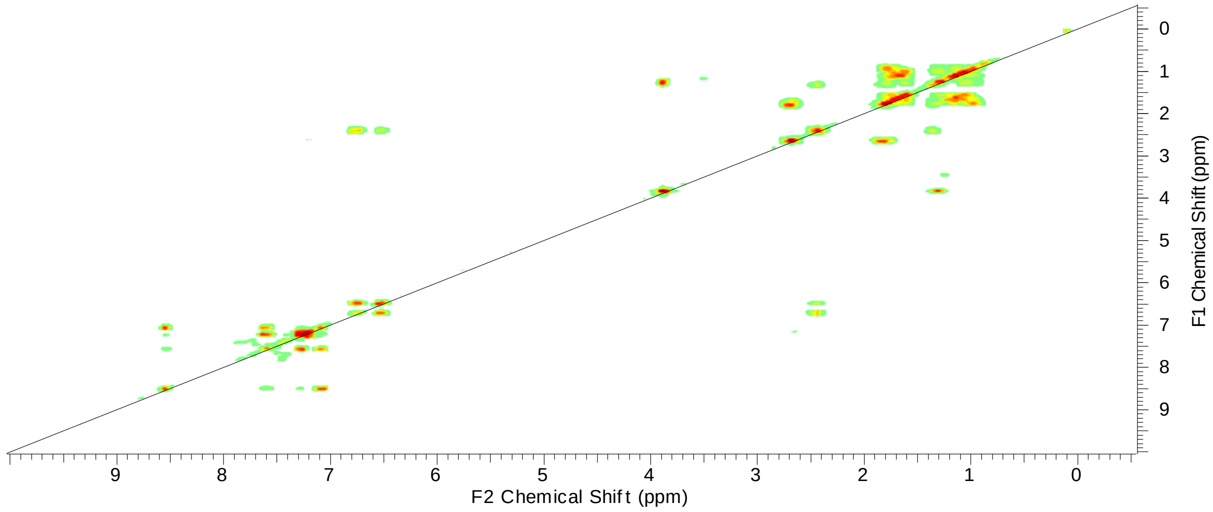

$^1H,^{13}C$-HSQC

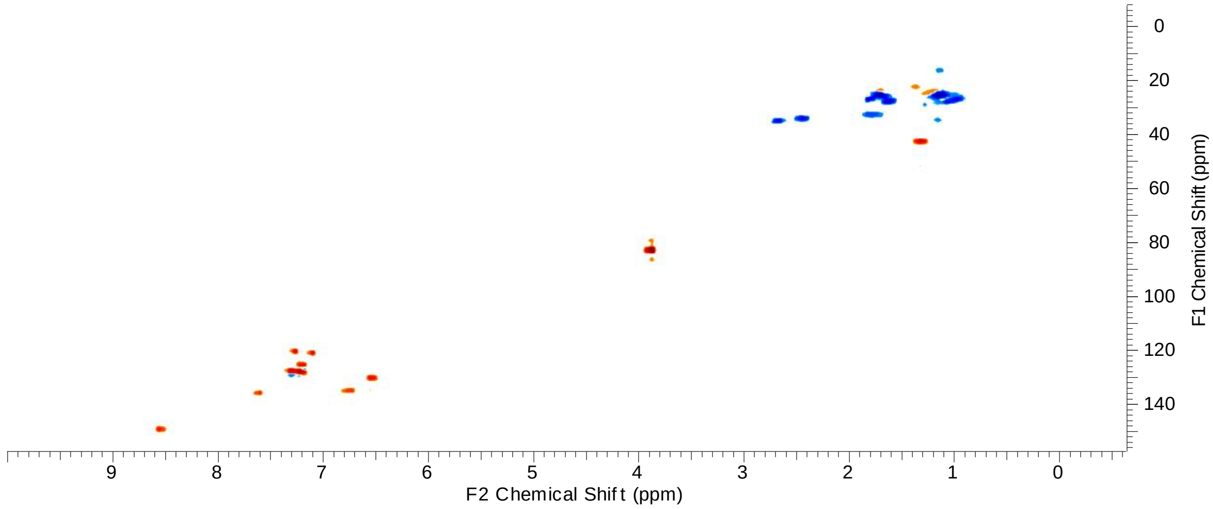

$^1H,^{13}C$-HMBC

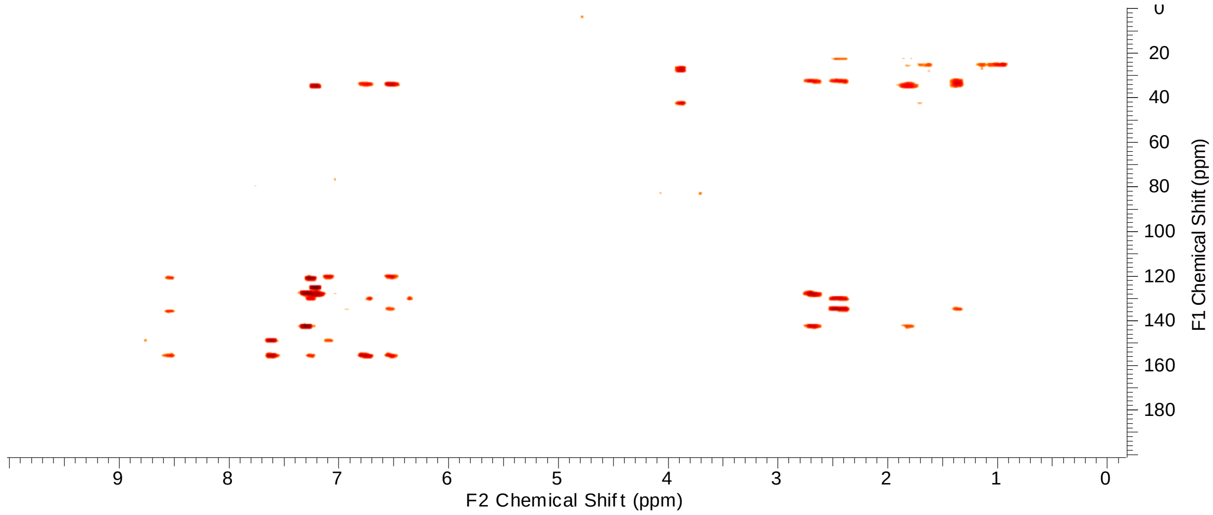

(4*R*,5*R*)-4,5-Dicyclohexyl-2-((*R*,*E*)-1,7-diphenylhept-5-en-3-yl)-1,3,2-dioxaborolane (6m)

^{1}H-NMR (400 MHz, $CDCl_3$):

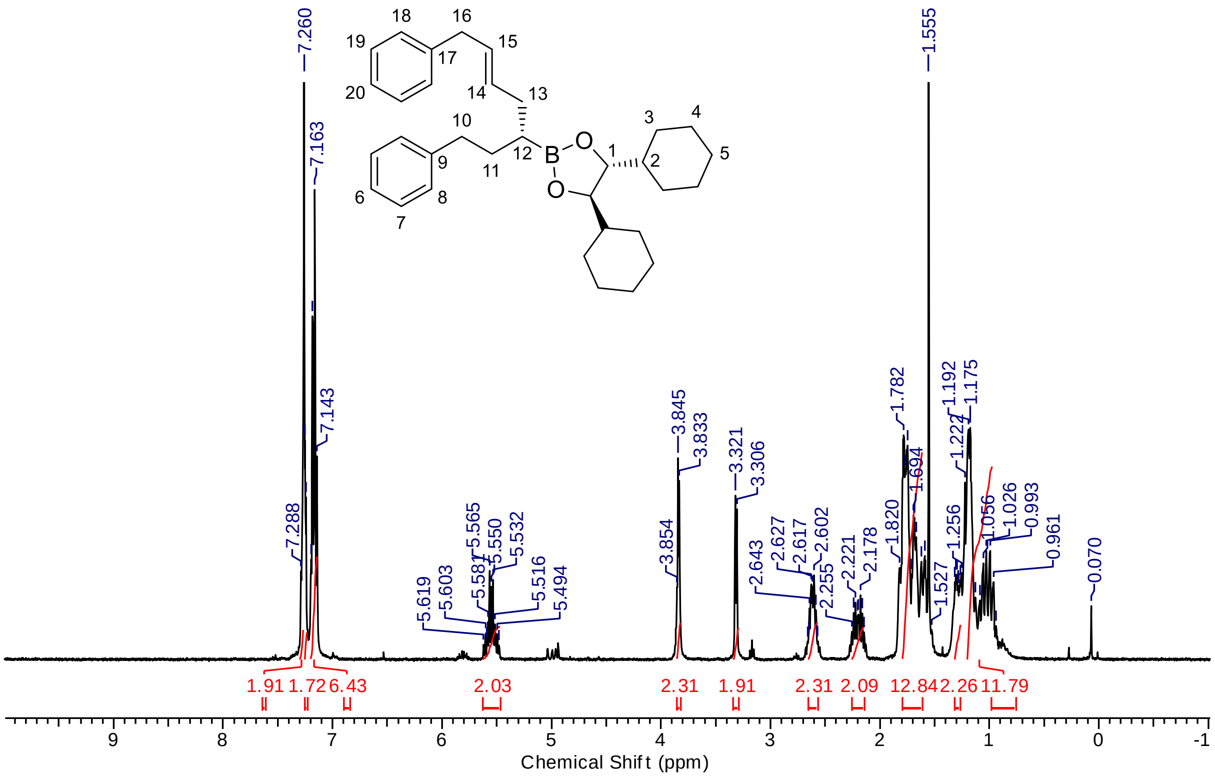

^{13}C-NMR (100 MHz, $CDCl_3$):

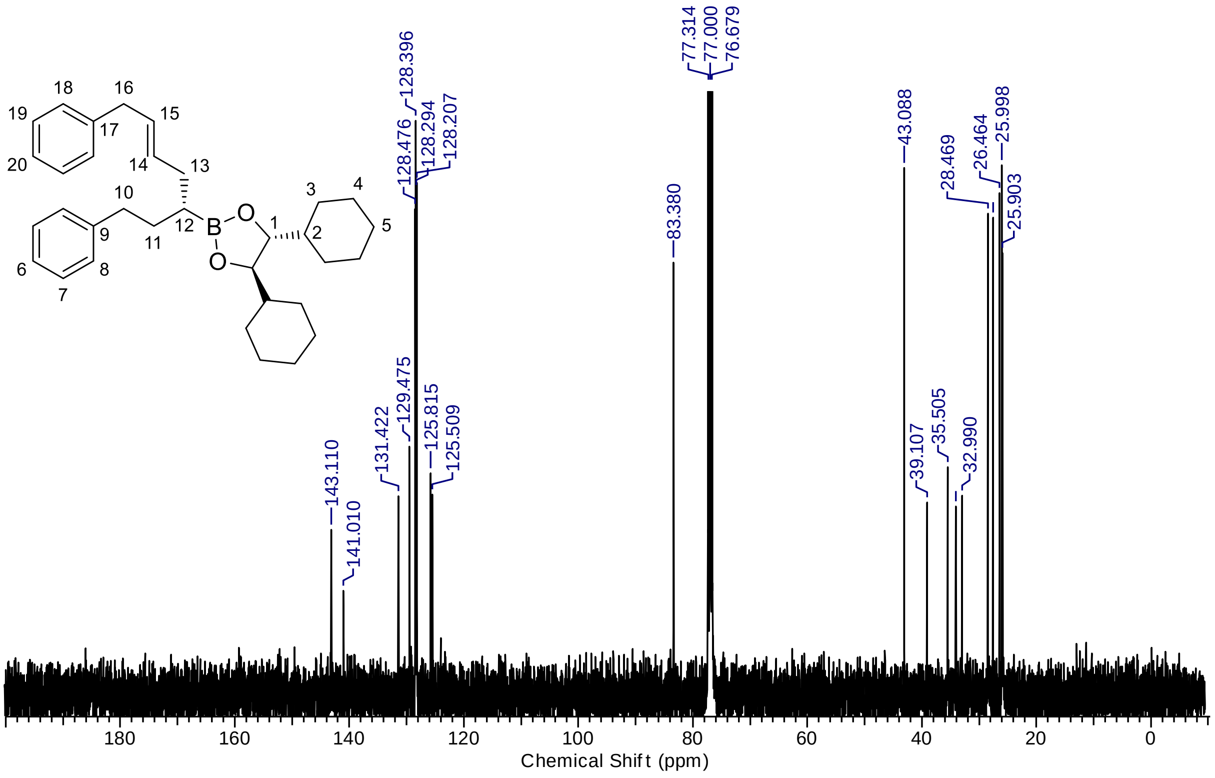

$^{1}H,^{1}H$-COSY

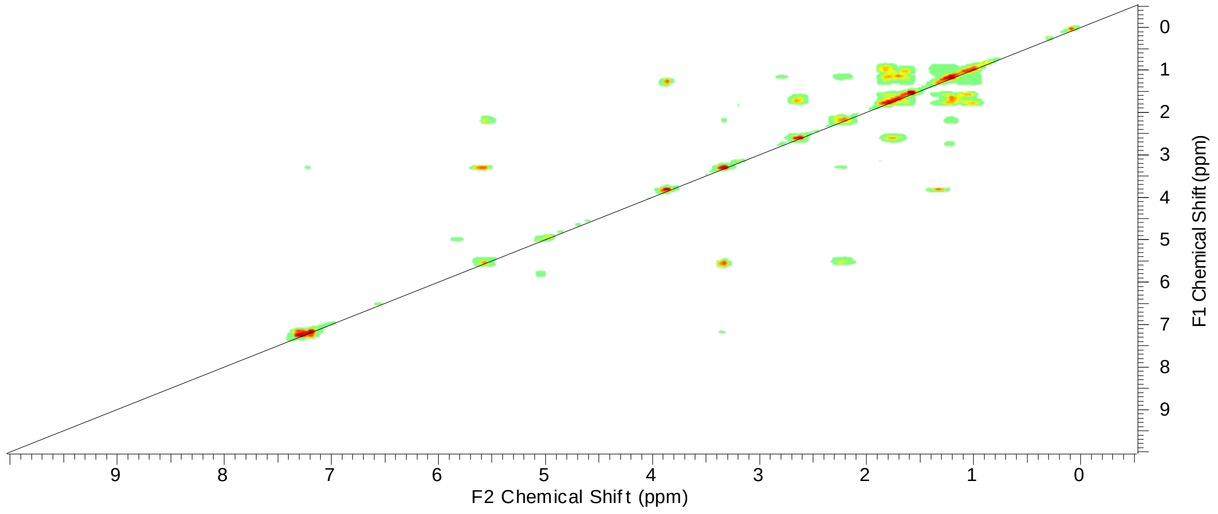

$^{1}H,^{13}C$-HSQC

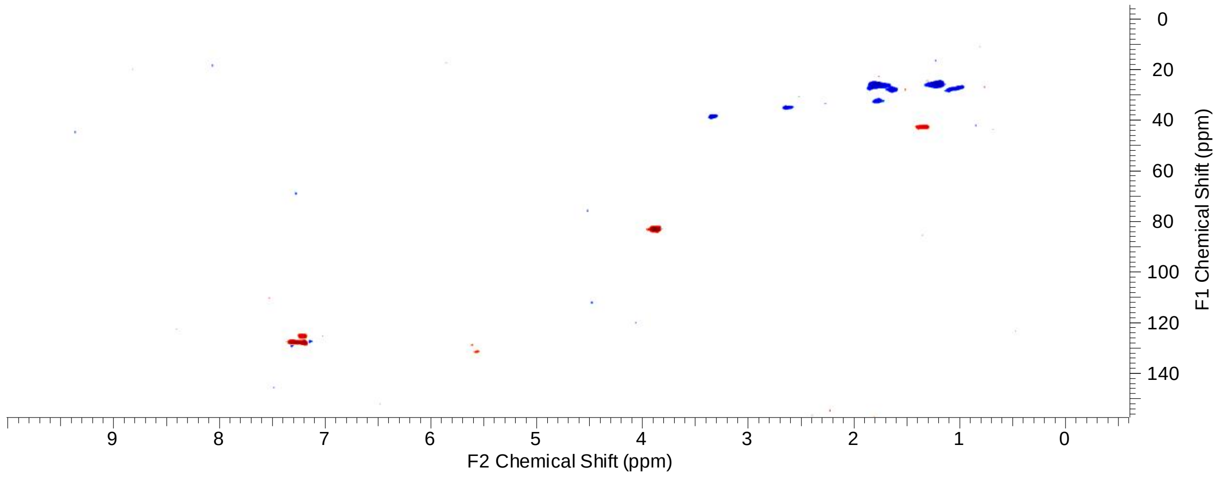

$^{1}H,^{13}C$-HMBC

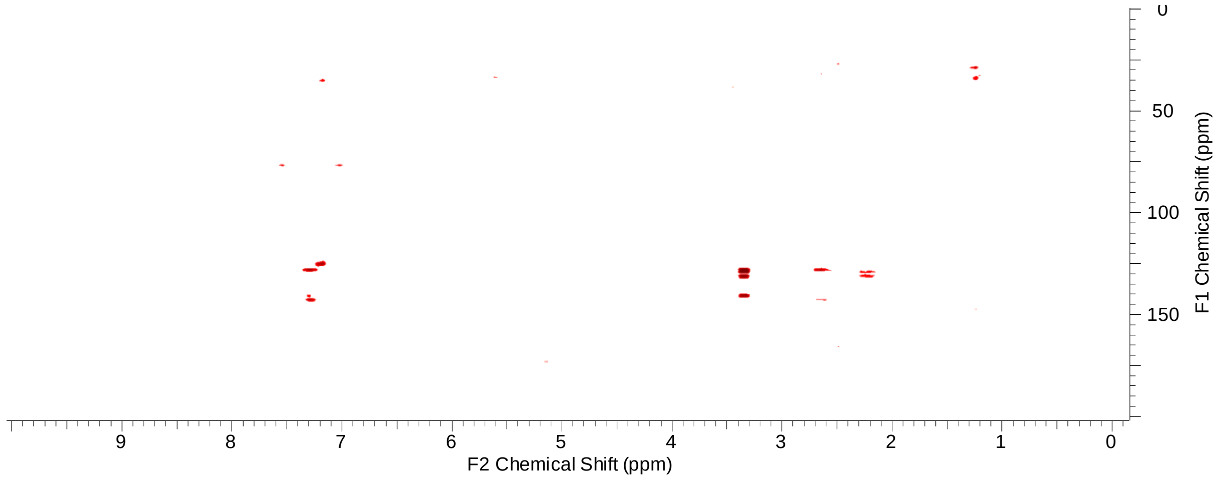

(4*R*,5*R*)-4,5-Dicyclohexyl-2-((*R*,*E*)-1-phenylocta-5,7-dien-3-yl)-1,3,2-dioxaborolane (6n)

^{1}H-NMR (400 MHz, $CDCl_3$):

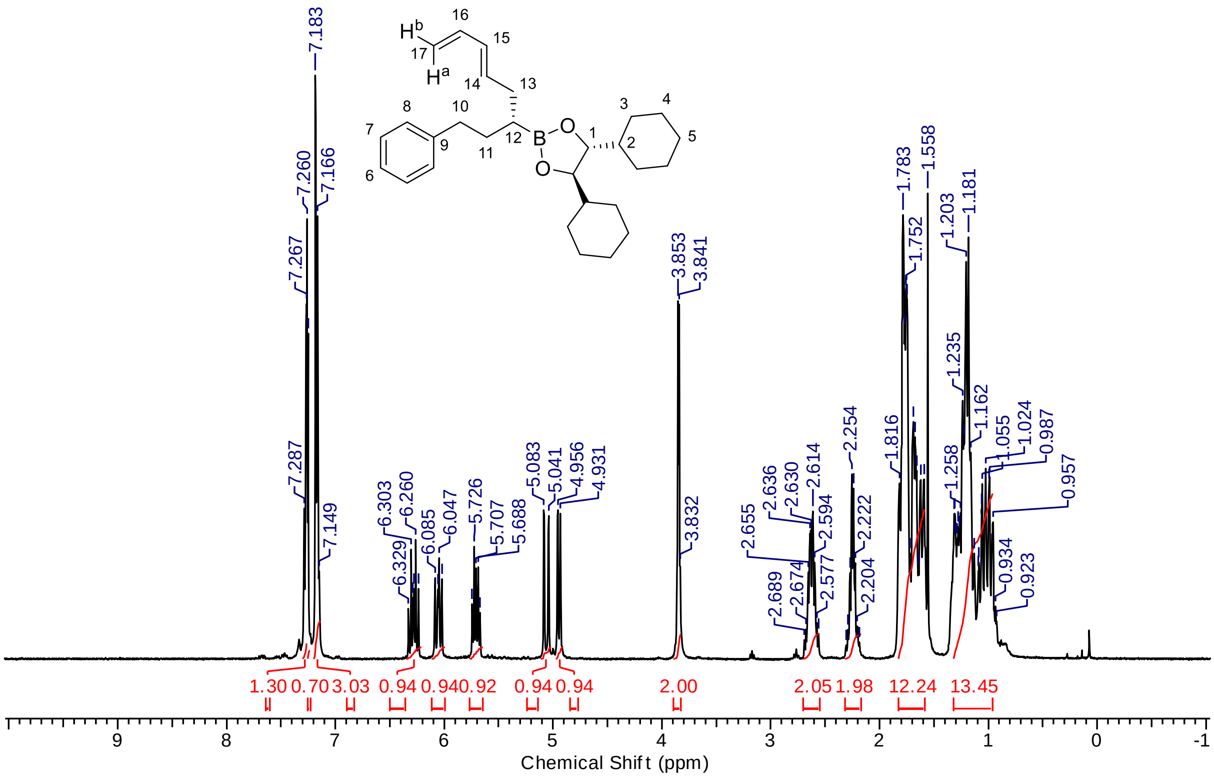

^{13}C-NMR (100 MHz, $CDCl_3$):

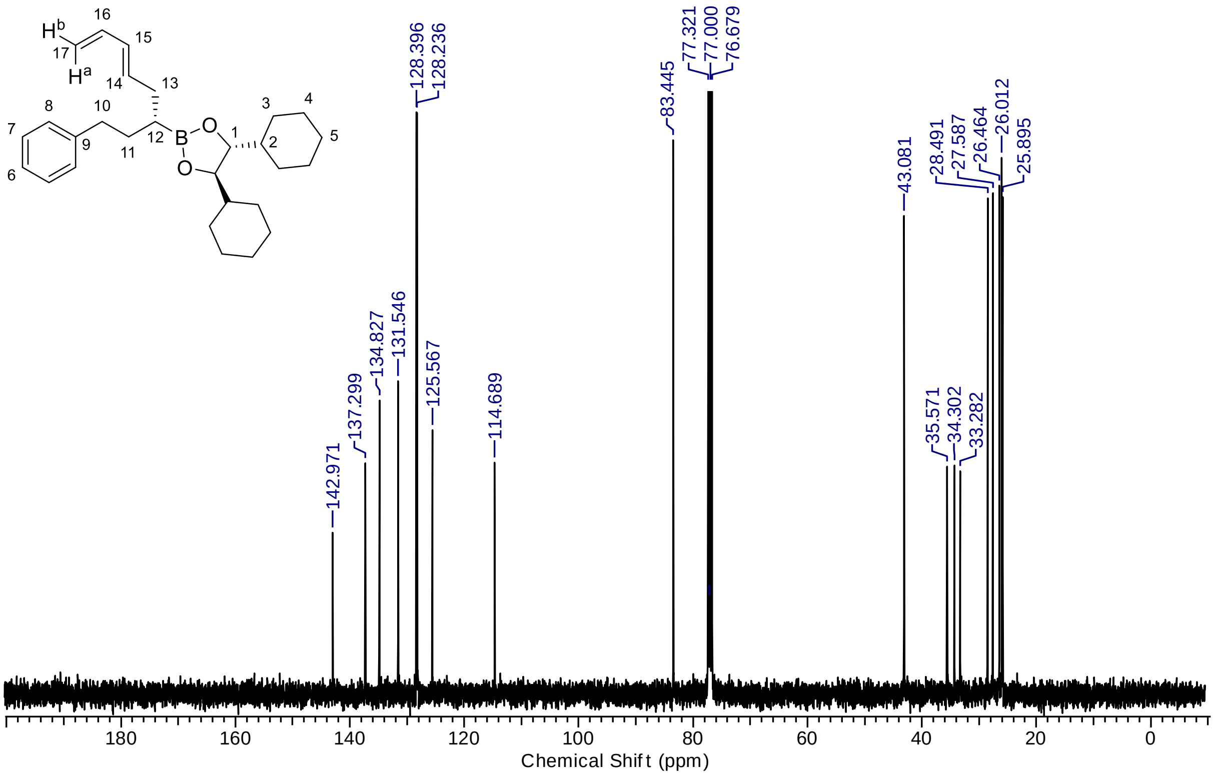

$^{1}H,^{1}H$-COSY

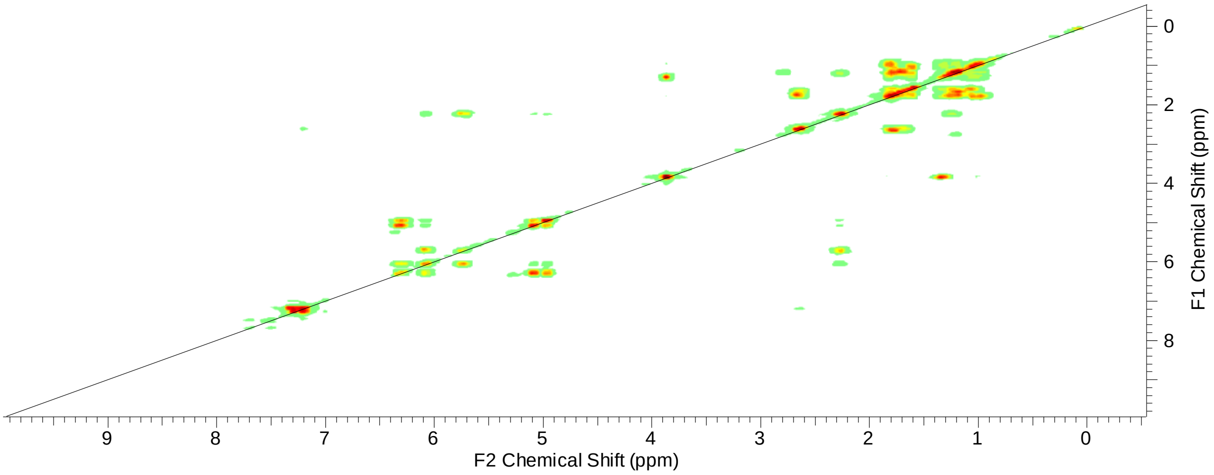

$^{1}H,^{13}C$-HSQC

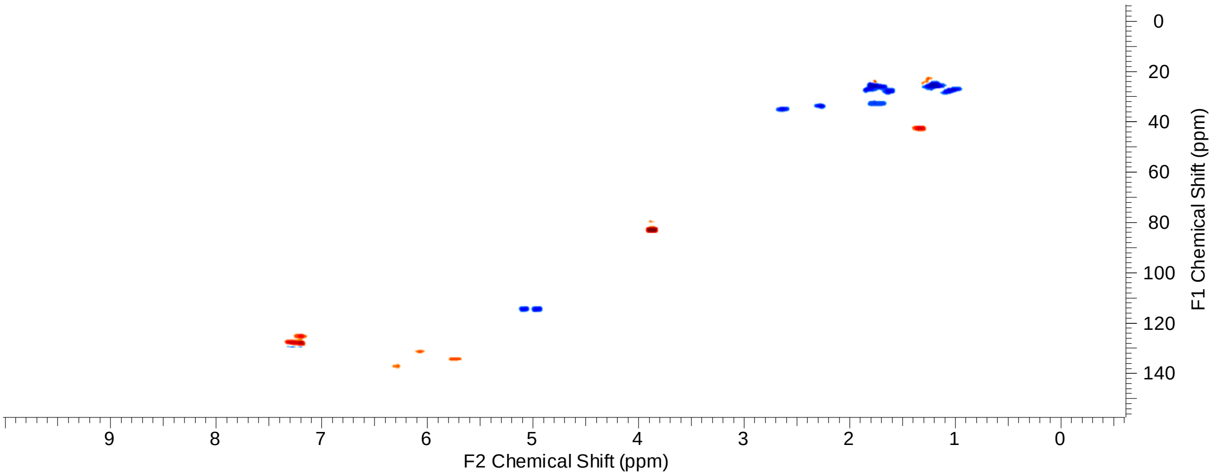

$^{1}H,^{13}C$-HMBC

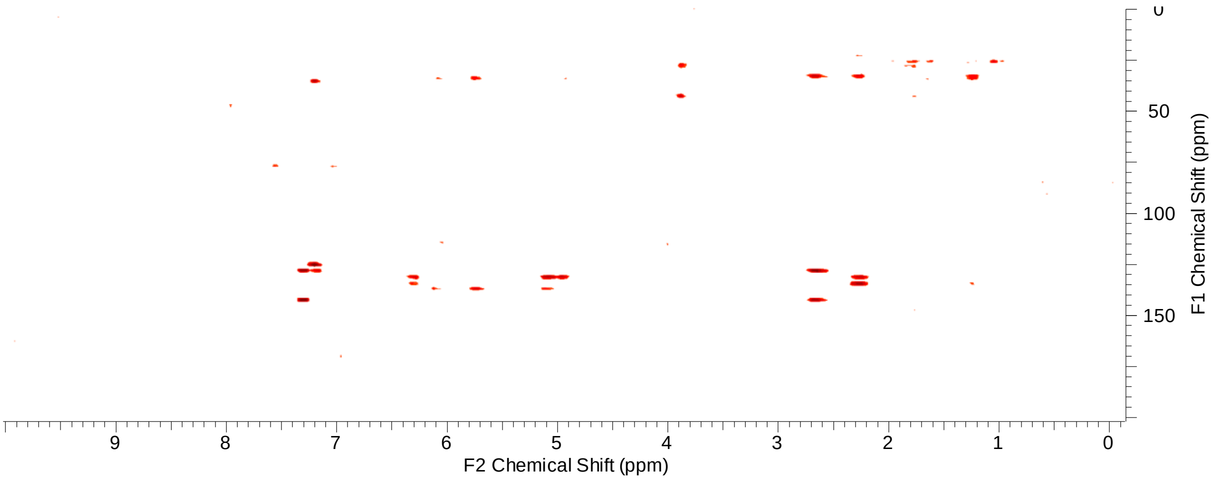

Methyl (*R*,2*E*,4*E*)-7-((4*R*,5*R*)-4,5-dicyclohexyl-1,3,2-dioxaborolan-2-yl)-9-phenylnona-2,4-dienoate (8a)

^{1}H-NMR (400 MHz, $CDCl_3$):

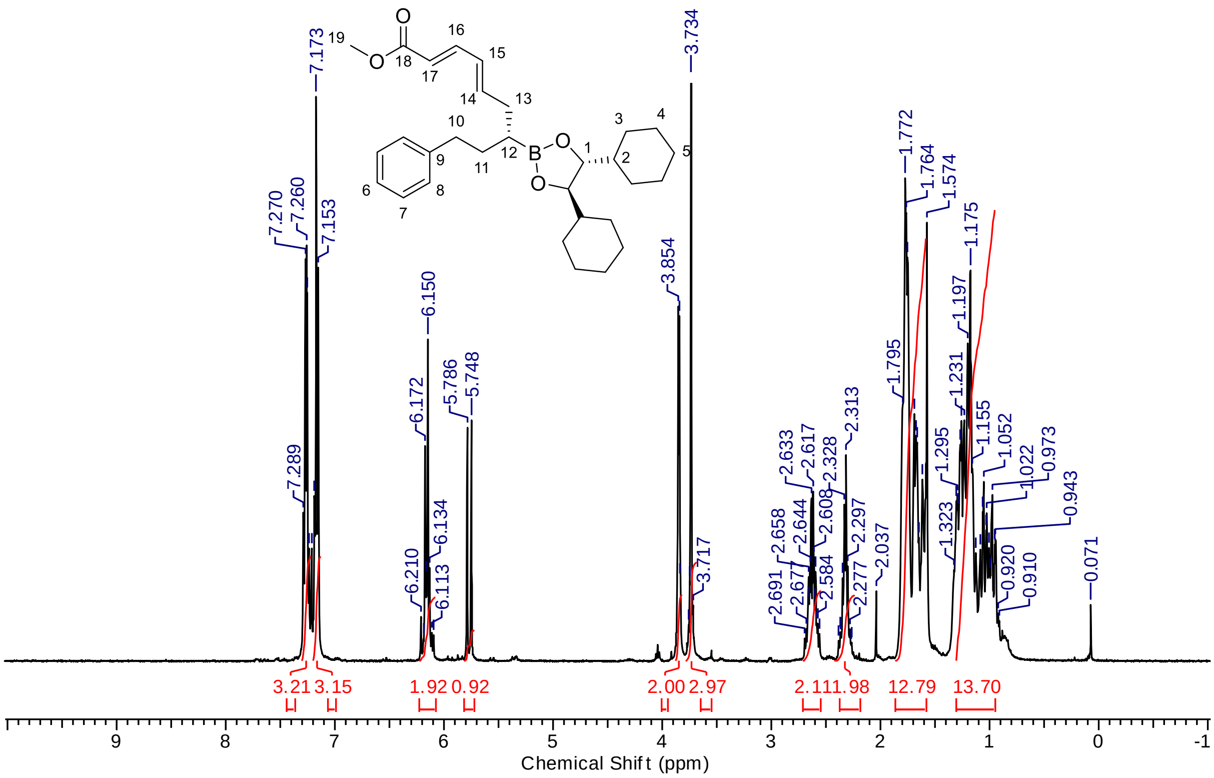

^{13}C-NMR (400 MHz, $CDCl_3$):

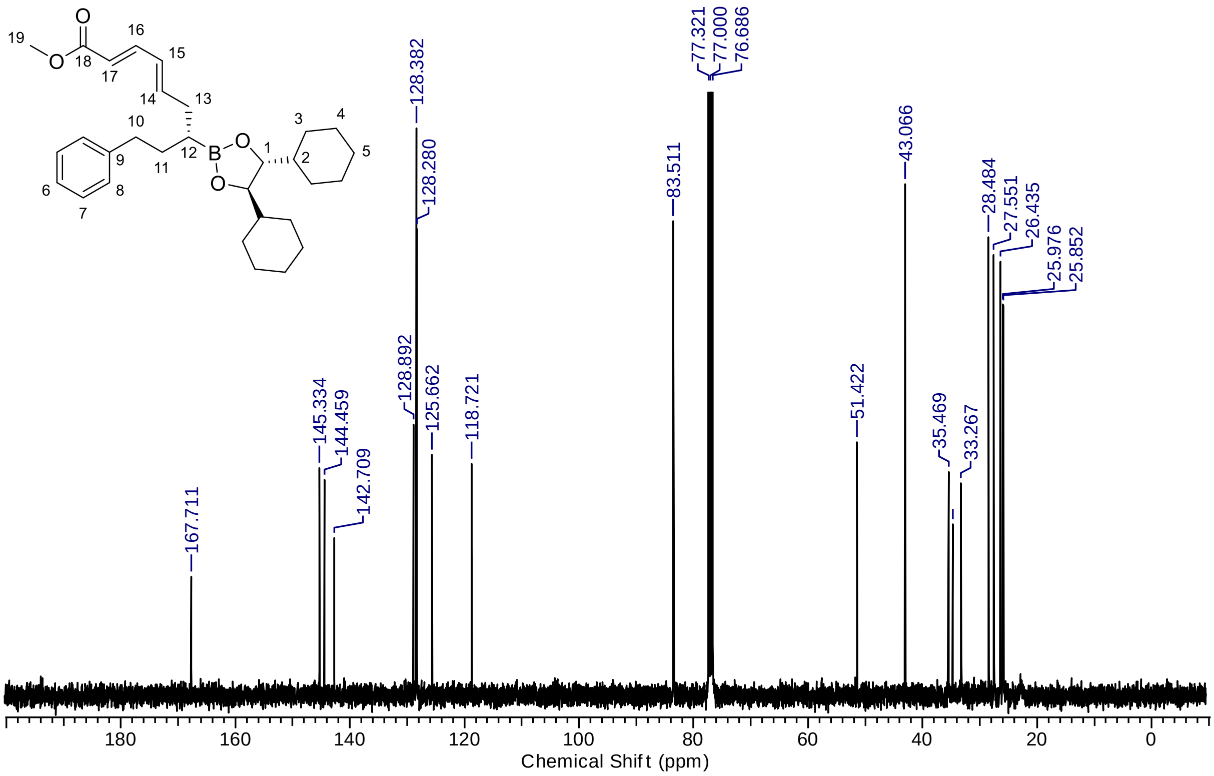

^{1}H,^{1}H-COSY

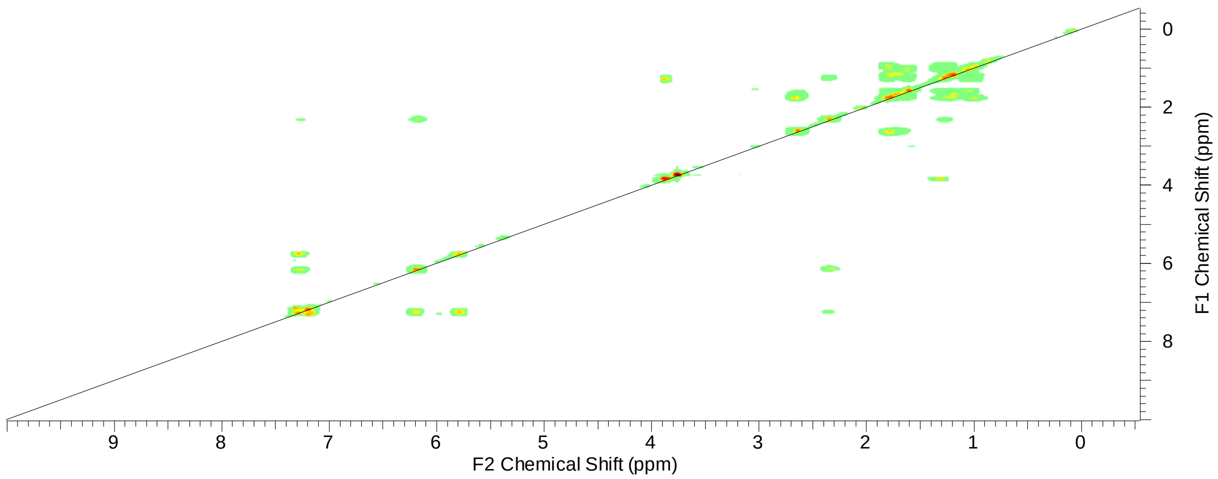

^{1}H,^{13}C-HSQC

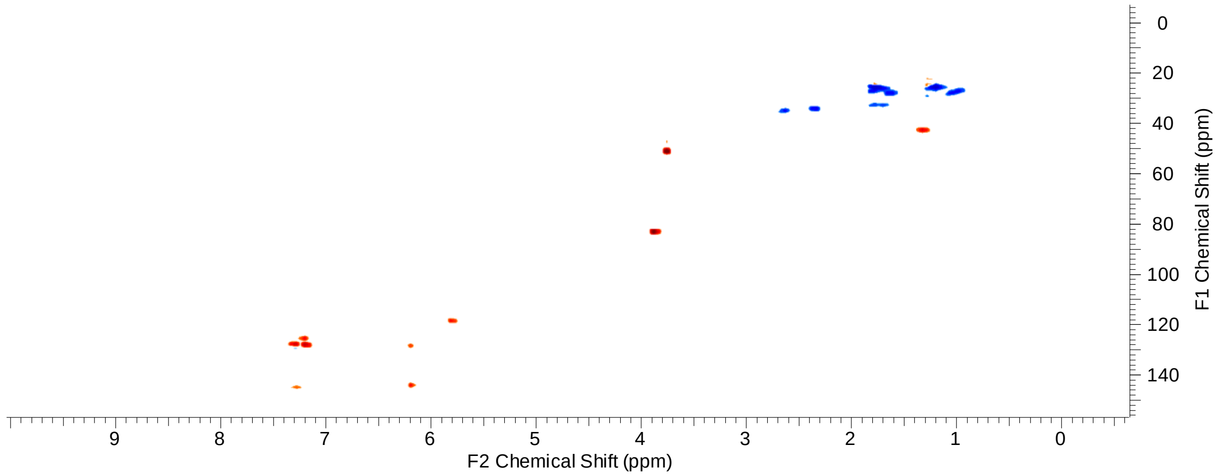

^{1}H,^{13}C-HMBC

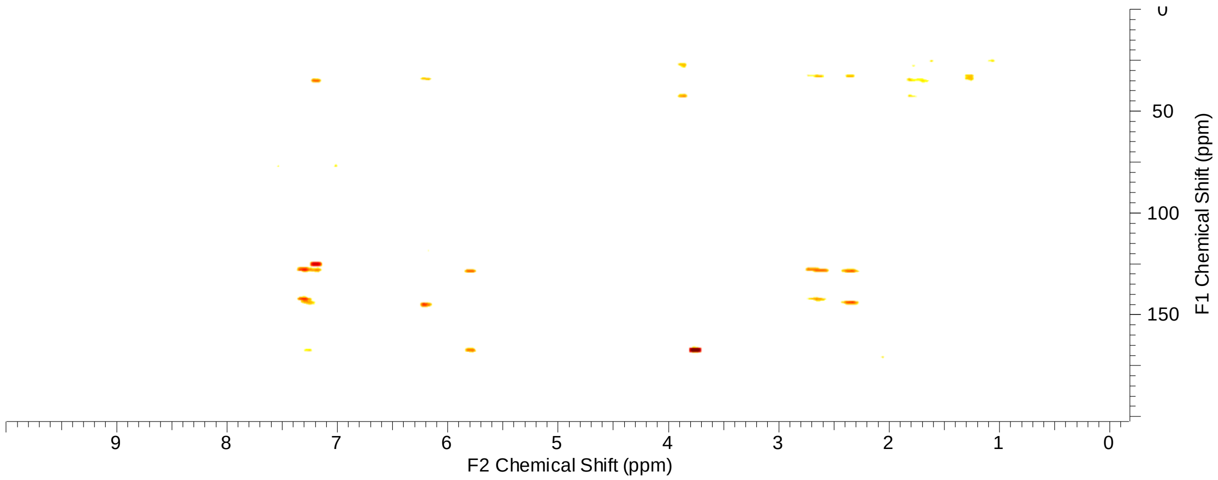

(*R*,4*E*)-7-((4*R*,5*R*)-4,5-Dicyclohexyl-1,3,2-dioxaborolan-2-yl)-9-phenylnona-2,4-dienenitrile (8b)

^{1}H-NMR (400 MHz, $CDCl_3$):

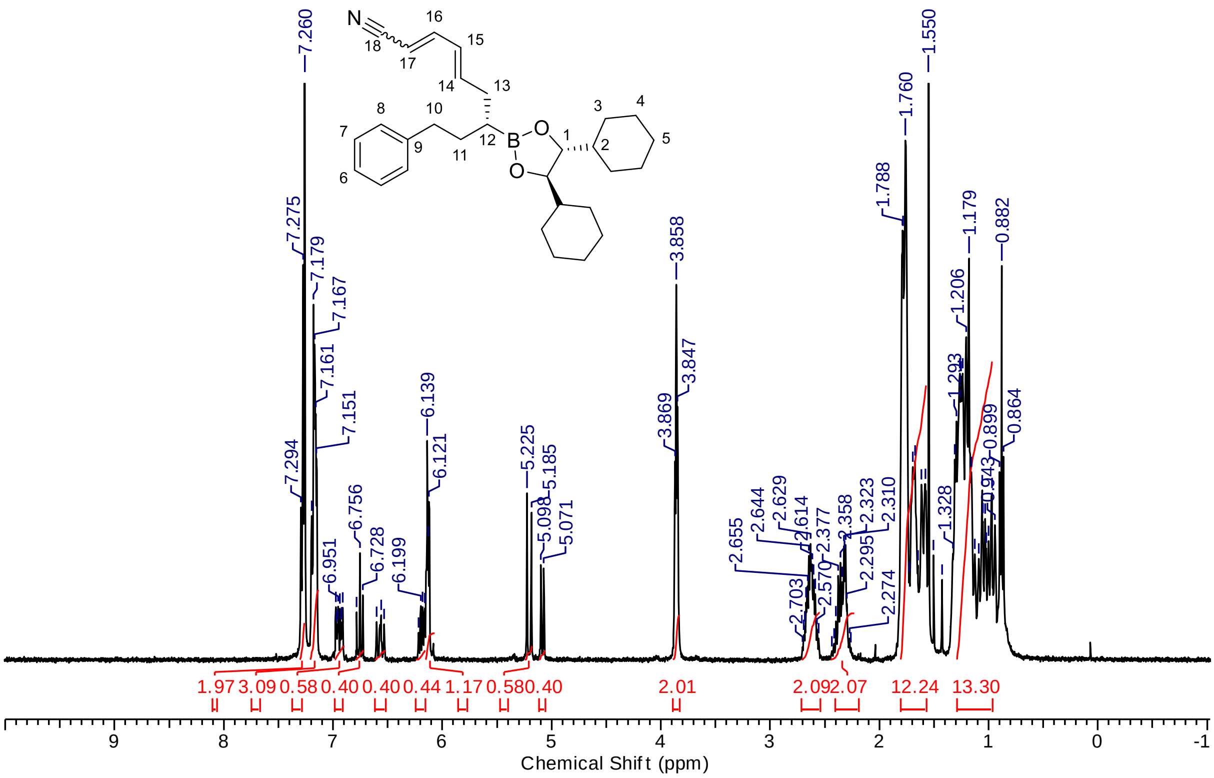

^{13}C-NMR (100 MHz, $CDCl_3$):

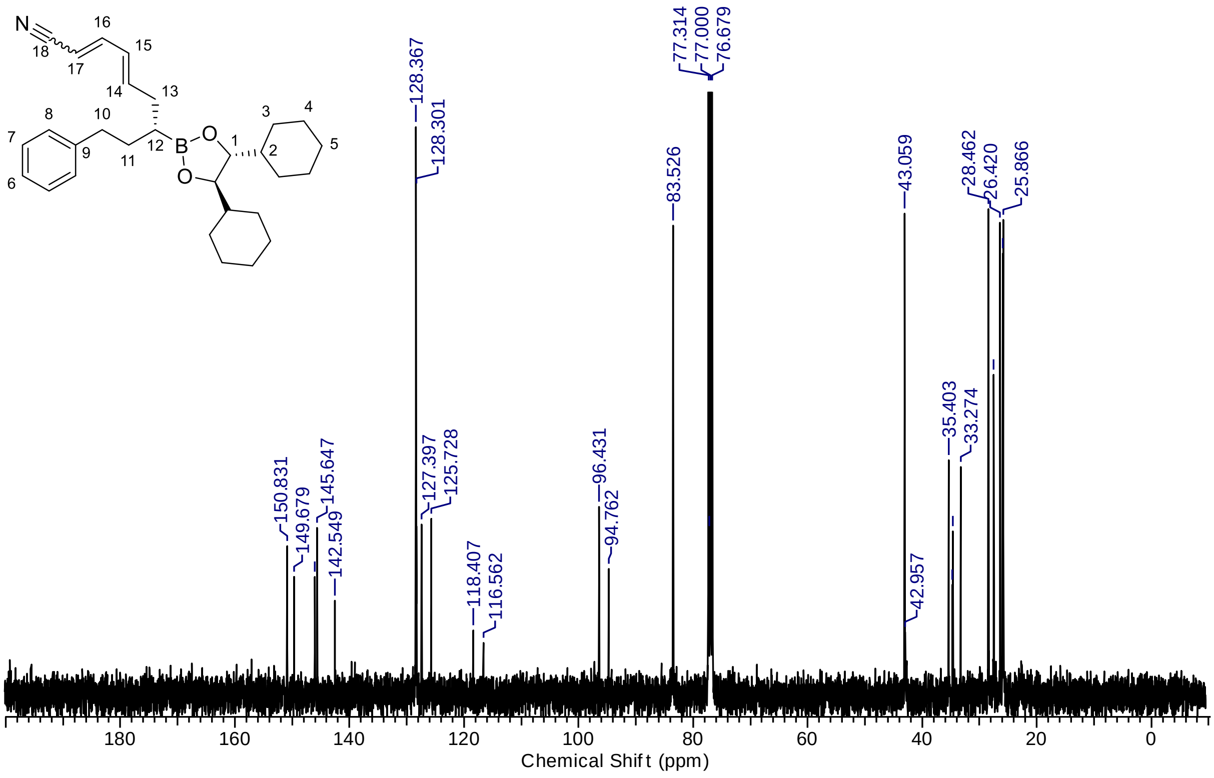

$^{1}H,^{1}H$-COSY

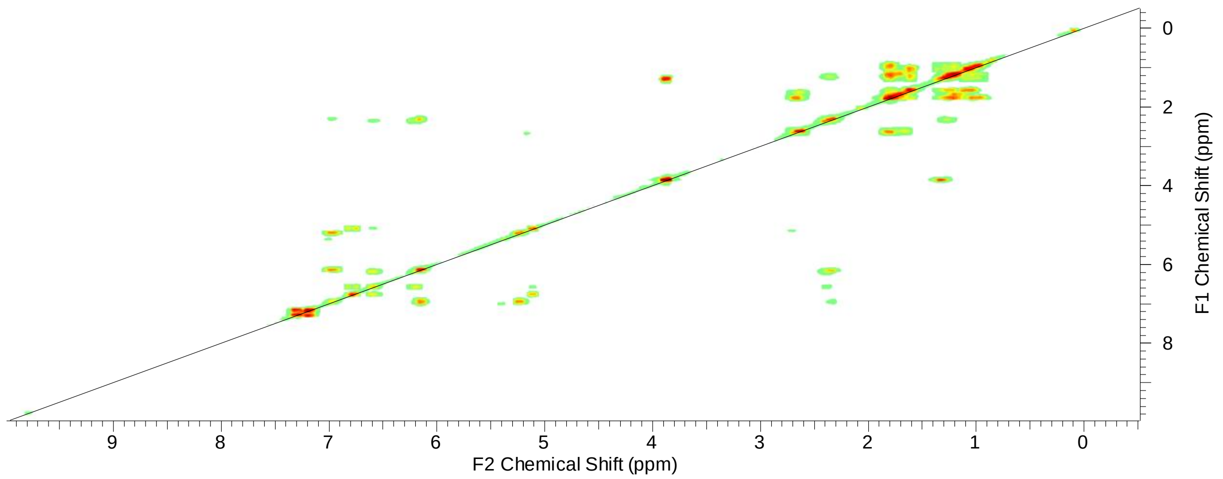

$^{1}H,^{13}C$-HSQC

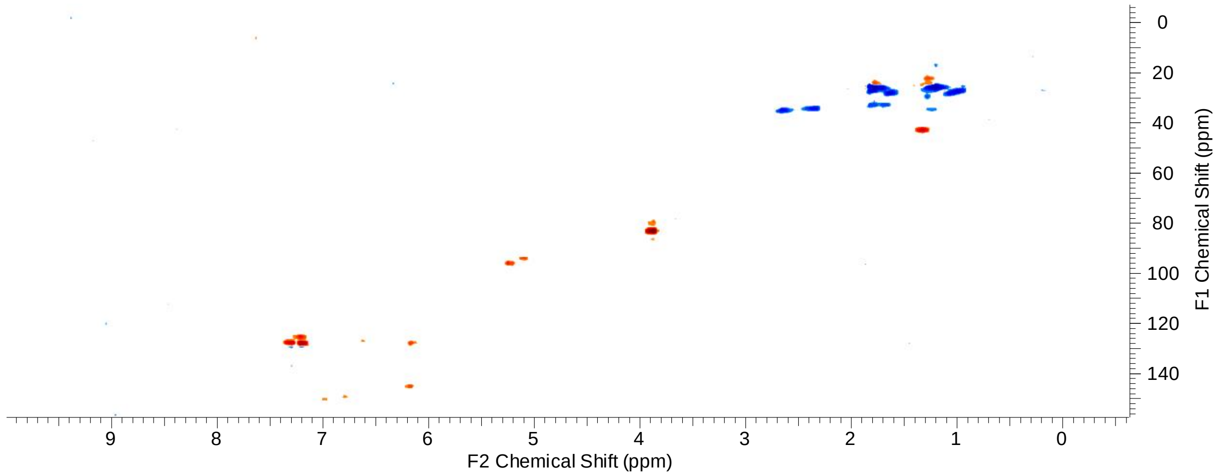

$^{1}H,^{13}C$-HMBC

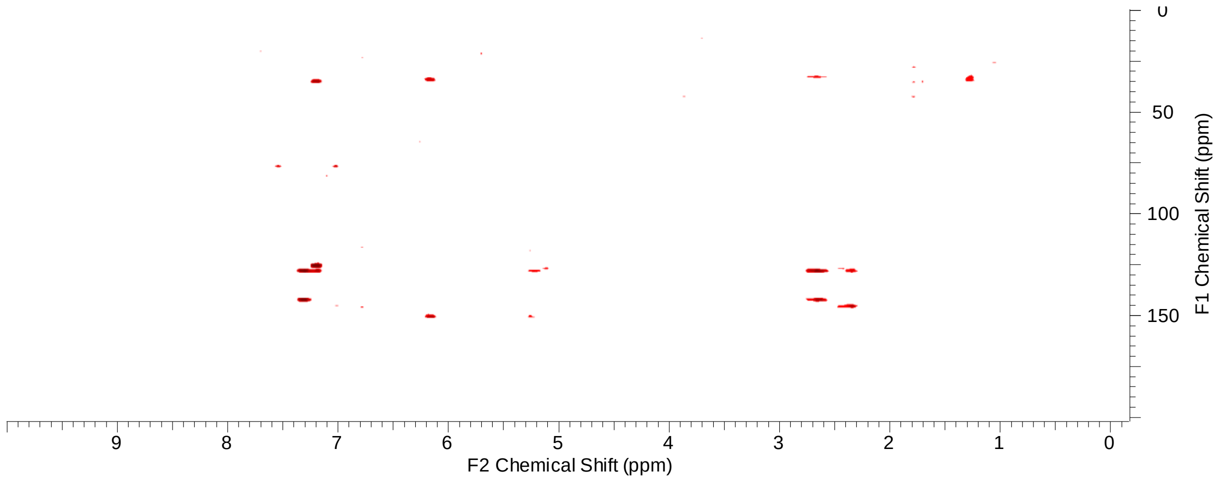

Ethyl (R,2E,4E)-7-((4R,5R)-4,5-dicyclohexyl-1,3,2-dioxaborolan-2-yl)-3-methyl-9-phenyl-nona-2,4-dienoate (8c)

^{1}H-NMR (400 MHz, $CDCl_3$):

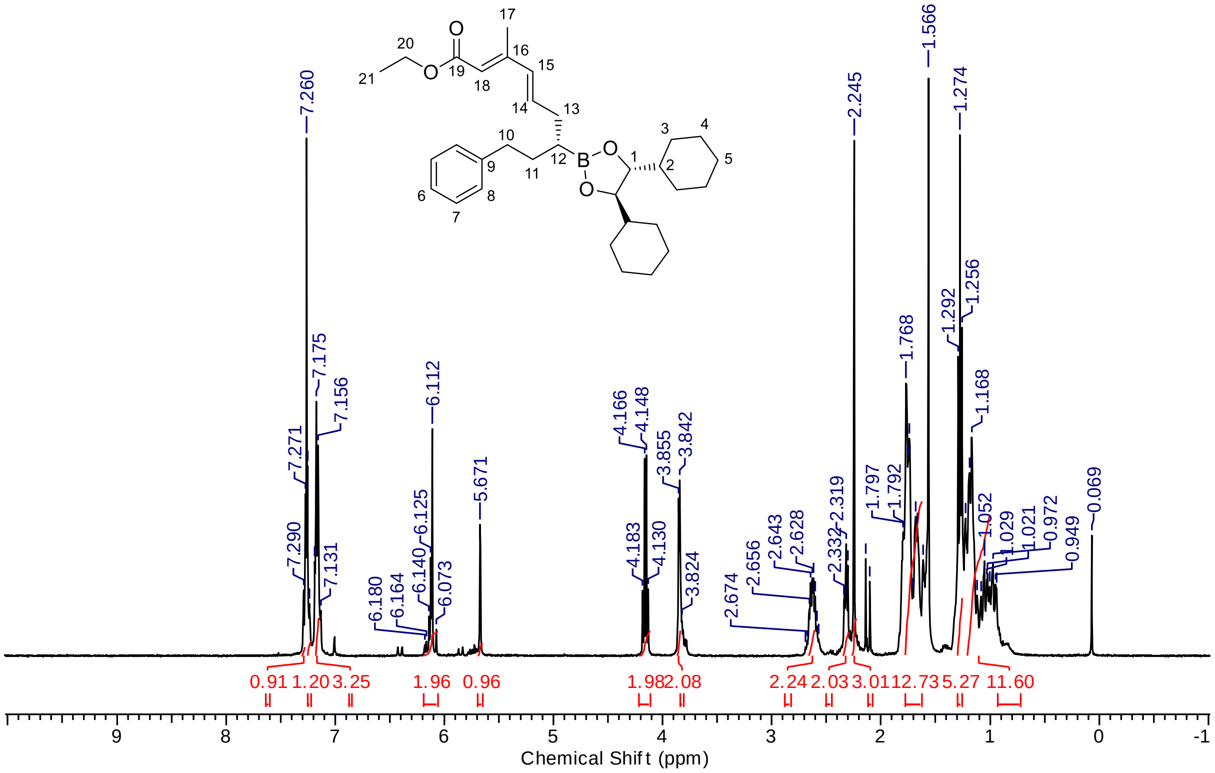

^{13}C-NMR (100 MHz, $CDCl_3$):

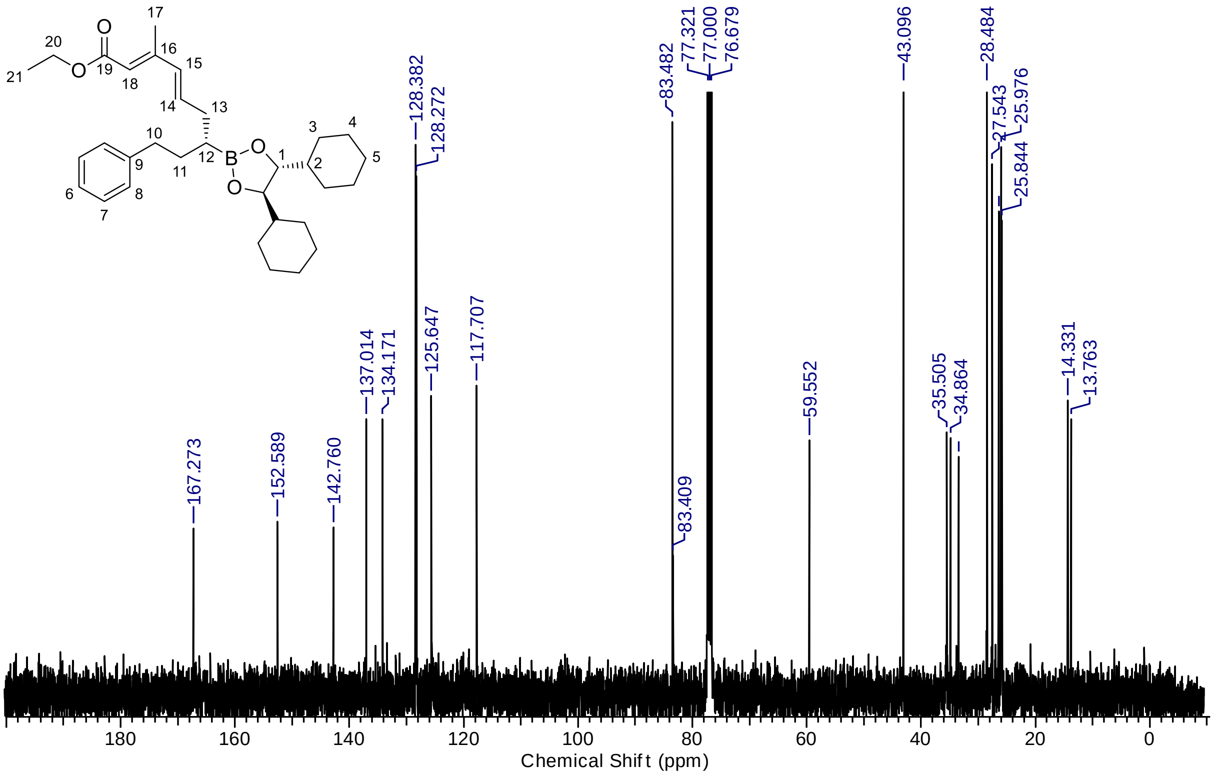

1D-NOESY (500 MHz, $CDCl_3$):

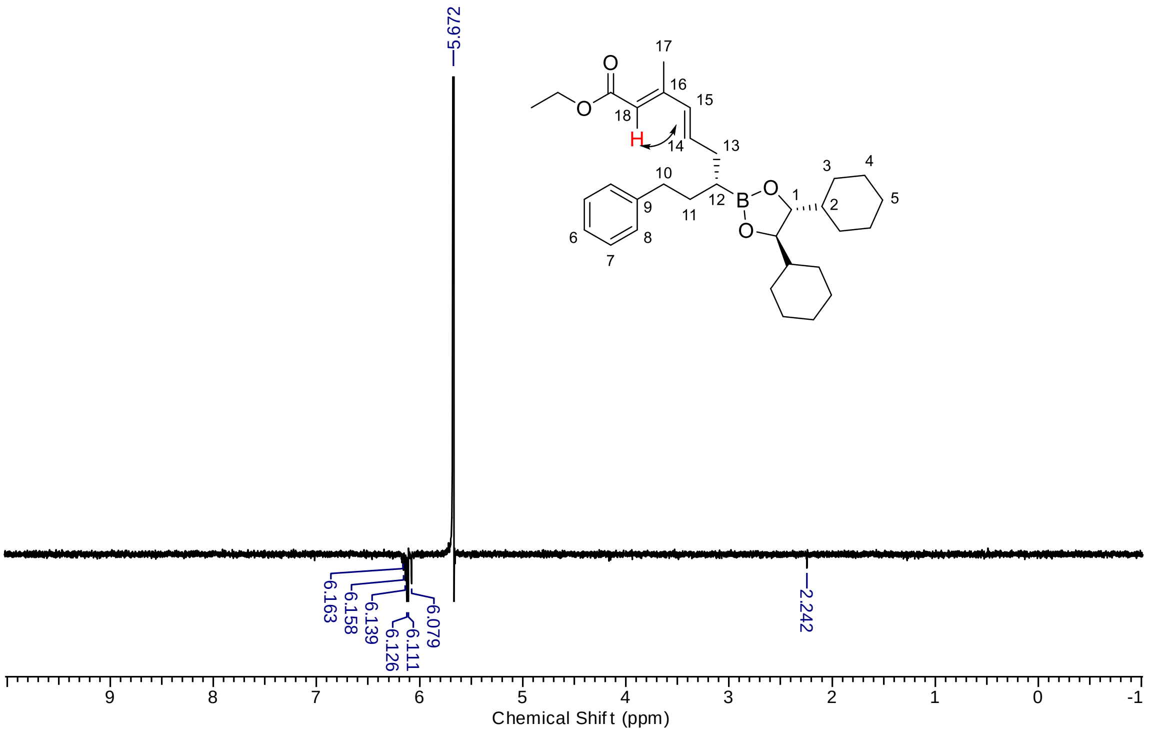

(6.16–6.08 (14-H, 15-H), 2.24 (17-H))

^{1}H,^{1}H-COSY

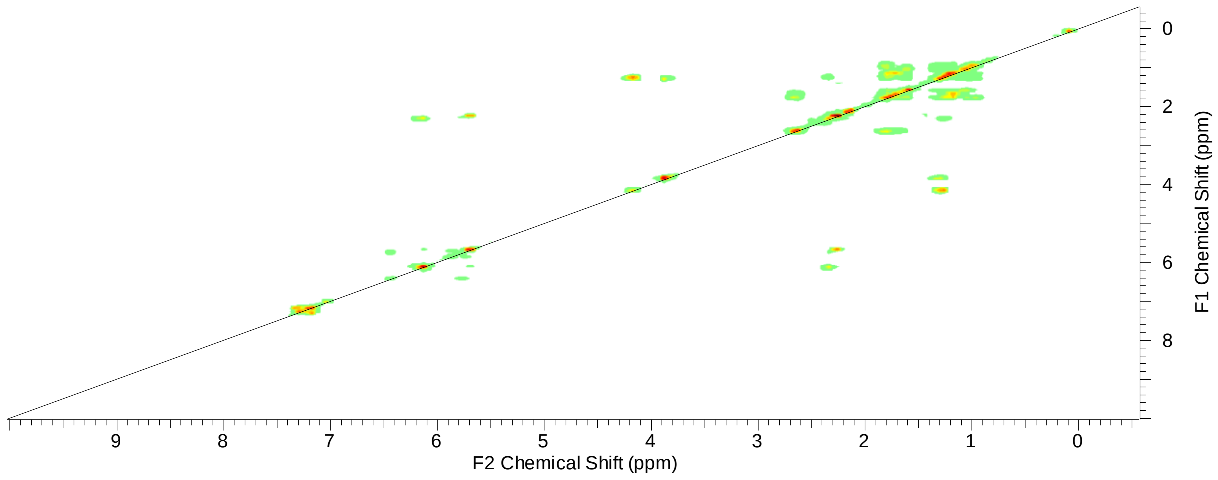

$^{1}H,^{13}C$-HSQC

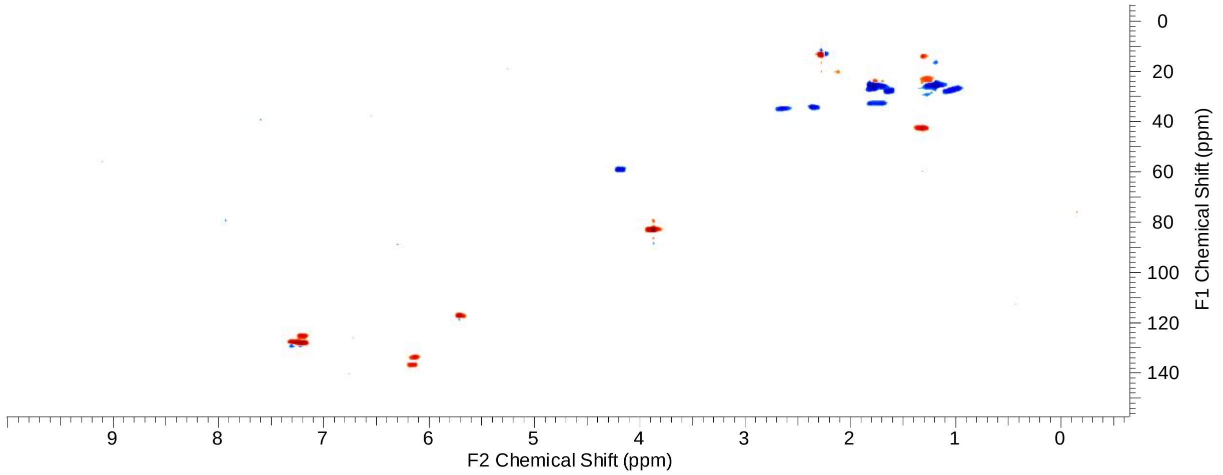

$^{1}H,^{13}C$-HMBC

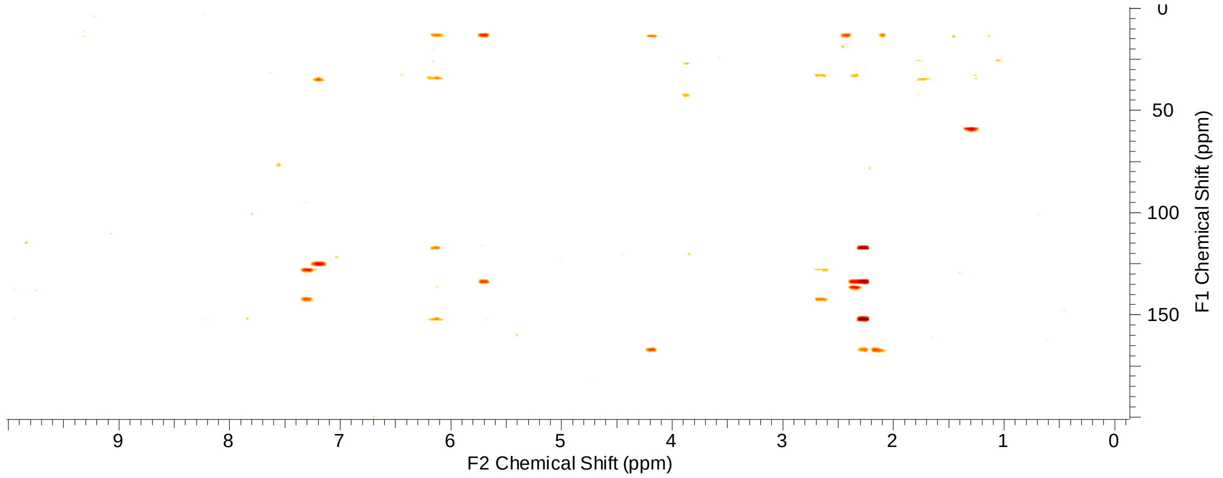

Ethyl (*R*,2*Z*,4*E*)-7-((4*R*,5*R*)-4,5-dicyclohexyl-1,3,2-dioxaborolan-2-yl)-3-methyl-9-phenylnona-2,4-dienoate (8d)

^{1}H-NMR (500 MHz, $CDCl_3$):

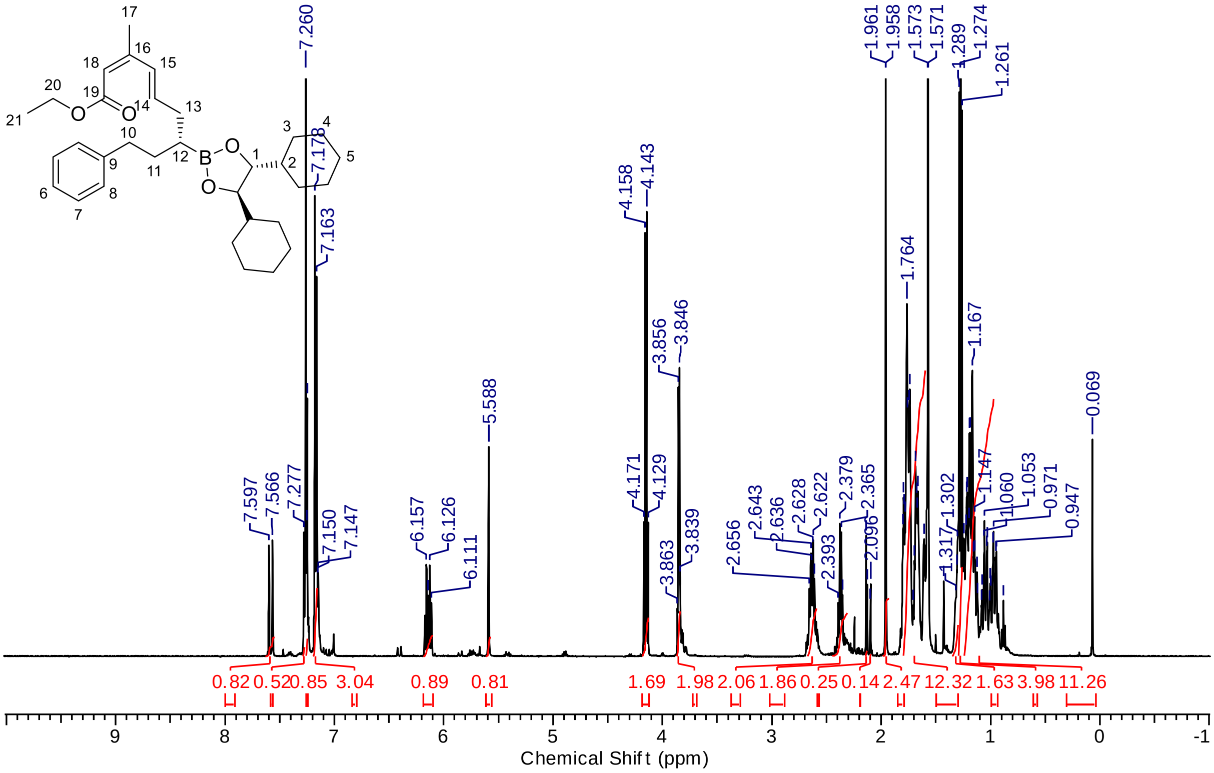

^{13}C-NMR (125 MHz, $CDCl_3$):

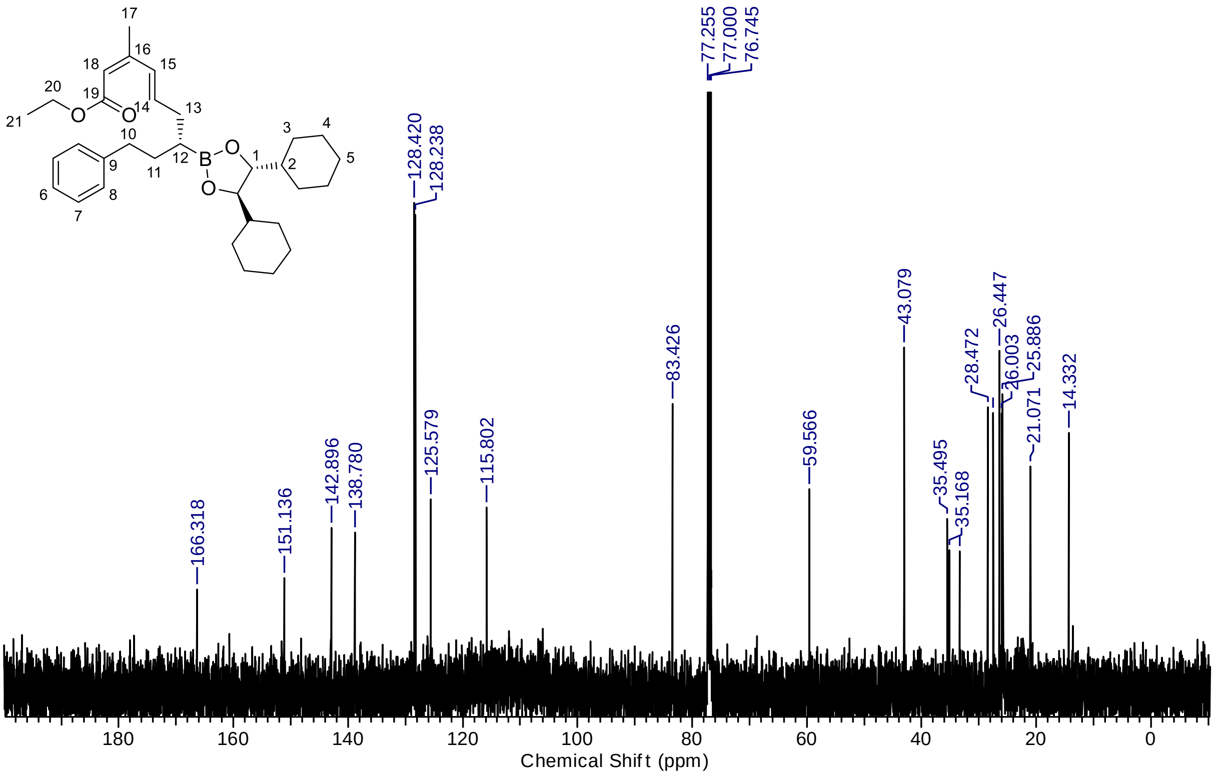

1D-NOESY (500 MHz, $CDCl_3$):

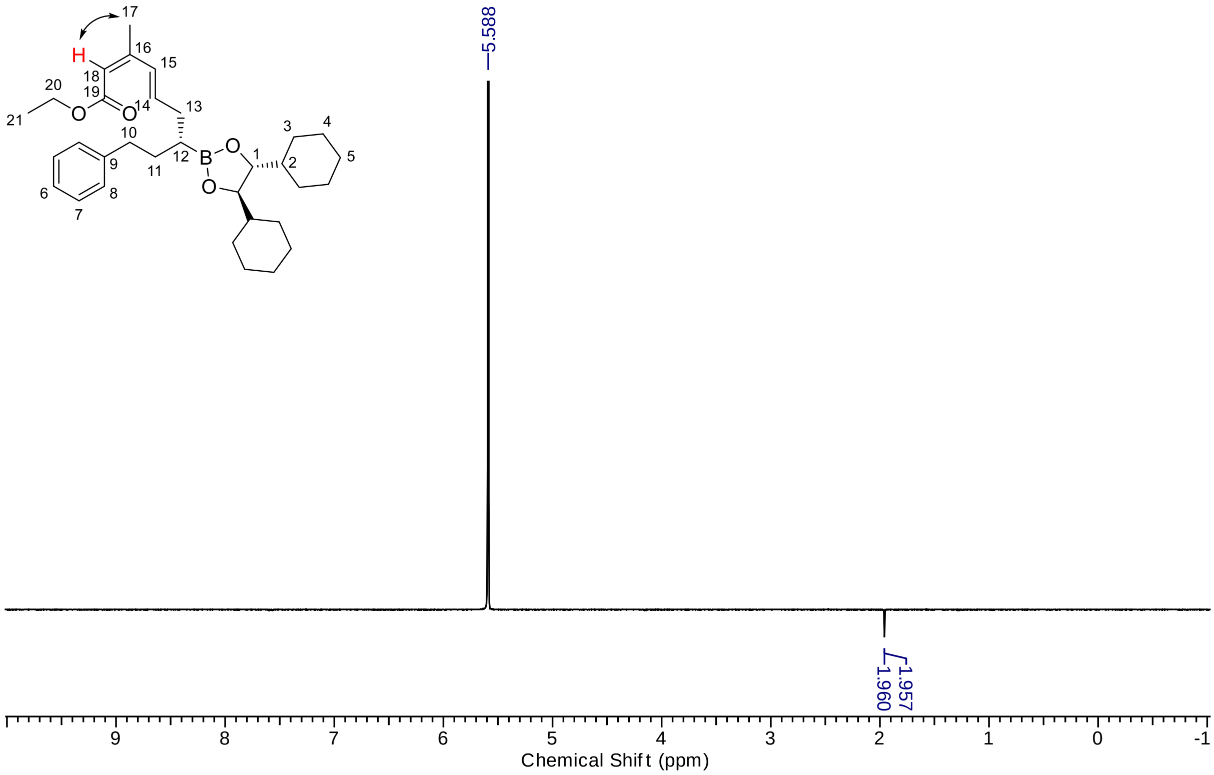

(1.96 (17-H))

^{1}H,^{1}H-COSY

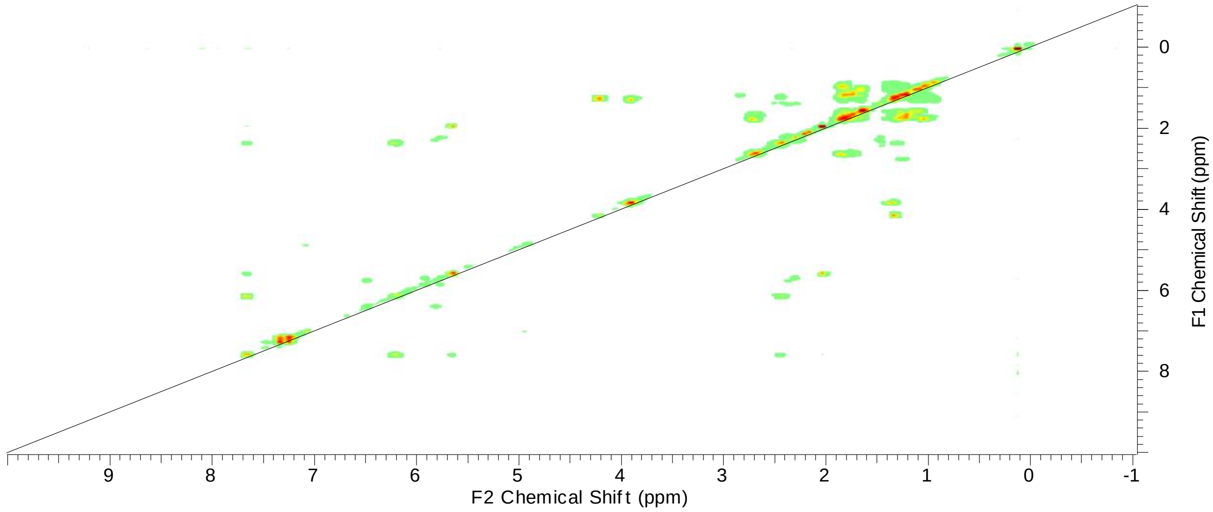

$^{1}H,^{13}C$-HSQC

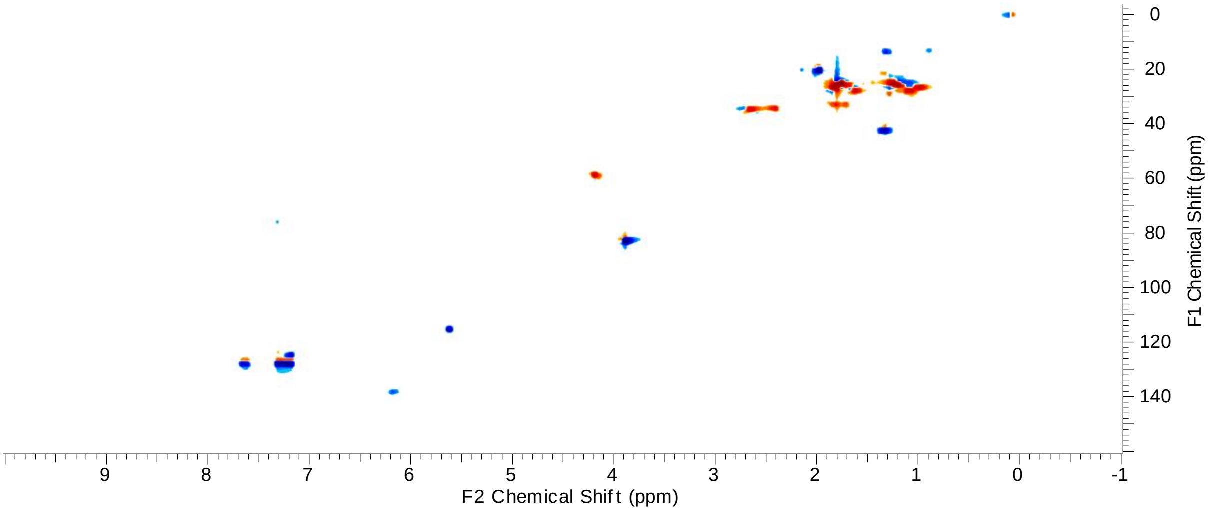

$^{1}H,^{13}C$-HMBC

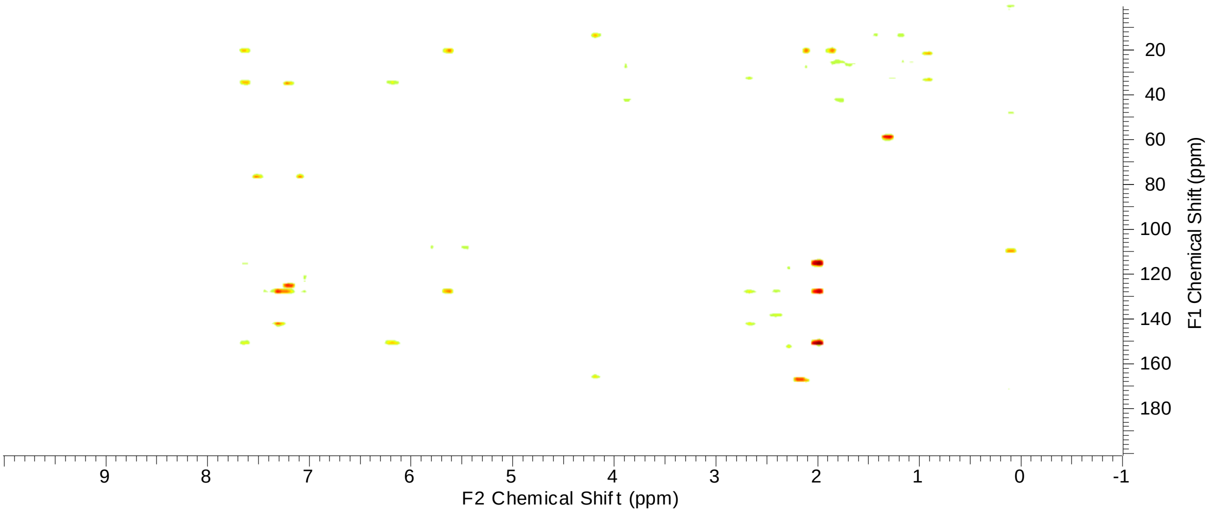

((*R*,*Z*)-5-((4*R*,5*R*)-4,5-Dicyclohexyl-1,3,2-dioxaborolan-2-yl)-7-phenylhept-2-en-2-yl)-trimethylsilane (9)

^{1}H-NMR (400 MHz, $CDCl_3$):

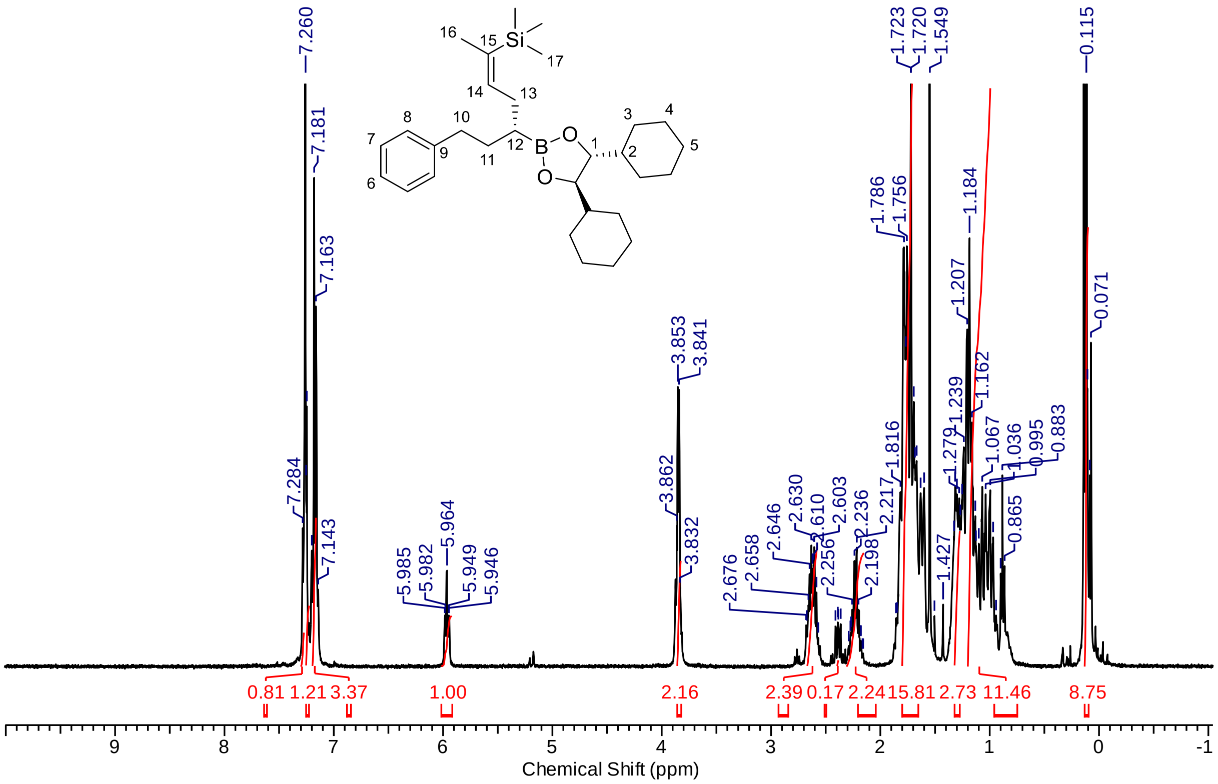

^{13}C-NMR (100 MHz, $CDCl_3$):

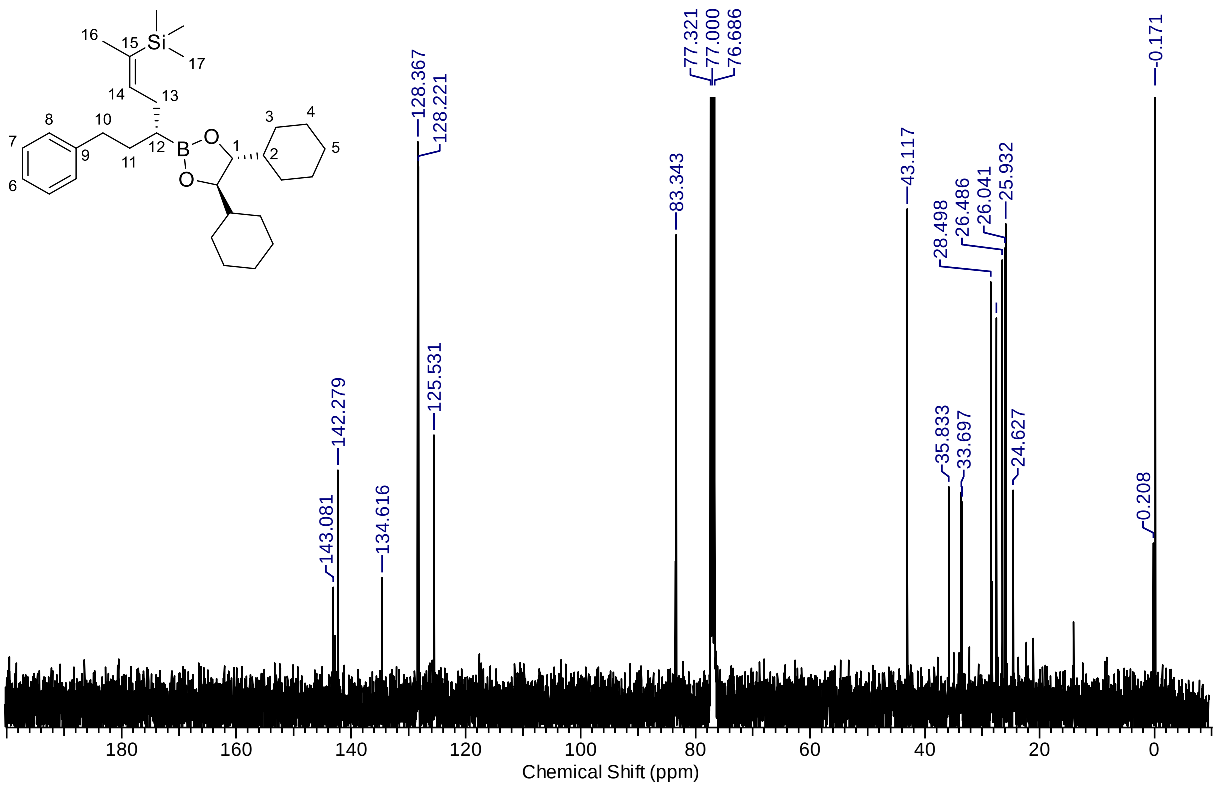

$^{1}H,^{1}H$-COSY

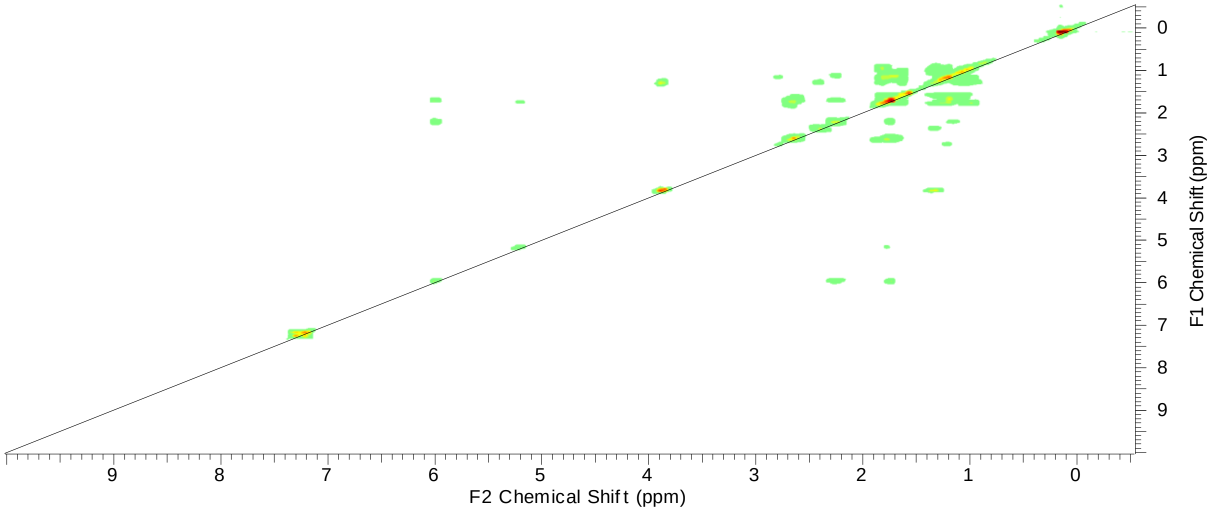

$^{1}H,^{13}C$-HSQC

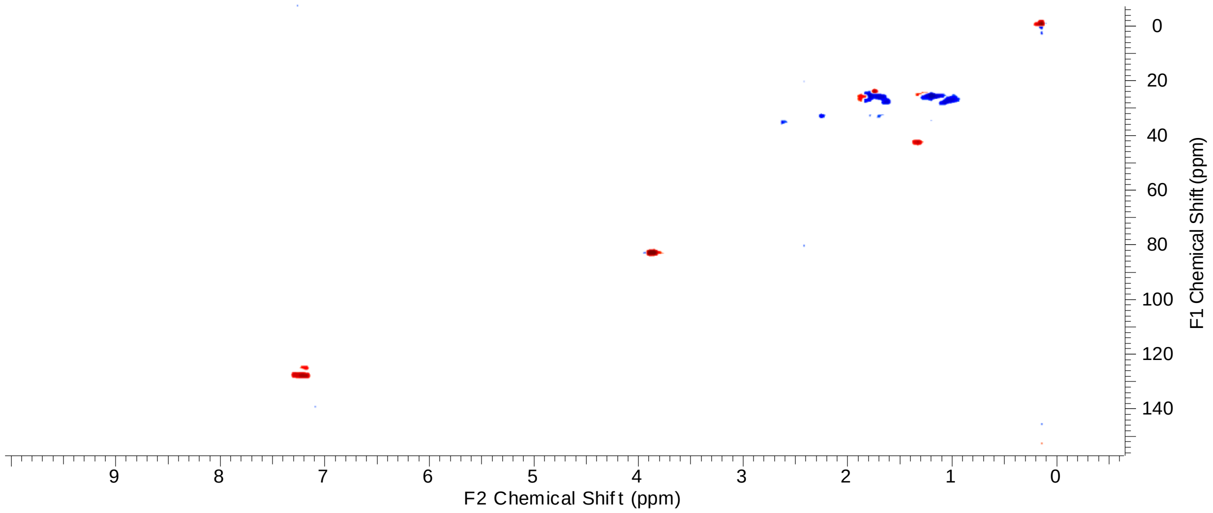

$^{1}H,^{13}C$-HMBC

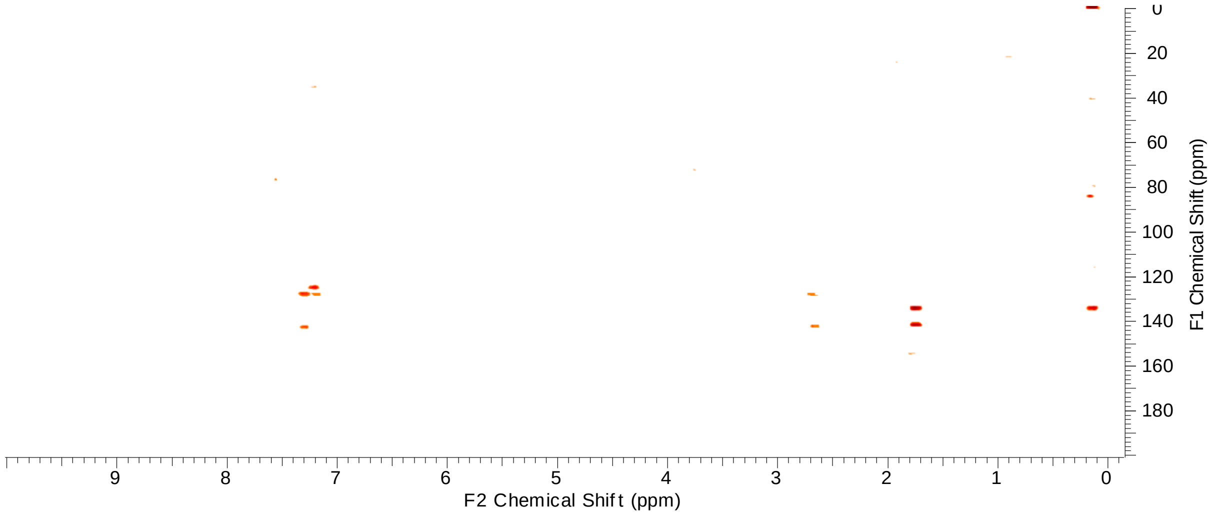

(*R*,*Z*)-6-methyl-1-phenyloct-5-en-3-ol (12)

^{1}H-NMR (400 MHz, $CDCl_3$):

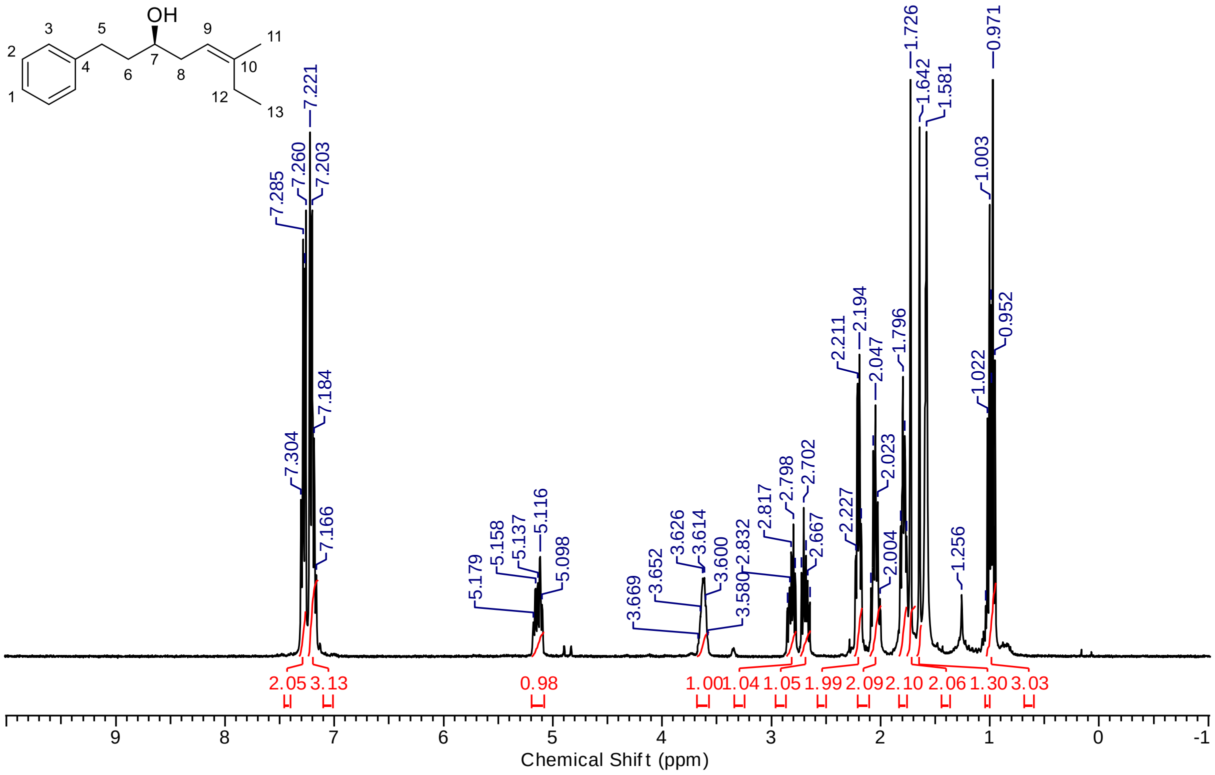

^{13}C-NMR (100 MHz, $CDCl_3$):

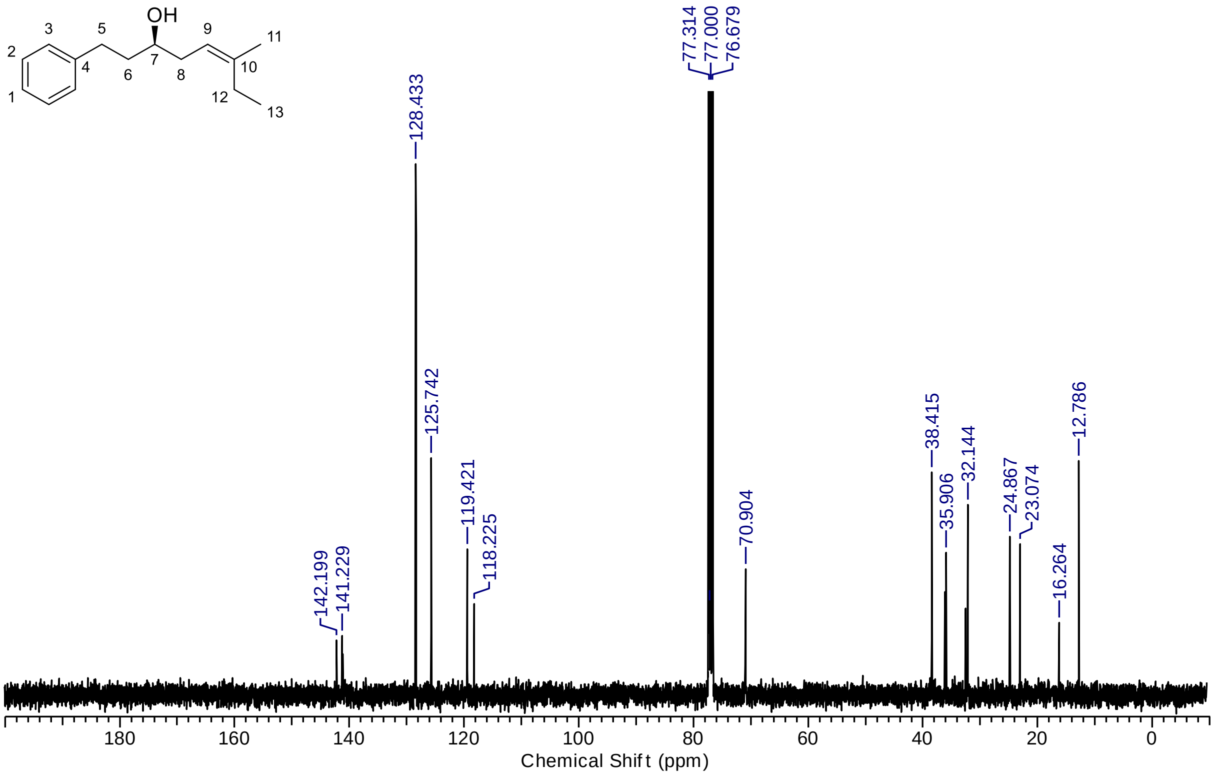

1D-NOESY (500 MHz, $CDCl_3$):

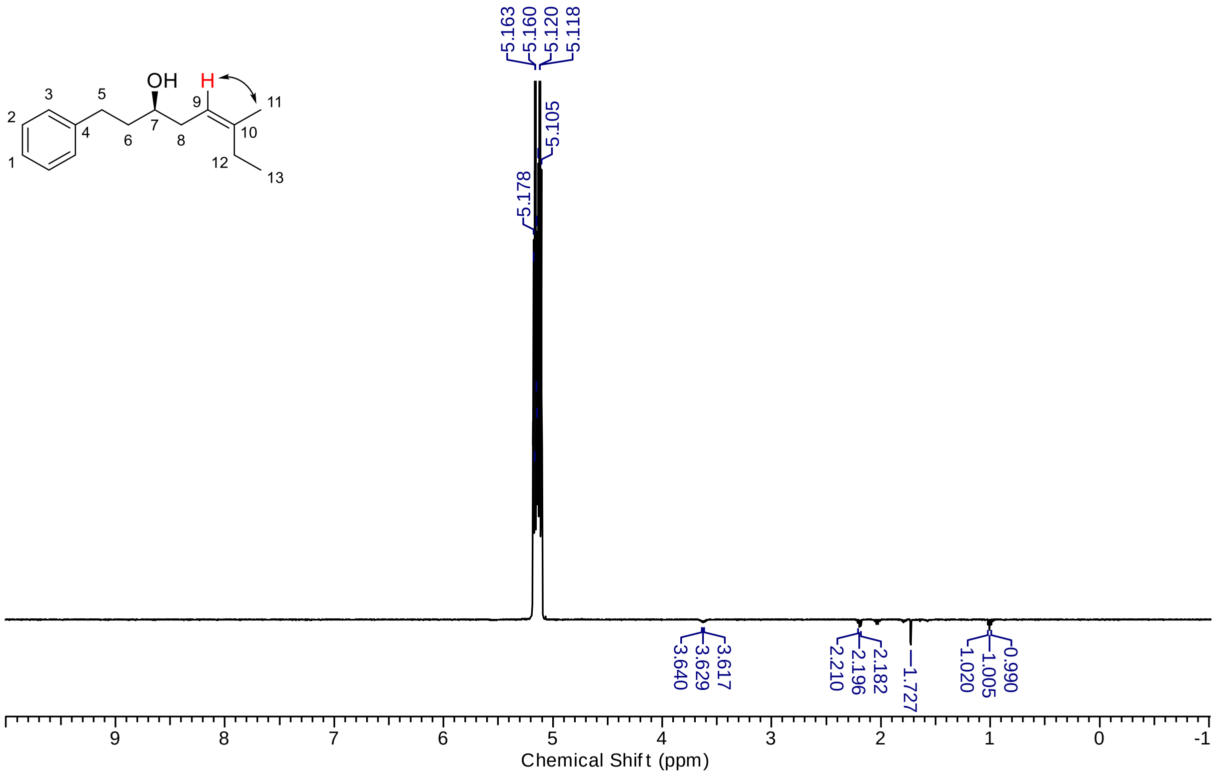

(5.18–5.11 (9-H), 3.64–3.62 (7-H), 2.21–2.18 (8-H), 1.73 (11-H), 1.02–0.99 (13-H))

^{1}H,^{1}H-COSY

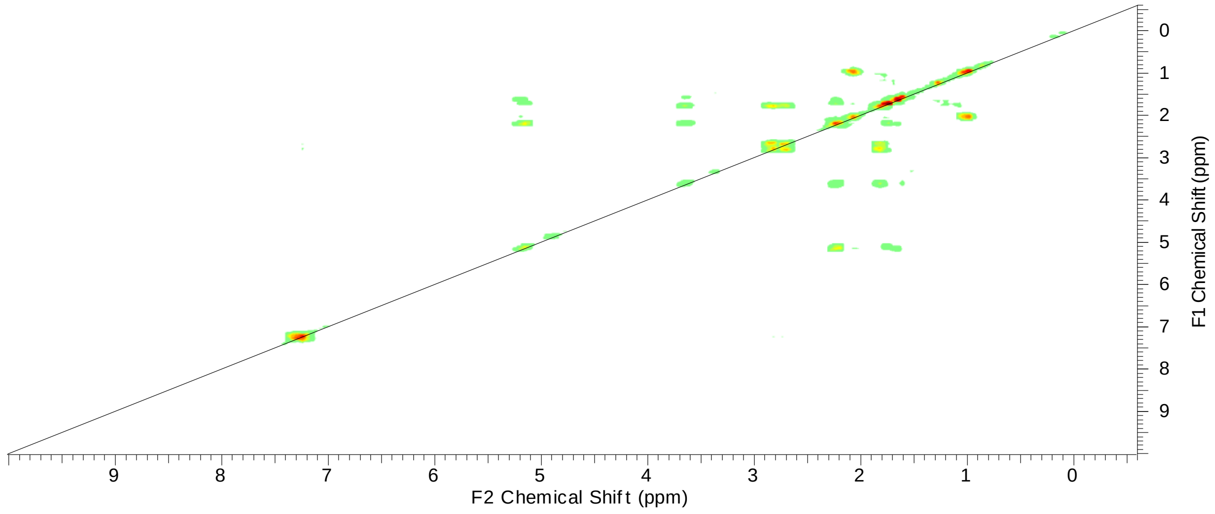

$^{1}H,^{13}C$-HSQC

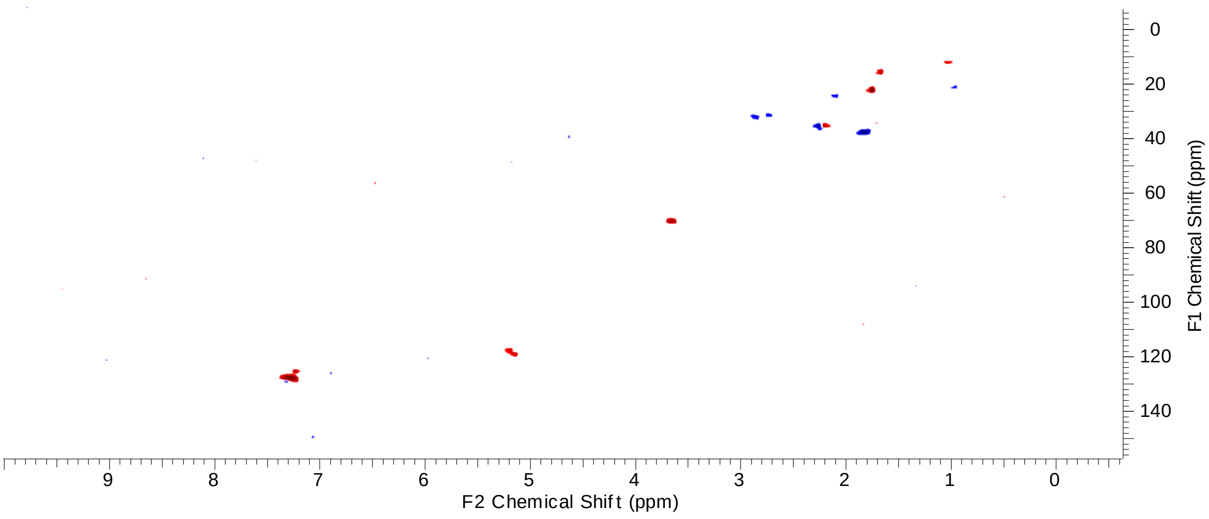

$^{1}H,^{13}C$-HMBC

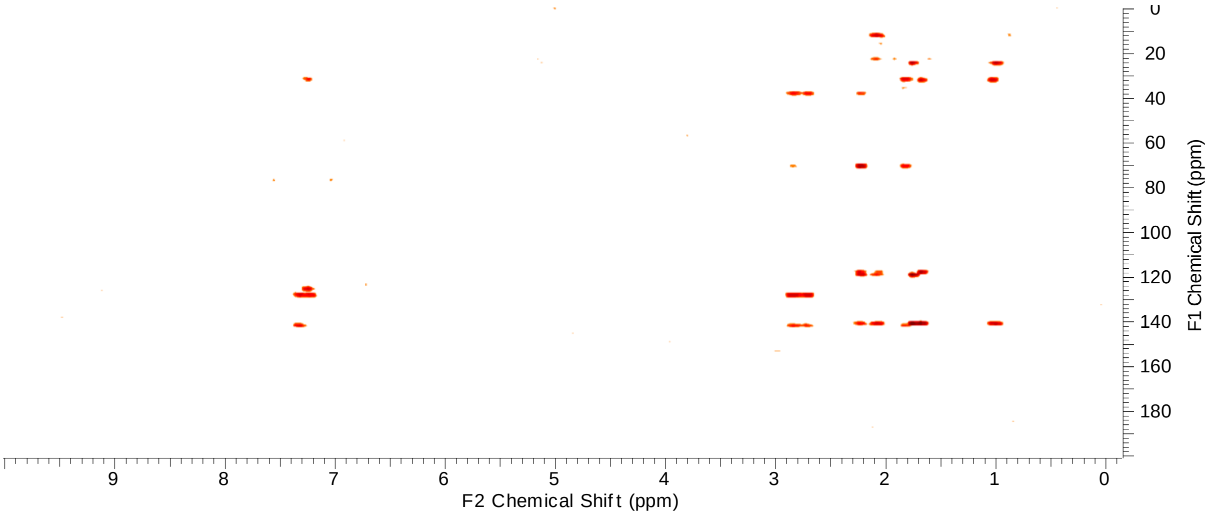

***tert*-Butyl(((2*S*,3*R*)-2-((4*R*,5*R*)-4,5-dicyclohexyl-1,3,2-dioxaborolan-2-yl)-4-methylpentan-3-yl)oxy)dimethylsilane (13)**

^{1}H-NMR (400 MHz, $CDCl_3$):

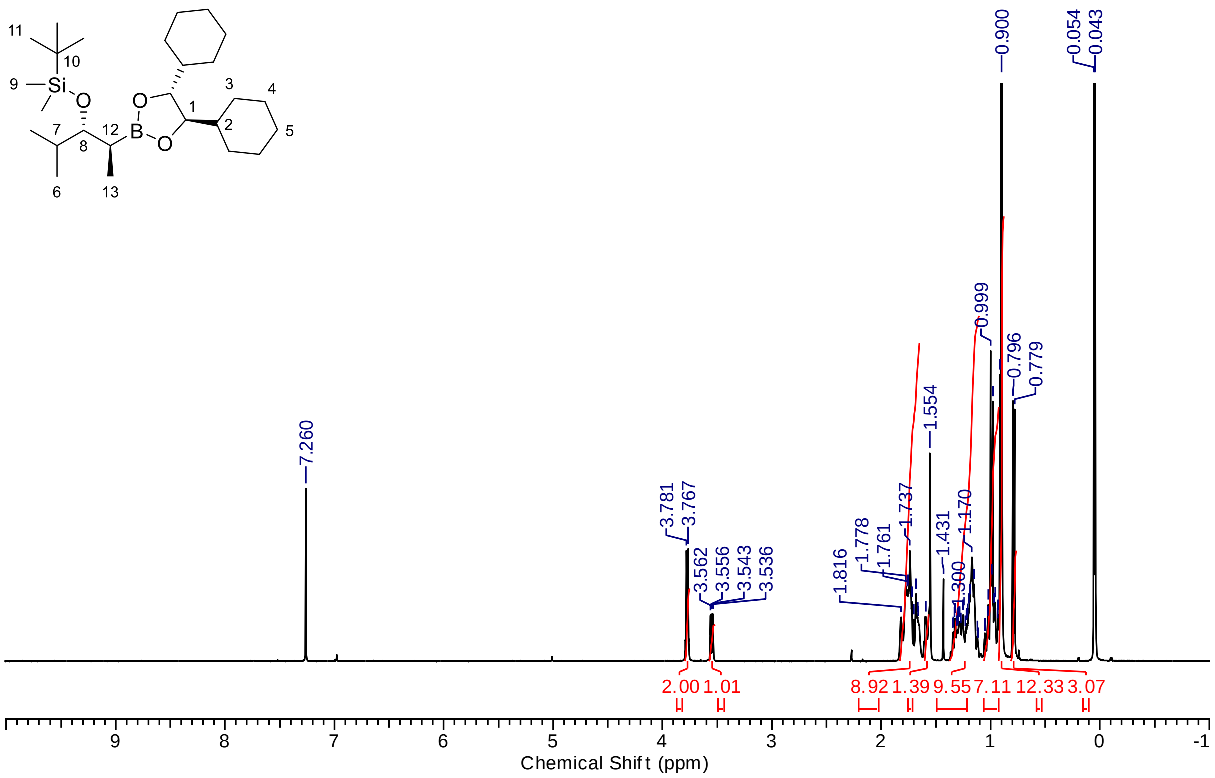

^{13}C-NMR (100 MHz, $CDCl_3$):

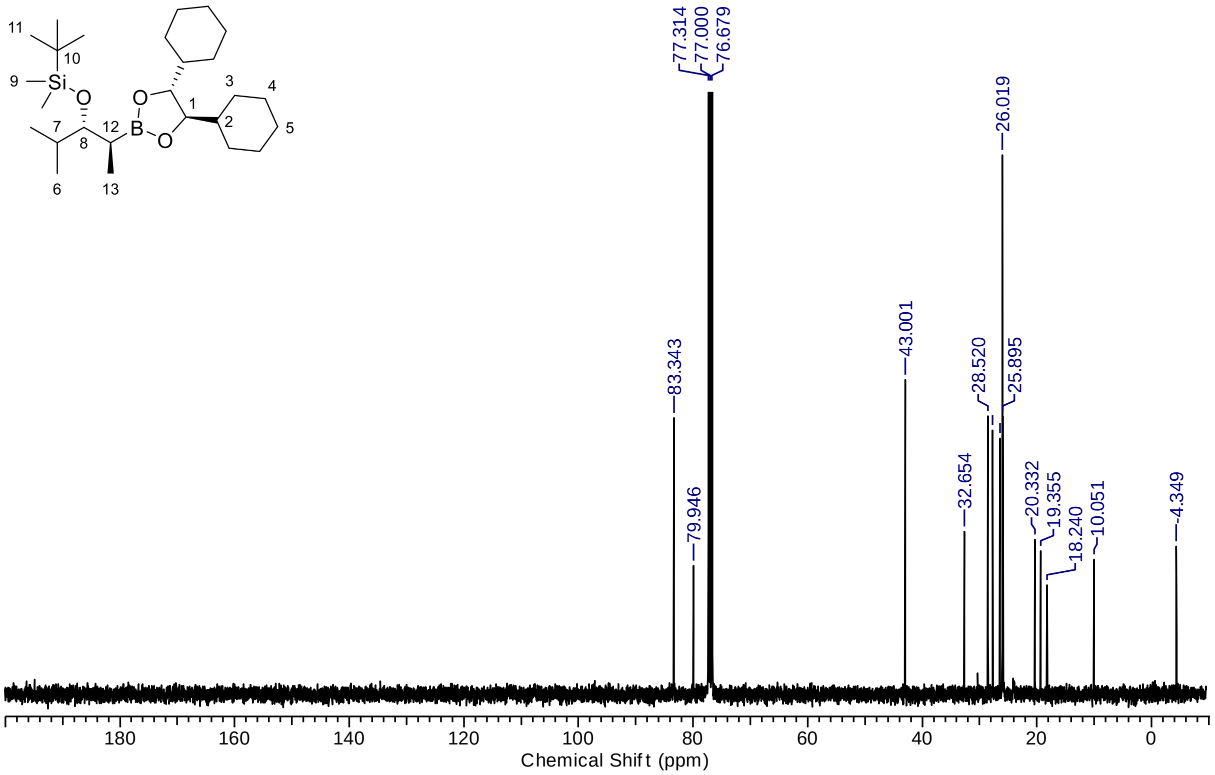

$^{1}H,^{1}H$-COSY

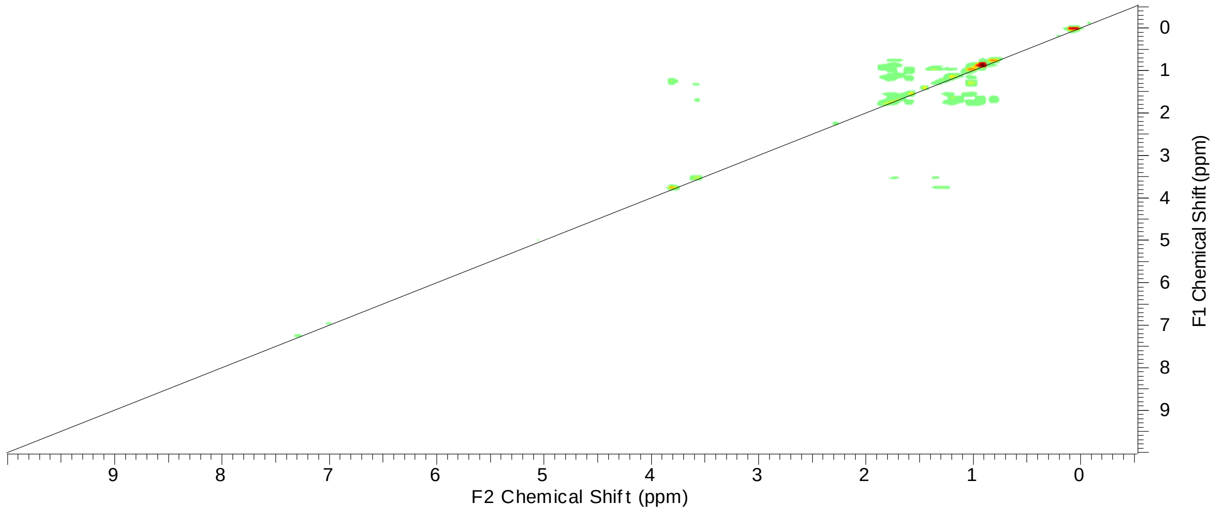

$^{1}H,^{13}C$-HSQC

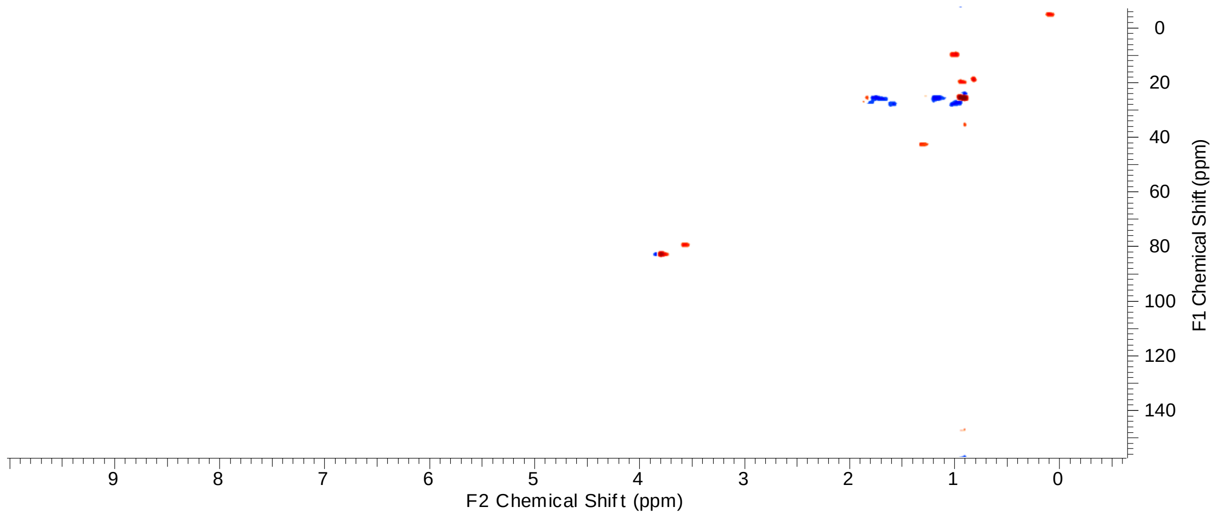

$^{1}H,^{13}C$-HMBC

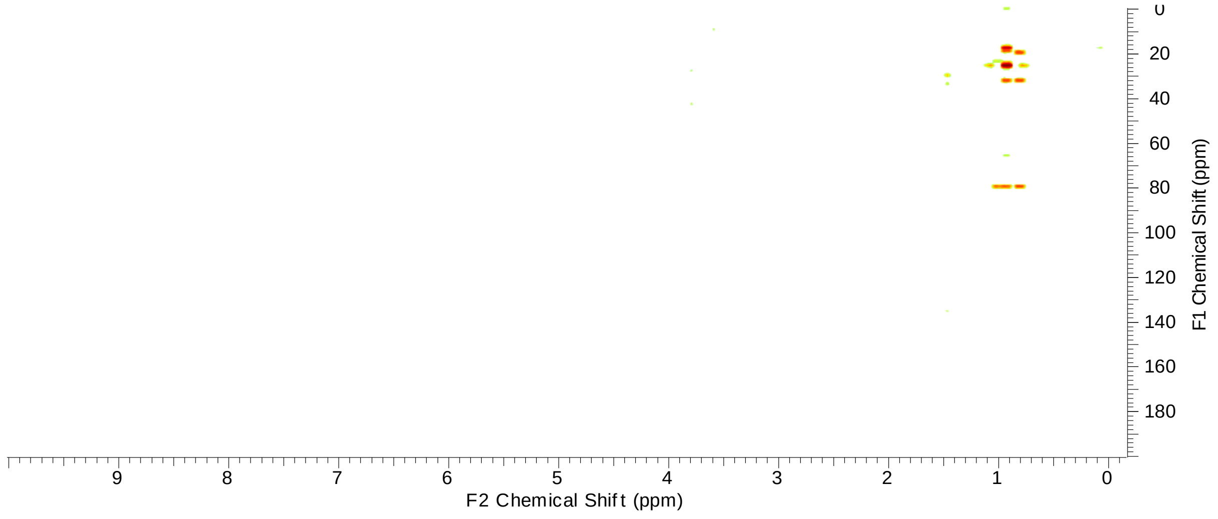

tert-Butyl(((3S,4R,5S)-5-((4R,5R)-4,5-dicyclohexyl-1,3,2-dioxaborolan-2-yl)-2,4-dimethyloct-7-yn-3-yl)oxy)dimethylsilane (14)

^{1}H-NMR (400 MHz, $CDCl_3$):

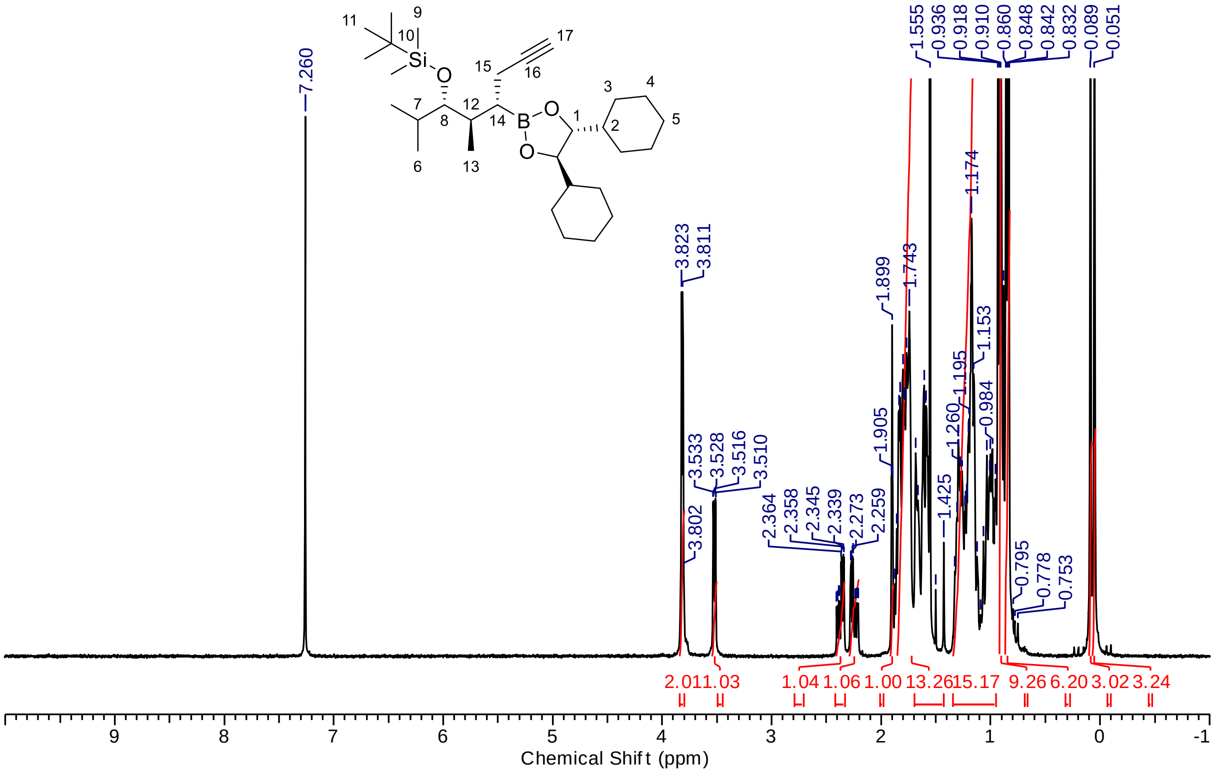

^{13}C-NMR (100 MHz, $CDCl_3$):

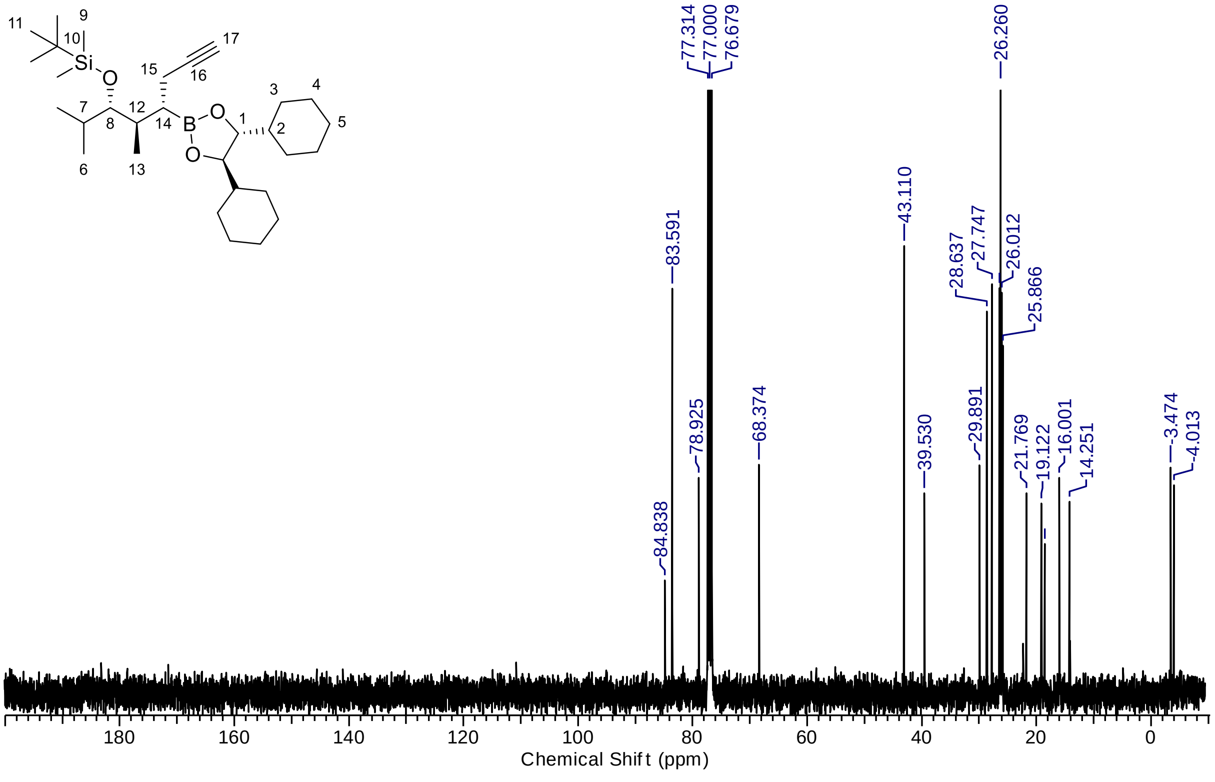

$^{1}H,^{1}H$-COSY

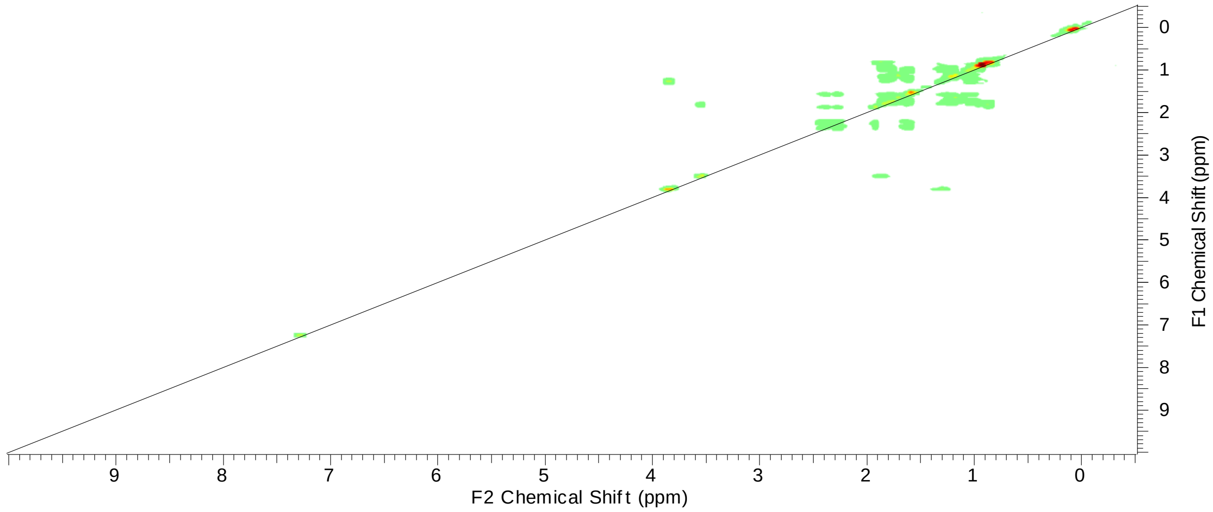

$^{1}H,^{13}C$-HSQC

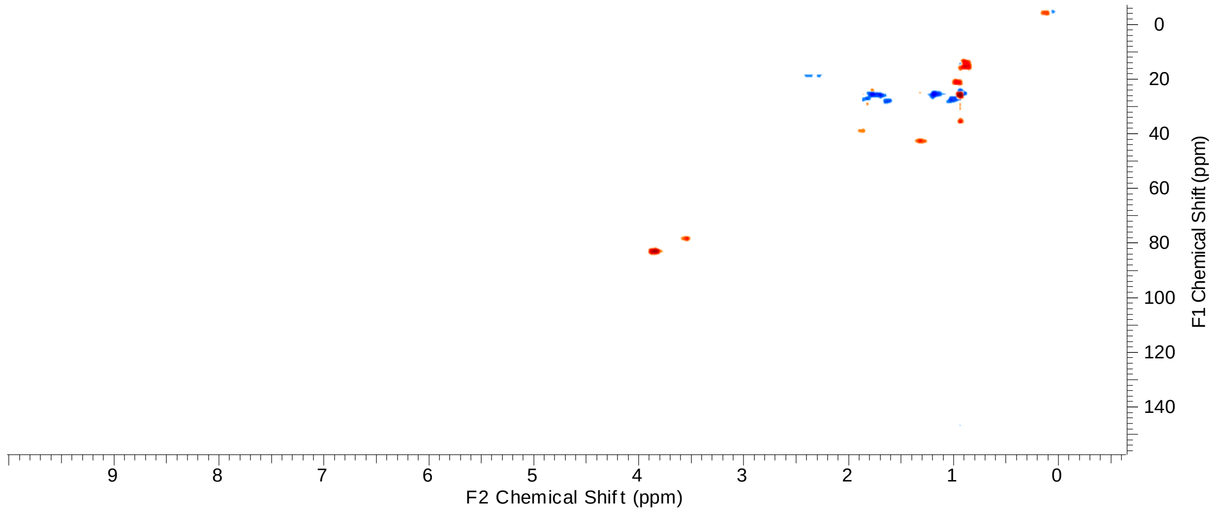

$^{1}H,^{13}C$-HMBC

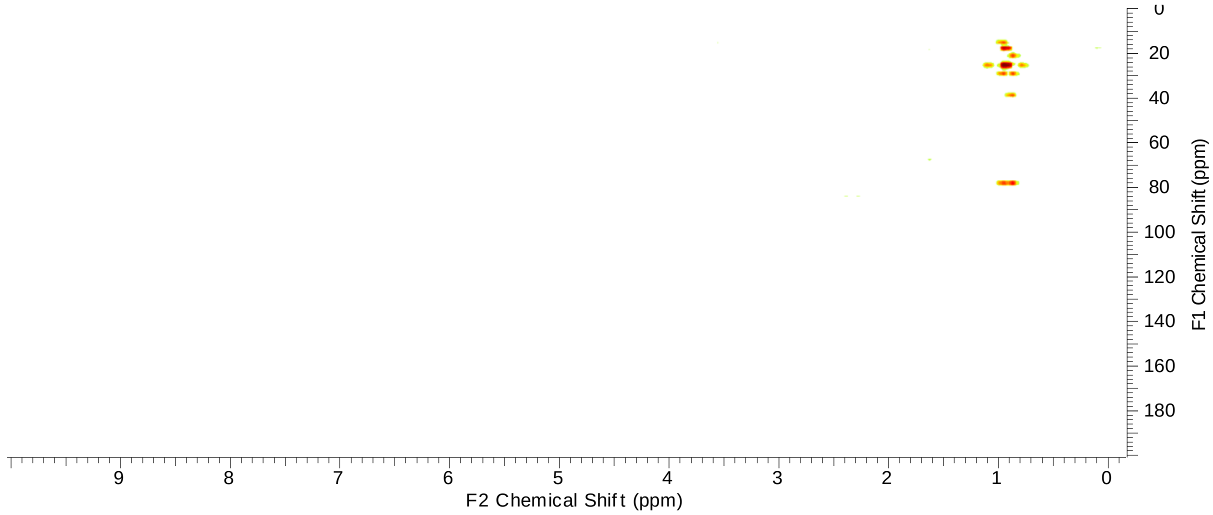

***tert*-Butyl(((3*S*,4*R*,5*S*,*E*)-5-((4*R*,5*R*)-4,5-dicyclohexyl-1,3,2-dioxaborolan-2-yl)-8-iodo-2,4-dimethyloct-7-en-3-yl)oxy)dimethylsilane (15)**

^{1}H-NMR (400 MHz, C_6D_6):

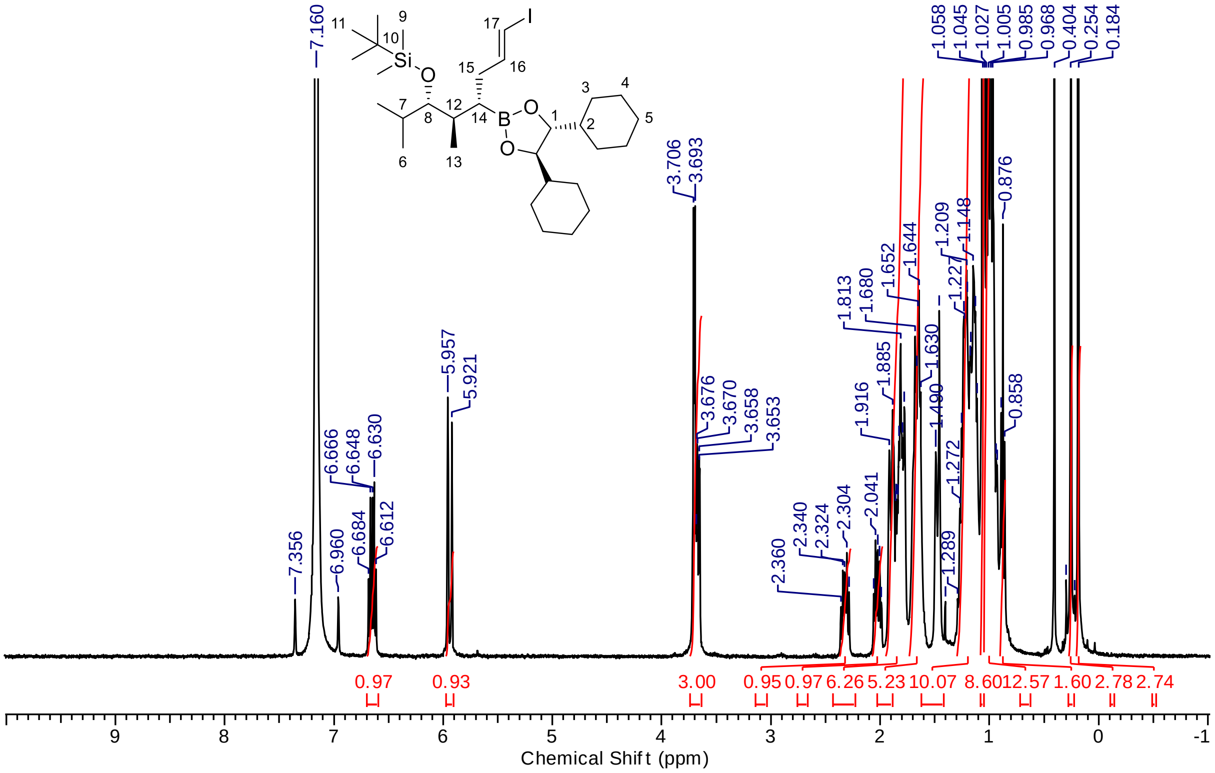

^{13}C-NMR (100 MHz, C_6D_6):

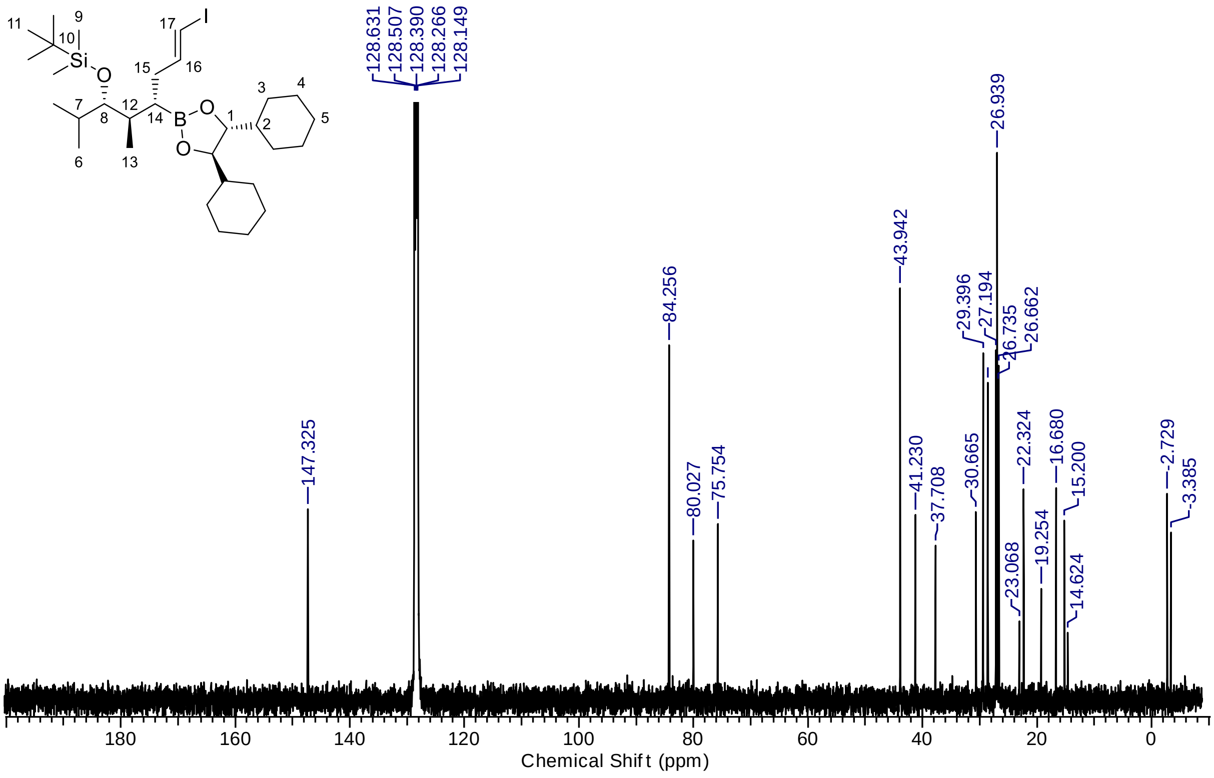

$^{1}H,^{1}H$-COSY

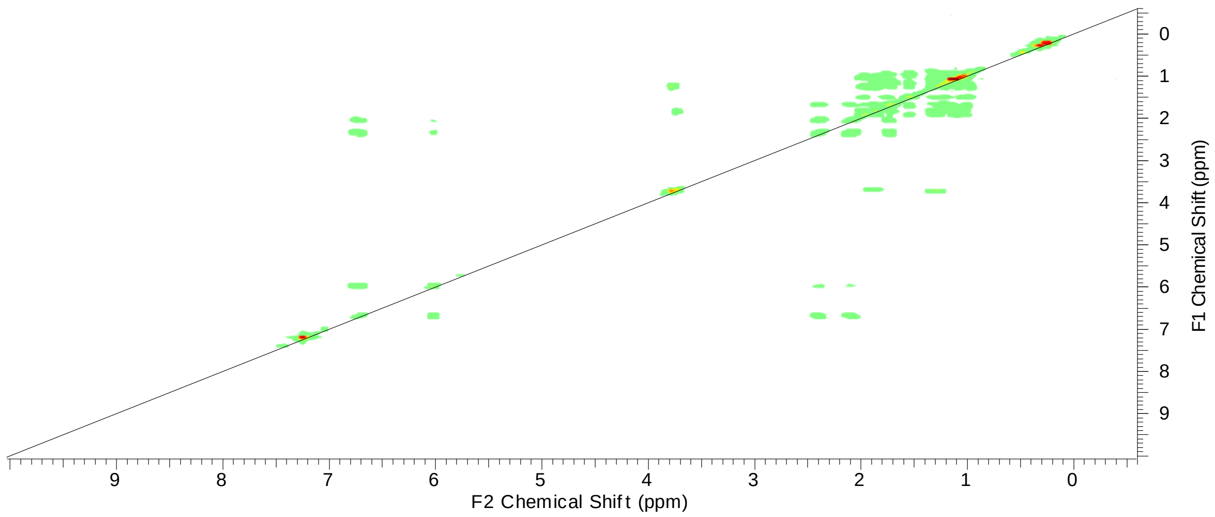

$^{1}H,^{13}C$-HSQC

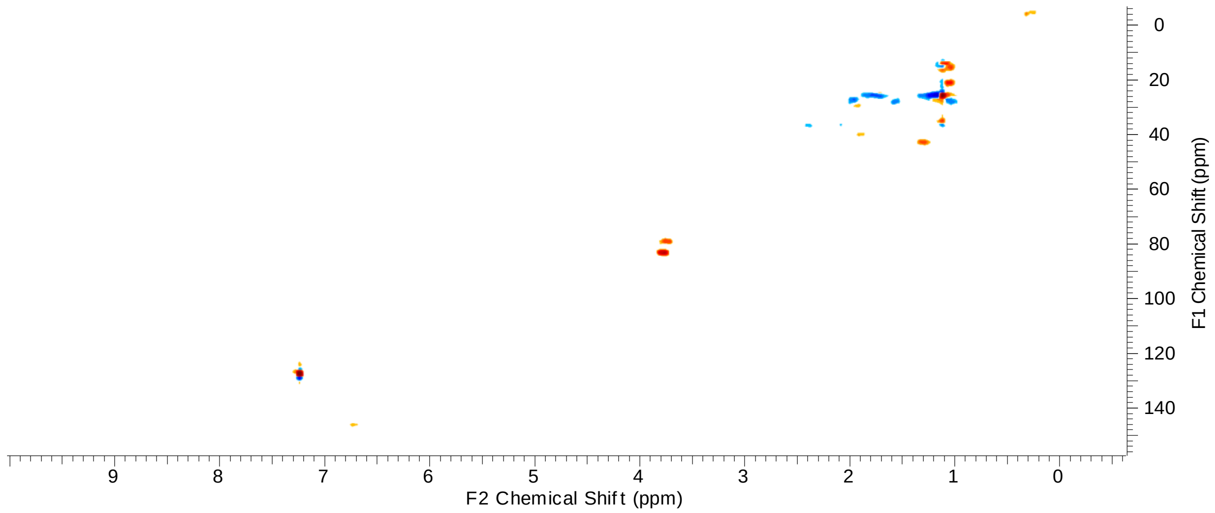

$^{1}H,^{13}C$-HMBC

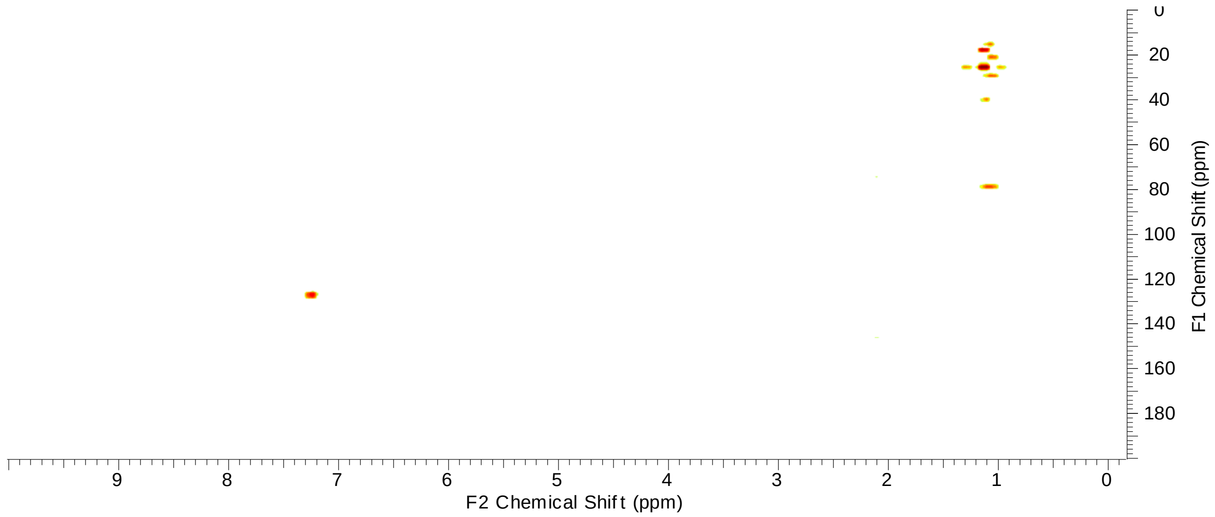

(4*R*,5*R*)-4,5-Dicyclohexyl-2-((*R*)-1-((4-methoxybenzyl)oxy)-3-phenylpropyl)-1,3,2-dioxaborolane (16)

^{1}H-NMR (500 MHz, $CDCl_3$):

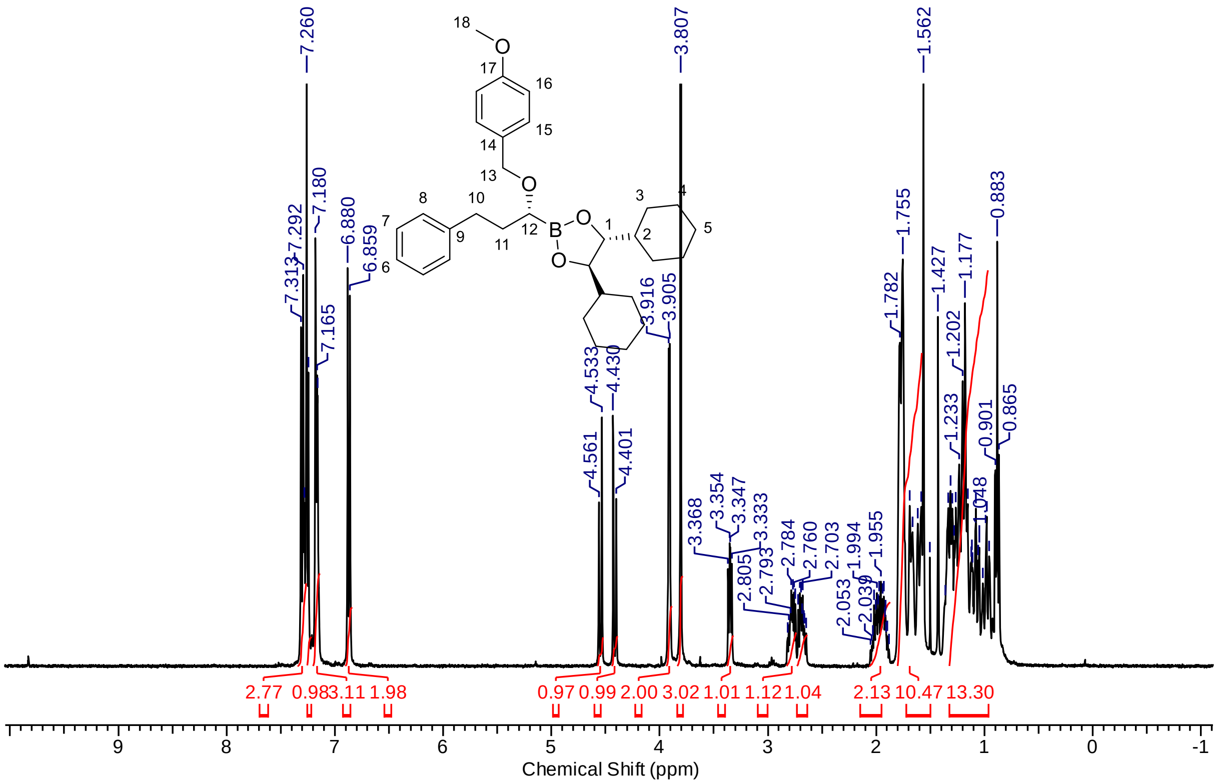

^{13}C-NMR (125 MHz, $CDCl_3$):

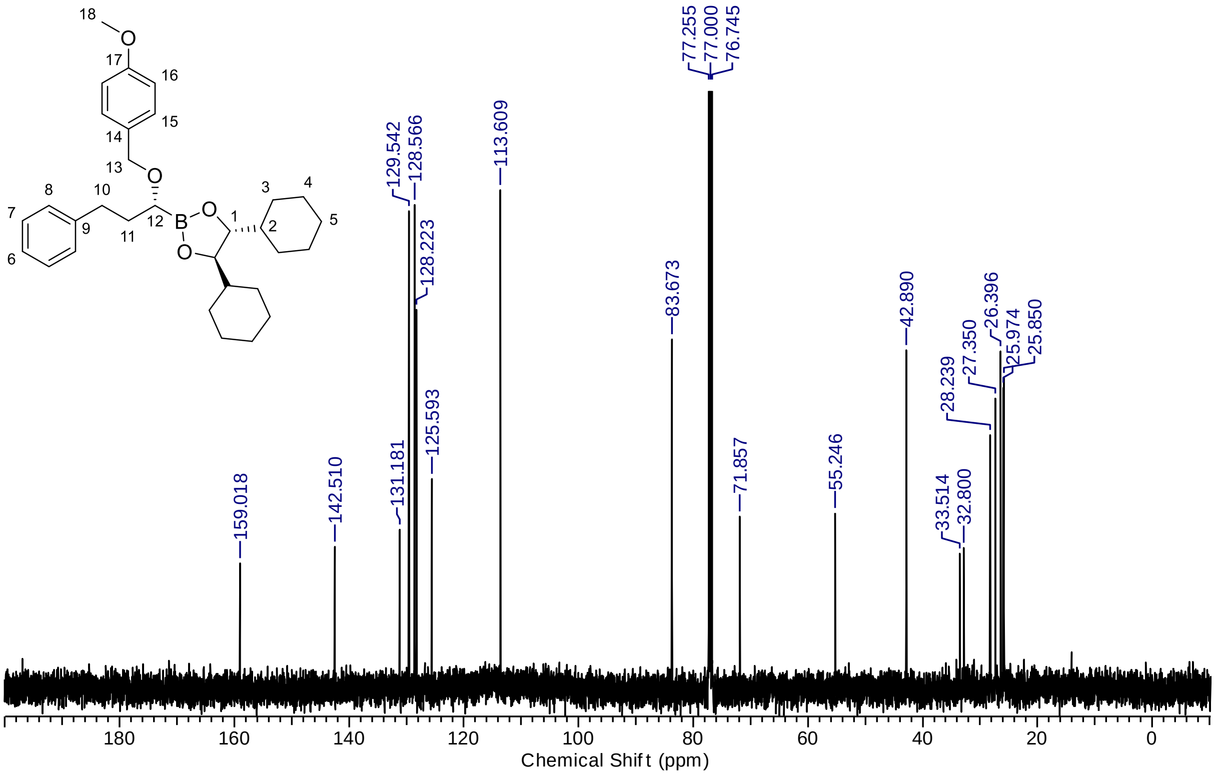

$^{1}H,^{1}H$-COSY

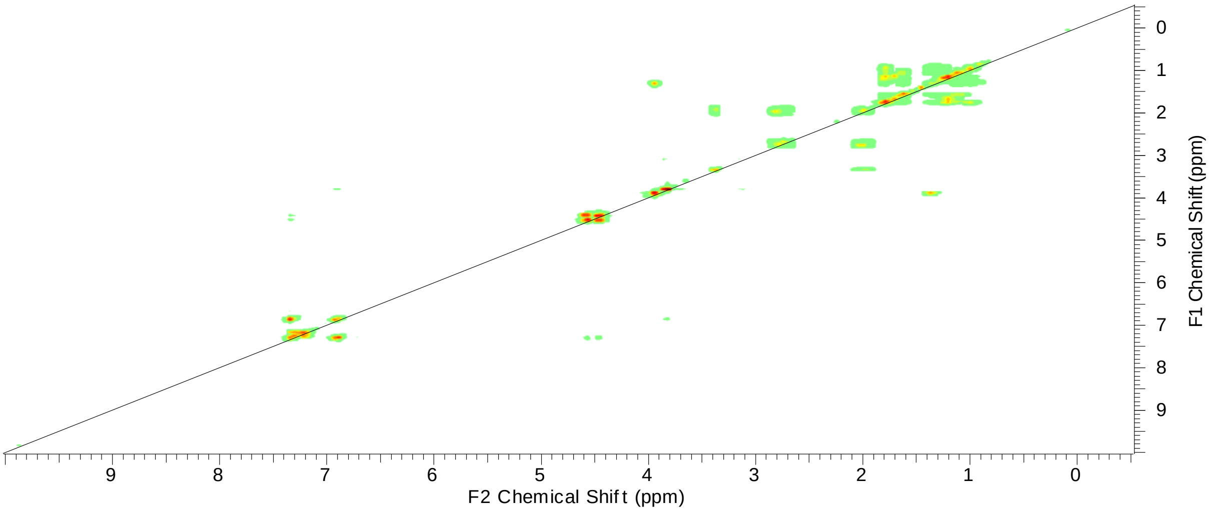

$^{1}H,^{13}C$-HSQC

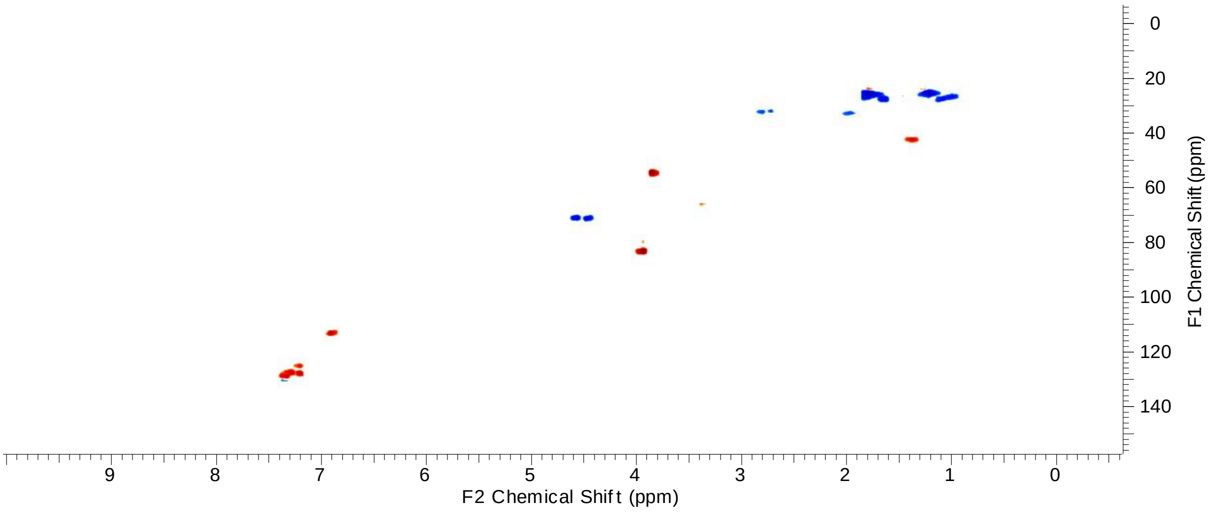

$^{1}H,^{13}C$-HMBC

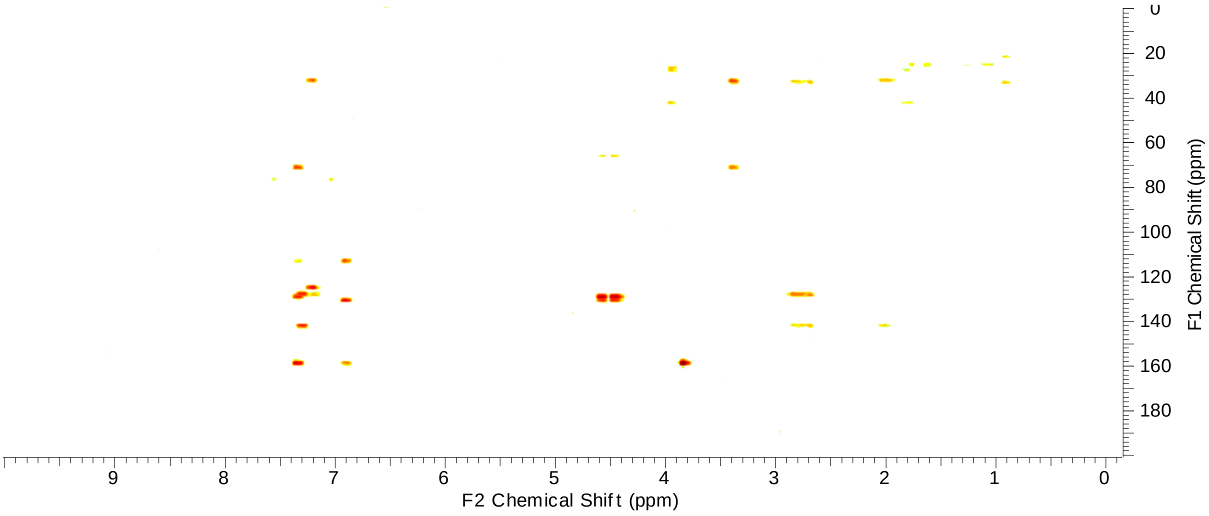

((4*S*,5*S*)-4-((4*R*,5*R*)-4,5-Dicyclohexyl-1,3,2-dioxaborolan-2-yl)-5-((4-methoxybenzyl)oxy)-7-phenylhept-1-yn-1-yl)trimethylsilane (17)

^{1}H-NMR (400 MHz, $CDCl_3$):

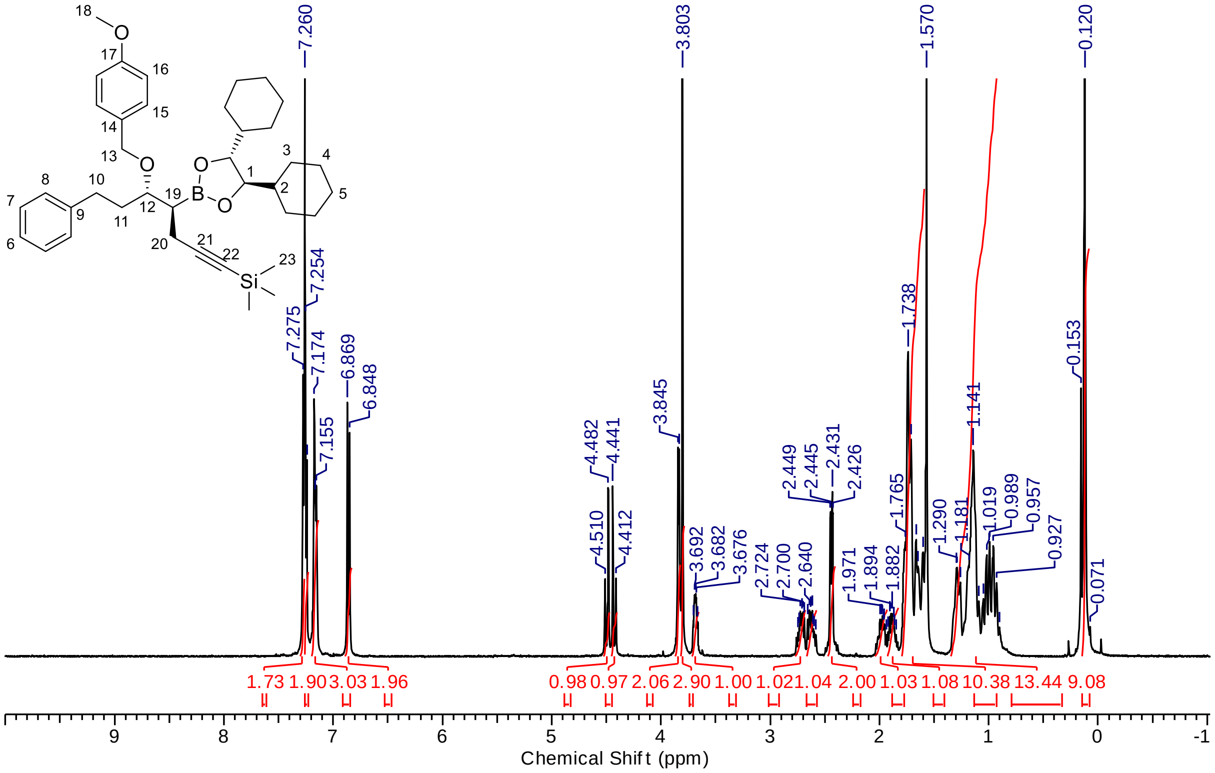

^{13}C-NMR (100 MHz, $CDCl_3$):

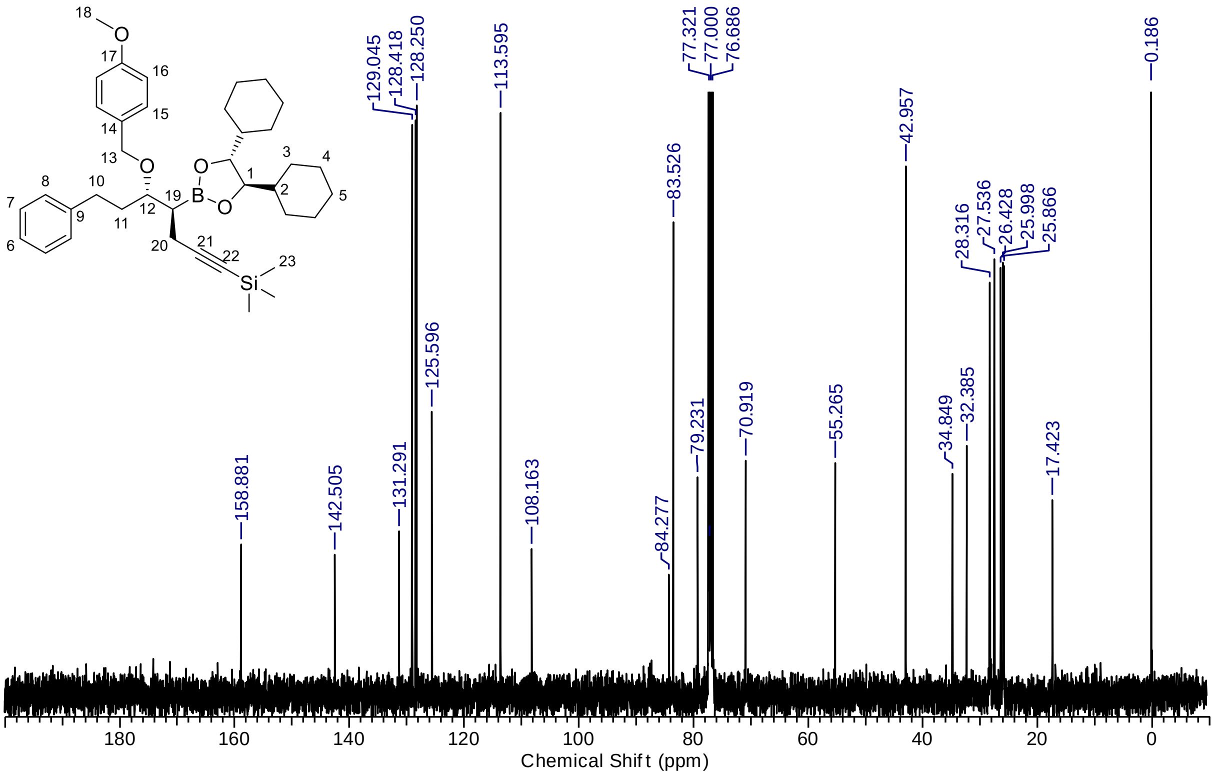

$^{1}H,^{1}H$-COSY

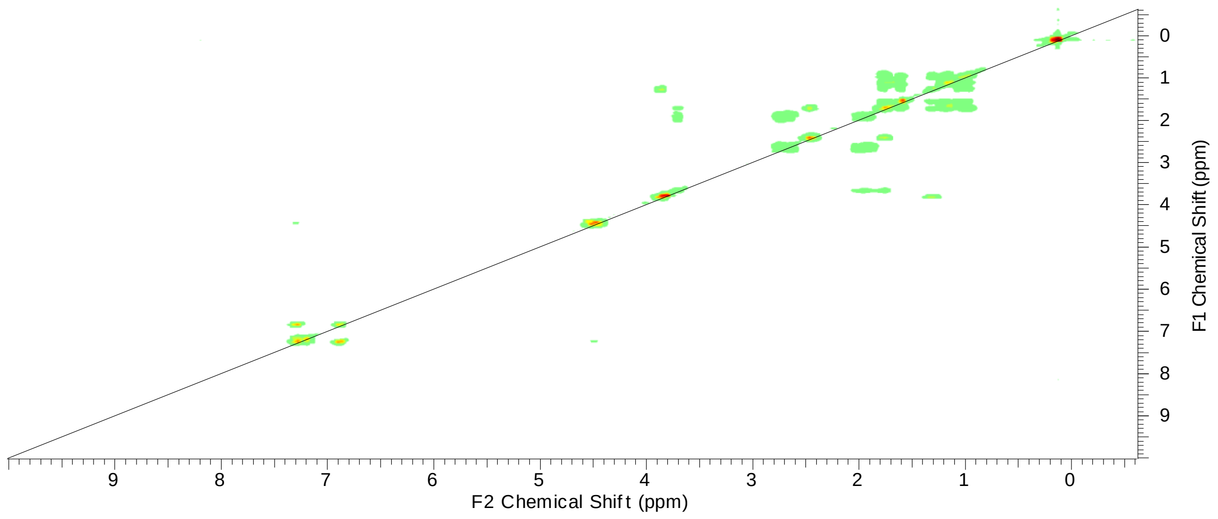

$^{1}H,^{13}C$-HSQC

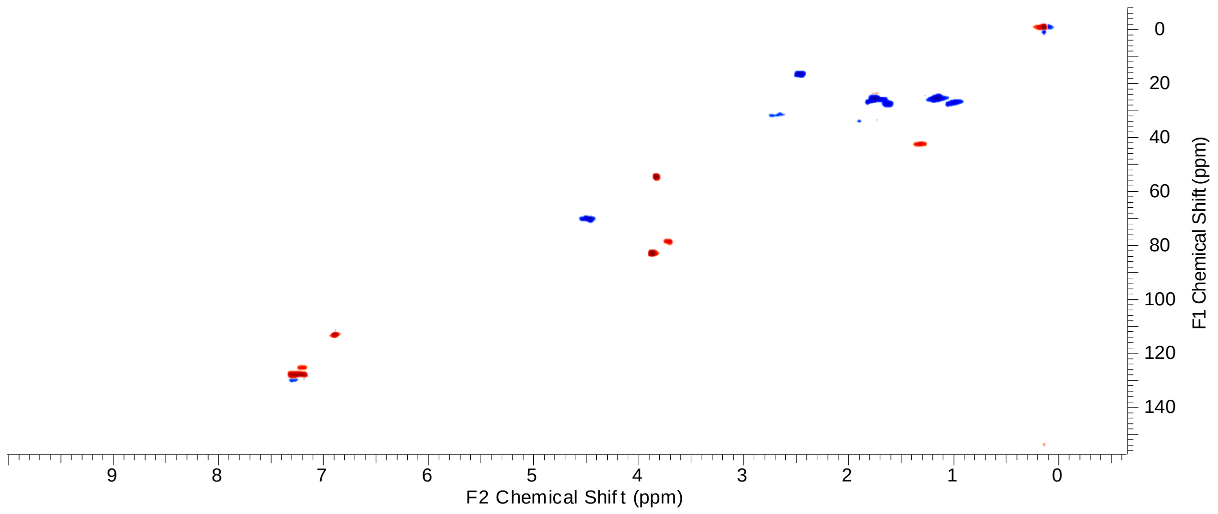

$^{1}H,^{13}C$-HMBC

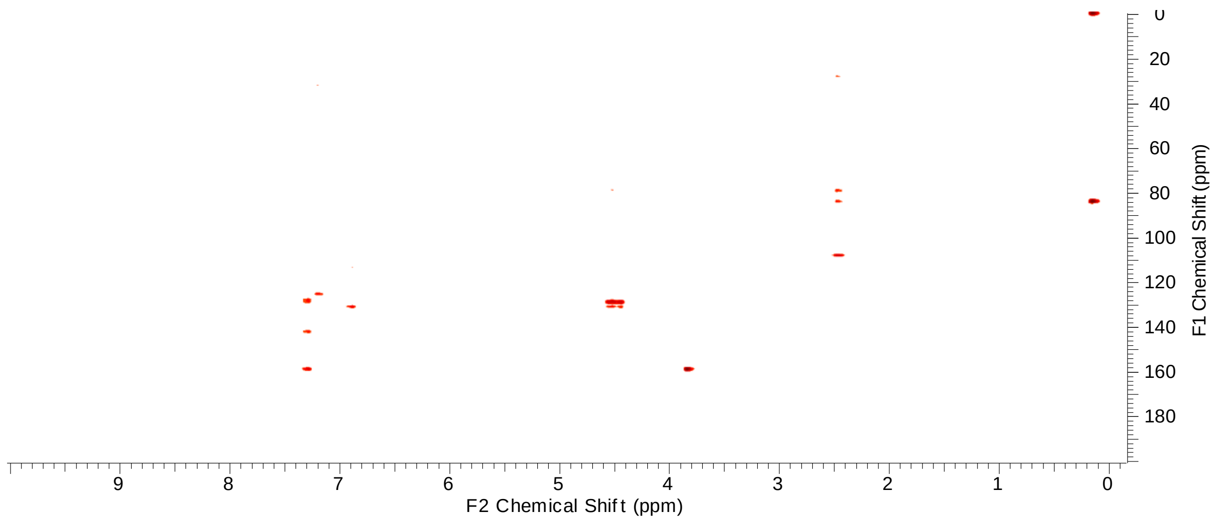

(4*R*,5*R*)-4,5-Dicyclohexyl-2-((4*S*,5*S*)-5-((4-methoxybenzyl)oxy)-7-phenylhept-1-yn-4-yl)-1,3,2-dioxaborolane (18)

^{1}H-NMR (400 MHz, $CDCl_3$):

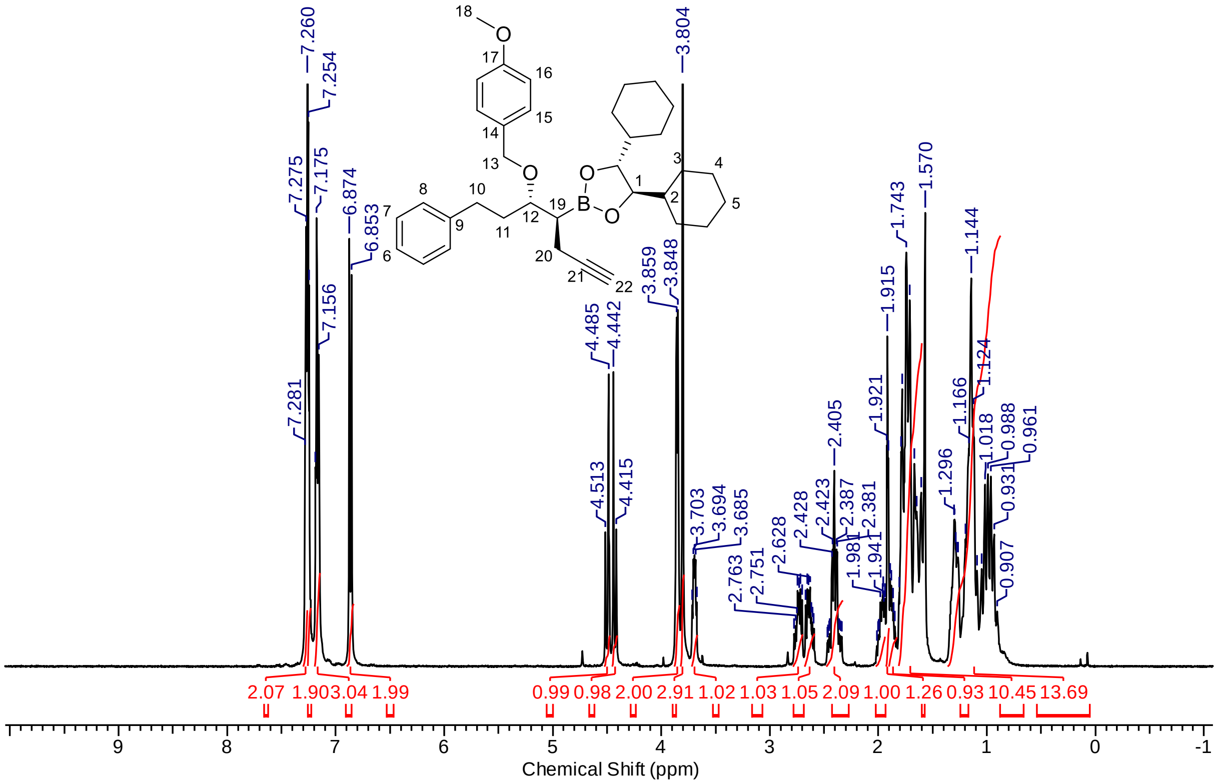

^{13}C-NMR (100 MHz, $CDCl_3$):

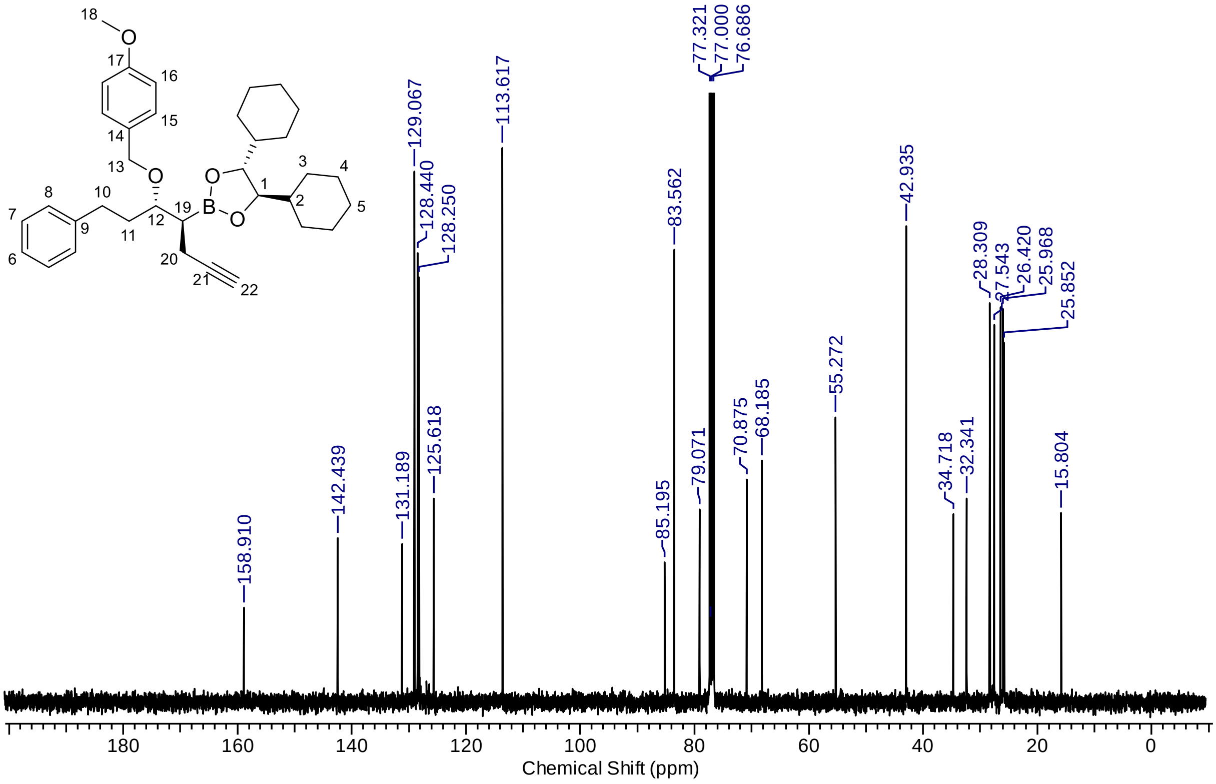

$^{1}H,^{1}H$-COSY

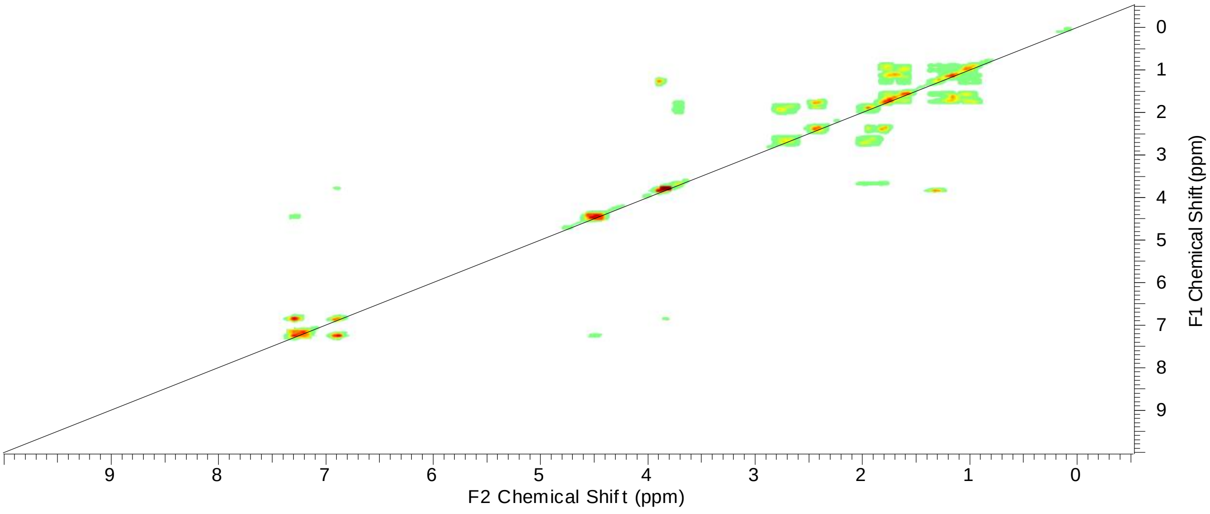

$^{1}H,^{13}C$-HSQC

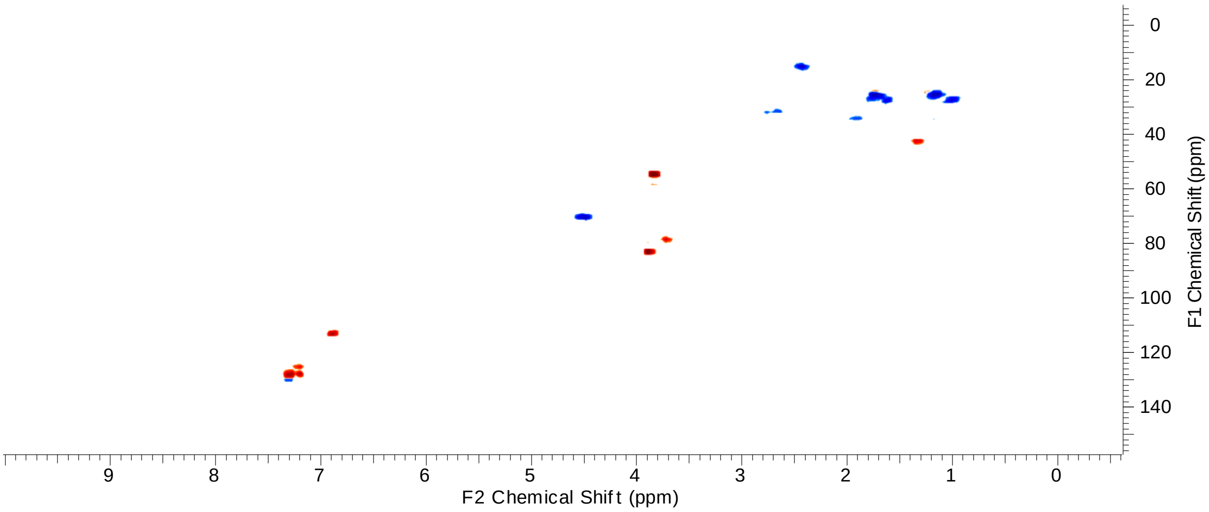

$^{1}H,^{13}C$-HMBC

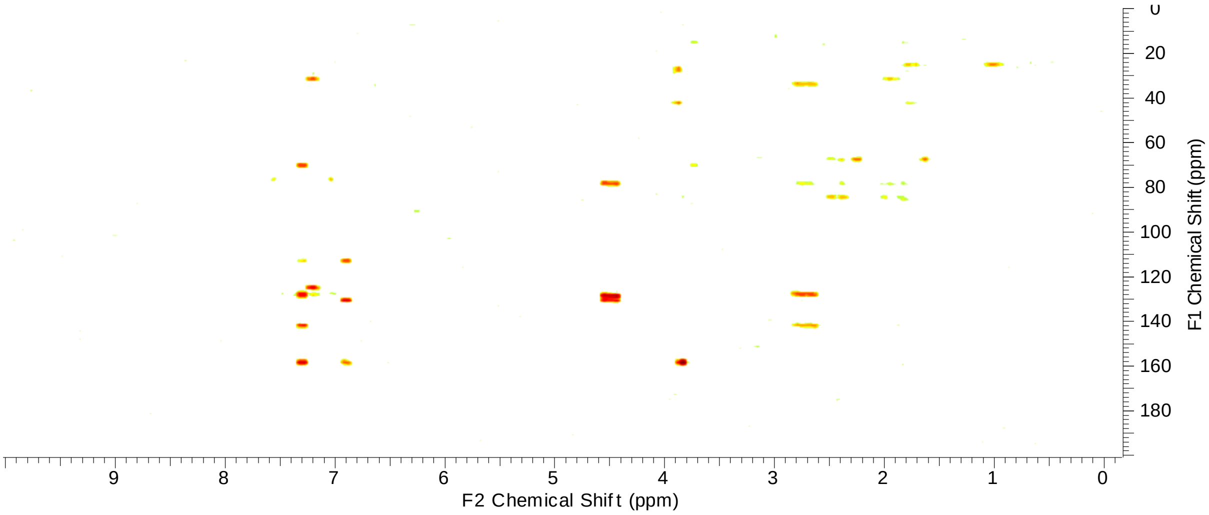

(3*S*,4*S*,*E*)-4-((4*R*,5*R*)-4,5-Dicyclohexyl-1,3,2-dioxaborolan-2-yl)-7-iodo-1-phenylhept-6-en-3-ol (19)

^{1}H-NMR (400 MHz, $CDCl_3$):

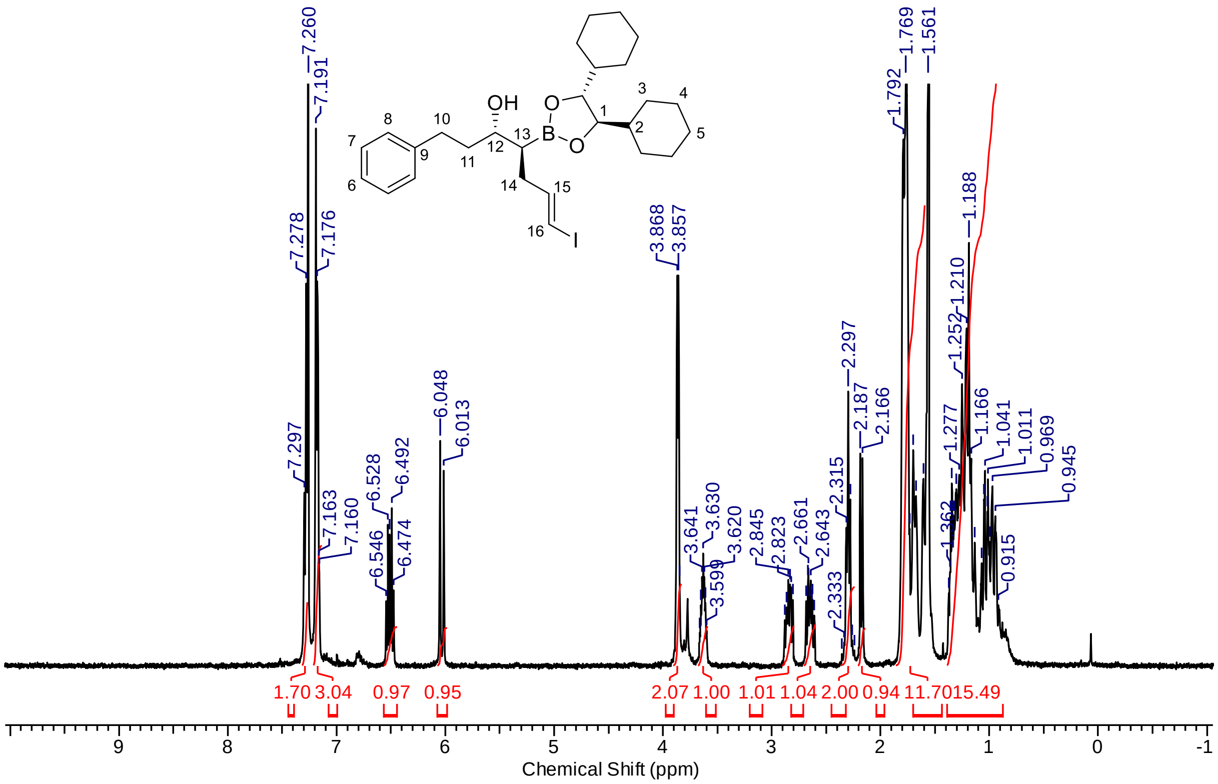

^{13}C-NMR (100 MHz, $CDCl_3$):

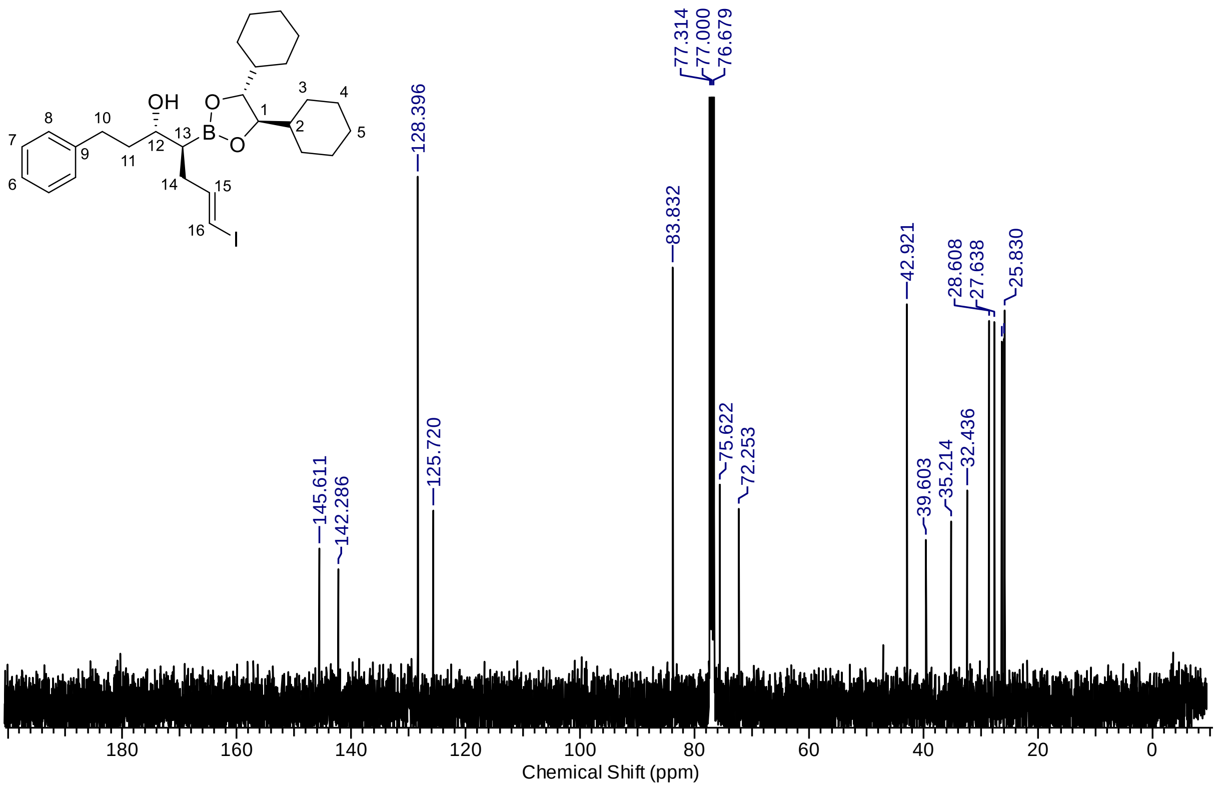

$^{1}H,^{1}H$-COSY

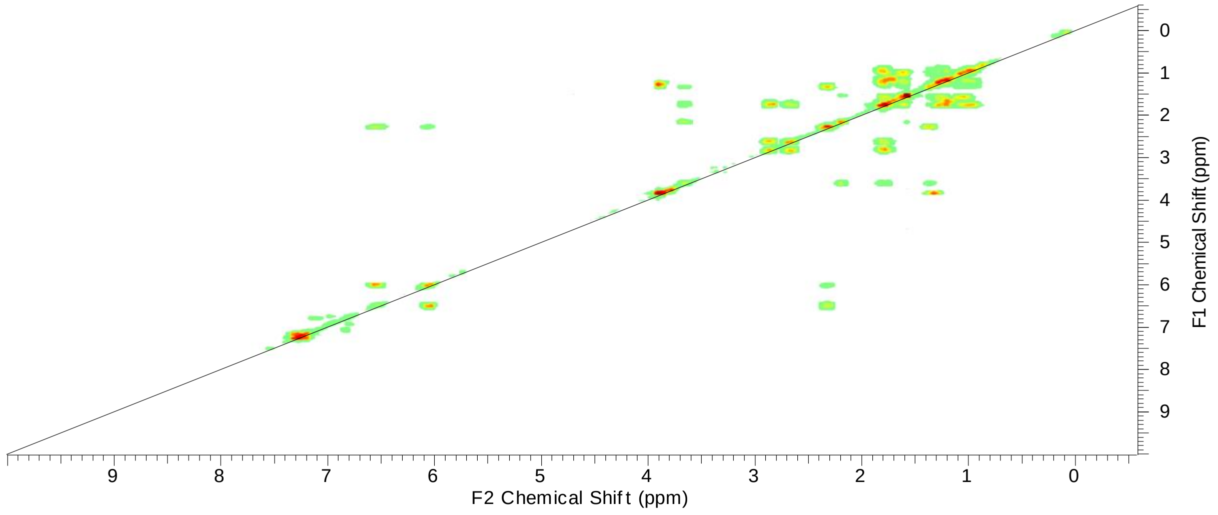

$^{1}H,^{13}C$-HSQC

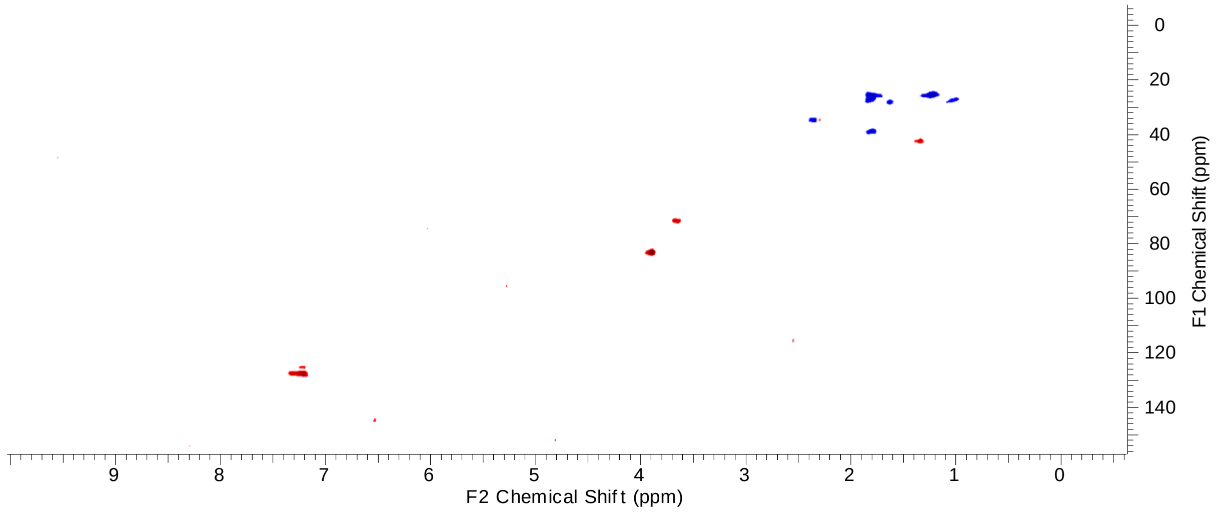

$^{1}H,^{13}C$-HMBC

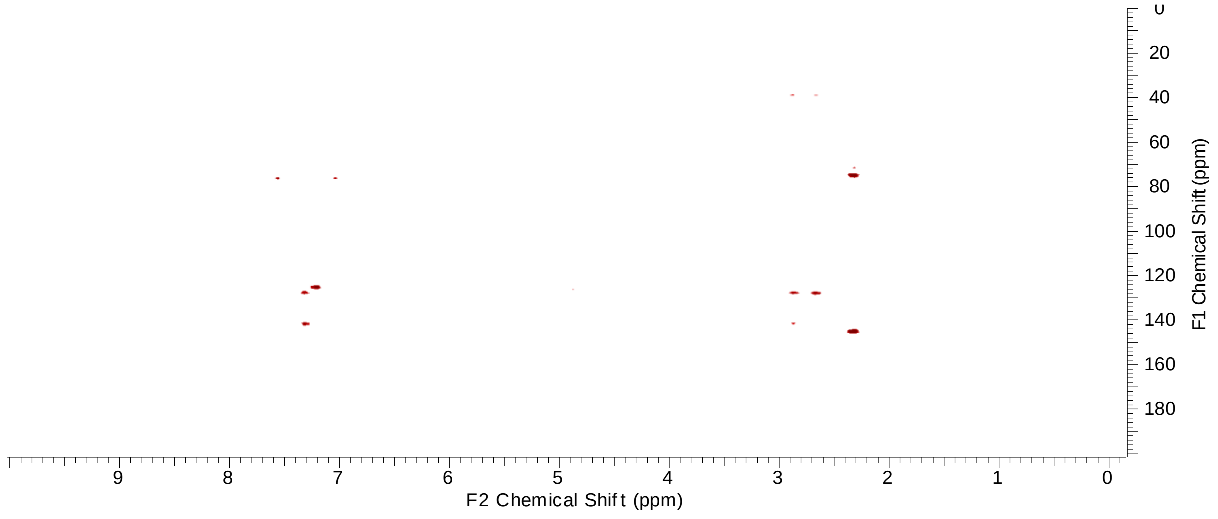

(4*S*,5*S*)-5-((4-Methoxybenzyl)oxy)-7-phenyl-1-(trimethylsilyl)hept-1-yn-4-ol (20)

^{1}H-NMR (400 MHz, $CDCl_3$):

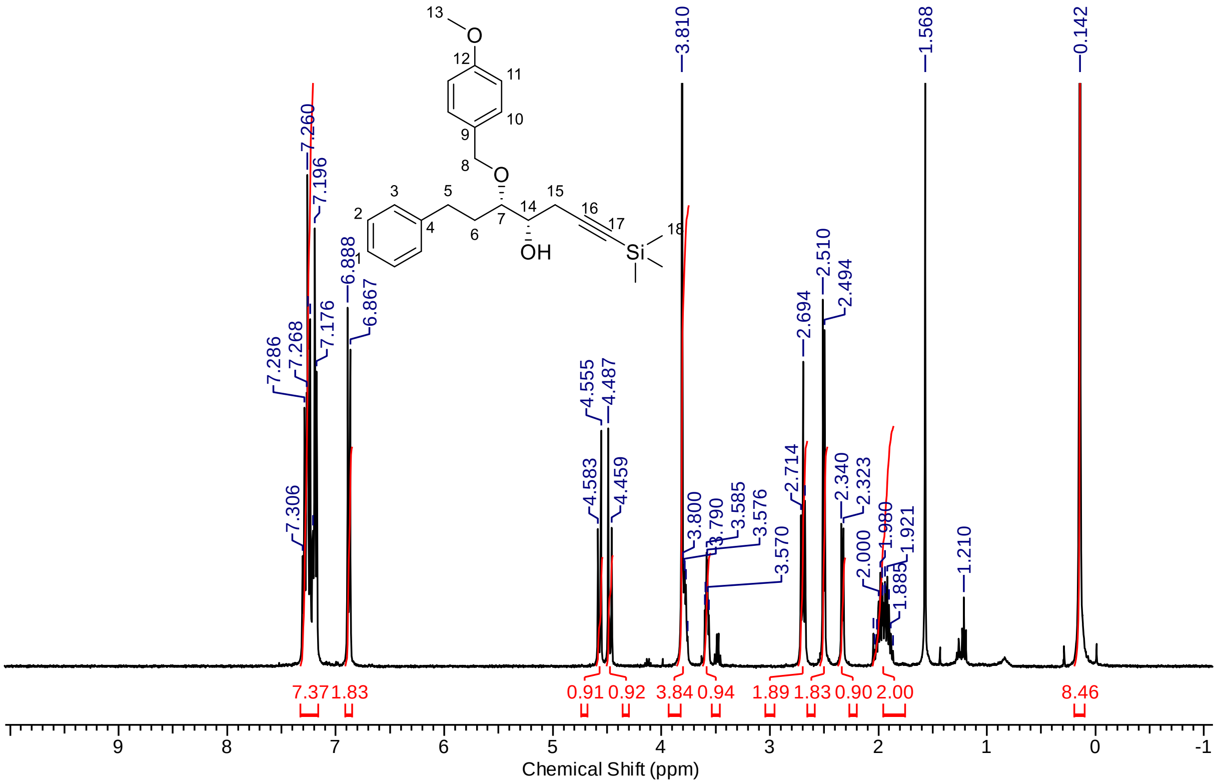

^{13}C-NMR (100 MHz, $CDCl_3$):

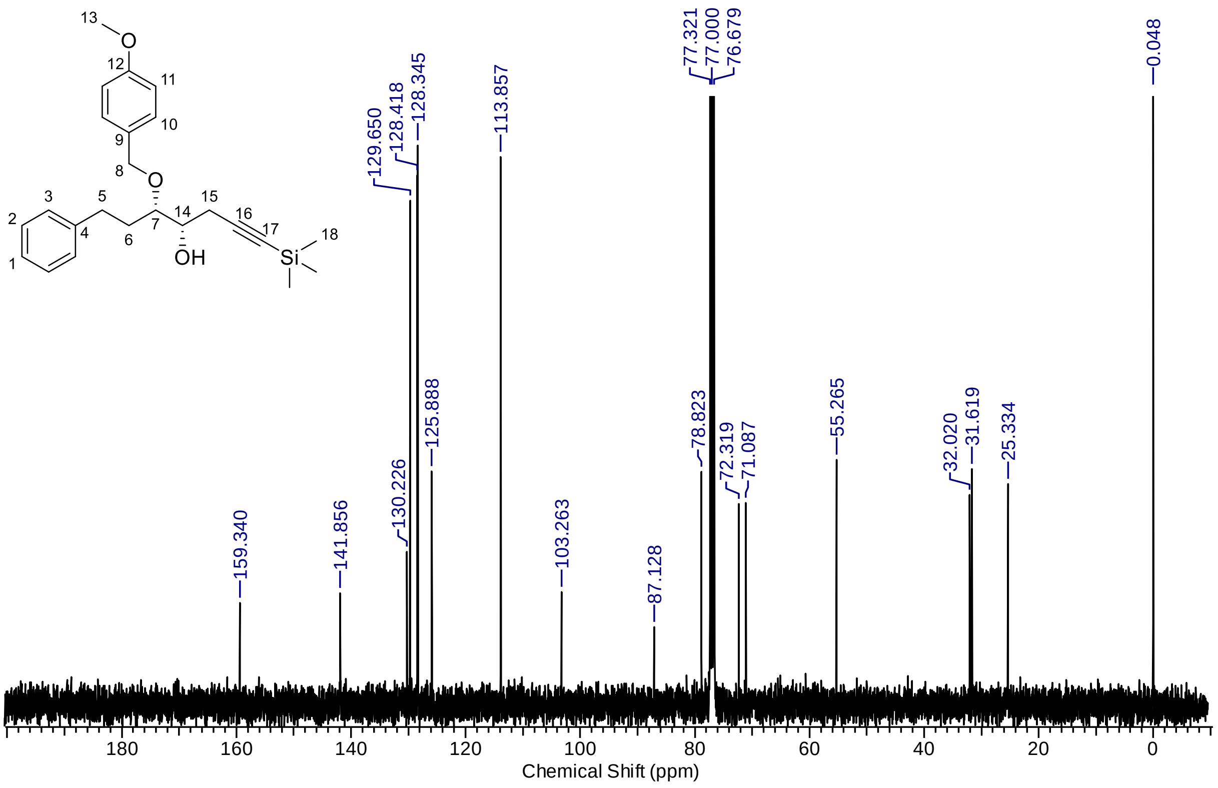

$^{1}H,^{1}H$-COSY

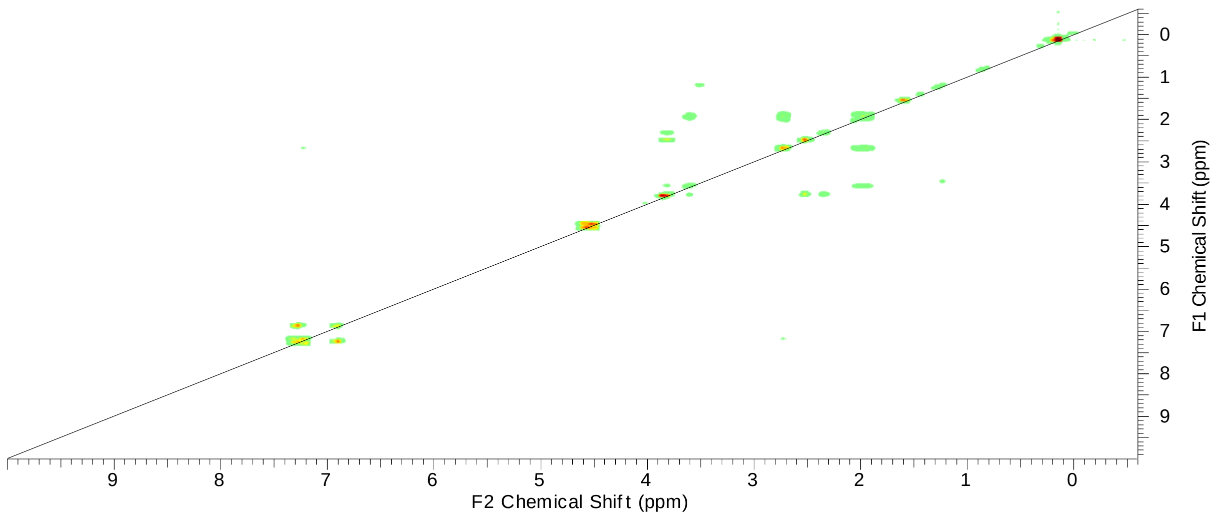

$^{1}H,^{13}C$-HSQC

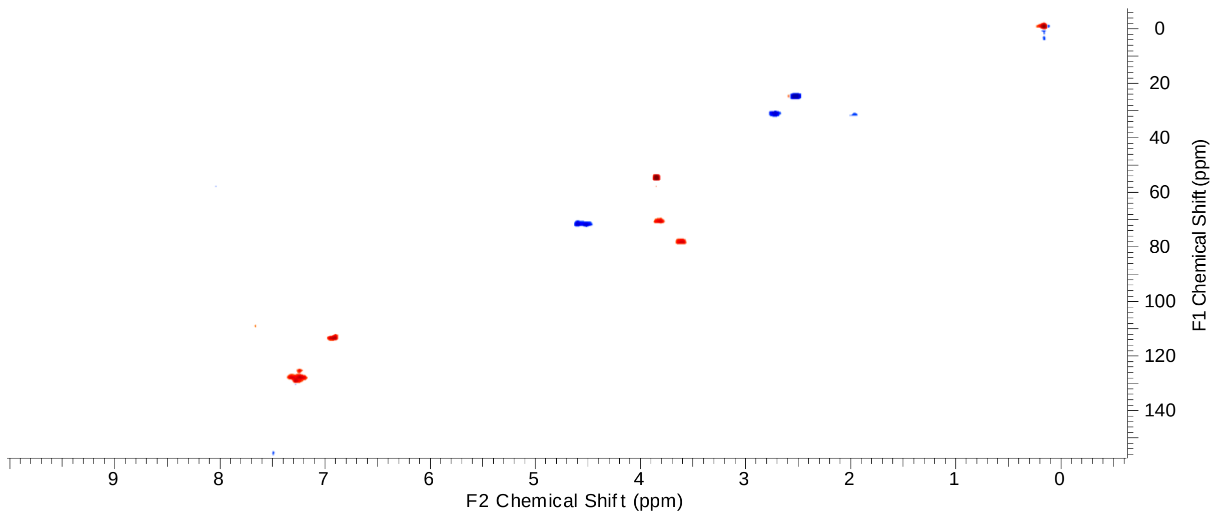

$^{1}H,^{13}C$-HMBC

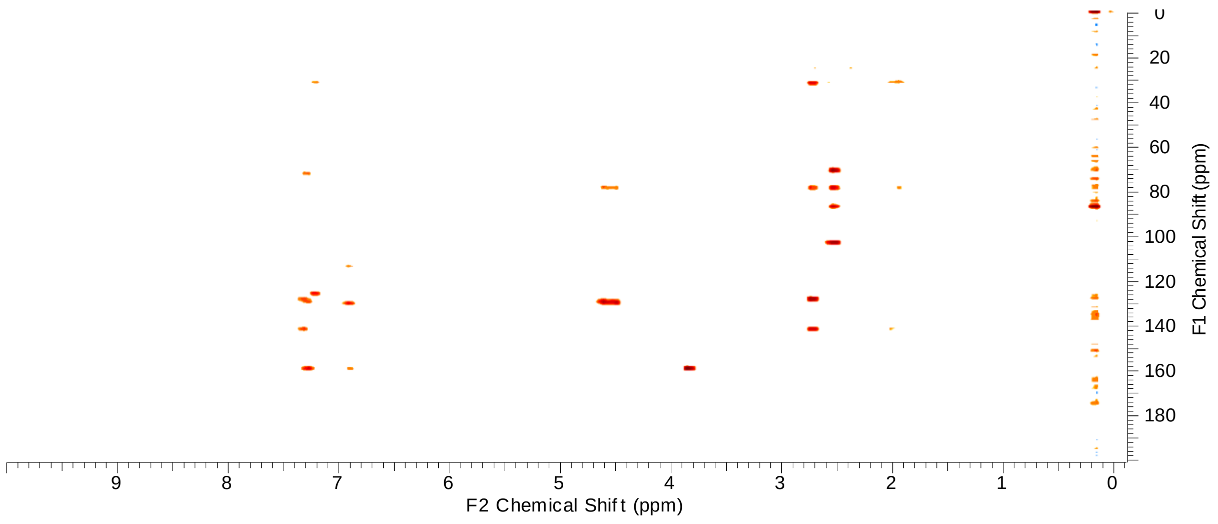

(4*S*,5*S*)-5-((4-Methoxybenzyl)oxy)-7-phenylhept-1-yn-4-ol (S2)

^{1}H-NMR (400 MHz, $CDCl_3$):

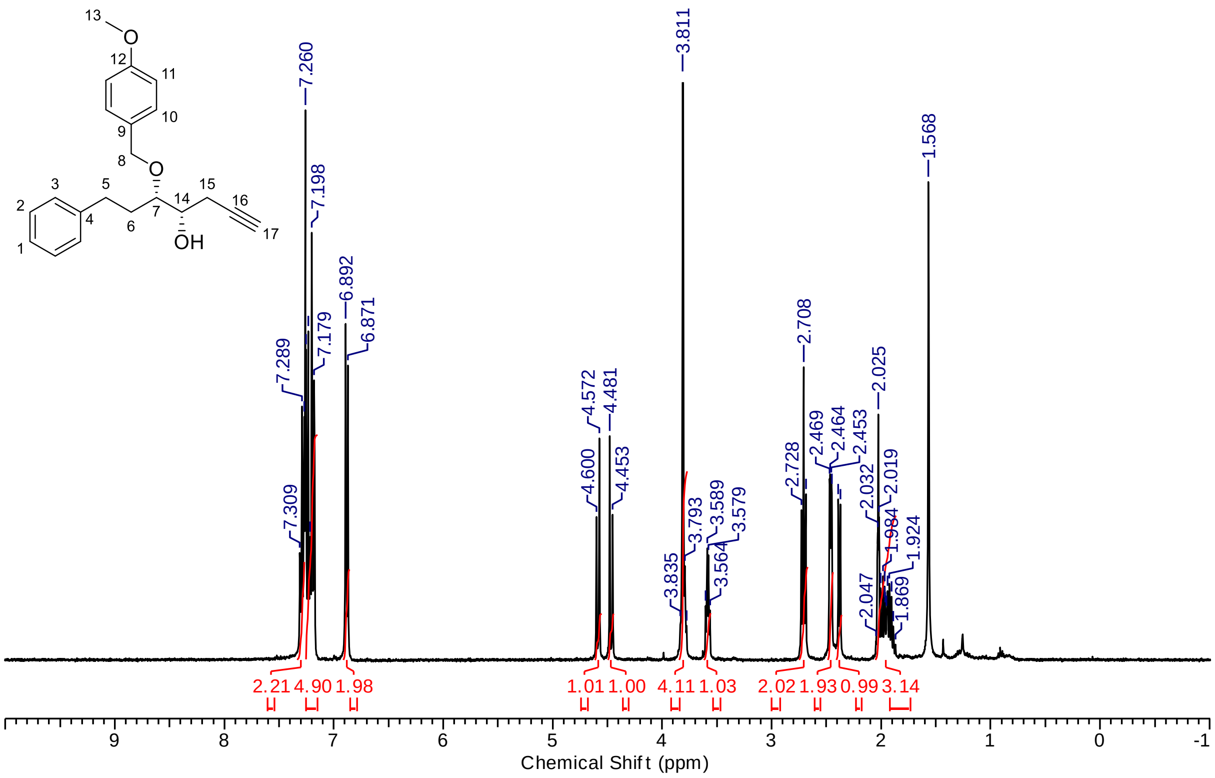

^{13}C-NMR (100 MHz, $CDCl_3$):

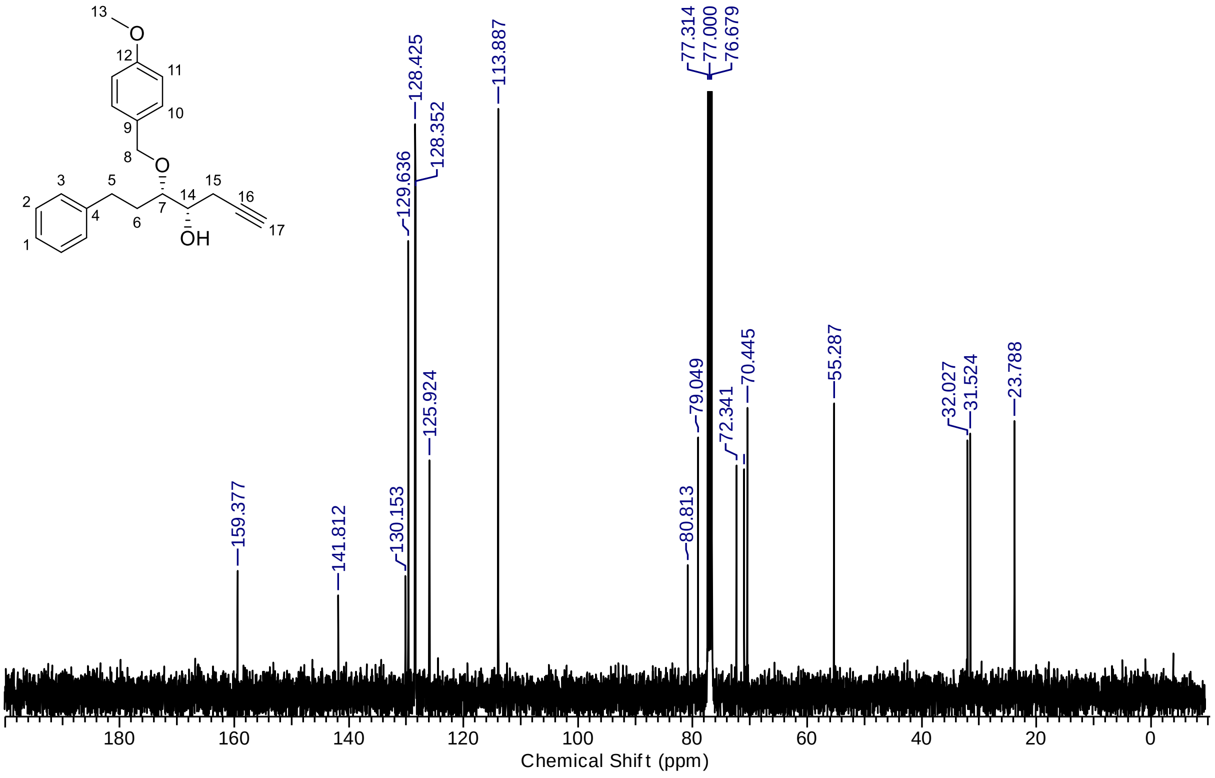

$^{1}H,^{1}H$-COSY

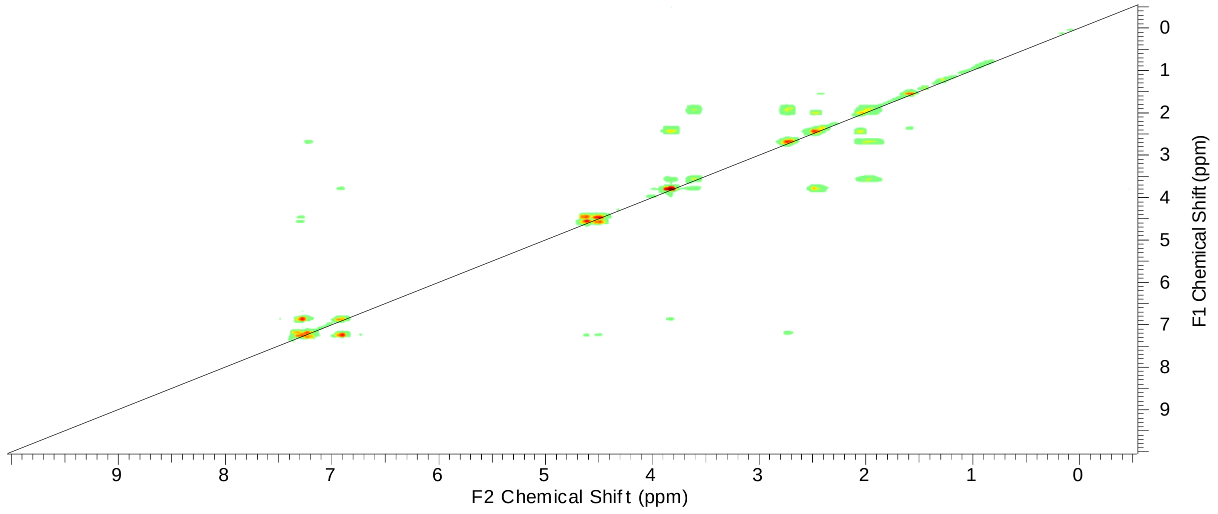

$^{1}H,^{13}C$-HSQC

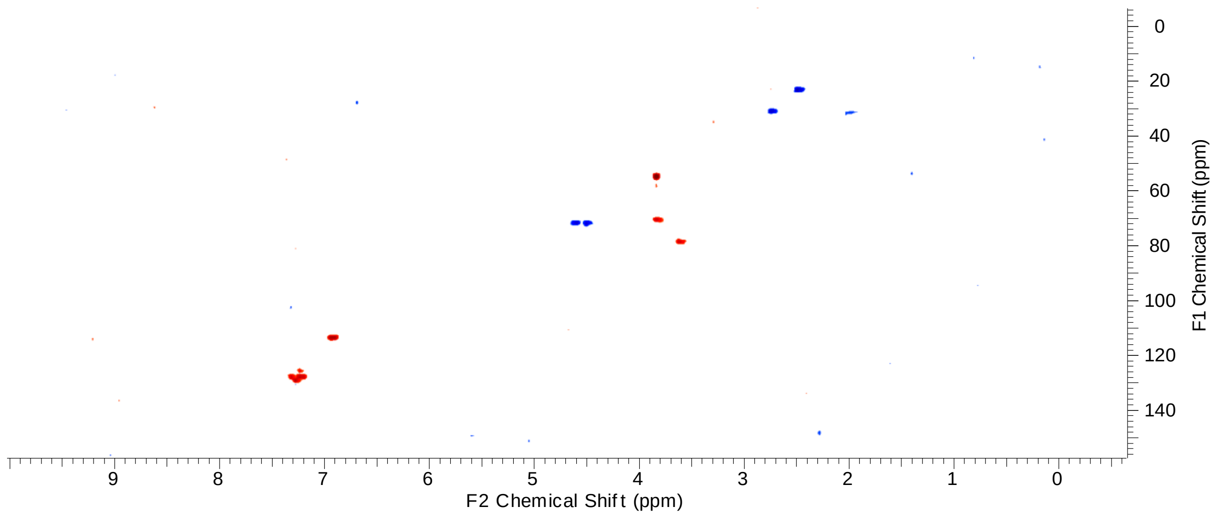

$^{1}H,^{13}C$-HMBC

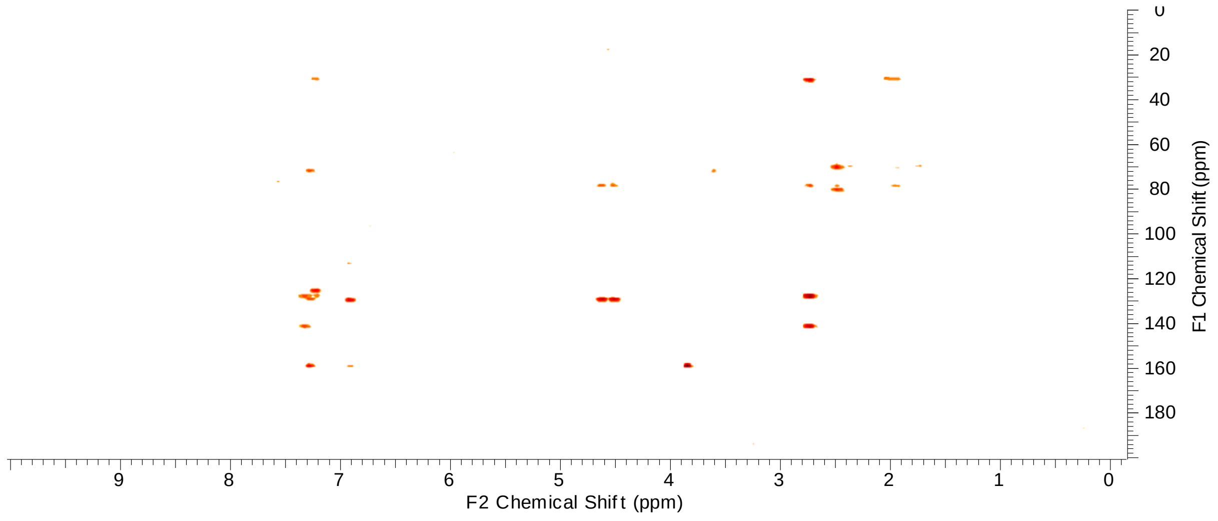

***tert*-Butyl(((4*S*,5*S*)-5-((4-methoxybenzyl)oxy)-7-phenylhept-1-yn-4-yl)oxy)dimethylsilane (21)**

^{1}H-NMR (400 MHz, $CDCl_3$):

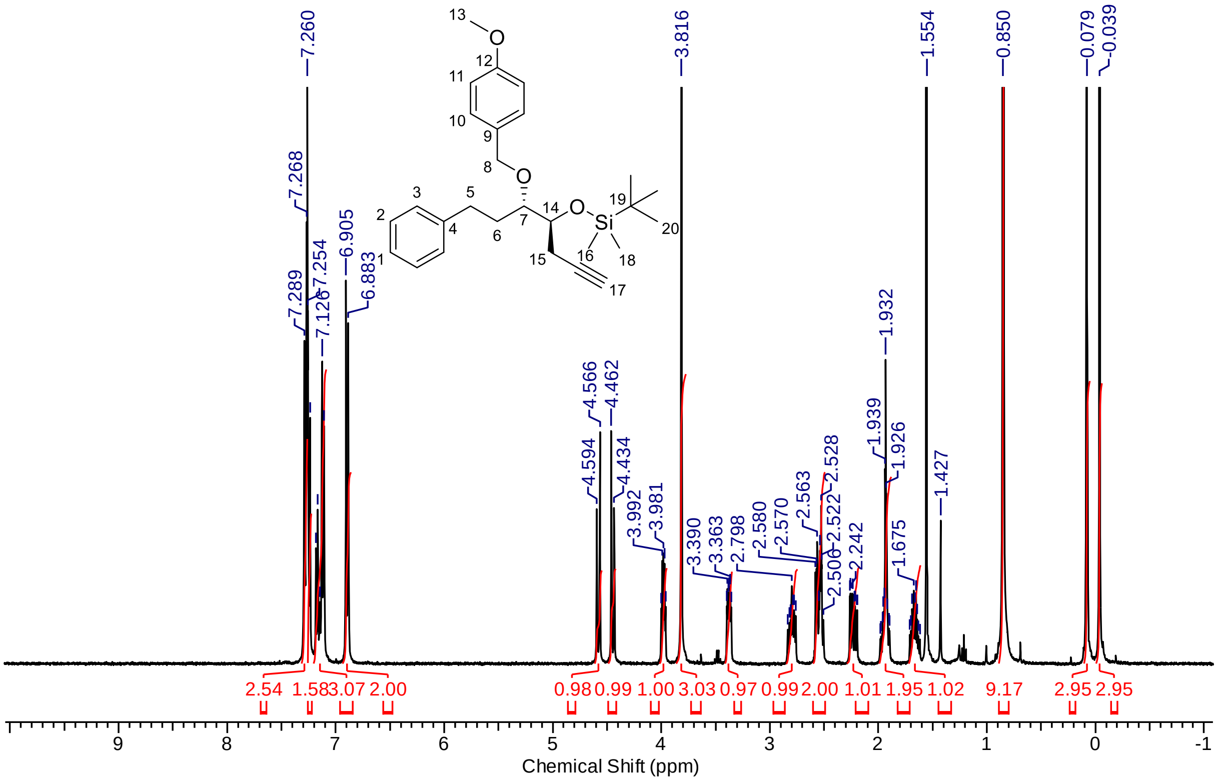

^{13}C-NMR (100 MHz, $CDCl_3$):

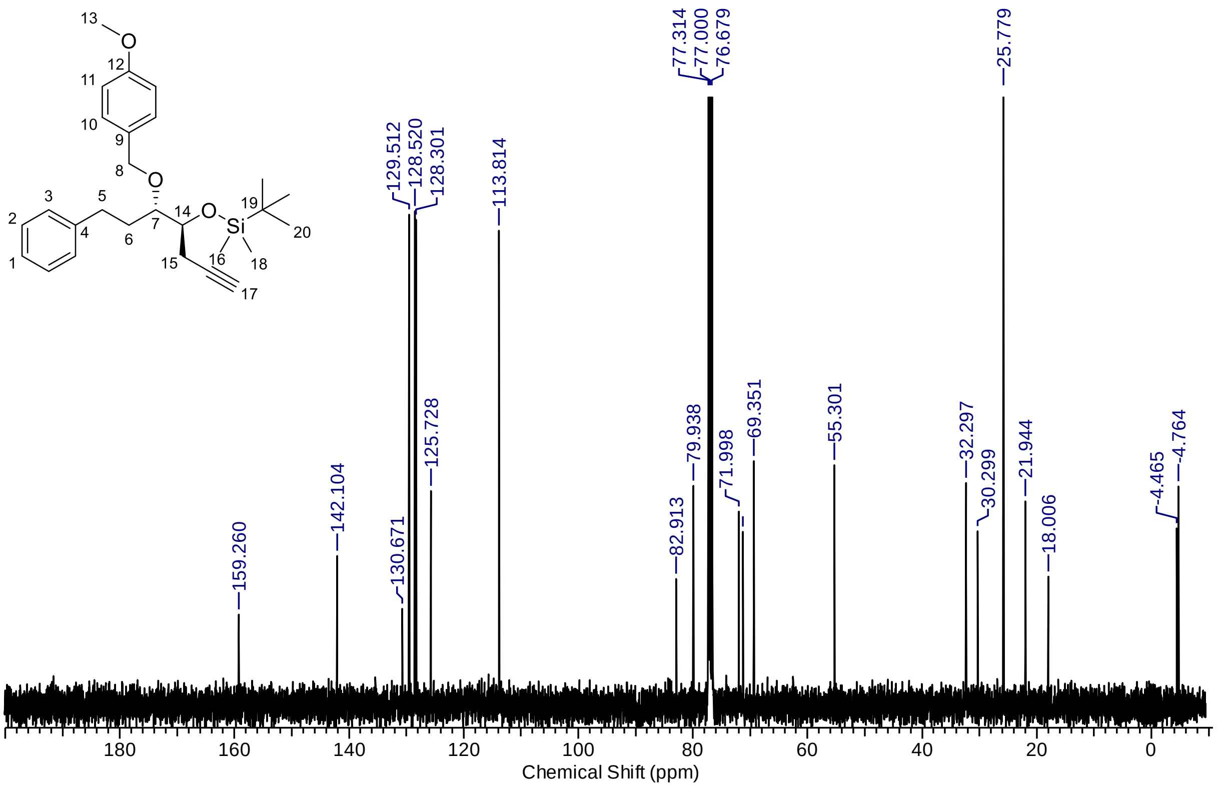

$^{1}H,^{1}H$-COSY

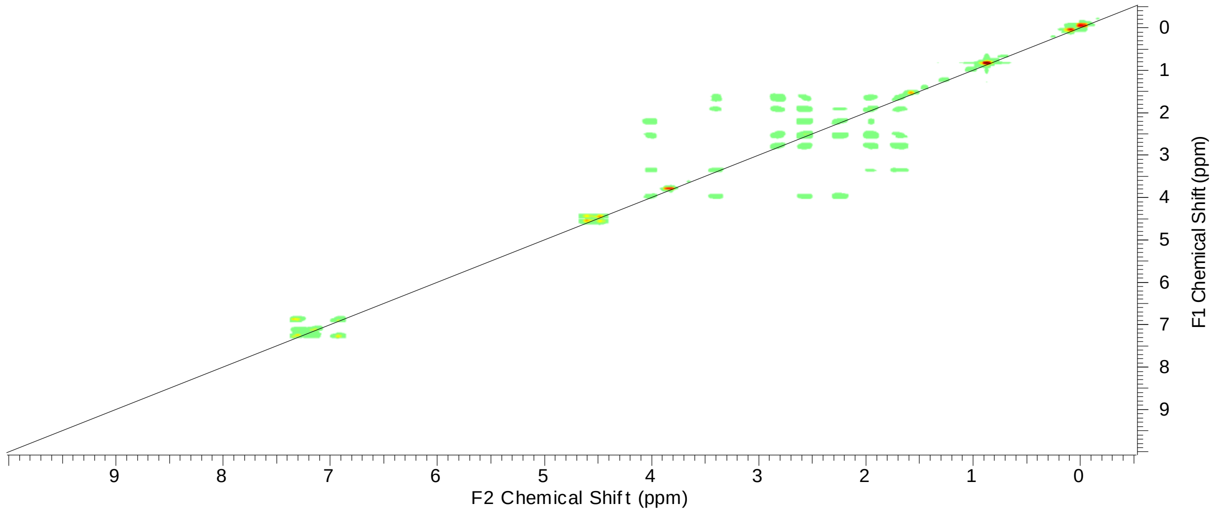

$^{1}H,^{13}C$-HSQC

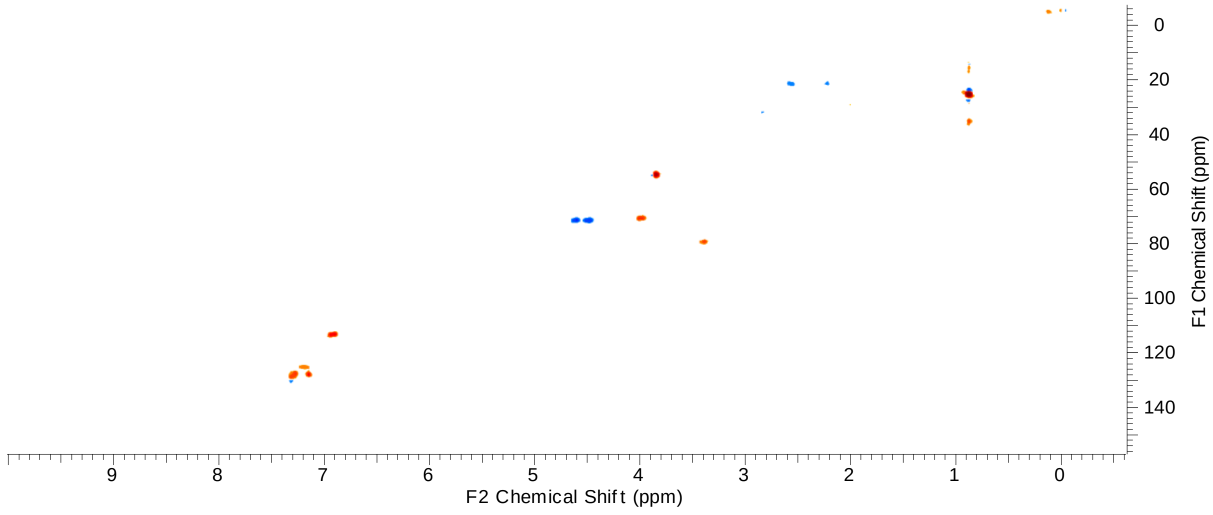

$^{1}H,^{13}C$-HMBC

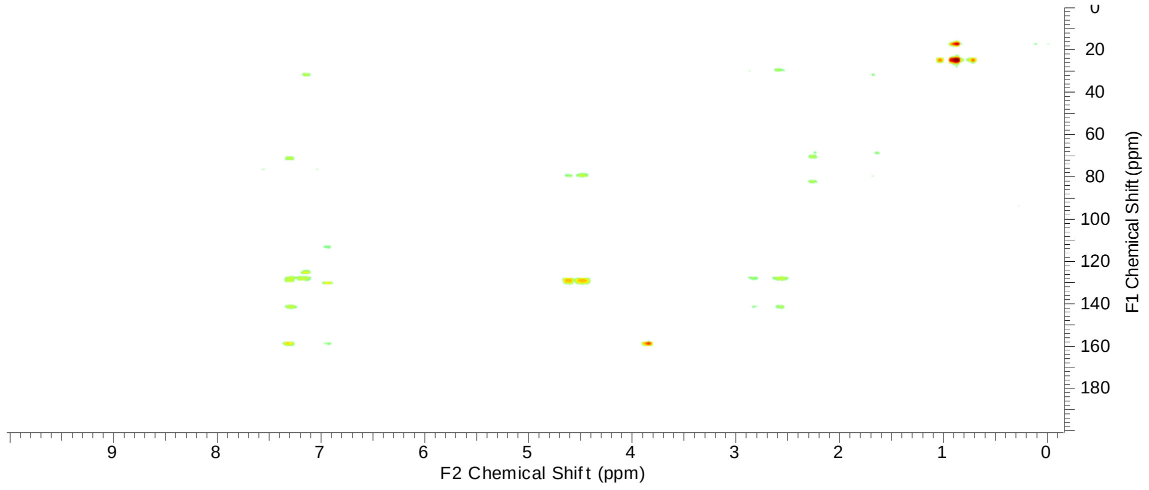

(3*S*,4*S*,*E*)-4-((*tert*-Butyldimethylsilyl)oxy)-7-iodo-1-phenylhept-6-en-3-ol (22)

^{1}H-NMR (400 MHz, $CDCl_3$):

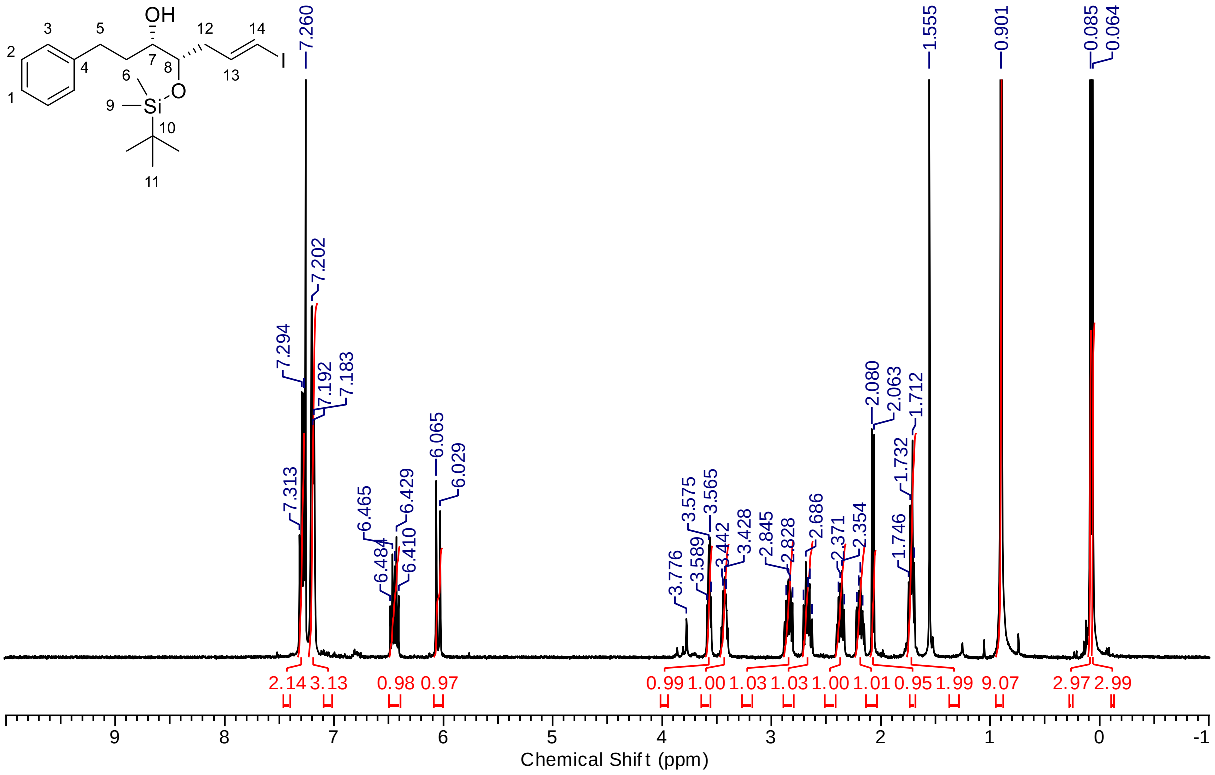

^{13}C-NMR (100 MHz, $CDCl_3$):

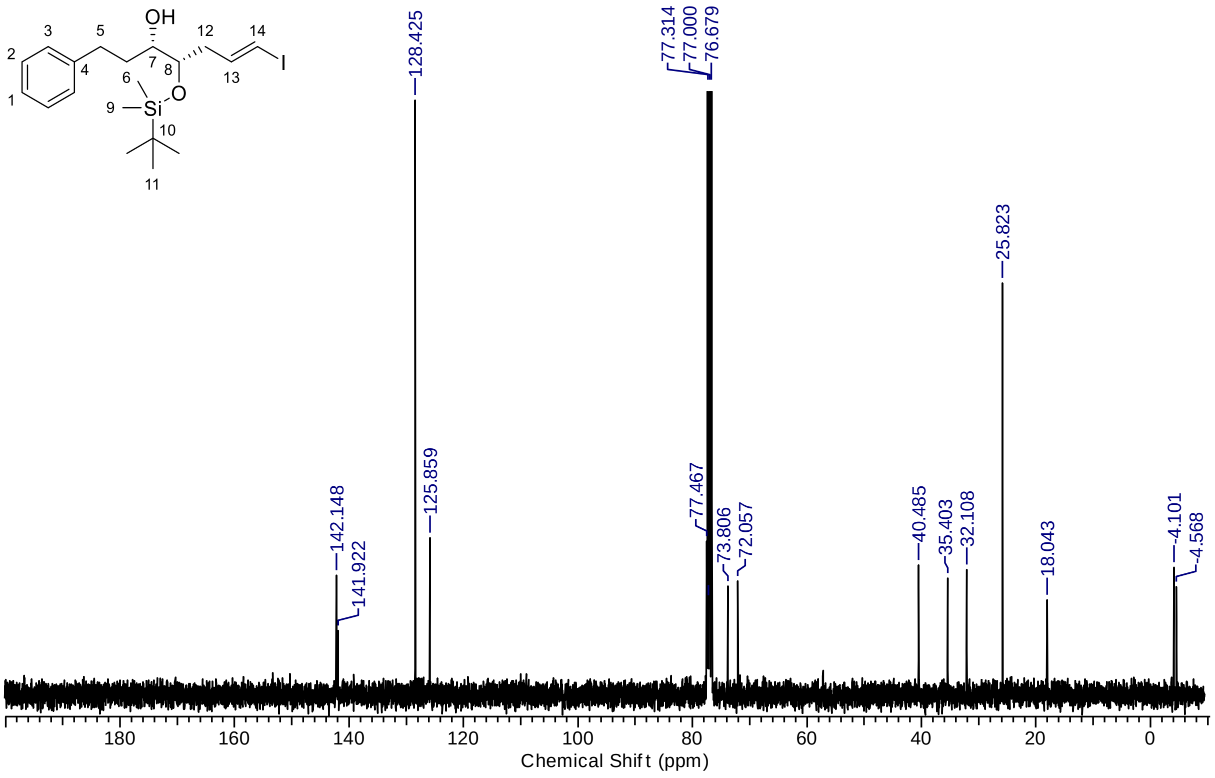

$^{1}H,^{1}H$-COSY

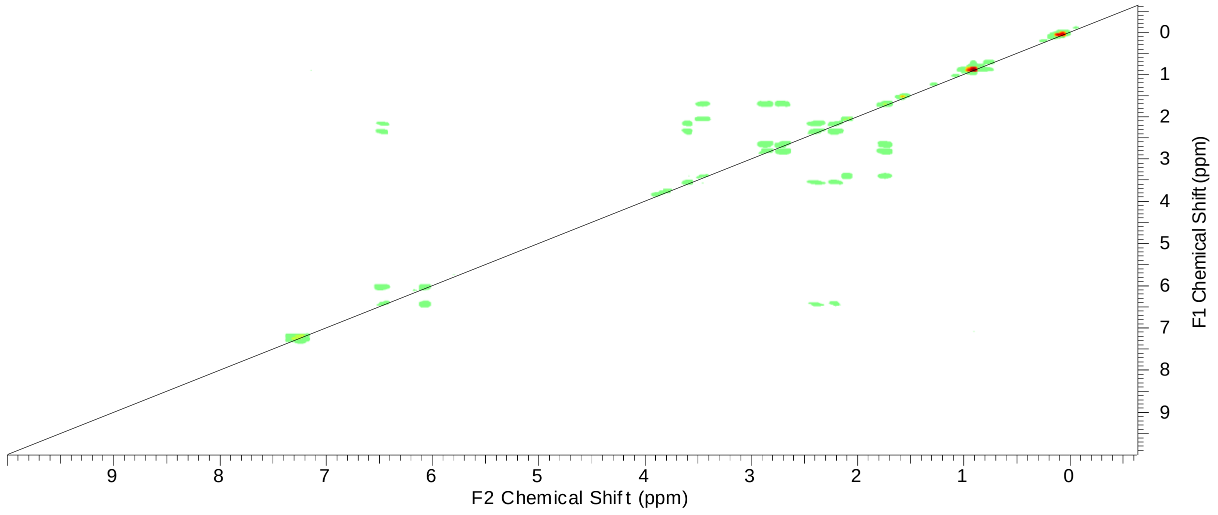

$^{1}H,^{13}C$-HSQC

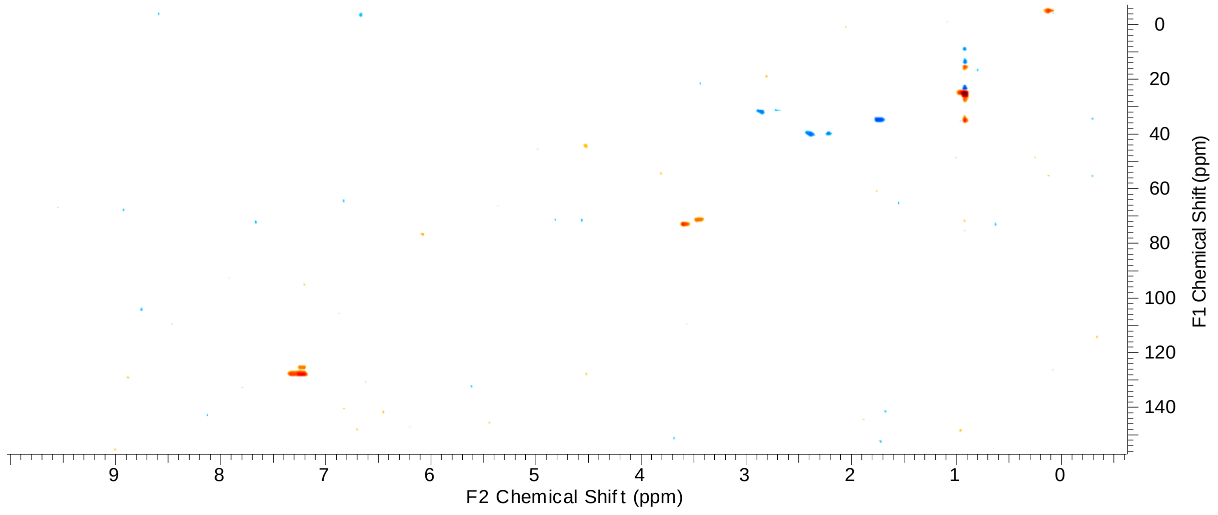

$^{1}H,^{13}C$_HMBC

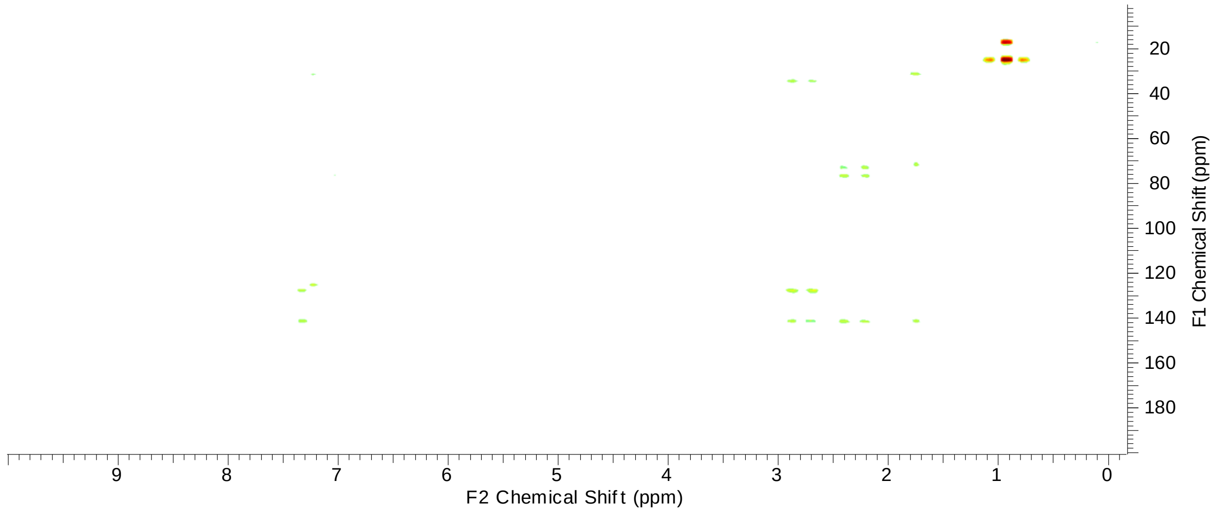

(5*S*,6*S*)-5-((*E*)-3-Iodoallyl)-2,2,3,3,8,8,9,9-octamethyl-6-phenethyl-4,7-dioxa-3,8-disila-decane (23)

^{1}H-NMR (400 MHz, $CDCl_3$):

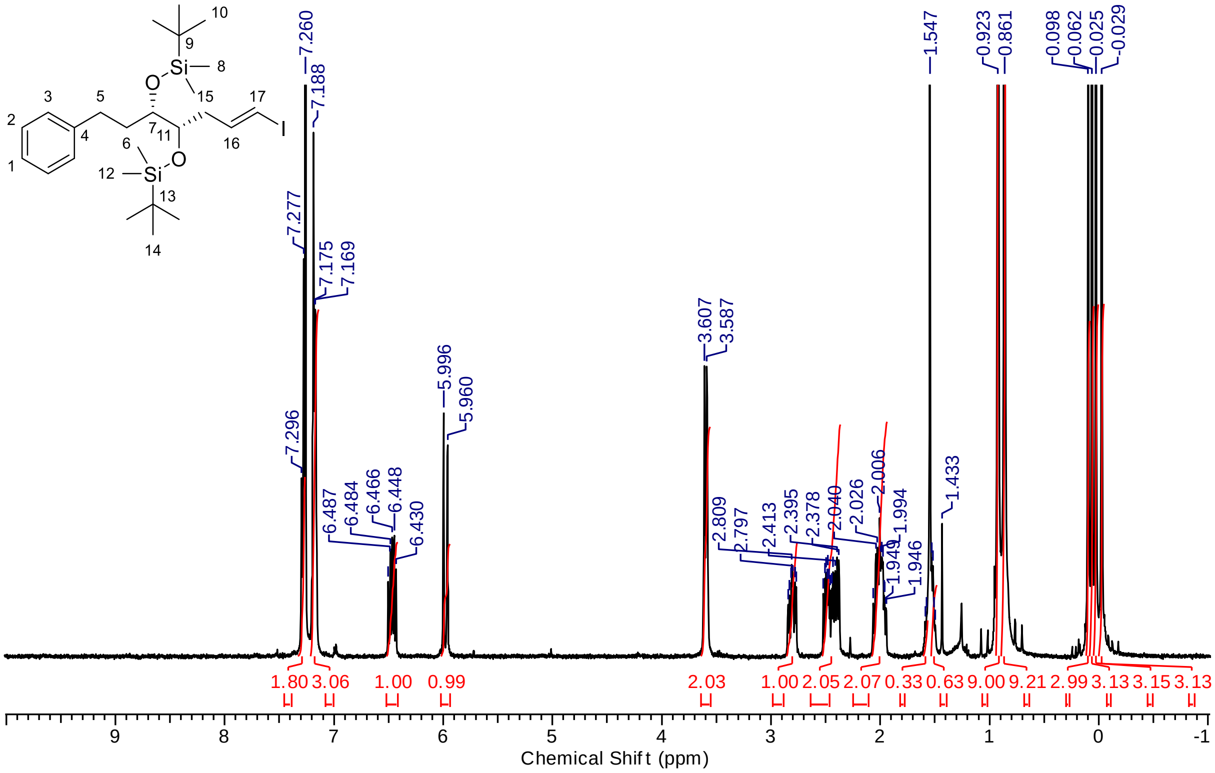

^{13}C-NMR (100 MHz, $CDCl_3$):

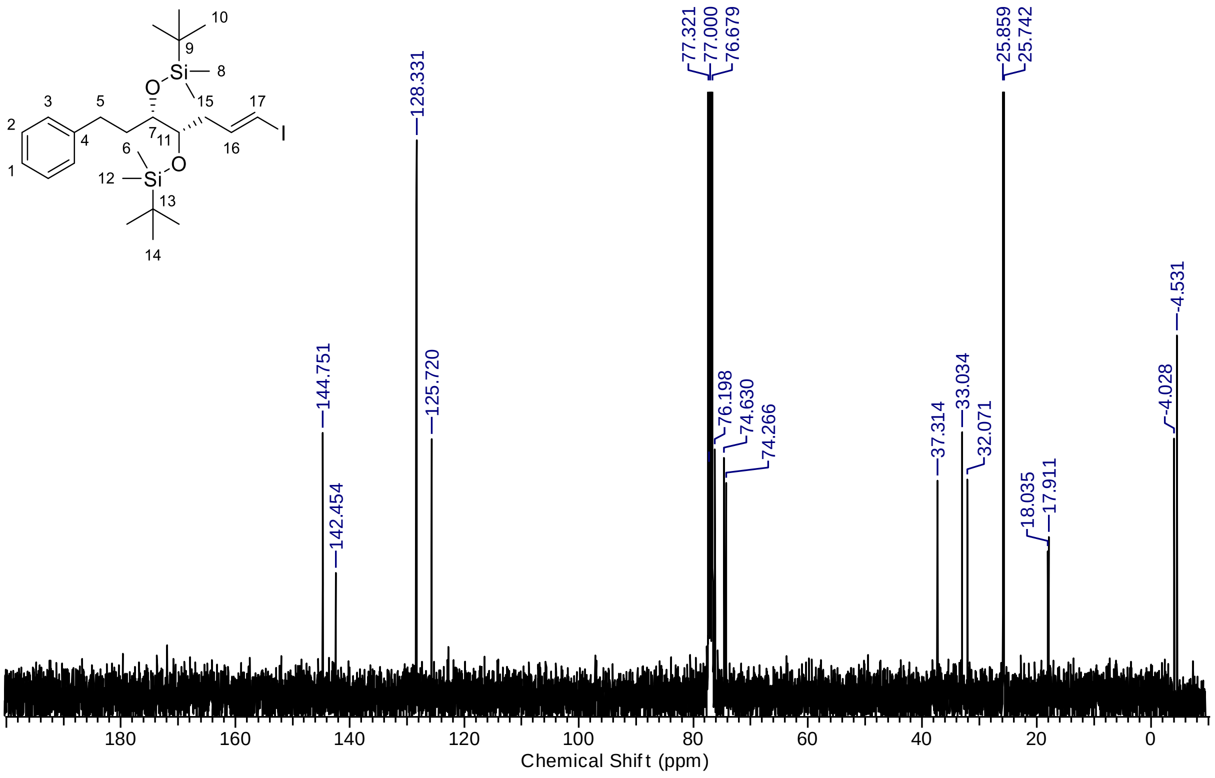

$^{1}H,^{1}H$-COSY

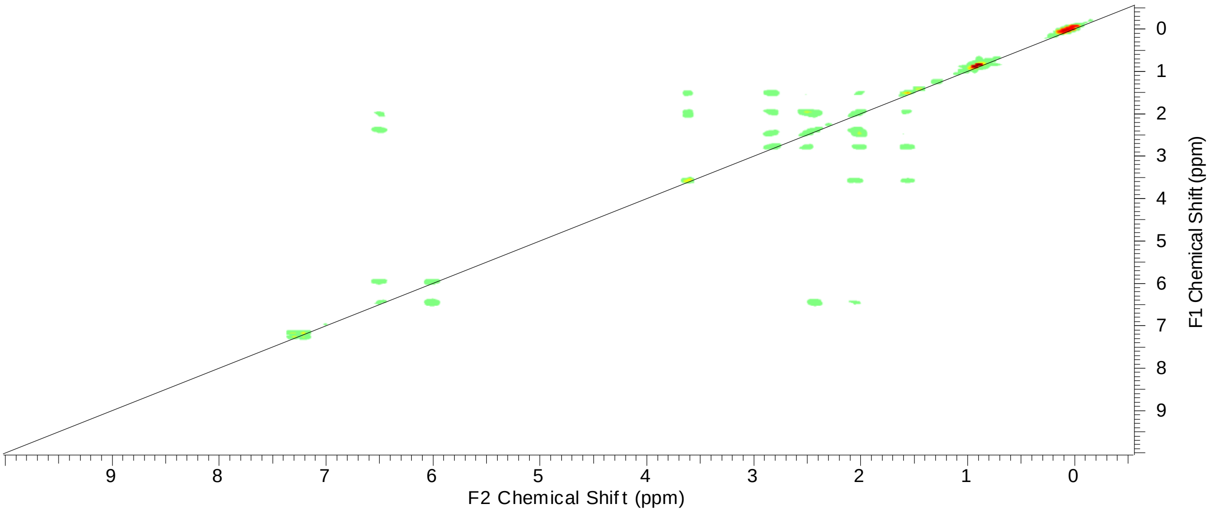

$^{1}H,^{13}C$-HSQC

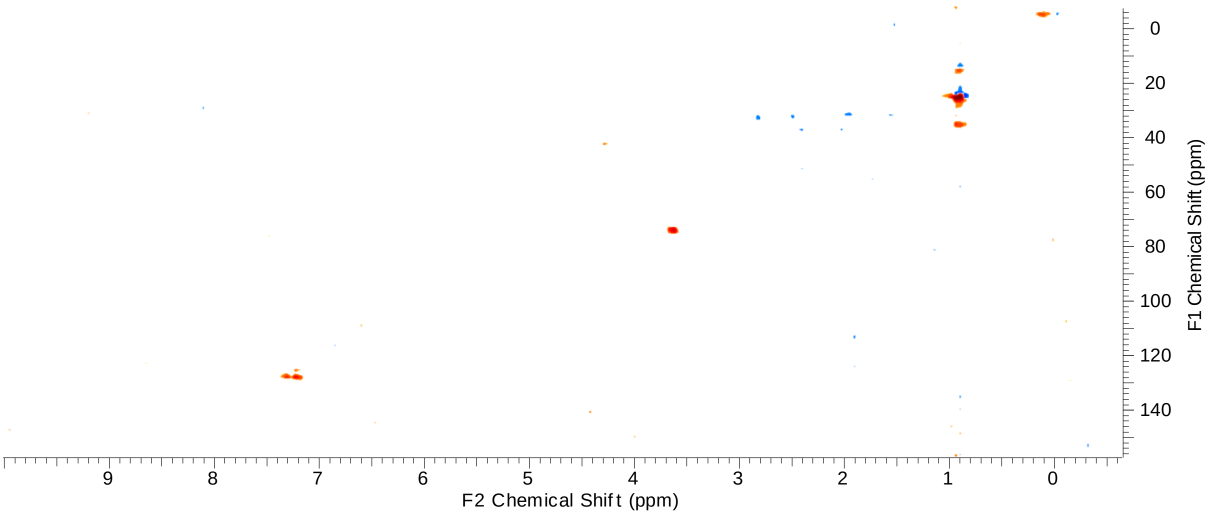

$^{1}H,^{13}C$-HMBC

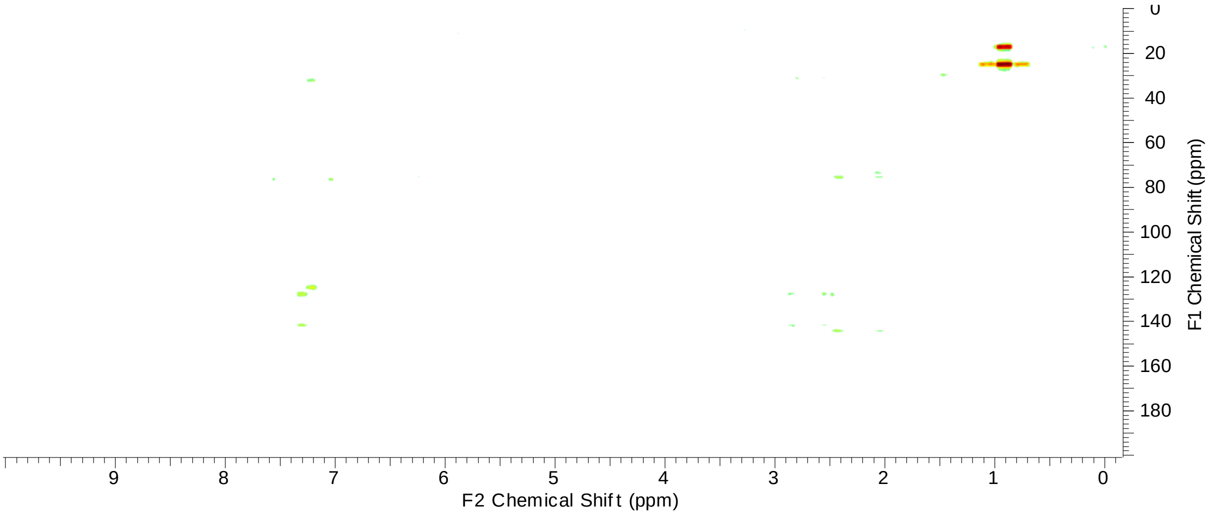

(5*S*,6*S*,8*E*,10*E*,13*R*,14*R*,15*S*)-6-((*tert*-Butyldimethylsilyl)oxy)-13-((4*R*,5*R*)-4,5-dicyclohexyl-1,3,2-dioxaborolan-2-yl)-15-isopropyl-2,2,3,3,14,17,17,18,18-nonamethyl-5-phenethyl-4,16-dioxa-3,17-disilanonadeca-8,10-diene (24)

^{1}H-NMR (400 MHz, $CDCl_3$):

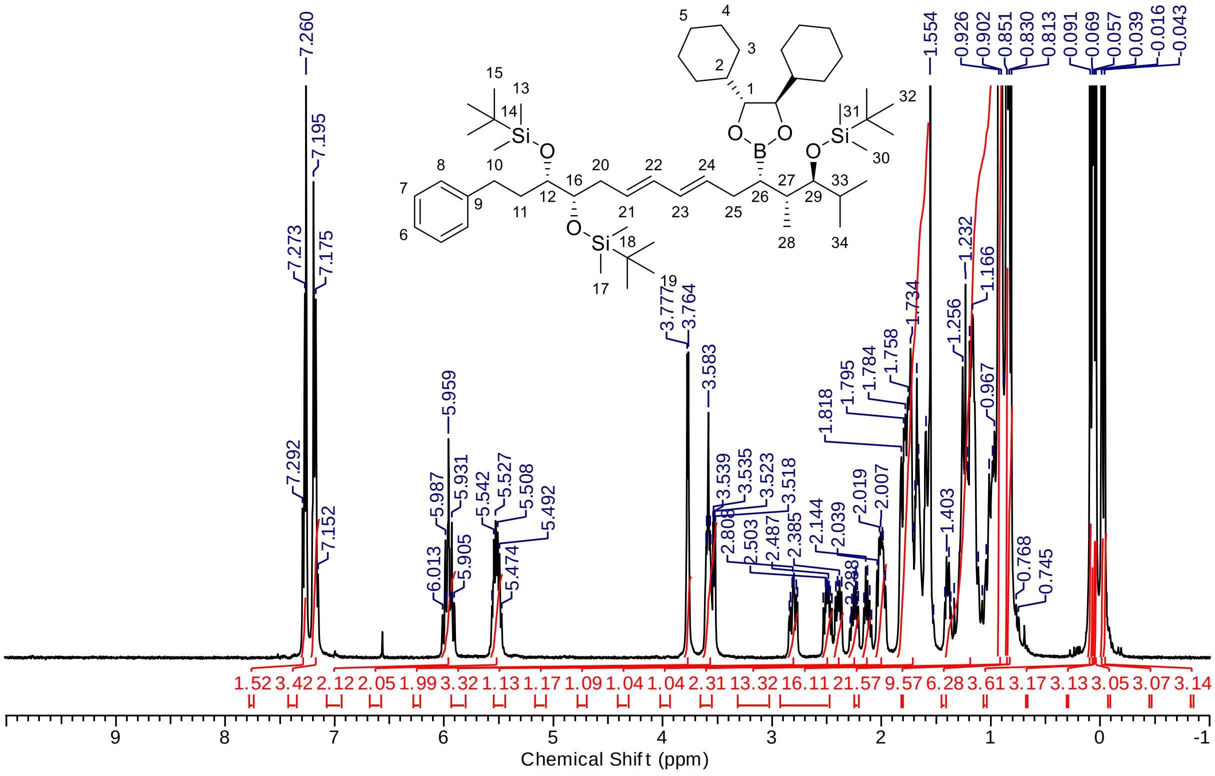

^{13}C-NMR (100 MHz, $CDCl_3$):

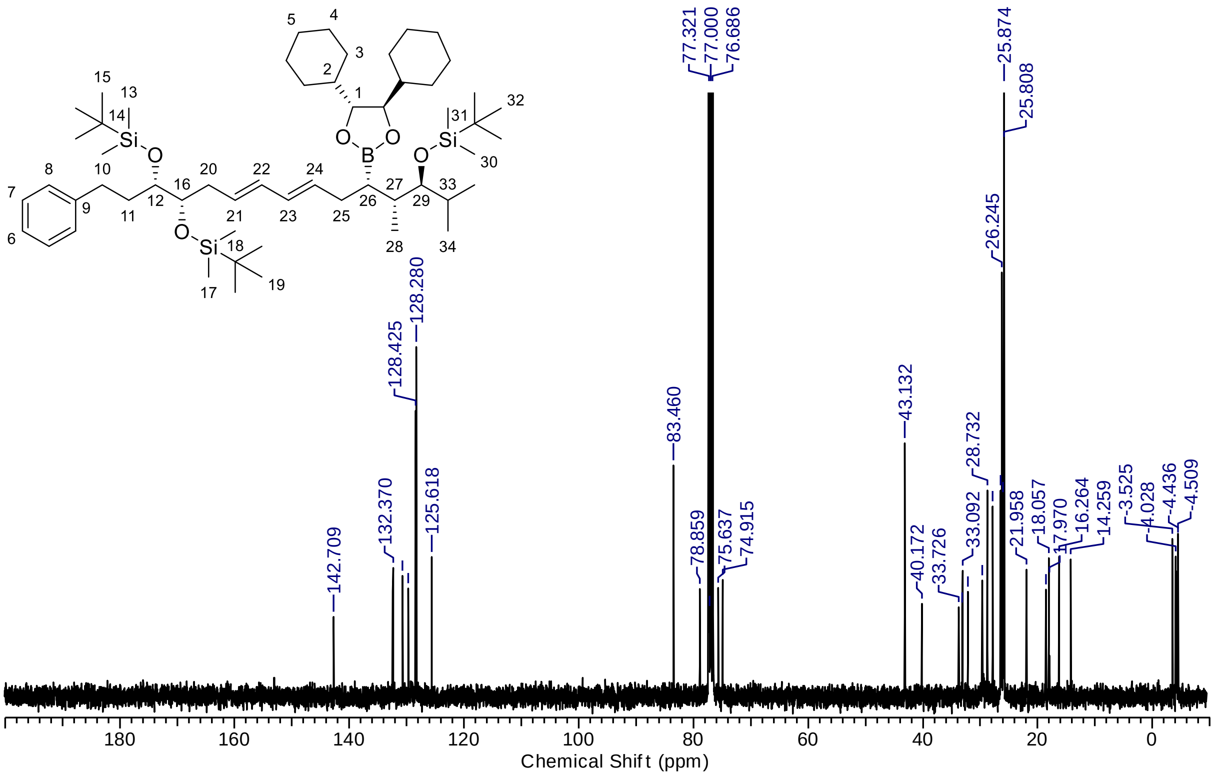

$^{1}H,^{1}H$-COSY

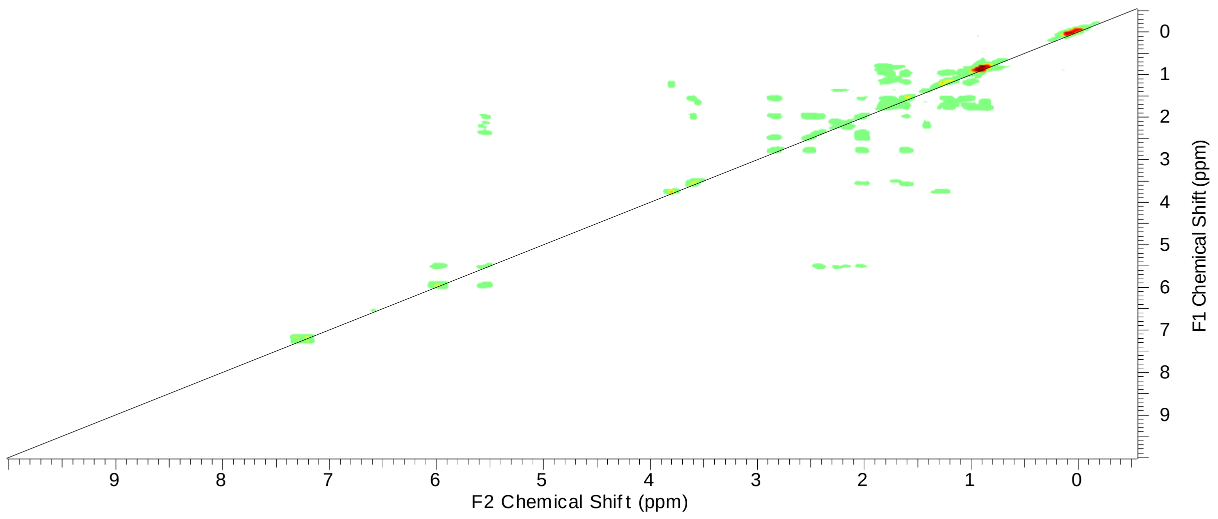

$^{1}H,^{13}C$-HSQC

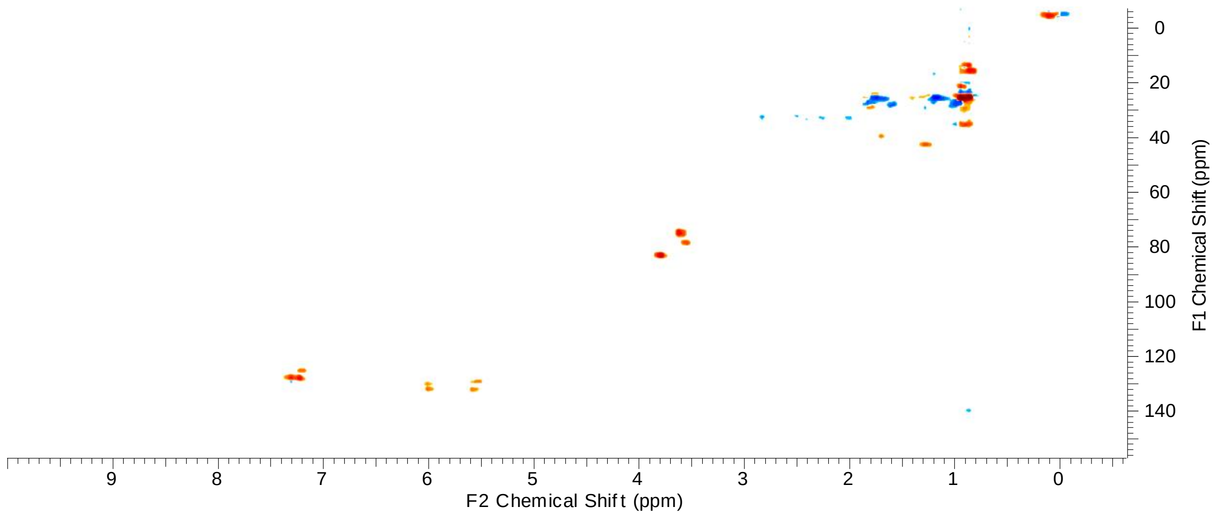

$^{1}H,^{13}C$-HMBC

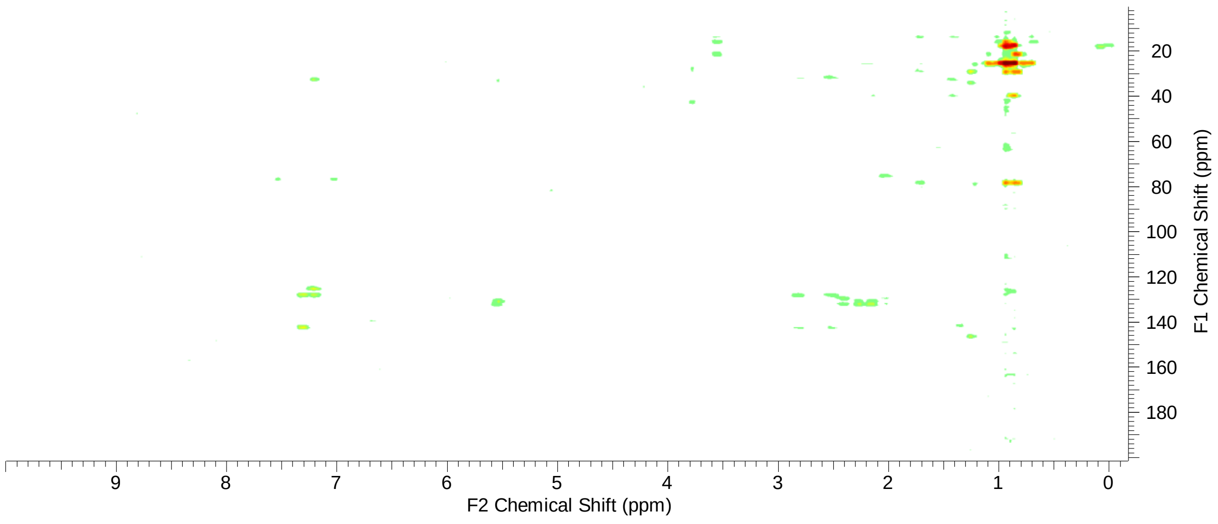

References

[1] A. Krasovskiy, P. Knochel, *Synthesis* **2006**, 890–891.

[2] S. Nave, R. P. Sonawane, T. G. Elford, V. K. Aggarwal, *J. Am. Chem. Soc.* **2010**, *132*, 17096–17098.

[3] T. G. Elford, S. Nave, R. P. Sonawane, V. K. Aggarwal, *J. Am. Chem. Soc.* **2011**, *133*, 16798–16801.

[4] C. Cai, A. Vasella, *Helv. Chim. Acta* **1995**, *78*, 732–757.

[5] V. Navickas, C. Rink, M. E. Maier, *Synlett* **2011**, 191–194.

[6] K. Nakatani, A. Okamoto, T. Matsuno, I. Saito, *J. Am. Chem. Soc.* **1998**, *120*, 11219–11225.

[7] E. M. Carreira, J. Du Bois, *J. Am. Chem. Soc.* **1995**, *117*, 8106–8125.

[8] G. A. Molander, S. R. Wisniewski, *J. Am. Chem. Soc.* **2012**, *134*, 16856.

[9] L. C. Peyrical, M. R. O. Du Berger, M. Boucher, M. Birepinte, J. F. Paquin, A. B. Charette, *Org. Lett.* **2023**, *25*, 2487–2491.

[10] S. G. Aiken, J. M. Bateman, H. H. Liao, A. Fawcett, T. Bootwicha, P. Vincetti, E. L. Myers, A. Noble, V. K. Aggarwal, *Nat. Chem.* **2023**, *15*, 248–256.

[11] S. He, T. J. Senter, J. Pollock, C. Han, S. K. Upadhyay, T. Purohit, R. D. Gogliotti, C. W. Lindsley, T. Cierpicki, S. R. Stauffer, J. Grembecka, *J. Med. Chem.* **2014**, *57*, 1543–1556.

[12] M. Tost, U. Kazmaier, *Chem. Eur. J.* **2025**, *31*, e202500560.

Assigning the absolute configuration of the Matteson homologation products

The absolute configuration of the shown boronic esters is a direct consequence of the mechanism of the Matteson reaction itself. The stereochemical outcome is solely controlled by the chiral diol ligand on boron. The usually very high diastereomeric ratios of the respective products result from sequential double diastereoselection.[1–3] Initially, the lithium carbenoid adds to the boron and forms an ate-complex. Then, zinc chloride is added to the reaction mixture which is complexed by one of the halogen atoms of the former carbenoid and by one of the oxygen atoms of the diol ligand. This can proceed via four possible transition states. **TS1** is thermodynamically more favored as it is by 12.6 kcal/mol lower in energy than the less favored transition states **TS2** and TS3 as confirmed by Midland.[4] TS4 is practically not formed due to steric hindrance. Therefore, the 1,2-migration proceeds more easily through **TS1** than through the others. This energy difference explains the high level of the first step of the diastereoselection.[2,3,5]

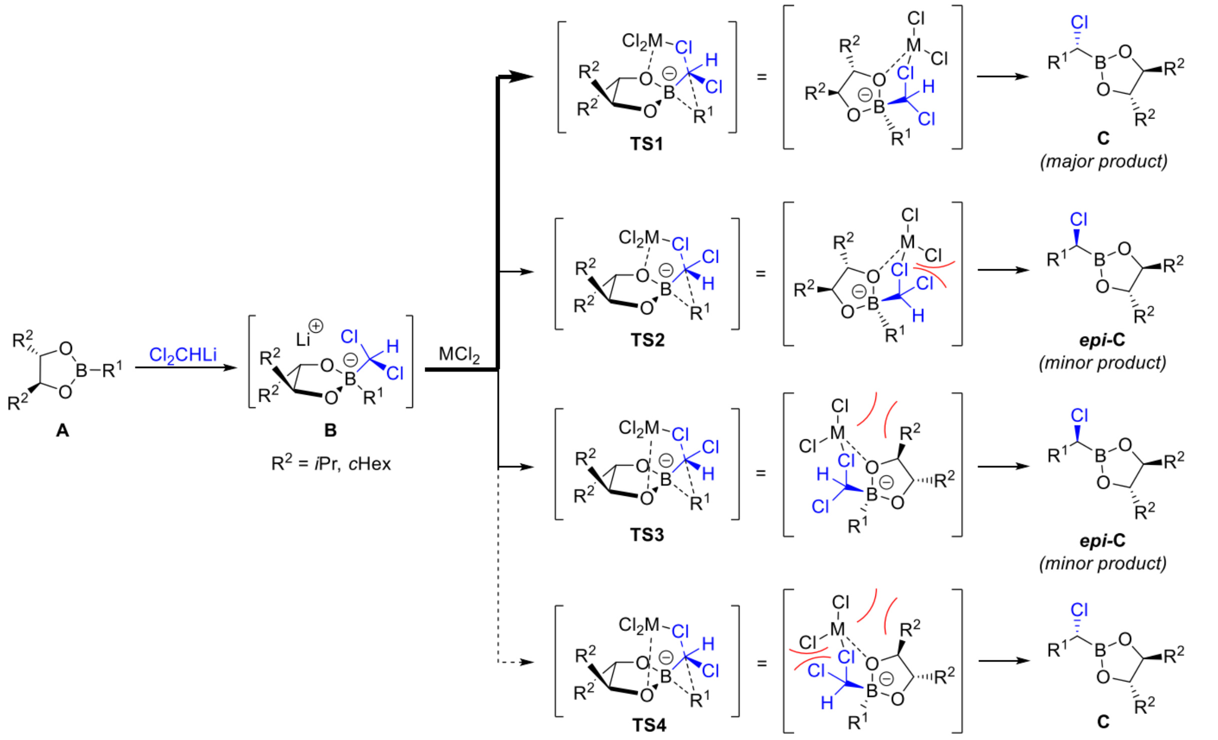

Scheme 1. Mechanism of the Matteson reaction. Extracted from ref. [1].

The next step follows when reacting the α-haloboronic ester with a nucleophile as a Grignard reagent. First, the nucleophile adds to the boron forming an ate-complex. For the following 1,2-rearrangement, the newly introduced nucleophile and the halogen atom complexed by metal salts (either from the step before or from the Grignard reagent or mixtures thereof) have to arrange antiperiplanar as in **TS5**. This transition state benefits from the chelation of the metal cation by the sterically less hindered oxygen of the diol, binding the leaving group to the metal (and facilitating the displacement) and minimizing the steric interaction. So, product **E** is formed.[2,3,6]

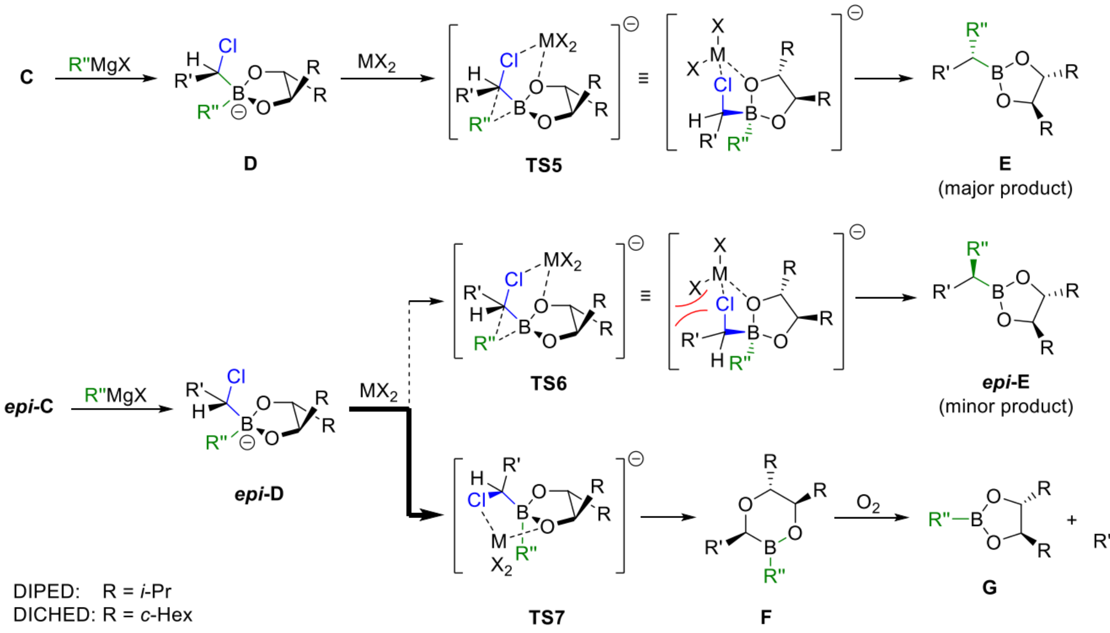

Scheme 2. Nucleophilic displacement of the α-haloboronic ester as part of the sequential double diastereoselection. Extracted from ref. [1].

When starting with *epi*-**C**, the antiperiplanar arrangement of halogen and introduced residue results in destabilizing steric interaction. So, the rearrangement from **TS6** hardly takes place. In this case, **TS7** is formed more preferably. But here, the halogen atom is no longer antiperiplanar to the introduced residue but to the diol. So, migration of the diol oxygen rather takes place by expanding the ring to **F**. This borinic ester decomposes upon air oxidation to the boronic ester **G** and the aldehyde **H**. As **C** and epi-**C** perform very differently in this step, diastereoselectivity is furtherly improved.

This insight into the mechanism demonstrates that just one absolute configuration is accessible and formed as major product after sequential double diastereoselection. Furtherly, it shows that the configuration of the migrating group is retained and the configuration of the carbon at the displacement center (which is introduced as carbenoid at the very beginning) is inverted. This has first been shown and confirmed by Matteson *et al.*[6–9] and since then in many other applications of the Matteson reaction.[1,7,18–21,10–17] On this base, the assignment of the absolute configuration of the boronic esters shown in the manuscript follows careful mechanistic considerations and is state of the art in the field of Matteson reaction. All literature cited in this statement is also cited in the introduction of the manuscript or is part of reviews which are cited therein. So, an interested reader is able to rapidly find the literature. Not to overload the introduction, the considerations of his statement are not described that detailed as herein.

References:

[1] U. Kazmaier, *Mar. Drugs* **2025**, *23*, 20.

[2] D. S. Matteson, B. S. L. Collins, V. K. Aggarwal, E. Ciganek, in *Org. React.*, **2021**, pp. 427–860.

[3] D. S. Matteson, *J. Org. Chem.* **2013**, *78*, 10009–10023.

[4] M. M. Midland, *J. Org. Chem.* **1998**, *63*, 914–915.

[5] E. J. Corey, D. Barnes-Seeman, T. W. Lee, *Tetrahedron Asymmetry* **1997**, *8*, 3711–3713.

[6] P. B. Tripathy, D. S. Matteson, *Synthesis* **1990**, 200–206.

[7] D. S. Matteson, K. M. Sadhu, M. L. Peterson, *J. Am. Chem. Soc.* **1986**, *108*, 810–819.

[8] D. S. Matteson, L. Peterson, *J. Org. Chem.* **1987**, *52*, 5116–5121.

[9] D. S. Matteson, A. A. Kandil, *J. Org. Chem.* **1987**, *52*, 5121–5124.

[10] J. Gorges, U. Kazmaier, *Org. Lett.* **2018**, *20*, 2033–2036.

[11] M. Tost, O. Andler, U. Kazmaier, *Eur. J. Org. Chem.* **2021**, *2021*, 6459–6471.

[12] O. Andler, U. Kazmaier, *Org. Biomol. Chem.* **2021**, *19*, 4866–4870.

[13] O. Andler, U. Kazmaier, *Org. Lett.* **2021**, *23*, 8439–8444.

[14] T. Kinsinger, U. Kazmaier, *Org. Lett.* **2022**, *24*, 3599–3603.

[15] O. Andler, U. Kazmaier, *Org. Lett.* **2022**, *24*, 2541–2545.

[16] A. Horn, E. Papadopoulos, T. Kinsinger, J. Greve, E. Bickel, S. Pachoula, U. Kazmaier, *Z. Anorg. Allg. Chem.* **2024**, 1–5.

[17] M. Tost, U. Kazmaier, *Mar. Drugs* **2024**, *22*, 165.

[18] D. Leonori, V. K. Aggarwal, *Acc. Chem. Res.* **2014**, *47*, 3174–3183.

[19] M. Kempf, O. Andler, U. Kazmaier, *Helv. Chim. Acta* **2023**, *106*, 1–9.

[20] T. Kinsinger, P. Schäfer, U. Kazmaier, *Org. Lett.* **2023**, *25*, 3303–3307.

[21] M. Tost, U. Kazmaier, *Org. Lett.* **2023**, *25*, 6835–6839.
